# Palladium-Catalyzed *N*-Alkenylation of *N*-Aryl Phosphoramidates with Alkenes

**DOI:** 10.3390/molecules28114466

**Published:** 2023-05-31

**Authors:** Yu-An Li, Ge Wu, Jia Li

**Affiliations:** 1 Department of Orthopaedics Surgery, The Second Affiliated Hospital and Yuying Children’s Hospital of Wenzhou Medical University, Wenzhou 325000, China; 2 School of Pharmaceutical Sciences, Wenzhou Medical University, Wenzhou 325015, China; 3 Department of Neurology, First Affiliated Hospital of Wenzhou Medical University, Wenzhou 325015, China

**Keywords:** palladium-catalyzed, oxidative amination, phosphoramidates, alkenes, O_2_

## Abstract

Versatile and concise Pd-catalyzed oxidative *N*-alkenylation of *N*-aryl phosphoramidates with alkenes is described in this study, a reaction that is of great significance but surprisingly unexploited. The transformation proceeds under mild reaction conditions, using O_2_ as a green oxidant and TBAB as an effective additive. An efficient catalytic system allows a variety of drug-related substrates to participate in these transformations, which is of great interest in the drug discovery and development of phosphoramidates.

## 1. Introduction

Multi-functionalized phosphoramidates represent a promising class of phosphorous compounds and have fascinating applications in the pharmaceutical industry, as flame retardants, and in asymmetric catalysis [1,2]. In medicinal chemistry, the late-stage modification of phosphoramidates skeletons led to the discovery of plentiful FDA-approved drugs [3,4], such as Tenofovir Alafenamide (for HBV), Sofosbuvir (a blockbuster drug for HCV) and Thymectacin (for cancer therapy). Recently established, Redesivir is a new broad-spectrum antiviral drug developed by Gilead, which has been effectively used to treat COVID-19 [5]. However, accessible synthetic approaches for *N*-modification of *N*-aryl phosphoramidates have surprisingly not been developed. To date, only alkylation with α, β-unsaturated ketones [6], allenylation with propargyl alcohols [7], intramolecular allylation reaction [8], and alkynylation with alkynyl bromides [9] have been reported to remould these privileged organophosphorus compounds. Therefore, the development of novel and ingenious methods to realize *N*-diversification of phosphoramidates based on an atom-economic strategy is still highly desirable.

**Scheme 1:**
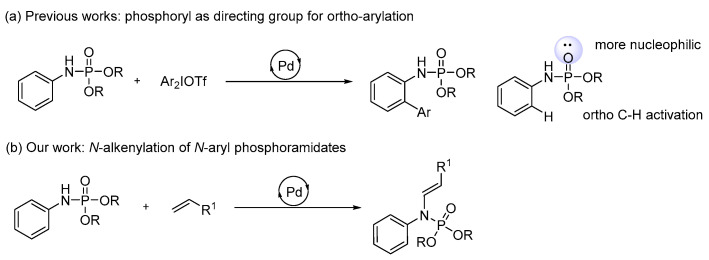
Tuning the reactivity of *N*-aryl phosphoramidates.

In recent years, palladium-catalyzed intermolecular oxidative amination of alkenes with anilines has been used to synthesize complex enamines [10,11,12,13], but it is unsuitable for electron-deficient aniline. Metal-catalyzed direct alkenylation of *N*-aryl phosphoramidates with alkenes represents a significant but challenging assignment, mainly due to the special structural characteristics of the *N*-aryl phosphoramidates. (1) The phosphoryl could function as a directing group, which easily gives rise to achieving the ortho functionalization of the aniline moiety, as demonstrated by palladium-catalyzed ortho-arylation of aniline with di(aryl)iodium triflate (Scheme 1a) [14]. (2) There is a large steric hindrance between the adjacent phosphoryl and aniline, which would weaken the N–H activation of *N*-aryl phosphoramidates with transition-metal catalysts. (3) The good affinity between phosphoramidates and the transition-metal centre would reduce the catalyst’s Lewis acidity [15,16], thus inhibiting the activation of alkenes. According to the theory of soft and hard acid-base, we envisioned that excessive soft ligands could occupy the coordination space of transition metal ions, thus reducing the chance of chelating phosphoramidate with the transition metal. In that case, it is expected to overcome the use of phosphoramidate as a directing group for the inevitable ortho C–H alkenylation of the aniline moiety [17,18] and to avoid the deactivation of the catalyst. Based on our long-term interest in alkene functionalization [19,20,21], herein, we describe a concise palladium-catalyzed oxidative coupling of *N*-aryl phosphoramidates with alkenes with excellent regio-and stereoselectivity, in which it was found that appropriate LiOAc and TBAB are essential for the successful transformation (Scheme 1b).

## 2. Results and Discussion

To assess the *N*-alkenylation of *N*-aryl phosphoramidates, diphenyl p-tolylphosphoramidate **1a,** and ethyl acrylate **2a** were chosen as substrates to explore the optimal reaction conditions, as summarized in Table 1 (see more details in Supporting Information). Initially, some additives, such as Cu(OAc)_2_, CuCl_2_, BQ, pyridine and LiBr, were evaluated. These additives are usually used in palladium-catalyzed alkene oxidative amination reactions but did not respond to this transformation (Entries 1–5). Fortunately, when the reaction was conducted using Pd(OAc)_2_ as a catalyst, TBAB as an additive and LiOAc as a suitable base, **3a** was obtained in a 65% yield in DME at 80 °C under an O_2_ atmosphere (Entry 6). Then, we further examined the common phase transfer catalysts. However, the addition of TBAF, TBAC and TBAI completely inhibited the reactions (Entries 7–9). Various ether solvents were screened. Using TMBE instead of DME could improve the yield of the desired product to 80% (Entry 10). The examination of different palladium salts showed that Pd(TFA)_2_ has higher catalytic activity than Pd(OAc)_2_ (entry 13), and the yield of **3a** is raised to 89%. Whereafter, examination of different ligands (such as pyridine and phthalimide), previously proved in palladium-catalyzed aza-Wacker reactions [22,23,24,25], which not only failed to improve the reaction efficiency but it was also detrimental for this transformation (Entries 11, 12). Other Pd(II) catalysts, such as PdCl_2_, Pd(CH_3_CN)_2_Cl_2_, and Pd(PhCN)_2_Cl_2,_ can not trigger the transformation, likely to be the difficulty in ligand exchange between the chloride ion and *N*-aryl phosphoramidates. Finally, when the reaction was performed under an N_2_ atmosphere (Entry 14), **3a** was isolated in only 7% of the yield, and the formation of palladium black indicated that O_2_ was used as an oxidant to regenerate the active palladium catalyst.

**Scheme 2:**
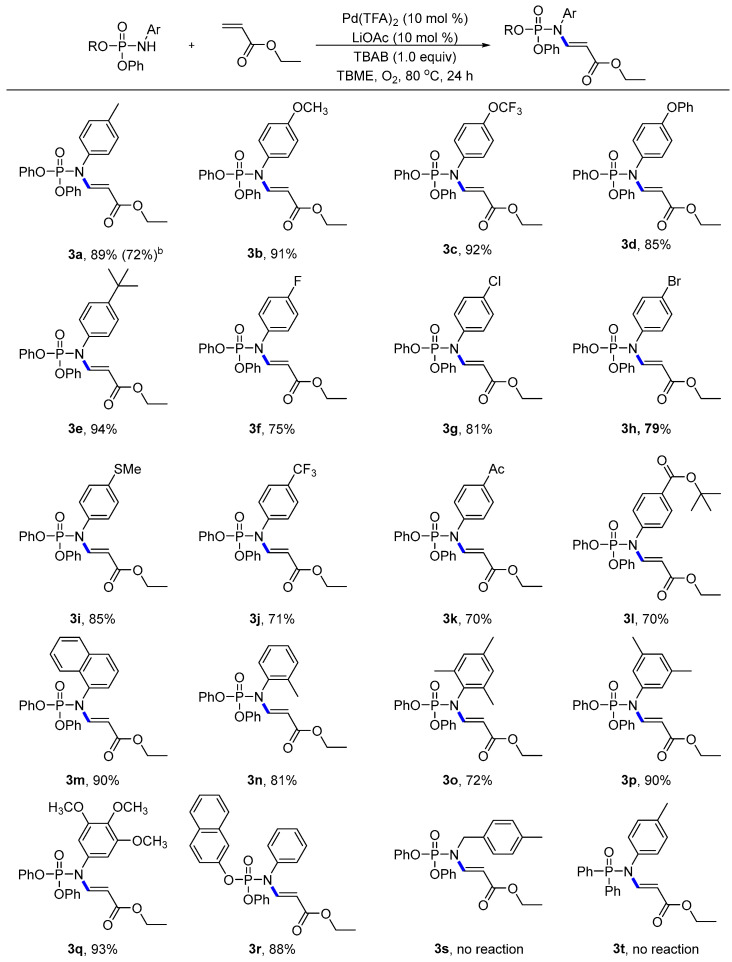
Scope of Phosphoramidates. Reaction conditions: phosphoramidates (0.2 mmol), ethyl acrylate (0.6 mmol), Pd(TFA)_2_ (0.02 mmol), LiOAc (0.02 mmol), TBAB (0.2 mmol), TBME (2 mL) under O_2_, heated at 80 °C for 24 h. ^b^ 5 mmol scale.

As illustrated in Scheme 2, palladium-catalyzed direct cross-coupling of *N*-aryl phosphoramidates with ethyl acrylate displayed spectral functional group compatibility. Various anilines of phosphoramidates bearing electron-donating substituents, such as methyl (**3a**), methoxy (**3b**, **3q**), trifluoromethoxy (**3c**), phenoxy (**3d**), and tertiary butyl (**3e**), and smoothly participated in these transformations to obtain the target products in excellent yields. Phosphoramidates with electron-withdrawing groups on aniline, such as trifluoromethyl (**3j**), acetyl (**3k**) and ester (**3l**), could be efficiently converted into the corresponding *N*-vinyl phosphoramidates with useful yields. Generally, *N*-aryl phosphoramidates bearing electron-donating substituents show much higher reactivity than that with electron-withdrawing counterparts. The current catalytic system could be compatible with important halogen functional groups (**3f**–**3h**), which provides an important synthetic tool for further derivatization. Remarkably, methylthio (**3i**) is sensitive to palladium catalysts because of its strong coordination ability and small steric hindrance, and it is also feasible under standard reaction conditions. As expected, the position of the substituent in the aniline ring of *N*-aryl phosphoramidates affected the efficiency of transformation, in the cases of diphenyl o-tolylphosphoramidate (**3n**) and diphenyl mesitylphosphoramidate (**3o**). Diphenyl naphthalen-1-ylphosphoramidate (**3m**) also worked in the same way and exhibited wonderful reaction efficiency. Naphthalen-2-yl phenyl *N*-phenyl phosphoramidate (**3r**) was subjected to the *N*-vinylation coupling reaction, which had predictable regioselectivity and good yield. However, the use of diphenyl (4-methylbenzyl)phosphoramidate (**3s**) and P,P-diphenyl-N-(p-tolyl)phosphinic amide (**3t**) as substrates failed to give the corresponding *N*-alkenylation product. These experimental results show that aryloxy on the P atom and aryl group on the N atom of phosphoramidate are indispensable for reaction efficiency. Importantly, the 5 mmol scale reaction progressed smoothly, and a satisfactory yield of **3a** could be obtained.

**Scheme 3:**
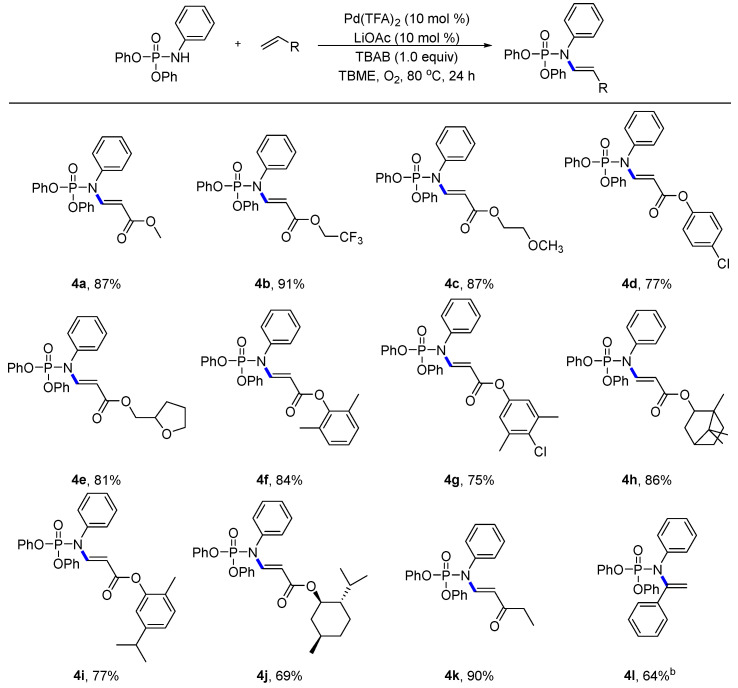
Scope of various alkenes. Reaction conditions: diphenyl *N*-phenyl phosphoramidate (0.2 mmol), alkenes (0.6 mmol), Pd(TFA)_2_ (0.02 mmol), LiOAc (0.02 mmol), TBAB (0.2 mmol), TBME (2 mL) under O_2_, heated at 80 °C for 24 h. ^b^ Reaction conditions: Pd(TFA)_2_ (10 mol%), Cu(OAc)_2_ (20 mol%), LiOAc (20 mol%), DME as solvent, under O_2_, heated at 80 °C for 24 h.

Subsequently, we turned our attention to the scope of acrylate. As shown in Scheme 3, various functionalized acrylates show good reactivity. Trifluoromethyl (**4b**), methoxyl (**4c**), halogen (**4d**) and tetrahydrofuran-2-yl (**4e**) could be well accommodated and afforded the respective products. The compound 2-methoxyethyl acrylate, mainly used as an adhesive, could undergo selective amination to gain **4c** in a good yield. Isobornyl acrylate, a monomer of acrylic resins, was easily transformed into the anticipated product **4h**. Furthermore, this *N*-alkenylation of *N*-phenyl phosphoramidate could also be applied for the late-stage modification of important medical agents. Acrylate alkenes derived from 2,6-dimethylphenol (**4f,** food spices), Chloroxylenol (**4g**, disinfector), Carvacrol (**4i**, local anaesthetic) and D-Menthol (**4j**, excitants) all worked well in the abovementioned protocols. It is undeniable that ethyl methacrylate and ethyl crotonate did not work at all under the present catalytic systems, suggesting that a substituent on a carbon carbon double bond had an obvious steric hindrance effect on alkene amination. Pleasingly, the treatment of α, β-unsaturated ketone also gave a good yield of the anticipated product (**4k**). Having extended the scope of electron-deficient alkenes with *N*-aryl phosphoramidates, we further examined styrene oxidative amination. Through systematic optimization of the reaction conditions, it was found that the direct cross-coupling of styrene with diphenyl *N*-phenyl phosphoramidate could be realized with the combination of Pd(TFA)_2_ with catalytic amounts of Cu(OAc)_2_ and 20 mol% LiOAc as the base, which is an important route for the preparation of 1,1-disubstituted alkenes. In addition, the anti-Markovniknov product originates from the possibility that the palladium centre coordinates with styrene to give a relatively stable benzyl carbocation intermediate.

**Scheme 4:**
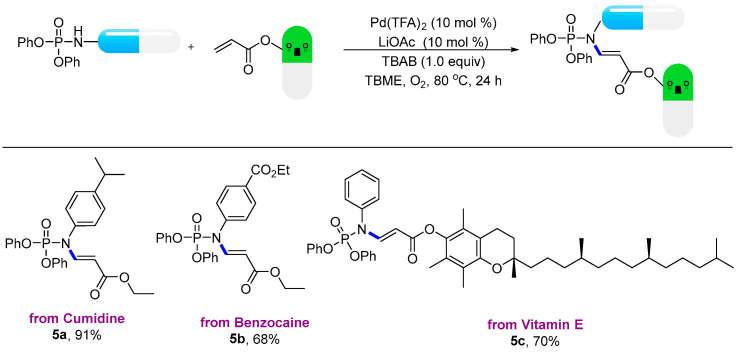
Late-stage vinylation of bioactive relevant *N*-aryl phosphoramidates.

The synthetic strategy was demonstrated in the incorporation of pharmaceutical molecules into *N*-alkenyl-*N*-aryl phosphoramidates (Scheme 4). *N*-aryl phosphoramidates derived from Cumidine (herbicide) and Benzocaine (local anaesthetics) were chosen as representative cases and treated with ethyl acrylate to provide the desired **5a** and **5b**, respectively. Furthermore, a vitamin E-derived electron-deficient alkene (**5c**) was also prepared and subjected to region-selective amination, providing the corresponding product with high reaction efficiency. 

**Scheme 5:**
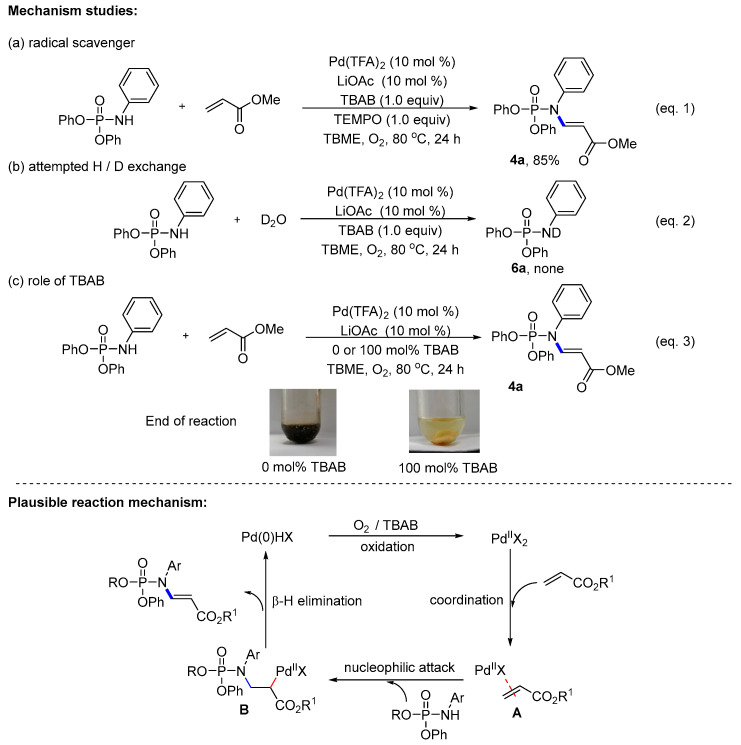
Mechanistic findings and plausible reaction mechanism.

Some experiments were performed to elucidate the oxidative cross-coupling reaction pathway between *N*-aryl phosphoramidates and alkenes (Scheme 5). Firstly, 1.0 equivalent TEMPO was added into the optimal reaction conditions, and the **4k** was obtained in an 85% yield (Equation (1)), which suggests that a radical pathway could be ruled out. Secondly, we did not observe any H/D exchange product (Equation (2)), which further excludes the first N–H activation of *N*-aryl phosphoramidates with a palladium catalyst. Finally, the formation of palladium black was observed in the absence of TBAB, whereas the reaction solution was always bright yellow in its presence. This result suggests that TBAB plays a role in stabilizing and assisting the oxidization of Pd (0) [26,27]. According to these experimental results and previous reports, a typical mechanism of palladium-catalyzed alkene chemistry is proposed in Scheme 4. Initially, the coordination of palladium salt with alkenes forms palladium–alkene adducts **A**, following the nucleophilic attack of *N*-aryl phosphoramidates to produce the alkylpalladium complex **B**. The subsequent β–H elimination of intermediate **B** generates the anticipated products. Finally, the Pd (0) species can be facilely oxidized to viable Pd (II) under 1 atm of O_2_, thus realizing the catalytic cycle. 

In summary, the versatile palladium-catalyzed oxidative cross-coupling of *N*-aryl phosphoramidates with alkenes has been discovered, providing a concise avenue by which to access structurally rich *N*-aryl-*N*-vinyl phosphoramidates. The outstanding attractions of these transformations are the use of O_2_ as a green oxidant, a cocatalyst-free catalytic system, and late-stage functionalization of important pharmaceuticals as well as readily accessible starting materials. In view of the wide application of phosphoramidates in medical chemistry, this concise synthetic method provides a practical tool for accelerating the discovery of phosphoramidates drugs.

## 3. Materials and Methods

### 3.1. General Information

Phosphoramidates and acrylates were prepared according to the reported procedures. The ^1^H and ^13^C spectra of known compounds were in accordance with those described in the literature. All other reagents were purchased from TCI (Shanghai, China), Sigma-Aldrich (Shanghai, China), Alfa Aesar (Shanghai, China), Acros (Shanghai, China), and Meryer (Shanghai, China) and used without further purification. The ^1^H NMR (500 MHz), ^13^C NMR (125 MHz) and ^19^F NMR (470 MHz) spectra were recorded in CDCl_3_ and DMSO-D6 solutions using a Burker AVANCE 500 spectrometer. High-resolution mass spectra were recorded on an ESI-Q-TOF mass spectrometer. Analysis of the crude reaction mixture was conducted on the Varian 4000 GC/MS and 1200 LC. All reactions were conducted using standard Schlenk techniques. Column chromatography was performed using EM silica gel 60 (300–400 m).

### 3.2. General Procedure of Palladium-Catalyzed N-Alkenylation of Phosphoramidates with Alkenes

A 25 mL Schlenk tube equipped with a stir bar was charged with phosphoramidates (0.2 mmol), alkenes (0.6 mmol), Pd(TFA)_2_ (0.02 mmol), TBAT (0.2 mmol) and TBME (0.06 mmol). The tube was fitted with a rubber septum, and then it was evacuated and refilled with dioxygen three times; then, the septum was replaced with a Teflon screwcap under oxygen flow. The reaction mixture was stirred at 80 °C for 24 h. After cooling down, the reaction mixture was diluted with 10 mL of ethyl ether, filtered through a pad of silica gel, followed by washing the pad of the silica gel with the same solvent (20 mL), concentrated under reduced pressure. The residue was then purified by flash chromatography on silica gel to provide the corresponding product.

### 3.3. Characterization of Products in Details

Compound: *ethyl (E)-3-((diphenoxyphosphoryl)(p-tolyl)amino)acrylate* (**3a**): Following the general procedure, using (petroleum ether:EtOAc = 9:1) as the eluant afforded a colourless liquid (77.8 mg, 89% yield). ^1^H NMR (400 MHz, CDCl_3_): δ 8.31 (dd, *J* = 13.7, 7.8 Hz, 1H), 7.36 (t, *J* = 7.8 Hz, 4H), 7.23 (d, *J* = 7.7 Hz, 8H), 7.01 (d, *J* = 7.9 Hz, 2H), 4.79 (d, *J* = 13.7 Hz, 1H), 4.17 (q, *J* = 7.1 Hz, 2H), 2.40 (s, 3H), 1.27 (t, *J* = 7.1 Hz, 3H). ^13^C NMR (100 MHz, CDCl_3_): δ 167.43, 150.26 (d, *J* = 6.7 Hz), 147.20 (d, *J* = 9.3 Hz), 139.10, 134.02 (d, *J* = 2.3 Hz), 130.65, 130.00, 128.57 (d, *J* = 2.5 Hz), 125.67, 120.10 (d, *J* = 5.1 Hz), 100.42 (d, *J* = 9.7 Hz), 60.00, 21.23, 14.43. ^31^P NMR (162 MHz, CDCl_3_) δ-9.54; HRMS (ESI): calculated for C_24_H_25_NO_5_P [M + H]^+^ 438.14649, found 438.14714.
Compound: *ethyl (E)-3-((diphenoxyphosphoryl)(4-methoxyphenyl)amino)acrylate* (**3b**): Following the general procedure, using (petroleum ether:EtOAc = 9:1) as the eluant afforded a colourless liquid (82.5 mg, 91% yield). ^1^H NMR (400 MHz, CDCl_3_): δ 8.20 (dd, *J* = 13.6, 7.5 Hz, 1H), 7.29 (t, *J* = 7.9 Hz, 4H), 7.14 (t, *J* = 8.1 Hz, 6H), 6.95 (d, *J* = 8.8 Hz, 2H), 6.85 (d, *J* = 8.9 Hz, 2H), 4.68 (dd, *J* = 13.7, 1.1 Hz, 1H), 4.09 (q, *J* = 7.2 Hz, 2H), 3.76 (s, 3H), 1.19 (t, *J* = 7.1 Hz, 3H). ^13^C NMR (100 MHz, CDCl_3_): δ 167.45, 159.81, 150.25 (d, *J* = 6.7 Hz), 147.43 (d, *J* = 9.8 Hz), 130.00, 129.95 (d, *J* = 2.6 Hz), 129.04 (d, *J* = 2.3 Hz), 125.67, 120.07 (d, *J* = 5.2 Hz), 115.17, 100.37 (d, *J* = 9.8 Hz), 60.02, 55.56, 14.42. ^31^P NMR (162 MHz, CDCl_3_) δ-9.47; HRMS (ESI): calculated for C_24_H_25_O_6_NP [M + H]^+^ 454.14140, found 454.14206.
Compound: *ethyl (E)-3-((diphenoxyphosphoryl)(4-(trifluoromethoxy)phenyl)amino)acrylate* (**3c**): Following the general procedure, using (petroleum ether:EtOAc = 9:1) as the eluant afforded a colourless liquid (93.3 mg, 92% yield). ^1^H NMR (400 MHz, CDCl_3_): δ 8.26 (dd, *J* = 13.8, 7.7 Hz, 1H), 7.41–7.38 (m, 4H), 7.28–7.22 (m, 8H), 7.16–7.14 (m, 2H), 4.78 (d, *J* = 13.8 Hz, 1H), 4.20 (q, *J* = 7.1 Hz, 2H), 1.29 (t, *J* = 7.1 Hz, 3H). ^13^C NMR (100 MHz, CDCl_3_): δ 167.10, 150.04 (d, *J* = 6.9 Hz), 149.25, 146.47 (d, *J* = 8.5 Hz), 135.25 (d, *J* = 2.9 Hz), 130.50 (d, *J* = 2.8 Hz), 130.11, 125.91, 122.32, 120.4 (q, *J* = 257.0 Hz), 120.05 (d, *J* = 5.1 Hz), 100.97 (d, *J* = 9.3 Hz), 60.22, 14.39. ^31^P NMR (162 MHz, CDCl_3_) δ-9.96; ^19^F NMR (375 MHz, CDCl_3_) δ-57.85; HRMS (ESI): calculated for C_24_H_22_O_6_NF_3_P [M + H]^+^ 508.11313, found 508.11384.
Compound: *ethyl (E)-3-((diphenoxyphosphoryl)(4-phenoxyphenyl)amino)acrylate* (**3d**): Following the general procedure, using (petroleum ether:EtOAc = 9:1) as the eluant afforded a colourless liquid (87.6 mg, 85% yield). ^1^H NMR (400 MHz, CDCl_3_): δ 8.30 (dd, *J* = 13.7, 7.6 Hz, 1H), 7.44–7.37 (m, 6H), 7.26–7.19 (m, 7H), 7.12–7.01 (m, 6H), 4.84 (d, *J* = 13.7 Hz, 1H), 4.21 (q, *J* = 7.1 Hz, 2H), 1.30 (t, *J* = 7.1 Hz, 3H). ^13^C NMR (100 MHz, CDCl_3_): δ 167.36, 158.15, 156.09, 150.21 (d, *J* = 6.7 Hz), 147.15 (d, *J* = 9.4 Hz), 131.07 (d, *J* = 2.4 Hz), 130.24 (d, *J* = 2.5 Hz), 125.76, 124.34, 120.09 (d, *J* = 5.1 Hz), 119.90, 119.20, 100.59 (d, *J* = 9.5 Hz), 60.11, 14.46. ^31^P NMR (162 MHz, CDCl_3_) δ-9.57; HRMS (ESI): calculated for C_29_H_27_NO_6_P [M + H]^+^ 516.15705, found 516.15730.
Compound: *ethyl (E)-3-((4-(tert-butyl)phenyl)(diphenoxyphosphoryl)amino)acrylate* (**3e**): Following the general procedure, using (petroleum ether:EtOAc = 9:1) as the eluant afforded a white solid (90.1 mg, 94% yield), Mp = 67–68 °C. ^1^H NMR (400 MHz, CDCl_3_): δ 8.29 (dd, *J* = 13.6, 7.8 Hz, 1H), 7.44 (d, *J* = 8.6 Hz, 2H), 7.38 (t, *J* = 7.8 Hz, 4H), 7.25–7.22 (m, 6H), 7.06–7.03 (m, 2H), 4.81 (d, *J* = 13.7 Hz, 1H), 4.48–4.02 (m, 12H), 1.37 (s, 9H), 1.29 (t, *J* = 7.1 Hz, 3H). ^13^C NMR (100 MHz, CDCl_3_): δ 167.47, 152.12, 150.25 (d, *J* = 6.8 Hz), 147.19 (d, *J* = 9.1 Hz), 133.91 (d, *J* = 2.3 Hz), 129.99, 128.22 (d, *J* = 2.5 Hz), 126.95, 125.68, 120.14 (d, *J* = 5.1 Hz), 100.39 (d, *J* = 9.8 Hz), 60.03, 34.80, 31.37, 14.45. ^31^P NMR (162 MHz, CDCl_3_) δ-9.47; HRMS (ESI): calculated for C_27_H_31_NO_5_P [M + H]^+^ 480.19344, found 480.19401.
Compound: *ethyl (E)-3-((diphenoxyphosphoryl)(4-fluorophenyl)amino)acrylate* (**3f**): Following the general procedure, using (petroleum ether:EtOAc = 9:1) as the eluant afforded a colourless liquid (66.1 mg, 75% yield). ^1^H NMR (400 MHz, CDCl_3_): δ 8.27 (dd, *J* = 13.7, 7.6 Hz, 1H), 7.41–7.37 (m, 4H), 7.27–7.21 (m, 6H), 7.15–7.07 (m, 4H), 4.75 (d, *J* = 13.7 Hz, 1H), 4.19 (q, *J* = 7.1 Hz, 2H), 1.28 (t, *J* = 7.1 Hz, 3H). ^13^C NMR (100 MHz, CDCl_3_): δ 167.20, 162.53 (d, *J* = 249.6 Hz), 150.11 (d, *J* = 6.8 Hz), 146.84 (d, *J* = 9.1 Hz), 132.64, 130.74 (dd, *J* = 8.8, 2.6 Hz), 130.07, 125.83, 120.04 (d, *J* = 5.1 Hz), 117.05 (d, *J* = 23.0 Hz), 100.76 (d, *J* = 9.5 Hz), 60.14, 14.40. ^31^P NMR (162 MHz, CDCl_3_) δ-9.82; ^19^F NMR (375 MHz, CDCl_3_) δ-111.60; HRMS (ESI): calculated for C_23_H_22_O_5_NFP [M + H]^+^ 442.12141, found 442.12214.
Compound: *ethyl (E)-3-((4-chlorophenyl)(diphenoxyphosphoryl)amino)acrylate* (**3g**): Following the general procedure, using (petroleum ether:EtOAc = 9:1) as the eluant afforded a colourless liquid (74.0 mg, 81% yield). ^1^H NMR (400 MHz, CDCl_3_): δ 8.25 (dd, *J* = 13.7, 7.7 Hz, 1H), 7.43–7.37 (m, 6H), 7.28–7.21 (m, 6H), 7.07–7.04 (m, 2H), 4.77 (d, *J* = 13.7 Hz, 1H), 4.19 (q, *J* = 7.1 Hz, 2H), 1.29 (t, *J* = 7.1 Hz, 3H). ^13^C NMR (100 MHz, CDCl_3_): δ 167.15, 150.07 (d, *J* = 6.8 Hz), 146.54 (d, *J* = 8.9 Hz), 135.21 (d, *J* = 29.0 Hz), 130.32, 130.26 (d, *J* = 2.5 Hz), 130.09, 125.87, 100.90 (d, *J* = 9.4 Hz), 60.17, 14.40. ^31^P NMR (162 MHz, CDCl_3_) δ-10.00; HRMS (ESI): calculated for C_23_H_22_NO_5_ClP [M + H]^+^ 458.09186, found 458.09268.
Compound: *ethyl (E)-3-((4-bromophenyl)(diphenoxyphosphoryl)amino)acrylate* (**3h**): Following the general procedure, using (petroleum ether:EtOAc = 9:1) as the eluant afforded a colourless liquid (79.2 mg, 79% yield). ^1^H NMR (400 MHz, CDCl_3_): δ 8.25 (dd, *J* = 13.7, 7.7 Hz, 1H), 7.58 (d, *J* = 8.6 Hz, 2H), 7.39 (t, *J* = 7.9 Hz, 4H), 7.27–7.22 (m, 6H), 7.00–6.98 (m, 2H), 4.77 (d, *J* = 13.7 Hz, 1H), 4.19 (q, *J* = 7.1 Hz, 2H), 1.29 (t, *J* = 7.1 Hz, 3H). ^13^C NMR (100 MHz, CDCl_3_): δ 167.12, 150.06 (d, *J* = 6.8 Hz), 146.46 (d, *J* = 8.6 Hz), 135.91 (d, *J* = 2.8 Hz), 133.33, 130.58 (d, *J* = 2.6 Hz), 130.10, 125.88, 123.13, 120.05 (d, *J* = 5.2 Hz), 100.93 (d, *J* = 9.5 Hz), 60.18, 14.41. ^31^P NMR (162 MHz, CDCl_3_) δ-10.10; HRMS (ESI): calculated for C_23_H_22_NO_5_BrP [M + H]^+^ 502.04135, found 502.04192.
Compound: *ethyl (E)-3-((diphenoxyphosphoryl)(4-(methylthio)phenyl)amino)acrylate* (**3i**): Following the general procedure, using (petroleum ether:EtOAc = 9:1) as the eluant afforded a colourless liquid (79.7 mg, 85% yield). ^1^H NMR (400 MHz, CDCl_3_): δ 8.28 (dd, *J* = 13.7, 7.6 Hz, 1H), 7.40–7.36 (m, 4H), 7.30–7.22 (m, 8H), 7.04–7.02 (m, 2H), 4.79 (d, *J* = 13.7 Hz, 1H), 4.18 (q, *J* = 7.1 Hz, 2H), 2.53 (s, 3H), 1.28 (t, *J* = 7.1 Hz, 3H). ^13^C NMR (100 MHz, CDCl_3_): δ 167.33, 150.18 (d, *J* = 6.8 Hz), 146.98 (d, *J* = 9.3 Hz), 140.30, 133.35, 130.04, 129.16 (d, *J* = 2.7 Hz), 127.31, 125.75, 120.08 (d, *J* = 5.2 Hz), 100.61 (d, *J* = 9.6 Hz), 60.08, 15.51, 14.42. ^31^P NMR (162 MHz, CDCl_3_) δ-9.72; HRMS (ESI): calculated for C_24_H_25_O_5_NPS [M + H]^+^ 470.11856, found 470.11920.
Compound: *ethyl (E)-3-((diphenoxyphosphoryl)(4-(trifluoromethyl)phenyl)amino)acrylate* (**3j**): Following the general procedure, using (petroleum ether:EtOAc = 9:1) as the eluant afforded a colourless liquid (69.7 mg, 71% yield). ^1^H NMR (400 MHz, CDCl_3_): δ 8.25 (dd, *J* = 15.3, 7.8 Hz, 1H), 7.71 (d, *J* = 8.3 Hz, 1H), 7.57 (d, *J* = 8.5 Hz, 1H), 7.42–7.37 (m, 4H), 7.27–7.21 (m, 7H), 6.99 (d, *J* = 8.5 Hz, 1H), 4.77 (d, *J* = 13.7 Hz, 1H), 4.18 (q, *J* = 7.1 Hz, 2H), 1.29 (t, *J* = 6.1 Hz, 3H). ^13^C NMR (100 MHz, CDCl_3_): δ 167.01, 149.97 (d, *J* = 7.0 Hz), 146.46 (d, *J* = 9.1 Hz), 146.08 (d, *J* = 8.0 Hz), 140.32, 133.33, 130.58, 130.12 (d, *J* = 5.4 Hz), 129.45 (d, *J* = 2.5 Hz), 127.25, 126.00, 125.87, 120.08 (d, *J* = 5.1 Hz), 101.24 (d, *J* = 9.4 Hz), 60.28, 14.38. ^31^P NMR (162 MHz, CDCl_3_) δ-10.18; ^19^F NMR (375 MHz, CDCl_3_) δ-62.71; HRMS (ESI): calculated for C_24_H_22_NO_5_F_3_P [M + H]^+^ 492.11822, found 492.11904.
Compound: *ethyl (E)-3-((4-acetylphenyl)(diphenoxyphosphoryl)amino)acrylate* (**3k**): Following the general procedure, using (petroleum ether:EtOAc = 9:1) as the eluant afforded a colourless liquid (65.1 mg, 70% yield). ^1^H NMR (400 MHz, CDCl_3_): δ 8.24 (dd, *J* = 13.8, 8.0 Hz, 1H), 8.03 (d, *J* = 8.4 Hz, 2H), 7.39 (t, *J* = 7.8 Hz, 4H), 7.28–7.21 (m, 8H), 4.78 (d, *J* = 13.8 Hz, 1H), 4.19 (q, *J* = 7.1 Hz, 2H), 2.65 (s, 3H), 1.28 (t, *J* = 7.1 Hz, 3H). ^13^C NMR (100 MHz, CDCl_3_): δ 196.93, 167.06, 149.98, 146.11 (d, *J* = 8.0 Hz), 141.26, 137.29, 130.11, 130.06, 129.14 (d, *J* = 2.6 Hz), 125.93, 120.08 (d, *J* = 5.1 Hz), 101.19 (d, *J* = 9.3 Hz), 60.21, 26.77, 14.39. ^31^P NMR (162 MHz, CDCl_3_) δ-10.23; HRMS (ESI): calculated for C_26_H_29_NO_5_P [M + H]^+^ 466.17779, found 466.17825.
Compound: *tert-butyl(E)-4-((diphenoxyphosphoryl)(3-ethoxy-3-oxoprop-1-en-1-yl)amino)benzoate* (**3l**): Following the general procedure, using (petroleum ether:EtOAc = 9:1) as the eluant afforded a colourless liquid (73.2 mg, 70% yield). ^1^H NMR (400 MHz, CDCl_3_): δ 8.26 (dd, *J* = 13.8, 7.9 Hz, 1H), 8.07 (d, *J* = 8.4 Hz, 2H), 7.39 (t, *J* = 7.8 Hz, 4H), 7.27–7.18 (m, 8H), 4.77 (d, *J* = 13.7 Hz, 1H), 4.18 (q, *J* = 7.1 Hz, 2H), 1.64 (s, 9H), 1.27 (t, *J* = 7.1 Hz, 3H). ^13^C NMR (100 MHz, CDCl_3_): δ 167.12, 164.70, 150.07 (d, *J* = 6.6 Hz), 146.36 (d, *J* = 8.5 Hz), 140.62, 132.64, 131.22, 130.08, 128.75 (d, *J* = 2.7 Hz), 120.06 (d, *J* = 5.2 Hz), 101.01 (d, *J* = 9.5 Hz), 81.74, 60.13, 28.21, 14.39. ^31^P NMR (162 MHz, CDCl_3_) δ-10.21; HRMS (ESI): calculated for C_28_H_31_NO_7_P [M + H]^+^ 524.18327, found 524.18371.
Compound: *ethyl (E)-3-((diphenoxyphosphoryl)(naphthalen-1-yl)amino)acrylate* (**3m**): Following the general procedure, using (petroleum ether:EtOAc = 9:1) as the eluant afforded a colourless liquid (85.1 mg, 90% yield). ^1^H NMR (400 MHz, CDCl_3_): δ 8.51 (dd, *J* = 13.6, 7.4 Hz, 1H), 7.96–7.91 (m, 2H), 7.73 (d, *J* = 8.5 Hz, 1H), 7.53–7.49 (m, 2H), 7.42–7.37 (m, 4H), 7.34–7.31 (m, 2H), 7.29–7.24 (m, 3H), 7.19–7.15 (m, 1H), 7.07–7.04 (m, 2H), 4.63 (d, *J* = 13.7 Hz, 1H), 4.20–4.12 (m, 2H), 1.24 (t, *J* = 7.1 Hz, 3H). ^13^C NMR (100 MHz, CDCl_3_): δ 167.34, 150.52 (d, *J* = 7.1 Hz), 150.25 (d, *J* = 7.0 Hz), 146.38 (d, *J* = 8.8 Hz), 134.87, 133.08, 130.14, 129.92 (d, *J* = 2.1 Hz), 129.85, 128.57, 127.29, 127.10 (d, *J* = 2.7 Hz), 126.77, 123.09, 120.23 (d, *J* = 2.4 Hz), 120.18 (d, *J* = 2.1 Hz), 101.28 (d, *J* = 9.9 Hz), 60.07, 14.38. ^31^P NMR (162 MHz, CDCl_3_) δ-9.31; HRMS (ESI): calculated for C_27_H_25_NO_5_P [M + H]^+^ 474.14649, found 474.14661.
Compound: *ethyl (E)-3-((diphenoxyphosphoryl)(o-tolyl)amino)acrylate* (**3n**): Following the general procedure, using (petroleum ether:EtOAc = 9:1) as the eluant afforded a colourless liquid (70.8 mg, 81% yield). ^1^H NMR (400 MHz, CDCl_3_): δ 8.28 (dd, *J* = 13.6, 7.5 Hz, 1H), 7.39–7.30 (m, 6H), 7.28–7.21 (m, 5H), 7.16–7.13 (m, 2H), 7.08–7.06(m, 1H), 4.67 (d, *J* = 13.6 Hz, 1H), 4.19 (qd, *J* = 7.1, 1.3 Hz, 2H), 2.14 (s, 3H), 1.28 (t, *J* = 7.1 Hz, 3H). ^13^C NMR (100 MHz, CDCl_3_): δ 167.45, 150.32 (dd, *J* = 21.5, 7.2 Hz), 145.56 (d, *J* = 8.7 Hz), 137.16 (d, *J* = 3.3 Hz), 135.26 (d, *J* = 2.3 Hz), 131.89, 129.99 (d, *J* = 10.8 Hz), 129.17 (d, *J* = 34.9 Hz), 127.58, 125.75 (d, *J* = 5.7 Hz), 120.28 (d, *J* = 4.8 Hz), 120.16 (d, *J* = 5.0 Hz), 100.14 (d, *J* = 10.0 Hz), 60.06, 17.52, 14.42. **^3^**^1^P NMR (162 MHz, CDCl_3_) δ-9.53; HRMS (ESI): calculated for C_24_H_25_NO_5_P [M + H]^+^ 438.14649, found 438.14709.
Compound: *ethyl (E)-3-((diphenoxyphosphoryl)(mesityl)amino)acrylate* (**3o**): Following the general procedure, using (petroleum ether:EtOAc = 9:1) as the eluant afforded a colourless liquid (66.9 mg, 72% yield). ^1^H NMR (400 MHz, CDCl_3_): δ 8.25 (dd, *J* = 13.5, 7.3 Hz, 1H), 7.36–7.32 (m, 4H), 7.24–7.19 (m, 6H), 6.95 (s, 2H), 4.75 (d, *J* = 13.4 Hz, 1H), 4.22 (q, *J* = 7.1 Hz, 2H), 2.32 (s, 3H), 2.13 (s, 6H), 1.31 (t, *J* = 7.1 Hz, 3H). ^13^C NMR (100 MHz, CDCl_3_): δ 167.74, 150.52 (d, *J* = 7.7 Hz), 144.67 (d, *J* = 8.5 Hz), 138.72, 136.77 (d, *J* = 2.5 Hz), 131.66 (d, *J* = 2.9 Hz), 130.12, 129.89, 125.68, 120.45 (d, *J* = 4.8 Hz), 99.32 (d, *J* = 9.8 Hz), 60.06, 21.02, 18.04, 14.42. ^31^P NMR (162 MHz, CDCl_3_) δ-9.34; HRMS (ESI): calculated for C_26_H_29_NO_5_P [M + H]^+^ 466.17779, found 466.17815.
Compound: *ethyl (E)-3-((3,5-dimethylphenyl)(diphenoxyphosphoryl)amino)acrylate* (**3p**): Following the general procedure, using (petroleum ether:EtOAc = 9:1) as the eluant afforded a white solid (81.2 mg, 90% yield), Mp = 69–70 °C. ^1^H NMR (400 MHz, CDCl_3_): δ 8.39 (dd, *J* = 13.7, 7.7 Hz, 1H), 7.52–7.48 (m, 4H), 7.37–7.34 (m, 6H), 7.15 (s, 1H), 6.79 (s, 2H), 4.90 (d, *J* = 13.6 Hz, 1H), 4.30 (q, *J* = 7.0 Hz, 2H), 2.41 (s, 4H), 1.40 (t, *J* = 7.0 Hz, 3H). ^13^C NMR (100 MHz, CDCl_3_): δ 167.49, 150.31 (d, *J* = 6.6 Hz), 147.15 (d, *J* = 9.0 Hz), 139.71, 136.40 (d, *J* = 2.3 Hz), 130.68, 129.96, 126.33 (d, *J* = 2.7 Hz), 125.66, 120.14 (d, *J* = 5.3 Hz), 100.41 (d, *J* = 9.8 Hz), 60.00, 21.22, 14.44. ^31^P NMR (162 MHz, CDCl_3_) δ-9.60; HRMS (ESI): calculated for C_25_H_27_NO_5_P [M + H]^+^ 452.16214, found 452.16280.
Compound: *ethyl (E)-3-((diphenoxyphosphoryl)(3,4,5-trimethoxyphenyl)amino)acrylate* (**3q**): Following the general procedure, using (petroleum ether:EtOAc = 9:1) as the eluant afforded a colourless liquid (95.4 mg, 93% yield). ^1^H NMR (400 MHz, CDCl_3_): δ 8.25 (dd, *J* = 13.6, 7.6 Hz, 1H), 7.40–7.36 (m, 4H), 7.27–7.21 (m, 6H), 6.20 (s, 2H), 4.84 (d, *J* = 13.6 Hz, 1H), 4.19 (q, *J* = 7.1 Hz, 2H), 3.87 (s, 3H), 3.67 (s, 6H), 1.29 (t, *J* = 7.1 Hz, 3H). ^13^C NMR (100 MHz, CDCl_3_): δ 167.37, 153.90, 150.24 (d, *J* = 6.6 Hz), 146.91 (d, *J* = 9.0 Hz), 138.29, 132.00 (d, *J* = 2.6 Hz), 130.04, 125.72, 120.04 (d, *J* = 5.3 Hz), 105.79 (d, *J* = 2.5 Hz), 100.67 (d, *J* = 9.5 Hz), 60.96, 60.12, 56.05, 14.43. **^3^**^1^P NMR (162 MHz, CDCl_3_) δ-9.99; HRMS (ESI): calculated for C_26_H_29_NO_8_P [M + H]^+^ 514.16253, found 514.16286.
Compound: *ethyl (E)-3-(((naphthalen-2-yloxy)(phenoxy)phosphoryl)(phenyl)amino)acrylate* (**3r**): Following the general procedure, using (petroleum ether:EtOAc = 9:1) as the eluant afforded a colourless liquid (83.3 mg, 88% yield). ^1^H NMR (400 MHz, CDCl_3_): δ 8.37 (dd, *J* = 13.7, 7.8 Hz, 1H), 7.87–7.82 (m, 3H), 7.74–7.73 (m, 1H), 7.58–7.43 (m, 5H), 7.41–7.37 (m, 2H), 7.34–7.30 (m, 1H), 7.27–7.23 (m, 3H), 7.19–7.16 (m, 2H), 4.81 (d, *J* = 14.5 Hz, 1H), 4.20 (q, *J* = 7.1 Hz, 2H), 1.29 (t, *J* = 7.1 Hz, 3H). ^13^C NMR (100 MHz, CDCl_3_): δ 167.37, 150.27 (d, *J* = 7.0 Hz), 147.82 (d, *J* = 7.0 Hz), 147.01 (d, *J* = 9.0 Hz), 136.82, 133.86, 131.21, 130.23, 130.06, 129.11, 128.93 (d, *J* = 2.7 Hz), 127.79 (d, *J* = 9.9 Hz), 127.04, 125.93, 125.77, 120.13 (d, *J* = 5.2 Hz), 119.77 (d, *J* = 5.6 Hz), 116.77 (d, *J* = 5.0 Hz), 100.70 (d, *J* = 9.8 Hz), 60.08, 14.45. ^31^P NMR (162 MHz, CDCl_3_) δ-9.59; HRMS (ESI): calculated for C_27_H_25_NO_5_P [M + H]^+^ 474.14649, found 474.14693.
Compound: *methyl (E)-3-((diphenoxyphosphoryl)(phenyl)amino)acrylate* (**4a**): Following the general procedure, using (petroleum ether:EtOAc = 9:1) as the eluant afforded a colourless liquid (71.2 mg, 87% yield). ^1^H NMR (400 MHz, CDCl_3_): δ 8.30 (dd, *J* = 13.7, 7.9 Hz, 1H), 7.46–7.43 (m, 3H), 7.41–7.37 (m, 4H), 7.27–7.21 (m, 6H), 7.15–7.12 (m, 2H), 4.78 (d, *J* = 13.7 Hz, 1H), 3.72 (s, 3H). ^13^C NMR (100 MHz, CDCl_3_): δ 167.77, 150.20 (d, *J* = 6.7 Hz), 147.21 (d, *J* = 9.0 Hz), 136.75, 130.02, 129.07, 128.84 (d, *J* = 2.5 Hz), 125.72, 120.08 (d, *J* = 5.2 Hz), 100.15 (d, *J* = 9.7 Hz), 51.31. ^31^P NMR (162 MHz, CDCl_3_) δ-9.77; HRMS (ESI): calculated for C_22_H_21_NO_5_P [M + H]^+^ 410.11519, found 410.11543.
Compound: *2,2,2-trifluoroethyl (E)-3-((diphenoxyphosphoryl)(phenyl)amino)acrylate* (**4b**): Following the general procedure, using (petroleum ether:EtOAc = 9:1) as the eluant afforded a white solid (86.8 mg, 91% yield), Mp = 84–85 °C. ^1^H NMR (400 MHz, CDCl_3_): δ 8.39 (dd, *J* = 13.7, 8.1 Hz, 1H), 7.49–7.47 (m, 3H), 7.39 (t, *J* = 7.8 Hz, 4H), 7.27–7.20 (m, 6H), 7.17–7.15(m, 2H), 4.80 (d, *J* = 13.7 Hz, 1H), 4.51 (q, *J* = 8.5 Hz, 2H). ^13^C NMR (100 MHz, CDCl_3_): δ 165.63, 150.13 (d, *J* = 6.8 Hz), 149.15 (d, *J* = 9.4 Hz), 136.46, 130.16, 130.06, 129.82, 129.33, 128.72, 125.83, 120.02 (d, *J* = 5.1 Hz), 98.01 (d, *J* = 9.8 Hz), 59.76 (t, *J* = 36.3 Hz). ^31^P NMR (162 MHz, CDCl_3_) δ-10.16; ^19^F NMR (375 MHz, CDCl_3_) δ-73.73 (3F); HRMS (ESI): calculated for C_23_H_20_NO_5_F_3_P [M + H]^+^ 478.10257, found 478.10314.
Compound: *2-methoxyethyl (E)-3-((diphenoxyphosphoryl)(phenyl)amino)acrylate* (**4c**): Following the general procedure, using (petroleum ether:EtOAc = 9:1) as the eluant afforded a white solid (78.8 mg, 87% yield), Mp = 46–47 °C. ^1^H NMR (400 MHz, CDCl_3_): δ 8.32 (dd, *J* = 13.7, 7.8 Hz, 1H), 7.44–7.42 (m, 3H), 7.40–7.36 (m, 4H), 7.26–7.20 (m, 6H), 7.13–7.11 (m, 2H), 4.84 (dd, *J* = 13.7, 1.1 Hz, 1H), 4.29–4.26 (m, 2H), 3.62–3.60 (m, 2H), 3.39 (s, 3H). ^13^C NMR (100 MHz, CDCl_3_): δ 167.41, 150.19 (d, *J* = 6.7 Hz), 147.45 (d, *J* = 9.0 Hz), 136.77, 130.01, 129.84, 129.07, 128.87 (d, *J* = 2.7 Hz), 125.72, 120.09 (d, *J* = 5.1 Hz), 100.12 (d, *J* = 9.8 Hz), 70.67, 63.12, 59.04.
^31^P NMR (162 MHz, CDCl_3_) δ-9.79; HRMS (ESI): calculated for C_24_H_25_NO_6_P [M + H]^+^ 454.14140, found 454.14180.
Compound: *4-chlorophenyl (E)-3-((diphenoxyphosphoryl)(phenyl)amino)acrylate* (**4d**): Following the general procedure, using (petroleum ether:EtOAc = 9:1) as the eluant afforded a white solid (77.8 mg, 77% yield), Mp = 123–124 °C. ^1^H NMR (400 MHz, CDCl_3_): δ 8.51 (dd, *J* = 13.7, 8.0 Hz, 1H), 7.52–7.49 (m, 3H), 7.42–7.35 (m, 6H), 7.28–7.23 (m, 8H), 7.08–7.06 (m, 2H), 4.94 (d, *J* = 13.7 Hz, 1H). ^13^C NMR (100 MHz, CDCl_3_): δ 165.58, 150.17 (d, *J* = 6.8 Hz), 149.34, 149.13 (d, *J* = 9.3 Hz), 136.54, 130.91, 130.20, 130.10, 129.44, 129.38, 128.80 (d, *J* = 2.6 Hz), 125.87, 123.20, 120.04 (d, *J* = 5.1 Hz), 99.06 (d, *J* = 9.9 Hz). ^31^P NMR (162 MHz, CDCl_3_) δ-10.10; HRMS (ESI): calculated for C_27_H_22_NO_5_ClP [M + H]^+^ 506.09186, found 506.09252.
Compound: *(tetrahydrofuran-2-yl)methyl (E)-3-((diphenoxyphosphoryl)(phenyl)amino)acrylate* (**4e**): Following the general procedure, using (petroleum ether:EtOAc = 9:1) as the eluant afforded a white solid (77.6 mg, 81% yield), Mp = 64–65 °C. ^1^H NMR (400 MHz, CDCl_3_): δ 8.32 (dd, *J* = 13.7, 7.8 Hz, 1H), 7.45–7.41 (m, 3H), 7.39–7.30 (m, 5H), 7.24–7.21 (m, 5H), 7.13–7.10 (m, 2H), 4.84 (d, *J* = 13.7 Hz, 1H), 4.23 (dd, *J* = 11.3, 3.3 Hz, 1H), 4.13 (qd, *J* = 7.1, 3.3 Hz, 1H), 4.02 (dd, *J* = 11.3, 7.3 Hz, 1H), 3.89 (dt, *J* = 8.5, 6.7 Hz, 1H), 3.82–3.77 (m, 1H), 2.07–1.98 (m, 1H), 1.97–1.86 (m, 2H), 1.64–1.56 (m, 1H). ^13^C NMR (100 MHz, CDCl_3_): δ 167.37, 150.19 (d, *J* = 6.7 Hz), 147.40 (d, *J* = 8.9 Hz), 136.75, 130.01, 129.81, 129.48, 129.06, 128.86 (d, *J* = 2.6 Hz), 125.72, 125.42, 122.57, 120.47 (d, *J* = 4.8 Hz), 120.09 (d, *J* = 5.1 Hz), 100.16 (d, *J* = 9.8 Hz), 76.70, 68.44, 66.23, 28.02, 25.66. ^31^P NMR (162 MHz, CDCl_3_) δ-9.77; HRMS (ESI): calculated for C_26_H_27_NO_6_P [M + H]^+^ 480.15705, found 480.15766.
Compound: *2,6-dimethylphenyl (E)-3-((diphenoxyphosphoryl)(phenyl)amino)acrylate* (**4f**): Following the general procedure, using (petroleum ether:EtOAc = 9:1) as the eluant afforded a white solid (83.8 mg, 84% yield), Mp = 121–122 °C. ^1^H NMR (400 MHz, CDCl_3_): δ 8.52 (dd, *J* = 13.7, 8.0 Hz, 1H), 7.53–7.48 (m, 3H), 7.42–7.38 (m, 4H), 7.28–7.23 (m, 8H), 7.09–7.07 (m, 3H), 4.99 (d, *J* = 13.0 Hz, 1H), 2.18 (s, 6H). ^13^C NMR (100 MHz, CDCl_3_): δ 165.03, 150.21 (d, *J* = 7.0 Hz), 148.50 (d, *J* = 9.3 Hz), 148.20, 136.65, 130.52, 130.16, 130.05, 129.26, 128.88 (d, *J* = 2.4 Hz), 128.52, 125.81, 125.71, 120.09 (d, *J* = 5.1 Hz), 99.04 (d, *J* = 9.9 Hz), 16.45. ^31^P NMR (162 MHz, CDCl_3_) δ-9.97; HRMS (ESI): calculated for C_29_H_27_NO_5_P [M + H]^+^ 500.16214, found 500.16265.
Compound: *4-chloro-3,5-dimethylphenyl (E)-3-((diphenoxyphosphoryl)(phenyl)amino)acrylate* (**4g**): Following the general procedure, using (petroleum ether:EtOAc = 9:1) as the eluant afforded a colourless liquid (79.9 mg, 75% yield). ^1^H NMR (400 MHz, CDCl_3_): δ 8.50 (dd, *J* = 13.6, 8.0 Hz, 1H), 7.51–7.49 (m, 3H), 7.40 (t, *J* = 7.8 Hz, 4H), 7.29–7.21 (m, 8H), 6.87 (s, 2H), 4.94 (d, *J* = 13.6 Hz, 1H), 2.40 (s, 6H). ^13^C NMR (100 MHz, CDCl_3_): δ 165.90, 150.19 (d, *J* = 6.9 Hz), 148.89 (d, *J* = 9.2 Hz), 148.45, 137.39, 136.58, 131.51, 130.19, 130.09, 129.34, 128.82 (d, *J* = 2.6 Hz), 125.85, 121.64, 120.06 (d, *J* = 5.2 Hz), 115.43, 99.31 (d, *J* = 9.8 Hz), 20.91. ^31^P NMR (162 MHz, CDCl_3_) δ-10.06; HRMS (ESI): calculated for C_29_H_26_NO_5_ClP [M + H]^+^ 534.12316, found 534.12377.
Compound: *1,7,7-trimethylbicyclo[2.2.1]heptan-2-yl (E)-3-((diphenoxyphosphoryl)(phenyl)amino)acrylate* (**4h**): Following the general procedure, using (petroleum ether:EtOAc = 9:1) as the eluant afforded a colourless liquid (91.4 mg, 86% yield). ^1^H NMR (400 MHz, CDCl_3_): δ 8.22 (dd, *J* = 13.6, 8.0 Hz, 1H), 7.46–7.44 (m, 3H), 7.40–7.36 (m, 4H), 7.26–7.21 (m, 6H), 7.15–7.12 (m, 2H), 4.75–4.71 (m, 2H), 1.84–1.70 (m, 6H), 1.67 (s, 1H), 1.04 (s, 3H), 0.88–0.87 (m, 6H). ^13^C NMR (100 MHz, CDCl_3_): δ 166.86, 150.21(d, *J* = 7.0 Hz), 146.49 (d, *J* = 8.9 Hz), 136.79, 129.42 (d, *J* = 116.1 Hz), 128.99, 128.86, 125.69, 120.09 (d, *J* = 5.2 Hz), 100.98 (d, *J* = 9.6 Hz), 80.62, 48.87, 46.99, 45.11, 38.91, 33.80, 27.11, 20.18, 20.09, 11.61. ^31^P NMR (162 MHz, CDCl_3_) δ-9.70; HRMS (ESI): calculated for C_31_H_35_NO_5_P [M + H]^+^ 532.22474, found 532.22549.
Compound: *5-isopropyl-2-methylphenyl (E)-3-((diphenoxyphosphoryl)(phenyl)amino)acrylate* (**4i**): Following the general procedure, using (petroleum ether:EtOAc = 9:1) as the eluant afforded a colourless liquid (81.2 mg, 77% yield). ^1^H NMR (400 MHz, CDCl_3_): δ 8.52 (dd, *J* = 13.7, 8.0 Hz, 1H), 7.54–7.49 (m, 3H), 7.43–7.39 (m, 4H), 7.30–7.24 (m, 8H), 7.18 (d, *J* = 7.8 Hz, 1H), 7.05 (dd, *J* = 7.8, 1.9 Hz, 1H), 6.92 (d, *J* = 1.9 Hz, 1H), 4.99 (d, *J* = 13.7 Hz, 1H), 2.92 (p, *J* = 6.9 Hz, 1H), 2.18 (s, 3H), 1.28 (d, *J* = 6.9 Hz, 6H). ^13^C NMR (100 MHz, CDCl_3_): δ 165.63, 150.23 (d, *J* = 6.9 Hz), 149.36, 148.53 (d, *J* = 9.1 Hz), 147.99, 136.69, 130.85, 130.16, 130.07, 129.27, 128.89 (d, *J* = 2.5 Hz), 127.59, 125.81, 124.04, 120.10 (d, *J* = 5.1 Hz), 120.03, 99.44 (d, *J* = 9.7 Hz), 33.63, 24.00, 15.95. ^31^P NMR (162 MHz, CDCl_3_) δ-9.95; HRMS (ESI): calculated for C_31_H_31_NO_5_P [M + H]^+^ 528.19344, found 528.19397.
Compound: *(1R,2S,5R)-2-isopropyl-5-methylcyclohexyl (E)-3-((diphenoxyphosphoryl)(phenyl)amino)acrylate* (**4j**): Following the general procedure, using (petroleum ether:EtOAc = 9:1) as the eluant afforded a colourless liquid (73.6 mg, 69% yield). ^1^H NMR (400 MHz, CDCl_3_): δ 8.26 (dd, *J* = 13.7, 8.0 Hz, 1H), 7.46–7.44 (m, 3H), 7.40–7.36 (m, 4H), 7.26–7.21 (m, 6H), 7.17–7.15 (m, 2H), 4.78–4.74 (m, 2H), 2.05–2.00 (m, 1H), 1.92–1.85 (m, 1H), 1.73–1.67 (m, 3H), 1.43–1.36 (m, 1H), 0.98–0.91 (m, 9H), 0.80 (d, J = 6.9 Hz, 3H). ^13^C NMR (100 MHz, CDCl_3_): δ 166.96, 150.24 (d, *J* = 6.8 Hz), 146.70 (d, *J* = 8.7 Hz), 136.83, 129.99, 129.71, 129.00, 128.94 (d, *J* = 2.5 Hz), 125.68, 120.10 (d, *J* = 5.1 Hz), 100.93 (d, *J* = 9.9 Hz), 73.67, 47.15, 41.17, 34.35, 31.45, 26.27, 23.53, 22.10, 20.85, 16.45. ^31^P NMR (162 MHz, CDCl_3_) δ-9.72; HRMS (ESI): calculated for C_31_H_37_NO_5_P [M + H]^+^ 534.24039, found 534.24108.
Compound: *diphenyl (E)-(3-oxopent-1-en-1-yl)(phenyl)phosphoramidate* (**4k**): Following the general procedure, using (petroleum ether:EtOAc = 9:1) as the eluant afforded a colourless liquid (73.3 mg, 90% yield). ^1^H NMR (400 MHz, CDCl_3_): δ 8.24 (dd, *J* = 13.9, 7.9 Hz, 1H), 7.48–7.44 (m, 3H), 7.39–7.36 (m, 4H), 7.26–7.20 (m, 6H), 7.16–7.13 (m, 2H), 5.15 (d, *J* = 13.9 Hz, 1H), 2.49 (q, *J* = 7.3 Hz, 2H), 1.09 (t, *J* = 7.4 Hz, 3H). ^13^C NMR (100 MHz, CDCl_3_): δ 199.93, 150.19 (d, *J* = 6.9 Hz), 145.99 (d, *J* = 8.8 Hz), 136.75, 130.02, 129.10, 128.84 (d, *J* = 2.5 Hz), 125.75, 120.06 (d, *J* = 5.2 Hz), 109.71 (d, *J* = 8.5 Hz), 33.76, 8.51. ^31^P NMR (162 MHz, CDCl_3_) δ-9.60; HRMS (ESI): calculated for C_23_H_23_NO_4_P [M + H]^+^ 408.13592, found 408.13654.
Compound: *diphenyl (E)-phenyl(styryl)phosphoramidate* (**4l**): Following the general procedure, using (petroleum ether:EtOAc = 9:1) as the eluant afforded a white solid (54.7 mg, 64% yield), Mp = 87–88 °C. ^1^H NMR (400 MHz, CDCl_3_): δ 7.51–7.49 (m, 2H), 7.44–7.42 (m, 2H), 7.36–7.25 (m, 9H), 7.23–7.19 (m, 5H), 7.14–7.11 (m, 1H), 5.75 (s, 1H), 5.62 (s, 1H). ^13^C NMR (100 MHz, CDCl_3_): δ 150.91 (d, *J* = 7.1 Hz), 146.37, 141.52, 137.13, 129.84, 129.02, 128.54, 128.34, 128.15, 127.09, 125.35, 124.98 (d, *J* = 3.6 Hz), 120.63 (d, *J* = 5.0 Hz), 113.51. ^31^P NMR (162 MHz, CDCl_3_) δ-7.65; HRMS (ESI): calculated for C_26_H_23_NO_3_P [M + H]^+^ 428.14101, found 428.14153.
Compound: *ethyl (E)-3-((diphenoxyphosphoryl)(4-isopropylphenyl)amino)acrylate* (**5a**): Following the general procedure, using (petroleum ether:EtOAc = 9:1) as the eluant afforded a colourless liquid (84.6 mg, 91% yield). ^1^H NMR (400 MHz, CDCl_3_): δ 8.29 (dd, *J* = 13.6, 7.8 Hz, 1H), 7.39–7.35 (m, 4H), 7.30–7.27 (m, 2H), 7.25–7.21 (m, 6H), 7.05–7.03 (m, 2H), 4.80 (d, *J* = 14.2 Hz, 1H), 4.18 (q, *J* = 7.1 Hz, 2H), 2.96 (p, *J* = 6.9 Hz, 1H), 1.30–1.29 (m, 6H). ^13^C NMR (100 MHz, CDCl_3_): δ 167.46, 150.26 (d, *J* = 6.7 Hz), 149.84, 147.22 (d, *J* = 9.2 Hz), 134.19 (d, *J* = 2.3 Hz), 129.99, 128.58 (d, *J* = 2.6 Hz), 128.01, 125.67, 120.12 (d, *J* = 5.2 Hz), 100.40 (d, *J* = 9.5 Hz), 60.02, 33.90, 23.97, 14.45. ^31^P NMR (162 MHz, CDCl_3_) δ-9.50; HRMS (ESI): calculated for C_26_H_29_NO_5_P [M + H]^+^ 466.17779, found 466.17812.
Compound: *ethyl (E)-4-((diphenoxyphosphoryl)(3-ethoxy-3-oxoprop-1-en-1-yl)amino)benzoate* (**5b**): Following the general procedure, using (petroleum ether:EtOAc = 9:1) as the eluant afforded a colourless liquid (67.3 mg, 68% yield). ^1^H NMR (400 MHz, CDCl_3_): δ 8.26 (dd, *J* = 13.8, 7.9 Hz, 1H), 8.13 (d, *J* = 8.2 Hz, 2H), 7.39 (t, *J* = 7.8 Hz, 4H), 7.30–7.19 (m, 8H), 4.77 (d, *J* = 13.8 Hz, 1H), 4.43 (q, *J* = 7.2 Hz, 2H), 4.18 (q, *J* = 7.1 Hz, 2H), 1.44 (t, *J* = 7.2 Hz, 3H), 1.28 (t, *J* = 7.1 Hz, 3H). ^13^C NMR (100 MHz, CDCl_3_): δ 167.09, 165.59, 150.05 (d, *J* = 6.7 Hz), 146.25 (d, *J* = 8.4 Hz), 141.02, 131.34, 131.09, 130.09, 128.89, 125.88, 120.06 (d, *J* = 5.1 Hz), 101.09 (d, *J* = 9.4 Hz), 61.45, 60.17, 14.38, 14.36. ^31^P NMR (162 MHz, CDCl_3_) δ-10.25; HRMS (ESI): calculated for C_26_H_27_NO_7_P [M + H]^+^ 496.15197, found 496.15246.
Compound: *(S)-2,5,7,8-tetramethyl-2-((4S,8S)-4,8,12-trimethyltridecyl)chroman-6-yl (E)-3-((diphenoxyphosphoryl)(phenyl)amino)acrylate* (**5c**): Following the general procedure, using (petroleum ether:EtOAc = 9:1) as the eluant afforded a colourless liquid (113.0 mg, 70% yield). ^1^H NMR (400 MHz, CDCl_3_): δ 8.50 (dd, *J* = 13.8, 7.9 Hz, 1H), 7.51–7.49 (m, 3H), 7.42–7.38 (m, 4H), 7.28–7.23 (m, 8H), 5.01 (d, *J* = 13.8 Hz, 1H), 2.62 (t, *J* = 6.8 Hz, 1H), 2.12 (s, 3H), 2.05 (s, 3H), 2.00 (s, 3H), 1.81 (dq, *J* = 20.7, 6.8 Hz, 2H), 1.61–1.53 (m, 4H), 1.46–1.25 (m, 14H), 1.20–1.14 (m, 3H), 1.15–1.09 (m, 3H), 0.92–0.88 (m, 12H). ^13^C NMR (100 MHz, CDCl_3_): δ 165.92, 150.25, 149.36, 148.13, 140.40, 136.74, 130.12, 130.03, 129.18, 128.91, 127.07, 125.78, 125.29, 122.96, 120.12 (d, *J* = 5.2 Hz), 117.33, 99.50 (d, *J* = 9.1 Hz), 75.05, 39.44, 37.52, 37.36, 32.86, 32.79, 28.05, 24.87, 24.52, 22.80, 22.70, 21.11, 20.66, 19.83, 19.73, 13.10, 12.26, 11.90. ^31^P NMR (162 MHz, CDCl_3_) δ-9.89; HRMS (ESI): calculated for C_50_H_67_NO_6_P [M + H]^+^ 808.47005, found 808.47048.

## Figures and Tables

**Table 1:** Reaction Optimization ^a^.

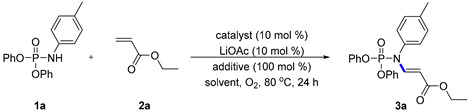				
Entry	Catalyst	Additive	Solvent	Yield (%) ^b^
1	Pd(OAc)_2_	Cu(OAc)_2_	DME	0
2	Pd(OAc)_2_	CuCl_2_	DME	0
3	Pd(OAc)_2_	BQ	DME	0
4	Pd(OAc)_2_	pyridine	DME	0
5	Pd(OAc)_2_	LiBr	DME	0
6	Pd(OAc)_2_	TBAB	DME	65
7	Pd(OAc)_2_	TBAF	DME	0
8	Pd(OAc)_2_	TBAC	DME	0
9	Pd(OAc)_2_	TBAI	DME	0
10	Pd(OAc)_2_	TBAB	TBME	80
11 ^c^	Pd(OAc)_2_	TBAB	TBME	11
12 ^d^	Pd(OAc)_2_	TBAB	TBME	14
13	Pd(TFA)_2_	TBAB	TBME	89
14 ^e^	Pd(TFA)_2_	TBAB	TBME	7

^a^ Reaction conditions: **1a** (0.2 mmol), **2a** (0.6 mmol), catalyst (0.02 mmol), LiOAc (0.02 mmol), additive (0.2 mmol), solvent (2 mL), under O_2_, heated at 80 °C for 24 h. ^b^ Isolated yield. ^c^ Pyridine as the ligand (0.04 mmol). ^d^ Phthalimide as the ligand (0.02 mmol). ^e^ Under N_2_ atmosphere. TBME = *tert*-Butyl methyl ether.

## Data Availability

The data for the synthesized compounds presented in this study are available in Supplementary Material.

## Supplementary Materials

The following supporting information can be downloaded at: https://www.mdpi.com/article/10.3390/molecules28114466/s1, ^1^H, ^13^C and ^19^F NMR spectral data of all compounds are reported in the Supporting Information file.

## References

[1] Zhu Y.-Y., Niu Y., Niu Y.-N., Yang S.-D. (2021). Recent Advances in the Synthesis and Applications of Phosphoramides. Org. Biomol. Chem..

[2] Oliveira F.M., Barbosa L.C.A., Ismailc F.M.D. (2014). The Diverse Pharmacology and Medicinal Chemistry of Phosphoramidates A Review. RSC Adv..

[3] Liu Z., Klapars A., Simmons B., Bellomo A., Kalinin A., Weisel M., Hill J., Silverman S.M. (2021). Development and Implementation of an Aluminum-Promoted Phosphorylation in the Uprifosbuvir Manufacturing Route. Org. Process Res. Dev..

[4] Mehellou Y., Rattan H.S., Balzarini J. (2018). The ProTide Prodrug Technology: From the Concept to the Clinic. J. Med. Chem..

[5] Holshue M.L., DeBolt C., Lindquist S., Lofy K.H., Wiesman J., Bruce H., Spitters C., Ericson K., Wilkerson S., Tural A. (2020). First Case of 2019 Novel Coronavirus in the United States. N. Engl. J. Med..

[6] Raia A., Yadav L.D.S. (2011). Strategic Applications of Baylis-Hillman Adducts to General Syntheses of 3-Nitroazetidines. Org. Biomol. Chem..

[7] Yin G., Zhu Y., Zhang L., Lu P., Wang Y. (2011). Preparation of Allenephosphoramide and Its Utility in the Preparation of 4,9-Dihydro-2H-benzo[f]isoindoles. Org. Lett..

[8] Shinde A.H., Thomas A.A., Mague J.T., Sathyamoorthi S. (2021). Highly Regio- and Diastereoselective Tethered Aza-Wacker Cyclizations of Alkenyl Phosphoramidates. J. Org. Chem..

[9] DeKorver K.A., Walton M.C., North T.D., Hsung R.P. (2011). Introducing a New Class of N-Phosphoryl Ynamides via Cu(I)-Catalyzed Amidations of Alkynyl Bromides. Org. Lett..

[10] Jillella R., Raju S., Hsiao H.-C., Hsu D.-S., Chuang S.-C. (2020). Pd-Catalyzed Redox-Neutral C-N Coupling Reaction of Iminoquinones with Electron-Deficient Alkenes without External Oxidants Access of Tertiary (E)-Enamines and Application to the Synthesis of Indoles and Quinolin-4-ones. Org. Lett..

[11] Pratap K., Kumar A. (2018). Palladium-Catalyzed Intermolecular Dehydrogenative Carboamination of Alkenes with Amines and N-Substituted Isatin. Org. Lett..

[12] Ji X., Huang H., Wu W., Li X., Jiang H. (2013). Palladium-Catalyzed Oxidative Coupling of Aromatic Primary Amines and Alkenes under Molecular Oxygen Stereoselective Assembly of (Z)-Enamines. J. Org. Chem..

[13] Mizuta Y., Yasuda K., Obora Y. (2013). Palladium-Catalyzed Z-Selective Oxidative Amination of ortho-Substituted Primary Anilines with Olefins under an Open Air Atmosphere. J. Org. Chem..

[14] Chary B.C., Kim S., Park Y., Kim J., Lee P.H. (2013). Palladium-Catalyzed C-H Arylation Using Phosphoramidate as a Directing Group at Room Temperature. Org. Lett..

[15] Wu G.-J., Han F.-S., Zhao Y.-L. (2015). Palladacycles Derived from Arylphosphinamides for Mild Suzuki-Miyaura Cross-Couplings. RSC Adv..

[16] Garcia P., Lau Y.Y., Perry M.R., Schafer L.L. (2013). Phosphoramidate Tantalum Complexes for Room-Temperature C-H Functionalization: Hydroaminoalkylation Catalysis. Angew. Chem. Int. Ed..

[17] Jiao L.-Y., Ferreira A.V., Oestreich M. (2016). Phosphinic Amide as Directing Group Enabling Palladium(II)-Catalyzed ortho C-H Alkenylation of Anilines without and with Alkylation at the Nitrogen Atom. Chem. Asian J..

[18] Park S., Seo B., Shin S., Son J.-Y., Lee P.H. (2013). Rhodium-Catalyzed Oxidative Coupling through C-H Activation and Annulation Directed by Phosphonamide and Phosphinamide Groups. Chem. Commun..

[19] Shi S., Ma Y., Zhou J., Li J., Chen L., Wu G. (2020). Copper-Catalyzed Oxidative Thioamination of Maleimides with Amines and Bunte Salts. Org. Lett..

[20] Gao X., Tang L., Huang L., Huang Z.-S., Ma Y., Wu G. (2019). Oxidative Aminoarylselenation of Maleimides via Copper-Catalyzed Four-Component Cross-Coupling. Org. Lett..

[21] Wu G., Su W. (2013). Regio- and Stereoselective Direct *N*-Alkenylation of Indoles via Pd-Catalyzed Aerobic Oxidation. Org. Lett..

[22] Bhatt S., Wang Y.-N., Pham H.T.P., Hull K.L. (2022). Palladium-Catalyzed Oxidative Amination of α-Olefins with Indoles. Org. Lett..

[23] Timokhin V.I., Stahl S.S. (2005). Brønsted Base-Modulated Regioselectivity in the Aerobic Oxidative Amination of Styrene Catalyzed by Palladium. J. Am. Chem. Soc..

[24] Geng M., Kuang J., Miao M., Ma Y. (2023). Cu(ii)-Catalyzed Domino Construction of Spironaphthalenones by Dearomatization of β-Naphthols and using *N*,*N*-Dimethylaminoethanol as a C1 Synthon. Org. Biomol. Chem..

[25] Fang J., Pan Z., Liu T., Rao Y., Jiang H., Ma Y. (2023). I_2_-Mediated Coupling of Quinazolinone Enamines with 2-Aminopyridines: A New Strategy to Access Spiroquinazolinones. Org. Biomol. Chem..

[26] Reetz M.T., de Vries J.G. (2004). Ligand-Free Heck Reactions Using Low Pd-Loading. Chem. Commun..

[27] de Vries A.H.M., Parlevliet F.J., Schmieder-van de Vondervoort L., Mommers J.H.M., Henderickx H.J.W., Walet M.A.M., de Vries J.G. (2002). A Practical Recycle of a Ligand-Free Palladium Catalyst for Heck Reactions. Adv. Synth. Catal..

