# Supplementary material for: Palladium-Catalyzed N-Alkenylation of N-Aryl Phosphoramidates with Alkenes
Source: Molecules. 2023 May 31;28(11):4466. doi: 10.3390/molecules28114466 (PMC10254562; doi:10.3390/molecules28114466)
Supplement: Supplementary file 1 [file molecules-28-04466-s001.zip › molecules-2398322-supplementary.pdf]

Palladium-Catalyzed *N*-Alkenylation of *N*-Aryl Phosphoramidates with Alkenes

Yu-An Li,[1] Ge Wu,[3] Jia Li*[2]
[1]Department of Orthopaedics Surgery, The Second Affiliated Hospital and Yuying Children's Hospital of Wenzhou Medical University, Wenzhou, China.
[2]Department of Neurology, First Affiliated Hospital of Wenzhou Medical University, Wenzhou, China.
[3]School of Pharmaceutical Sciences, Wenzhou Medical University, Wenzhou, China.

*E-mail: lijia@wzhospital.cn

Table of Contents

(1) Reaction Optimization……………………………………S2

(2) ^{1}H, ^{13}C and ^{19}F NMR spectra of products……..….S3-S54

(3) HRMS spectra of products…………………...…..S55-S71

Reaction Optimization[a]

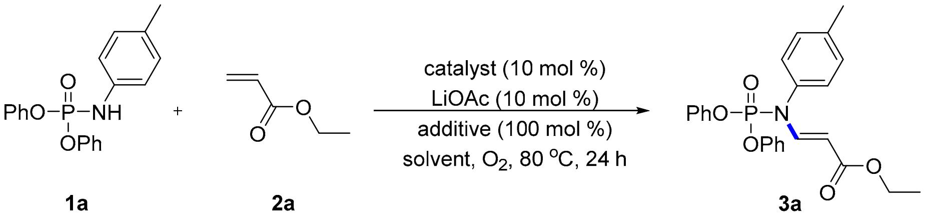

entry	catalyst	additive	solvent	yield (%)[b]
1	$Pd(OAc)_2$	$Cu(OAc)_2$	DME	0
2	$Pd(OAc)_2$	$CuCl_2$	DME	0
3	$Pd(OAc)_2$	BQ	DME	0
4	$Pd(OAc)_2$	pyridine	DME	0
5	$Pd(OAc)_2$	LiBr	DME	0
6	$Pd(OAc)_2$	TBAF	DME	0
7	$Pd(OAc)_2$	TBAC	DME	0
8	$Pd(OAc)_2$	TBAB	DME	65
9	$Pd(OAc)_2$	TBAI	DME	0
10	$PdCl_2$	TBAB	DME	0
11	$Pd(CH_3CN)_2Cl_2$	TBAB	DME	0
12	$Pd(PhCN)_2Cl_2$	TBAB	DME	0
13	$Pd(acac)_2$	TBAB	DME	0
14	$Pd(OAc)_2$	TBAB	THF	45
15	$Pd(OAc)_2$	TBAB	Dioxane	33
16	$Pd(OAc)_2$	TBAB	toluene	21
17	$Pd(OAc)_2$	TBAB	DMSO	0
18	$Pd(OAc)_2$	TBAB	DMF	0
19	$Pd(OAc)_2$	TBAB	TMBE	80
20	$Pd(TFA)_2$	TBAB	TMBE	89
21[c]	$Pd(TFA)_2$	TBAB	TMBE	7
22[d]	$Pd(TFA)_2$	TBAB	TMBE	29
23[e]	$Pd(TFA)_2$	TBAB	TMBE	77
24[f]	$Pd(TFA)_2$	TBAB	TMBE	72
25[g]	$Pd(TFA)_2$	TBAB	TMBE	71
26		TBAB	TMBE	0

[a] Reaction conditions: **1a** (0.2 mmol), **2a** (0.6 mmol), catalyst (0.02 mmol), LiOAc (0.02 mmol), additive (0.2 mmol), solvent (2 ml), under O_2, heated at 80 °C for 24 h. [b] Isolated yield. [c] Under N_2 atmosphere. [d] Under air atmosphere. [e] at 70 °C. [f] at 90 °C. [g] 80% TBAB.

Invalid substrate

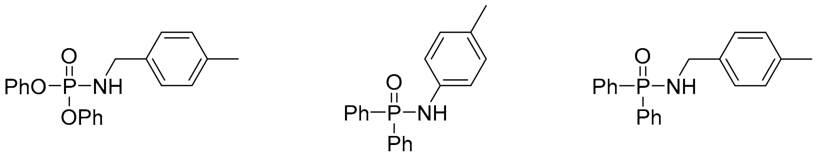

^1H, ^{13}C, ^{31}P and ^{19}F NMR spectra of products

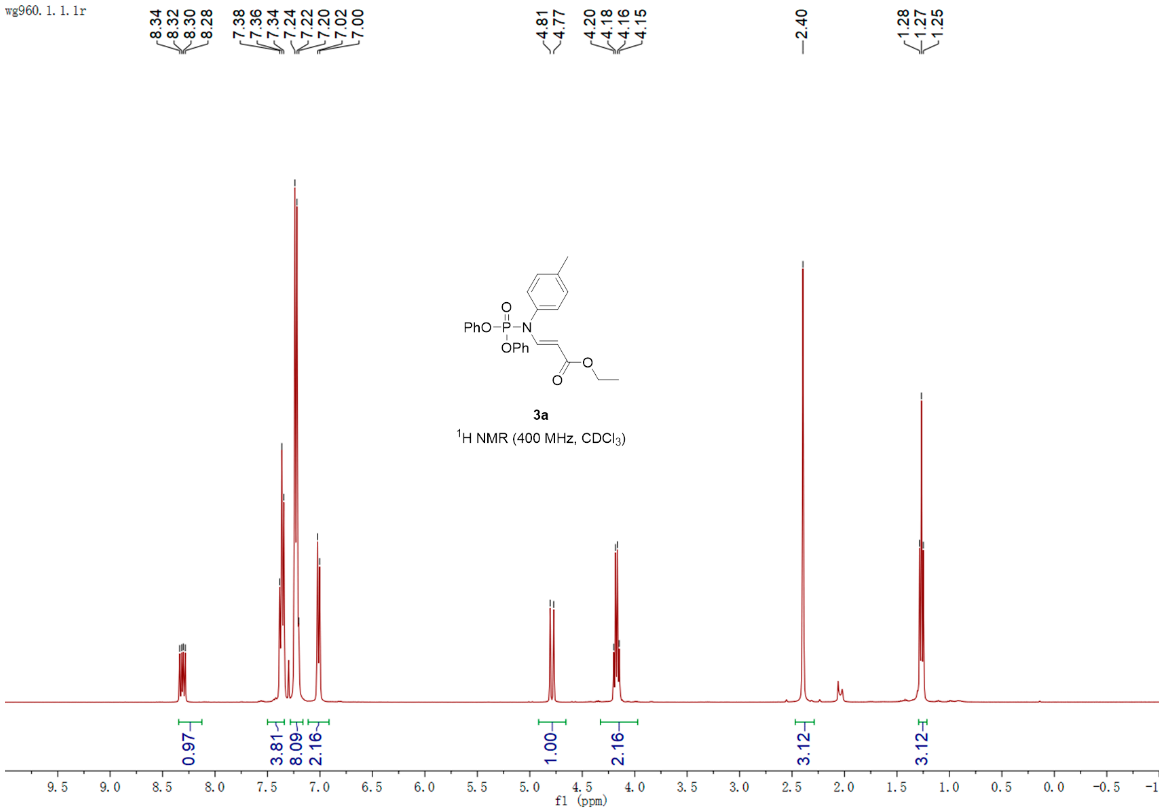

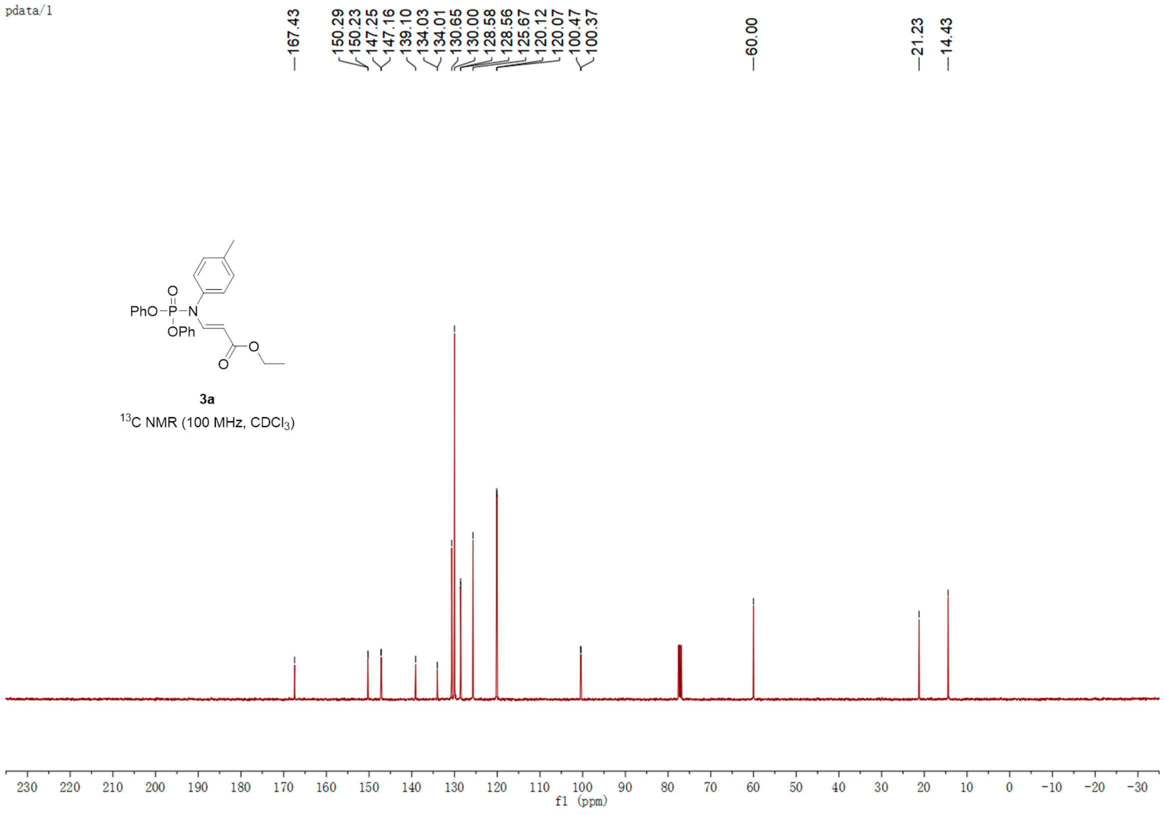

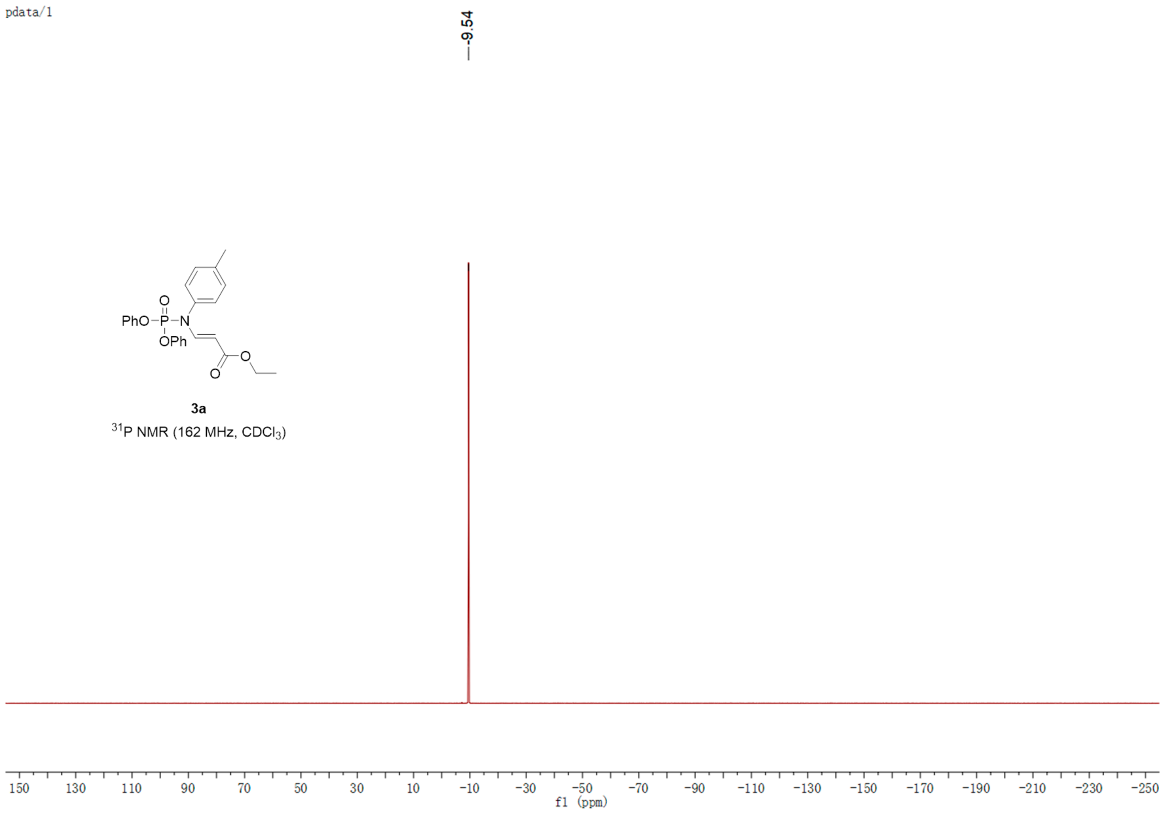

3a

^{31}P NMR (162 MHz, $CDCl_3$)

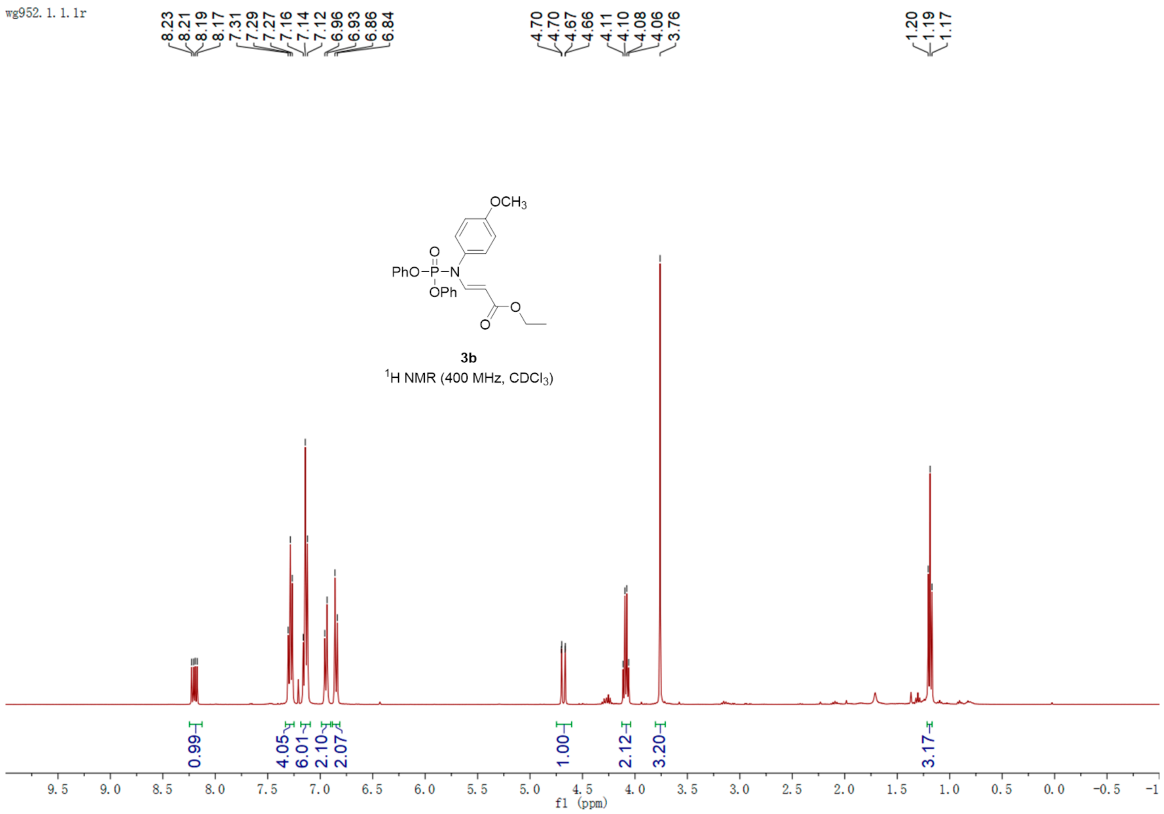

3b

^{1}H NMR (400 MHz, $CDCl_3$)

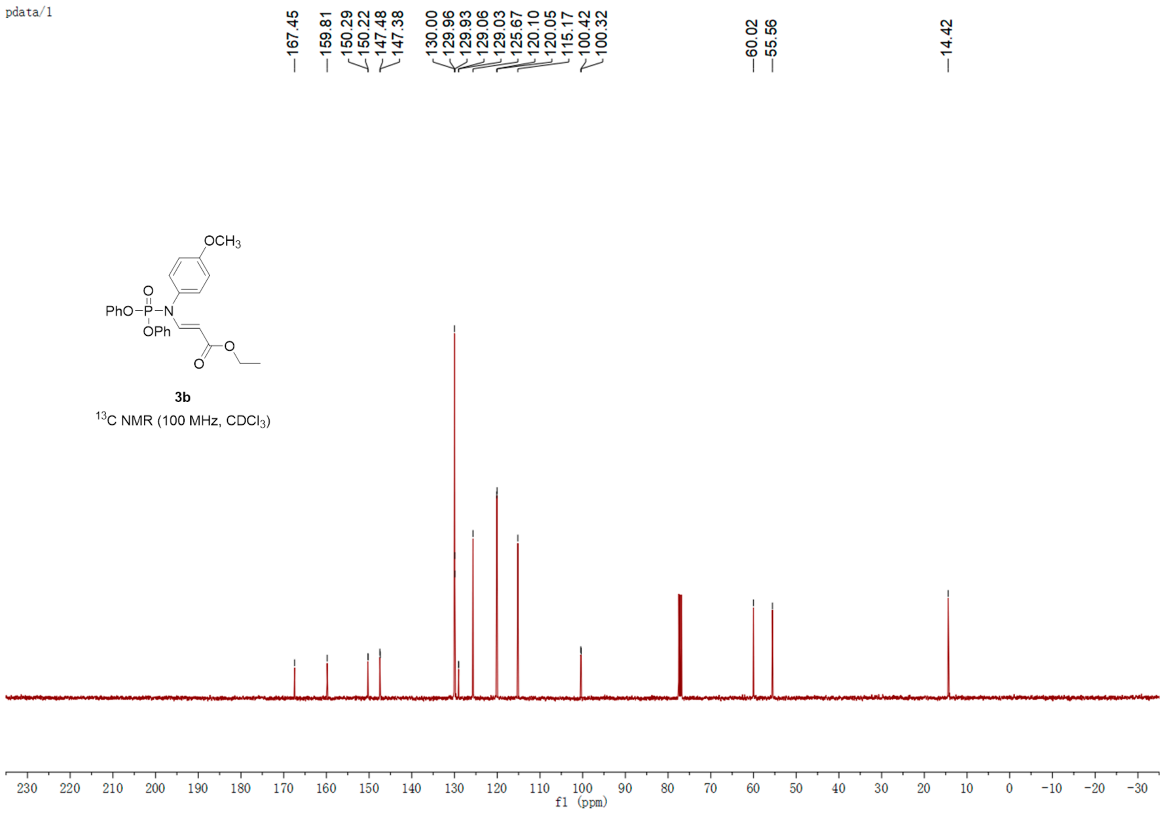
pdata/1
167.45
159.81
150.29
150.22
147.48
147.38
130.00
129.96
129.93
129.06
129.03
125.67
120.10
120.05
115.17
100.42
100.32
60.02
55.56
14.42
OCH3
PhO
P
N
O
OPh
O
O
3b
13C NMR (100 MHz, CDCl3)
230 220 210 200 190 180 170 160 150 140 130 120 110 100 90 80 70 60 50 40 30 20 10 0 -10 -20 -30
f1 (ppm)

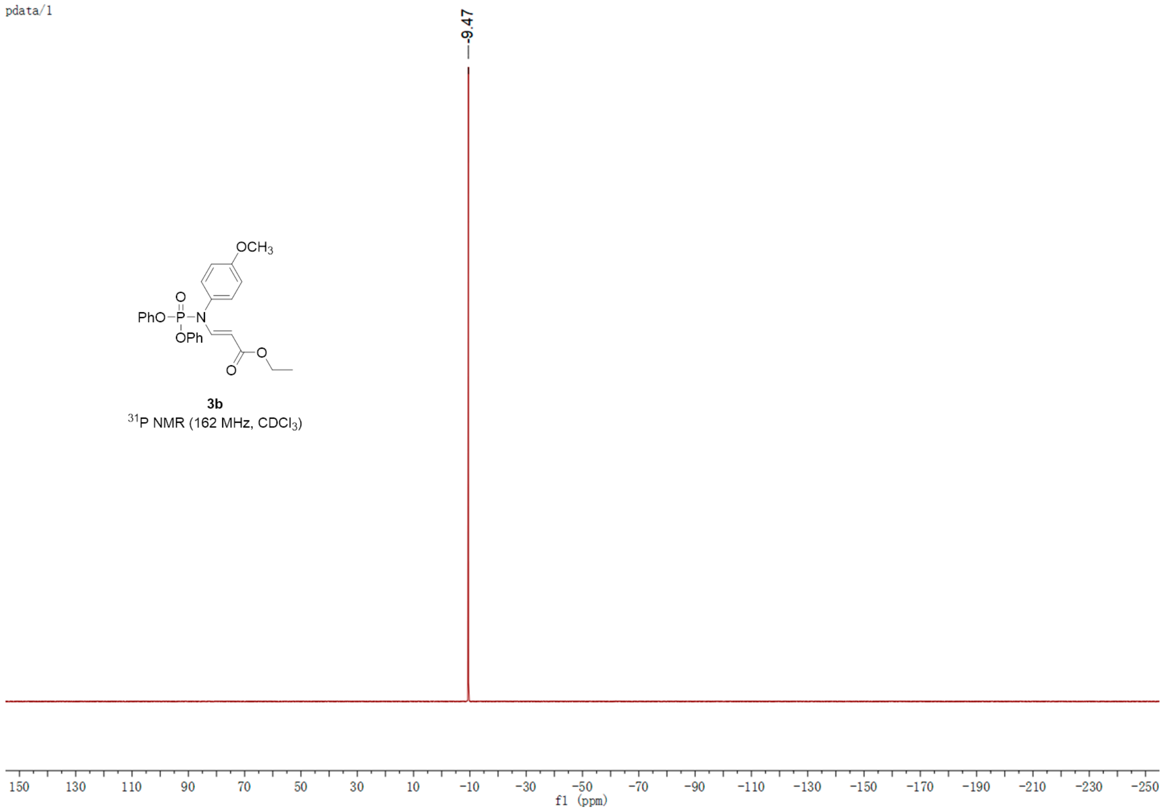
pdata/1
-9.47
OCH3
PhO
P
N
O
OPh
O
O
3b
31P NMR (162 MHz, CDCl3)
150 130 110 90 70 50 30 10 -10 -30 -50 -70 -90 -110 -130 -150 -170 -190 -210 -230 -250
f1 (ppm)

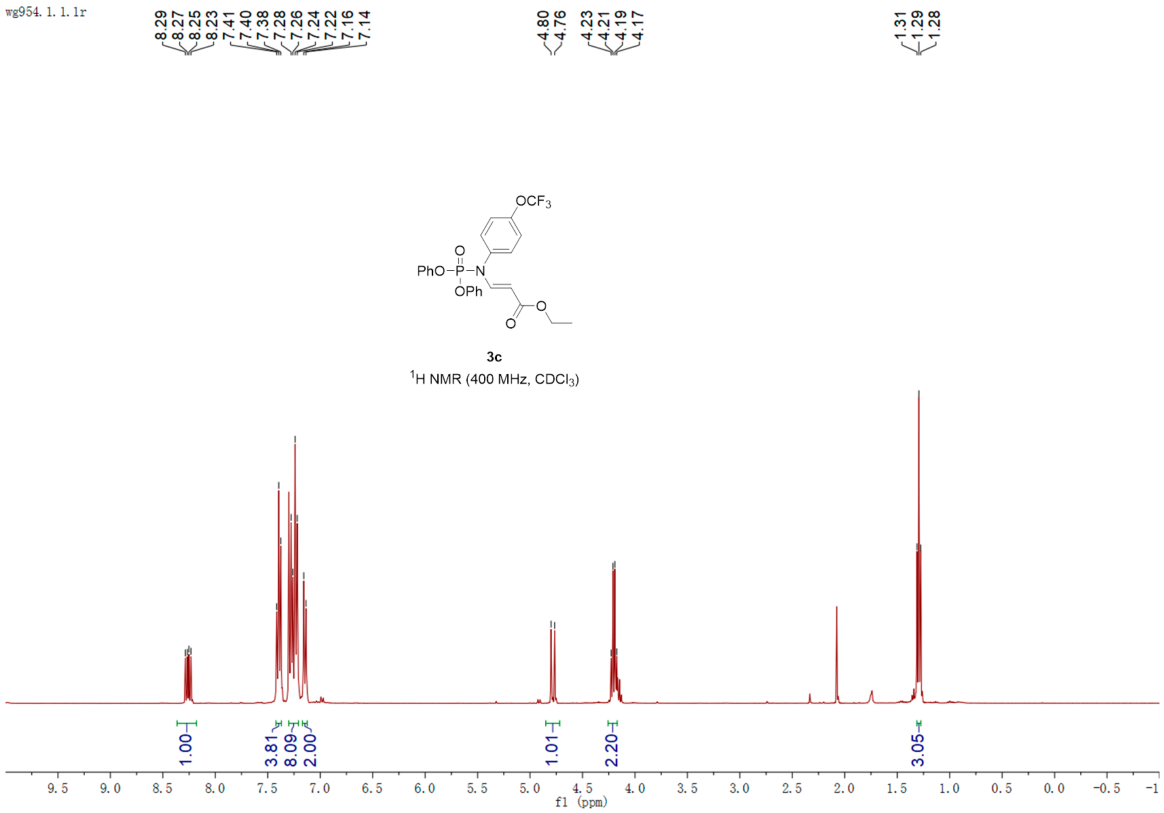
wg954.1.1.1r
8.29 8.27 8.25 8.23 7.41 7.40 7.38 7.28 7.26 7.24 7.22 7.16 7.14
4.80 4.76
4.23 4.21 4.19 4.17
1.31 1.29 1.28
OCF3
O
PhO-P-N
OPh
O
O
3c
1H NMR (400 MHz, CDCl3)
1.00 3.81 8.09 2.00 1.01 2.20 3.05
9.5 9.0 8.5 8.0 7.5 7.0 6.5 6.0 5.5 5.0 4.5 4.0 3.5 3.0 2.5 2.0 1.5 1.0 0.5 0.0 -0.5 -1
f1 (ppm)

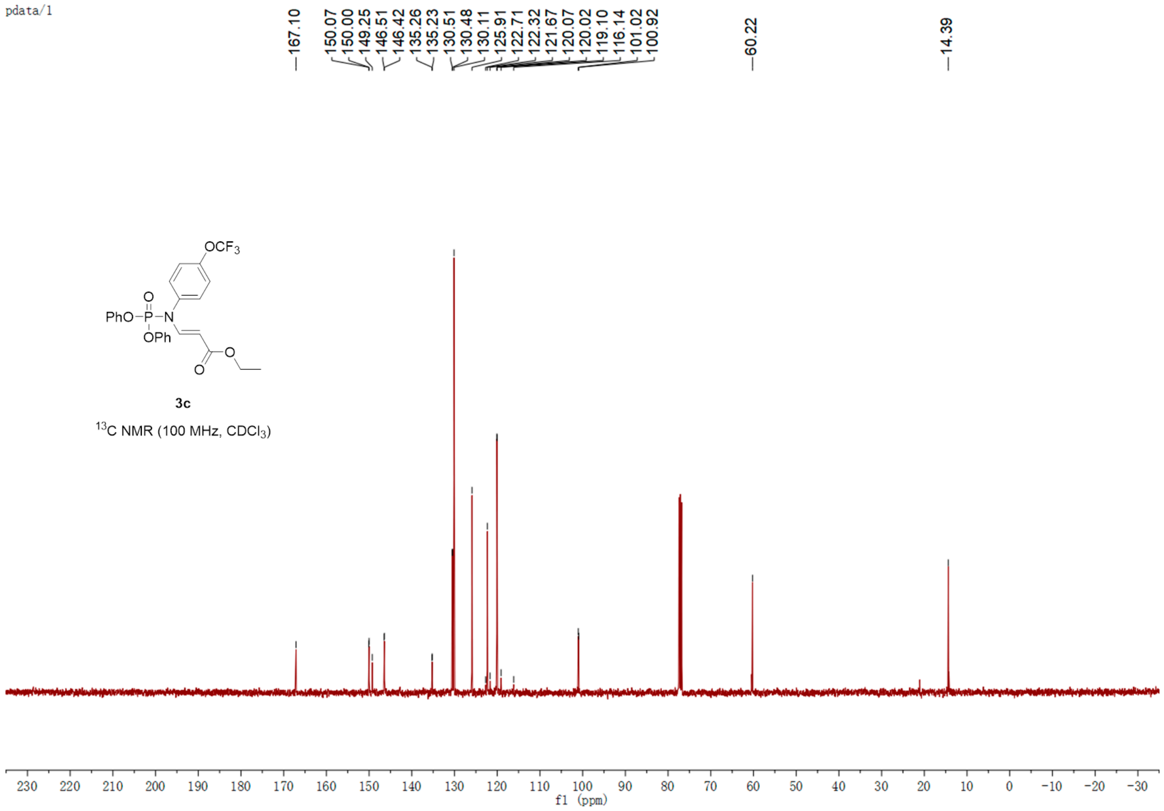
pdata/1
167.10
150.07 150.00 149.25 146.51 146.42 135.26 135.23 130.51 130.48 130.11 125.91 122.71 122.32 121.67 120.07 120.02 119.10 116.14 101.02 100.92
60.22
14.39
OCF3
O
PhO-P-N
OPh
O
O
3c
13C NMR (100 MHz, CDCl3)
230 220 210 200 190 180 170 160 150 140 130 120 110 100 90 80 70 60 50 40 30 20 10 0 -10 -20 -30
f1 (ppm)

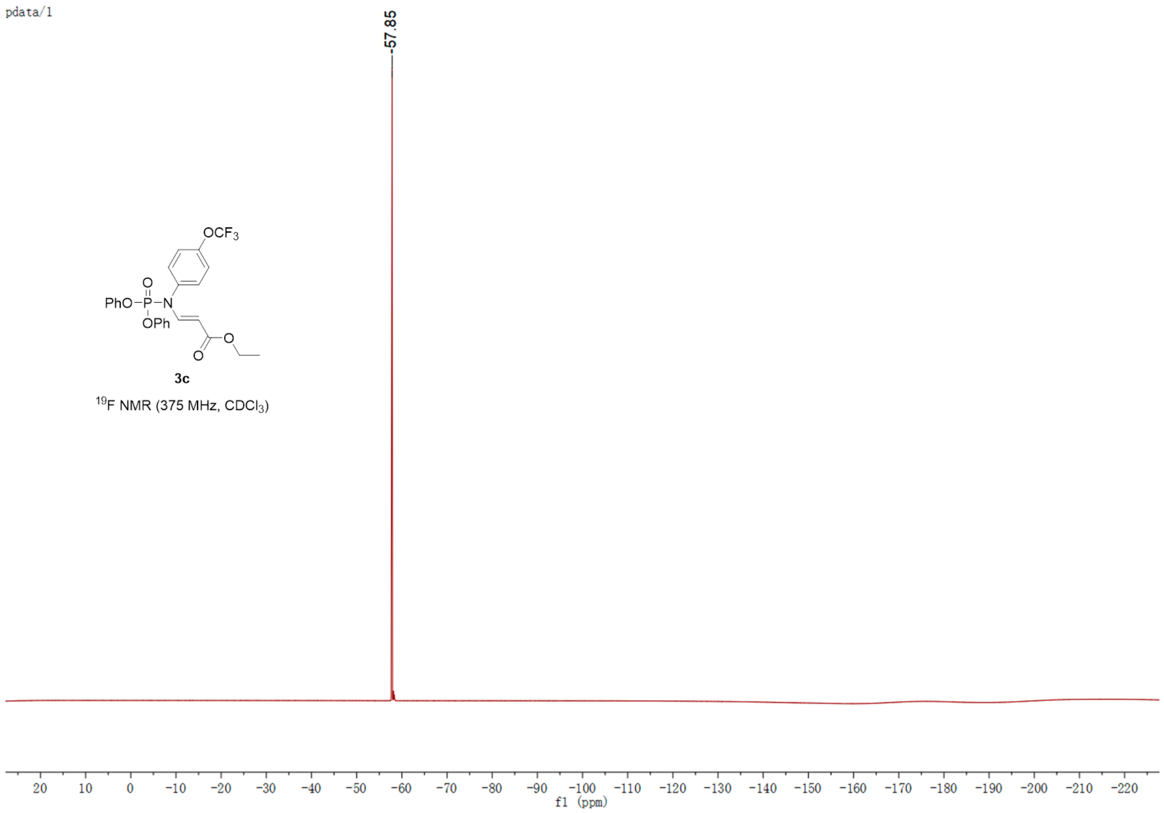
pdata/1
-57.85
OCF3
PhO
OPh
3c
19F NMR (375 MHz, CDCl3)
f1 (ppm)

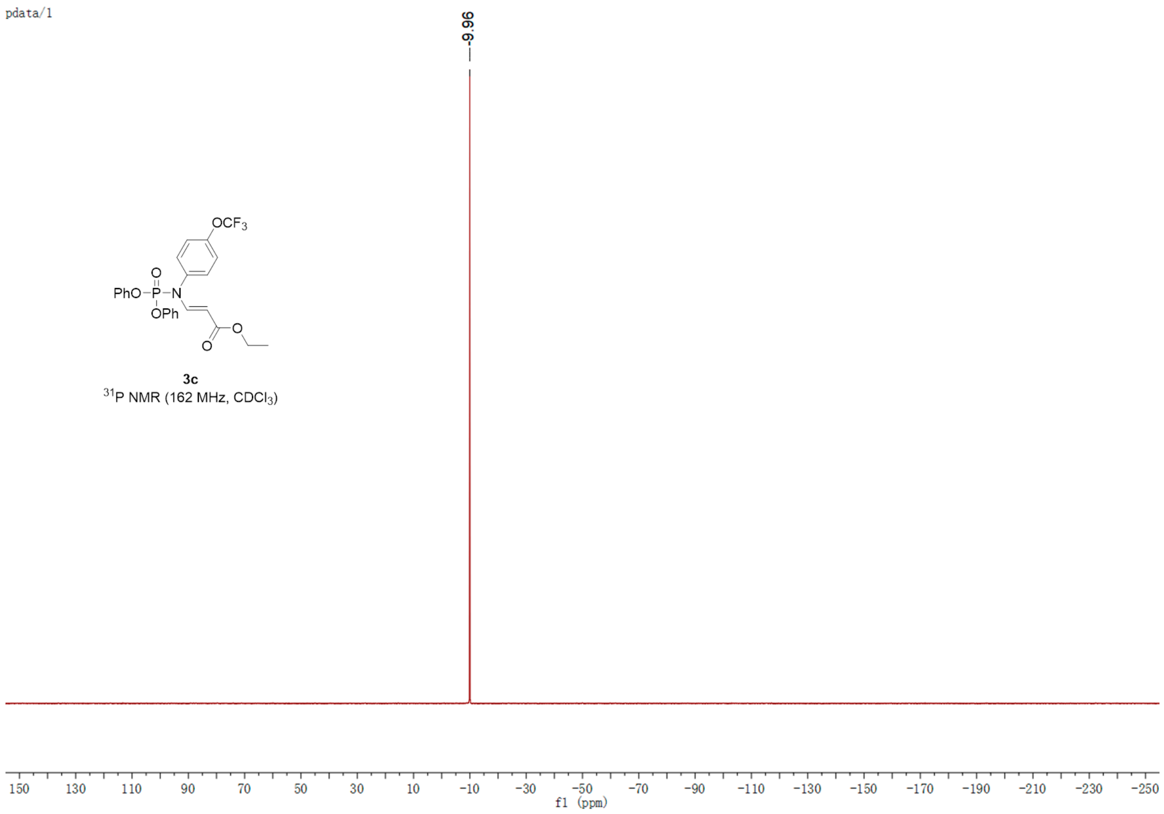
pdata/1
-9.96
OCF3
PhO
OPh
3c
31P NMR (162 MHz, CDCl3)
f1 (ppm)

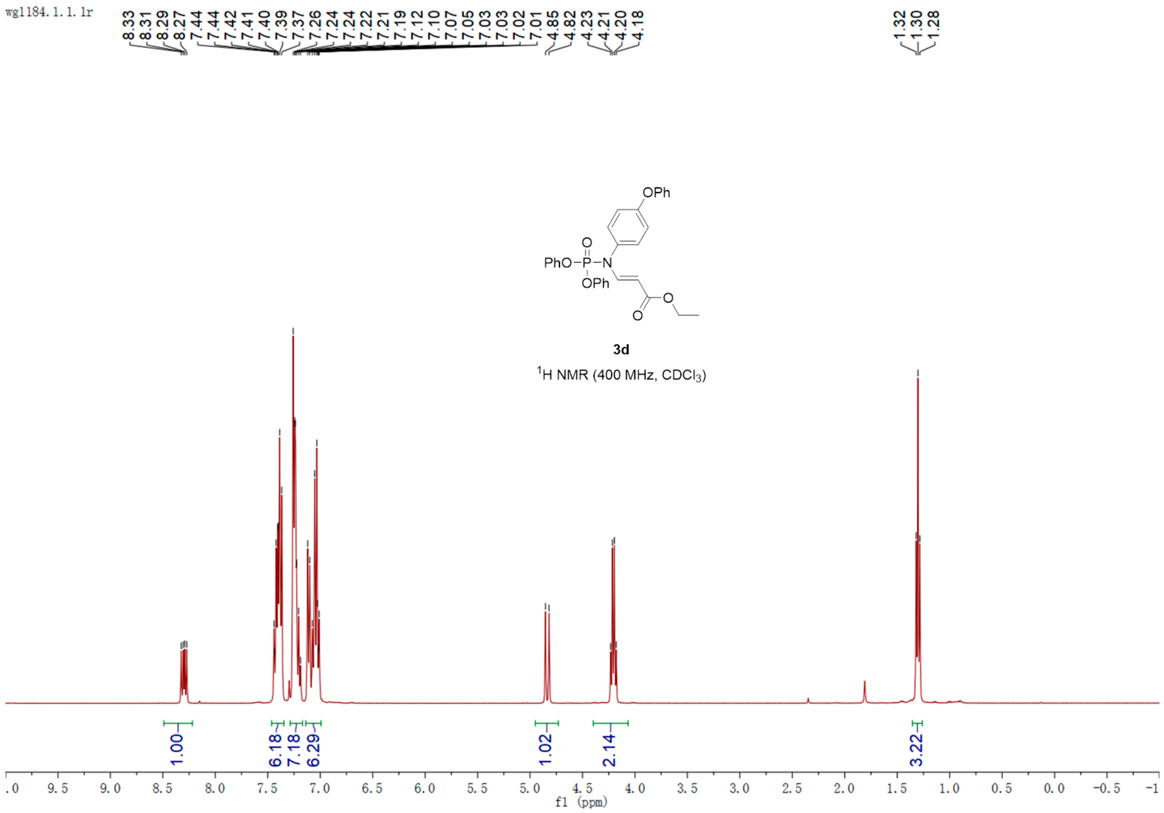
wg1184.1.1.1r
8.33
8.31
8.29
8.27
7.44
7.44
7.42
7.41
7.40
7.39
7.37
7.26
7.24
7.24
7.22
7.21
7.19
7.12
7.10
7.07
7.05
7.03
7.03
7.02
7.01
4.85
4.82
4.23
4.21
4.20
4.18
1.32
1.30
1.28
OPh
PhO
OPh
O
N
P
O
O
3d
1H NMR (400 MHz, CDCl3)
1.00
6.18
7.18
6.29
1.02
2.14
3.22
f1 (ppm)

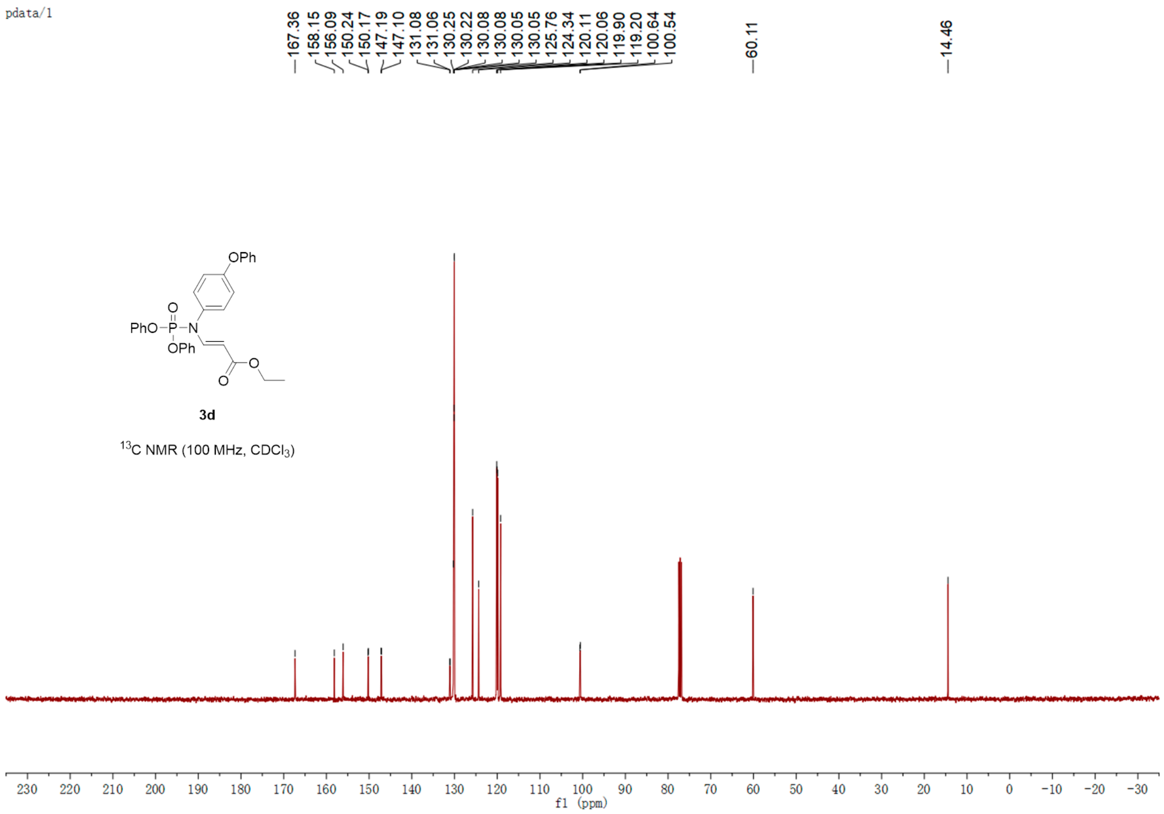
pdata/1
167.36
158.15
156.09
150.24
150.17
147.19
147.10
131.08
131.06
130.25
130.22
130.08
130.08
130.05
130.05
125.76
124.34
120.11
120.06
119.90
119.20
100.64
100.54
60.11
14.46
OPh
PhO
OPh
3d
13C NMR (100 MHz, CDCl3)
f1 (ppm)

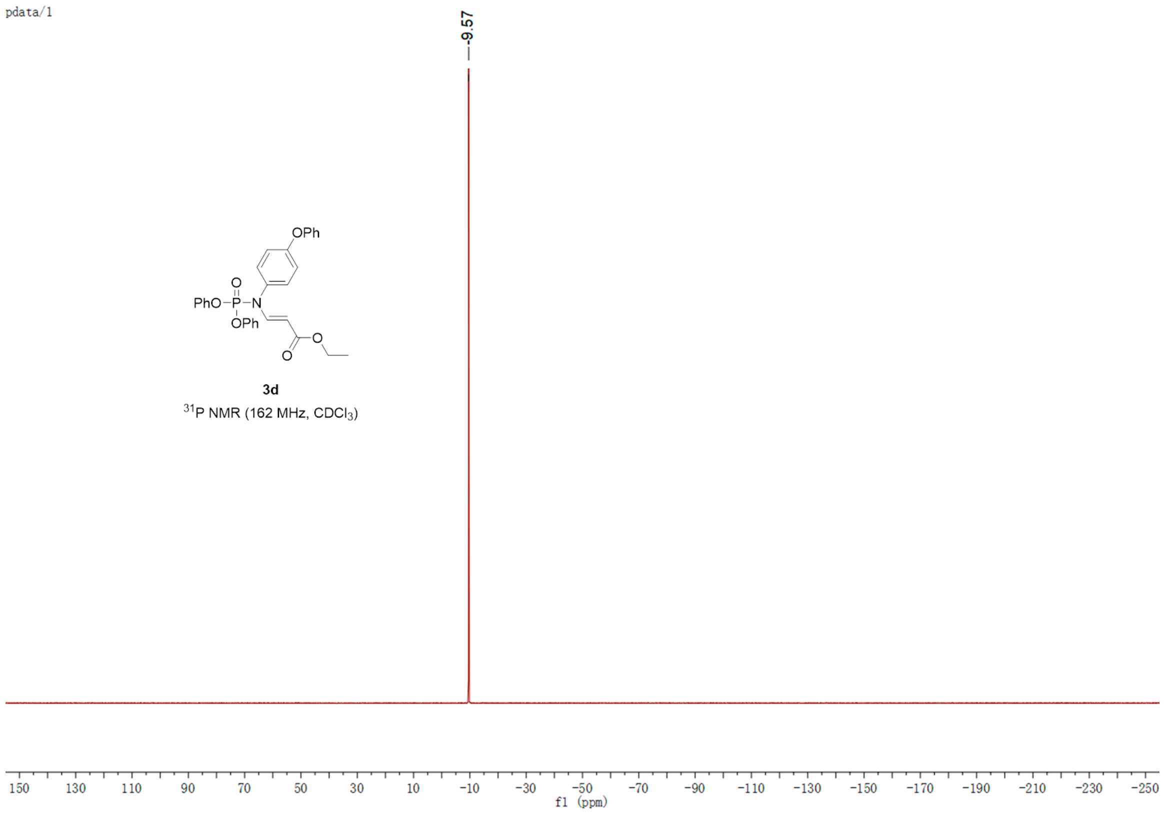

3d

^{31}P NMR (162 MHz, $CDCl_3$)

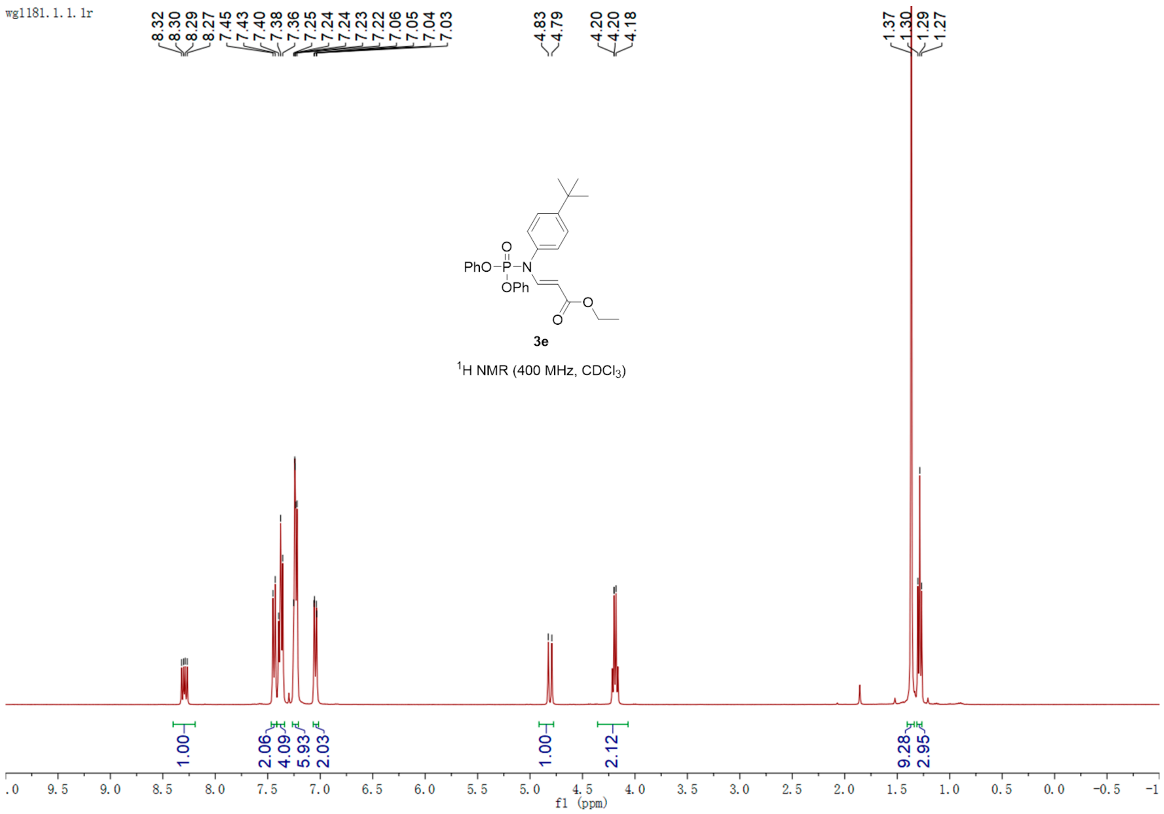

3e

^{1}H NMR (400 MHz, $CDCl_3$)

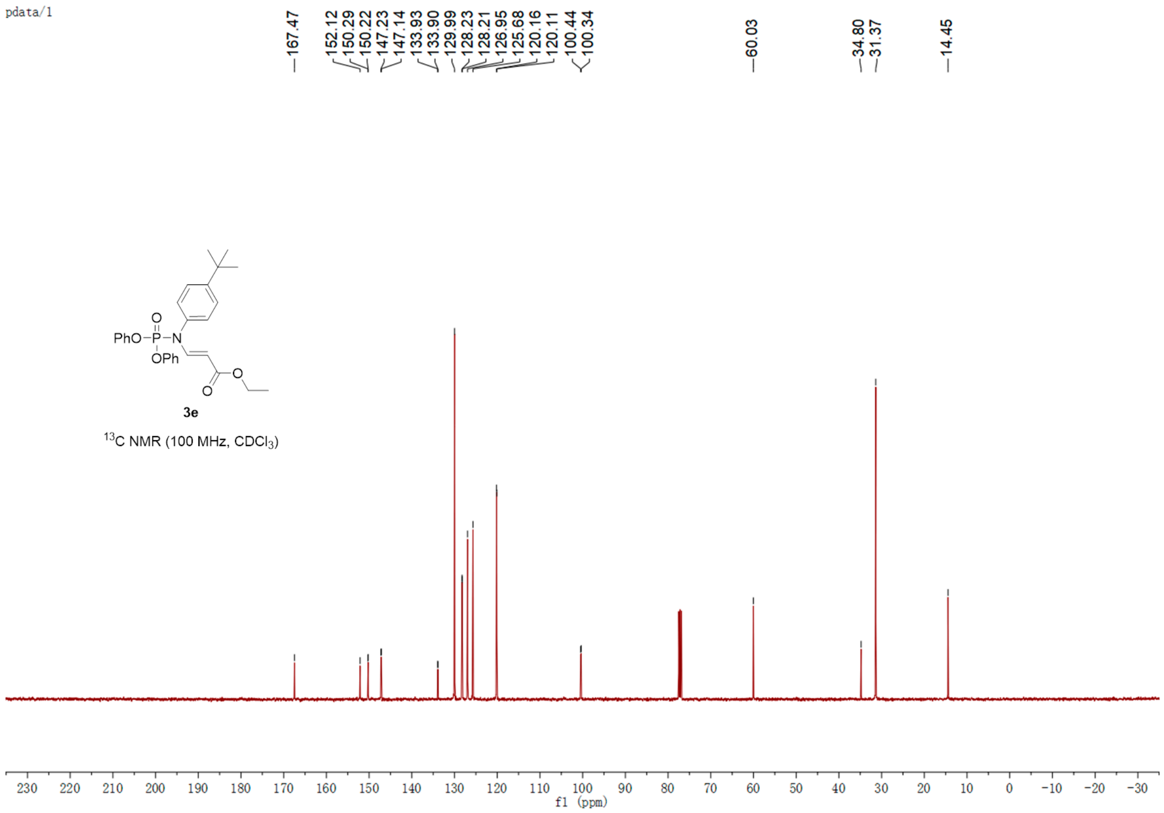
pdata/1
167.47
152.12
150.29
150.22
147.23
147.14
133.93
133.90
129.99
128.23
128.21
126.95
125.68
120.16
120.11
100.44
100.34
60.03
34.80
31.37
14.45
PhO
P
N
O
OPh
O
O
3e
^{13}C NMR (100 MHz, $CDCl_3$)
230 220 210 200 190 180 170 160 150 140 130 120 110 100 90 80 70 60 50 40 30 20 10 0 -10 -20 -30
f1 (ppm)

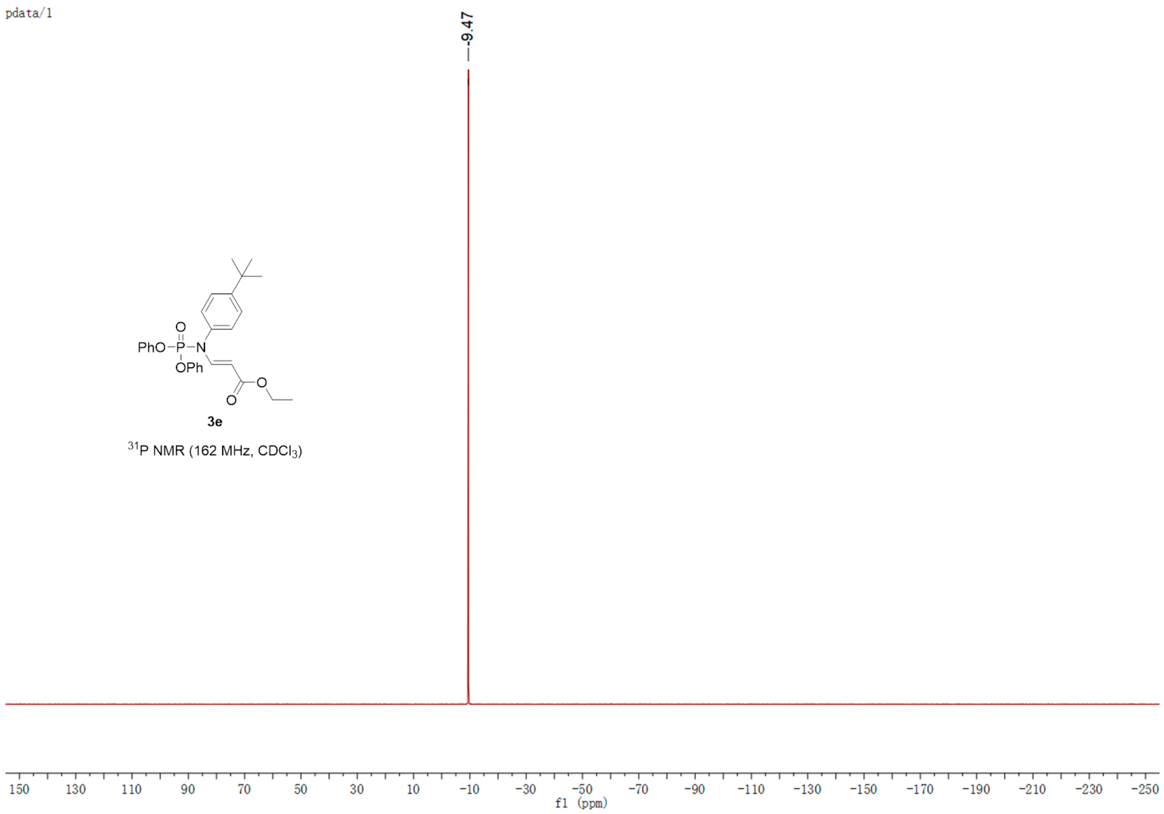
pdata/1
-9.47
PhO
P
N
O
OPh
O
O
3e
^{31}P NMR (162 MHz, $CDCl_3$)
150 130 110 90 70 50 30 10 -10 -30 -50 -70 -90 -110 -130 -150 -170 -190 -210 -230 -250
f1 (ppm)

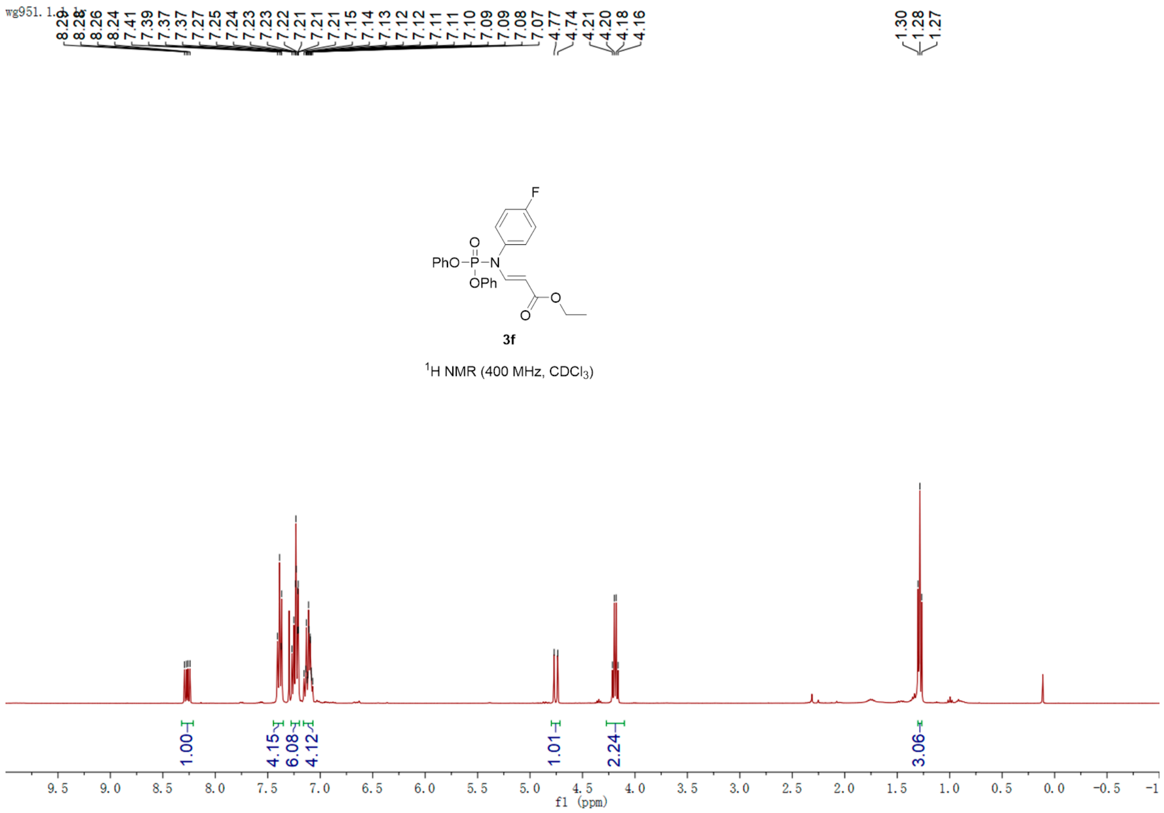

3f

^{1}H NMR (400 MHz, $CDCl_3$)

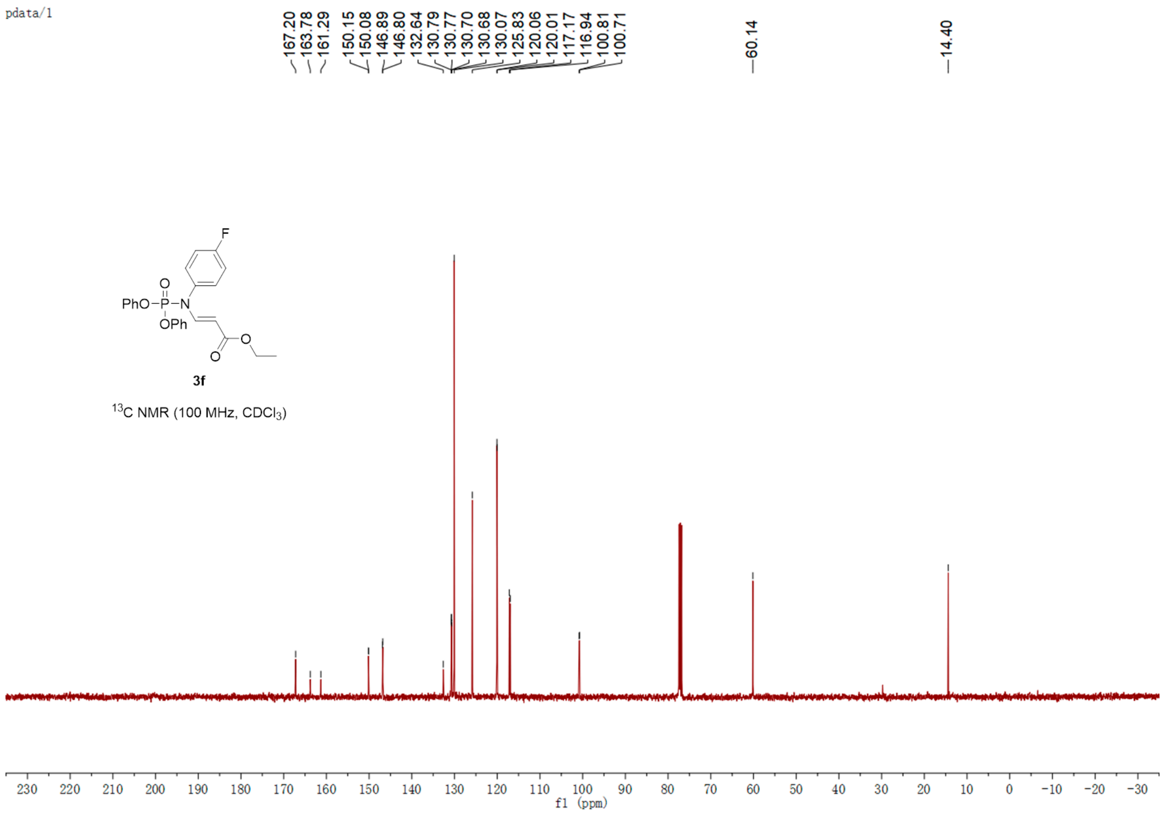

3f

^{13}C NMR (100 MHz, $CDCl_3$)

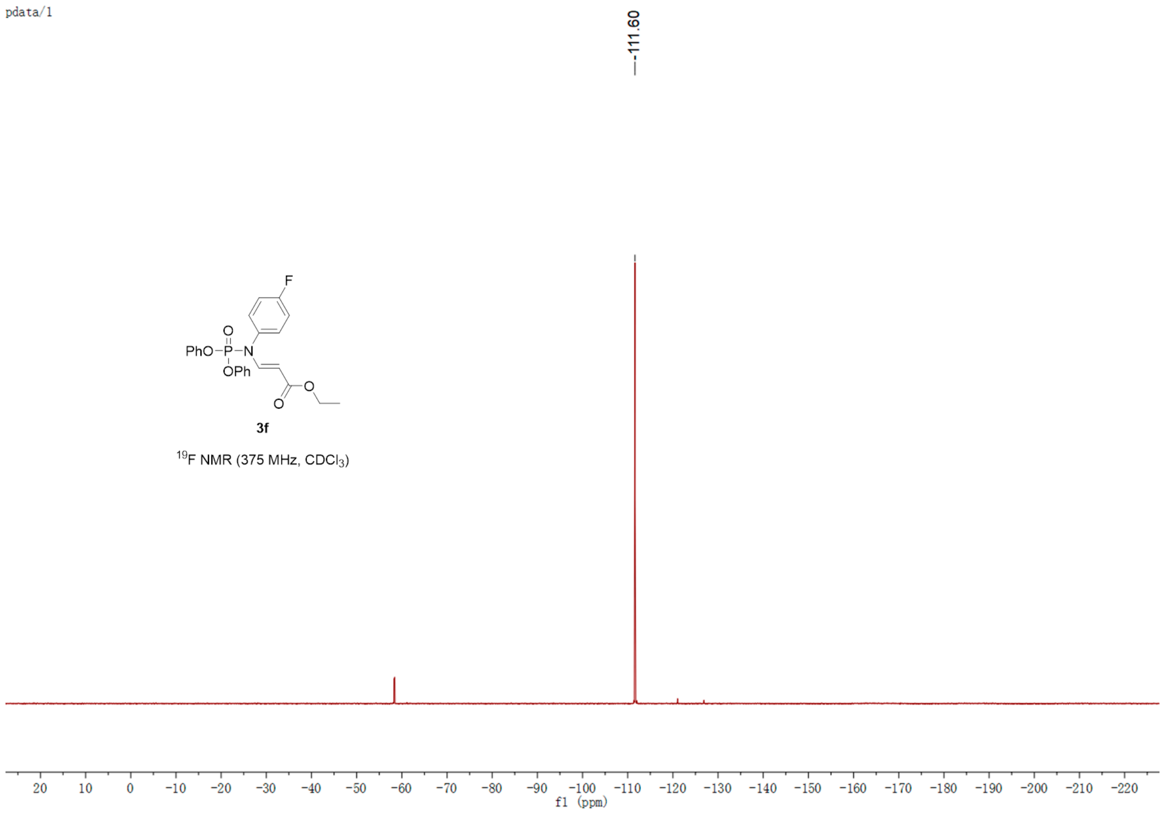
pdata/1
-111.60
F
O
PhO-P-N
OPh
O
O
3f
19F NMR (375 MHz, CDCl3)
20 10 0 -10 -20 -30 -40 -50 -60 -70 -80 -90 -100 -110 -120 -130 -140 -150 -160 -170 -180 -190 -200 -210 -220
f1 (ppm)

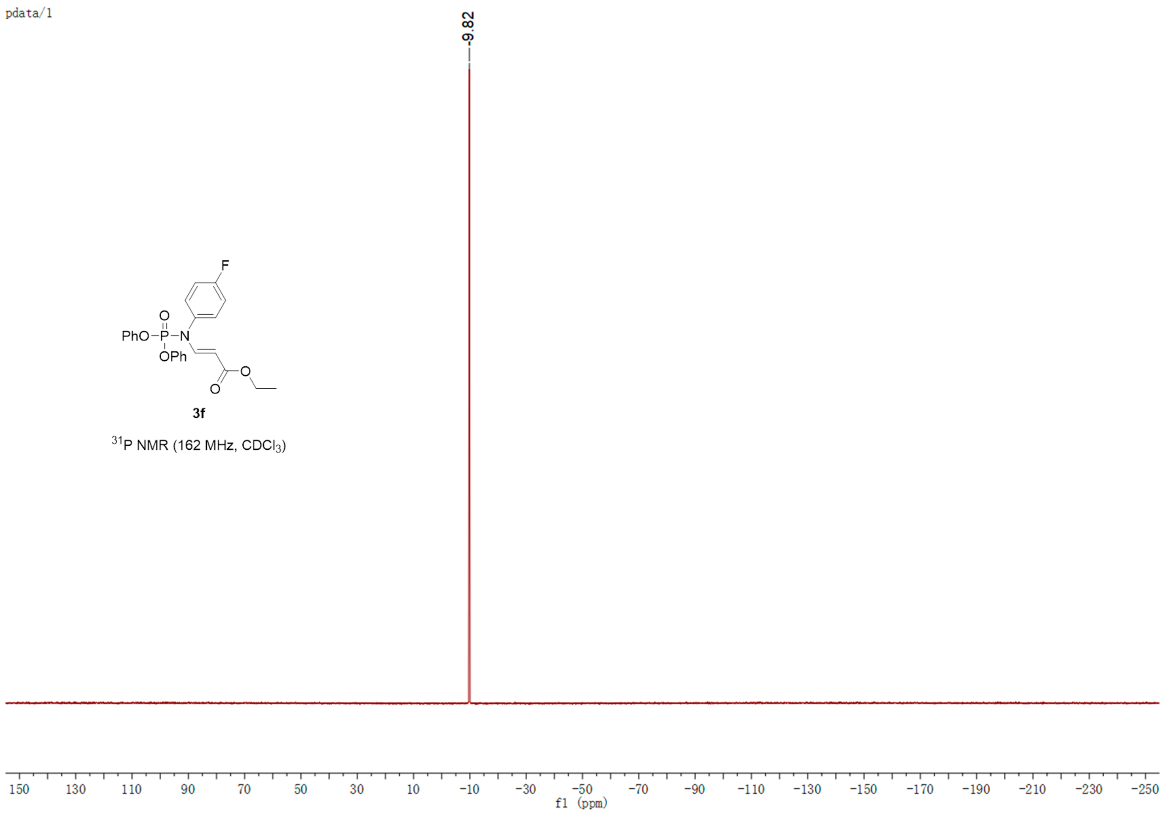
pdata/1
-9.82
F
O
PhO-P-N
OPh
O
O
3f
31P NMR (162 MHz, CDCl3)
150 130 110 90 70 50 30 10 -10 -30 -50 -70 -90 -110 -130 -150 -170 -190 -210 -230 -250
f1 (ppm)

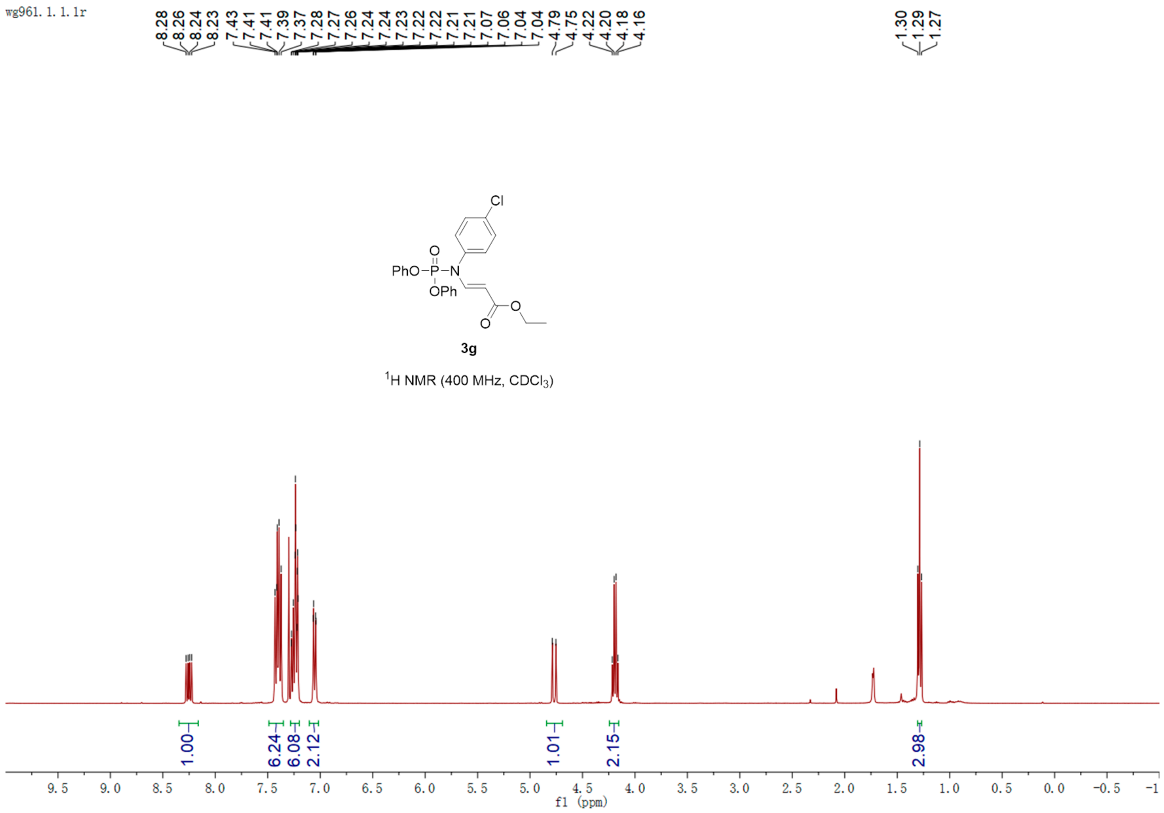

wg961.1.1.1r
8.28 8.26 8.24 8.23 7.43 7.41 7.41 7.39 7.37 7.28 7.27 7.26 7.24 7.24 7.23 7.22 7.22 7.21 7.21 7.07 7.06 7.04 7.04 4.79 4.75 4.22 4.20 4.18 4.16
1.30 1.29 1.27
Cl
PhO
P
N
O
OPh
O
O
3g
^{1}H NMR (400 MHz, $CDCl_3$)
1.00 6.24 6.08 2.12 1.01 2.15 2.98
9.5 9.0 8.5 8.0 7.5 7.0 6.5 6.0 5.5 5.0 4.5 4.0 3.5 3.0 2.5 2.0 1.5 1.0 0.5 0.0 -0.5 -1
f1 (ppm)

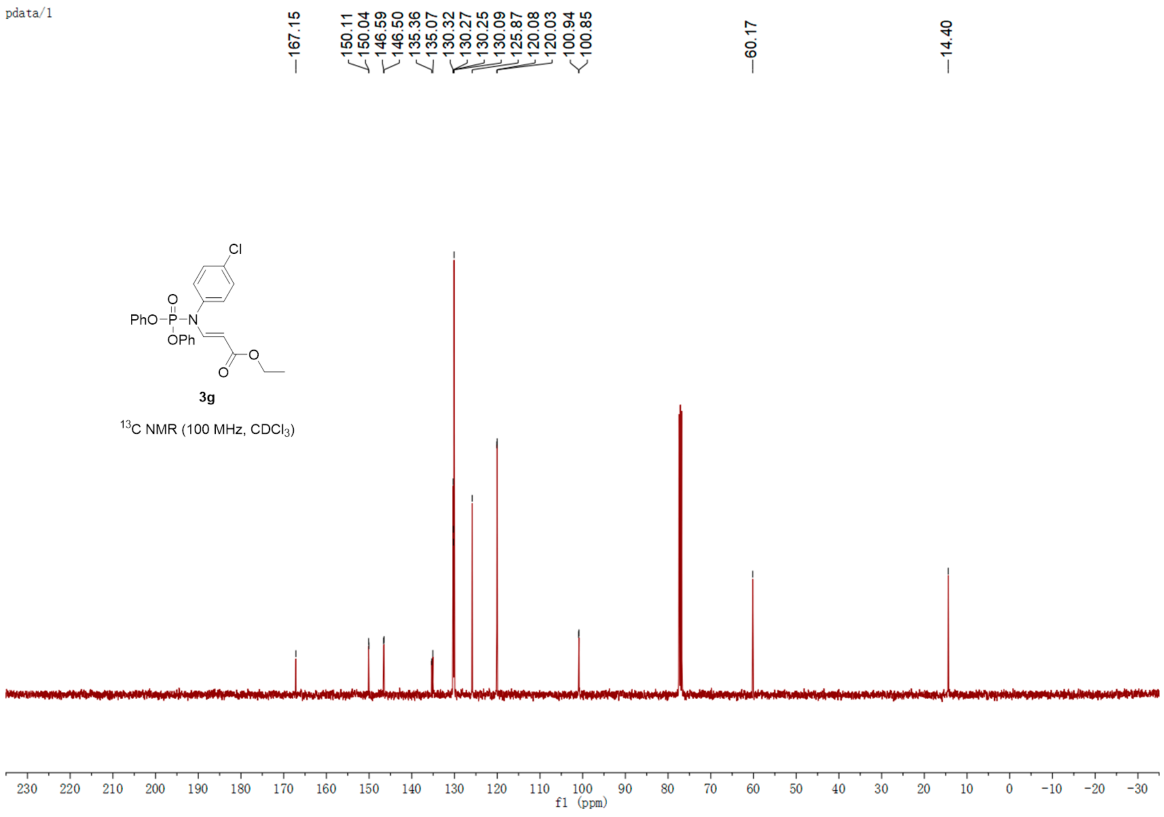

pdata/1
167.15
150.11 150.04 146.59 146.50 135.36 135.07 130.32 130.27 130.25 130.09 125.87 120.08 120.03 100.94 100.85
60.17
14.40
Cl
PhO
P
N
O
OPh
O
O
3g
^{13}C NMR (100 MHz, $CDCl_3$)
230 220 210 200 190 180 170 160 150 140 130 120 110 100 90 80 70 60 50 40 30 20 10 0 -10 -20 -30
f1 (ppm)

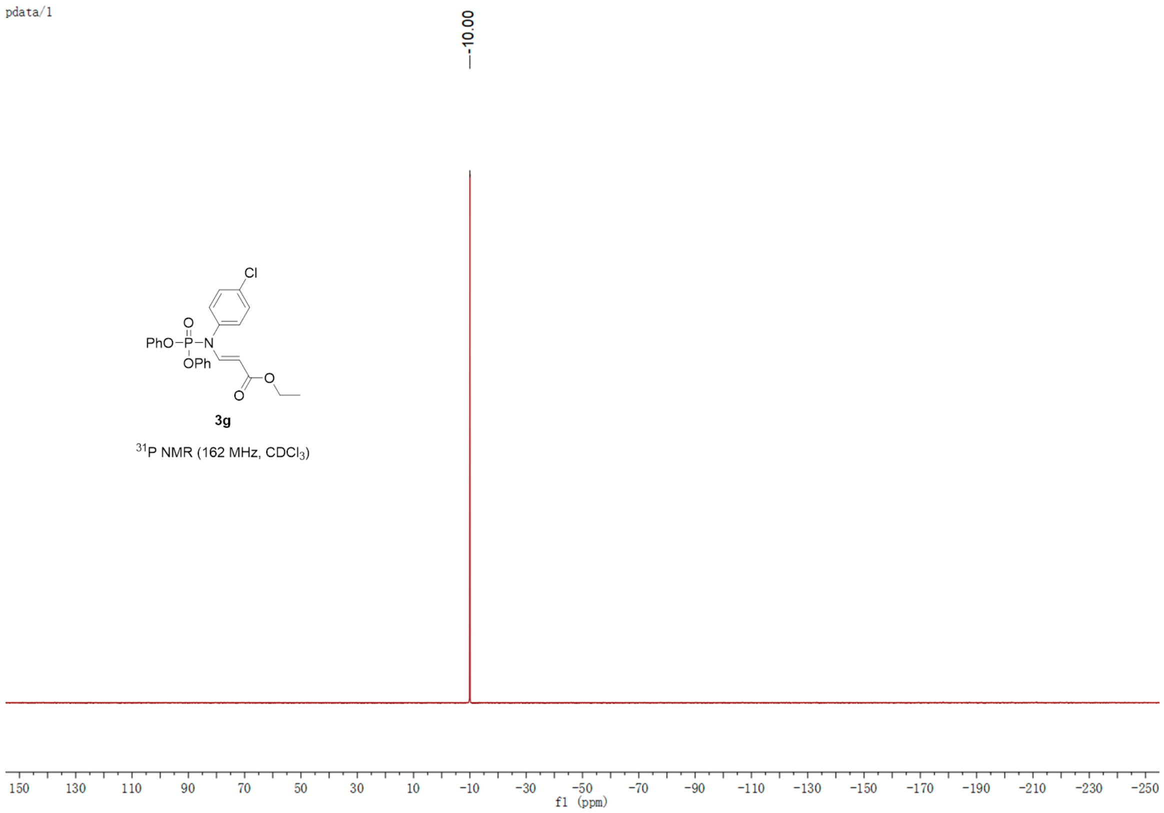

3g

^{31}P NMR (162 MHz, $CDCl_3$)

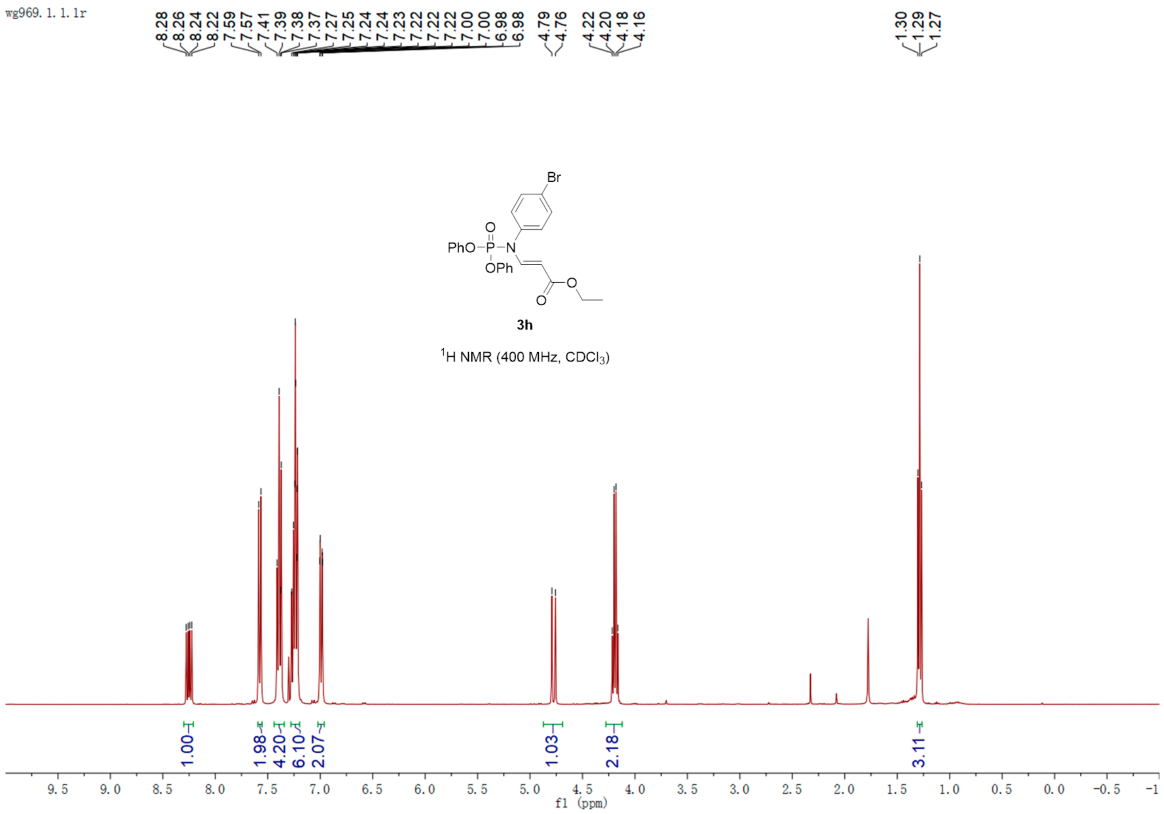

3h

^{1}H NMR (400 MHz, $CDCl_3$)

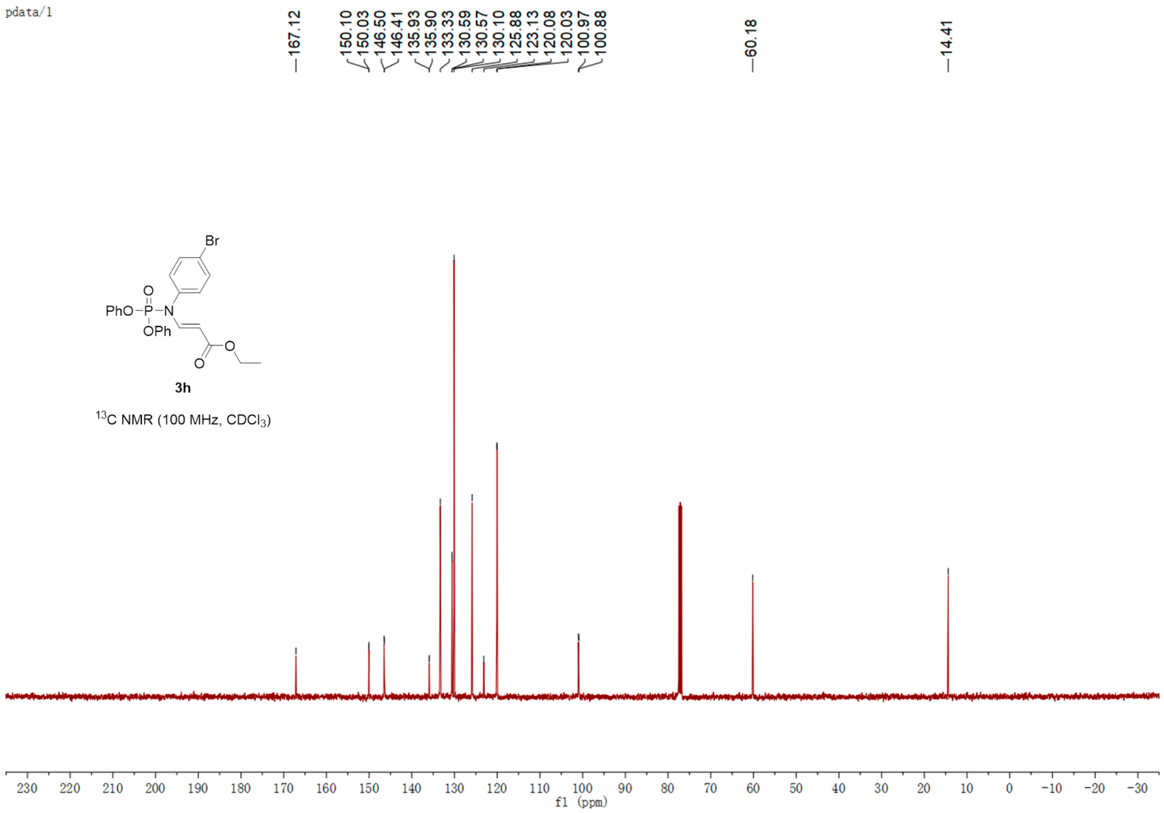
pdata/1
167.12
150.10
150.03
146.50
146.41
135.93
135.90
133.33
130.59
130.57
130.10
125.88
123.13
120.08
120.03
100.97
100.88
60.18
14.41
Br
O
PhO
P
N
OPh
O
O
3h
13C NMR (100 MHz, CDCl3)
f1 (ppm)

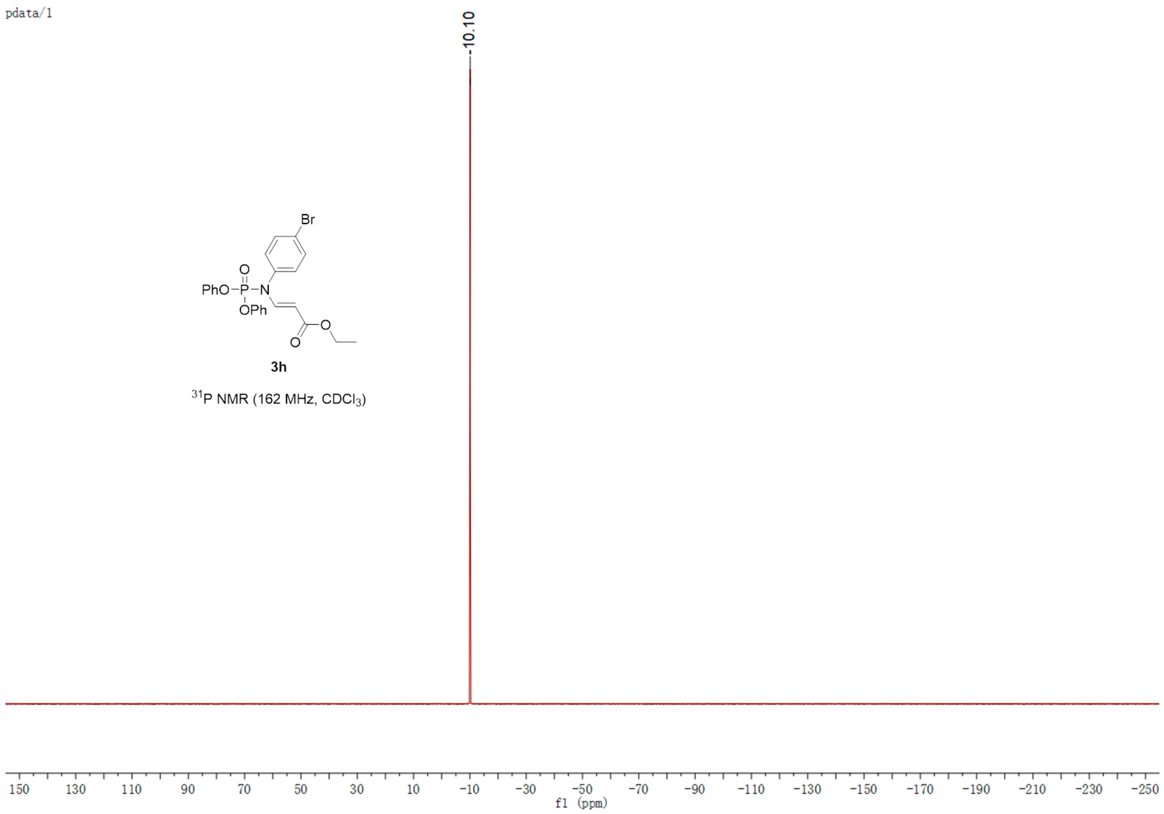
pdata/1
-10.10
Br
O
PhO
P
N
OPh
O
O
3h
31P NMR (162 MHz, CDCl3)
f1 (ppm)

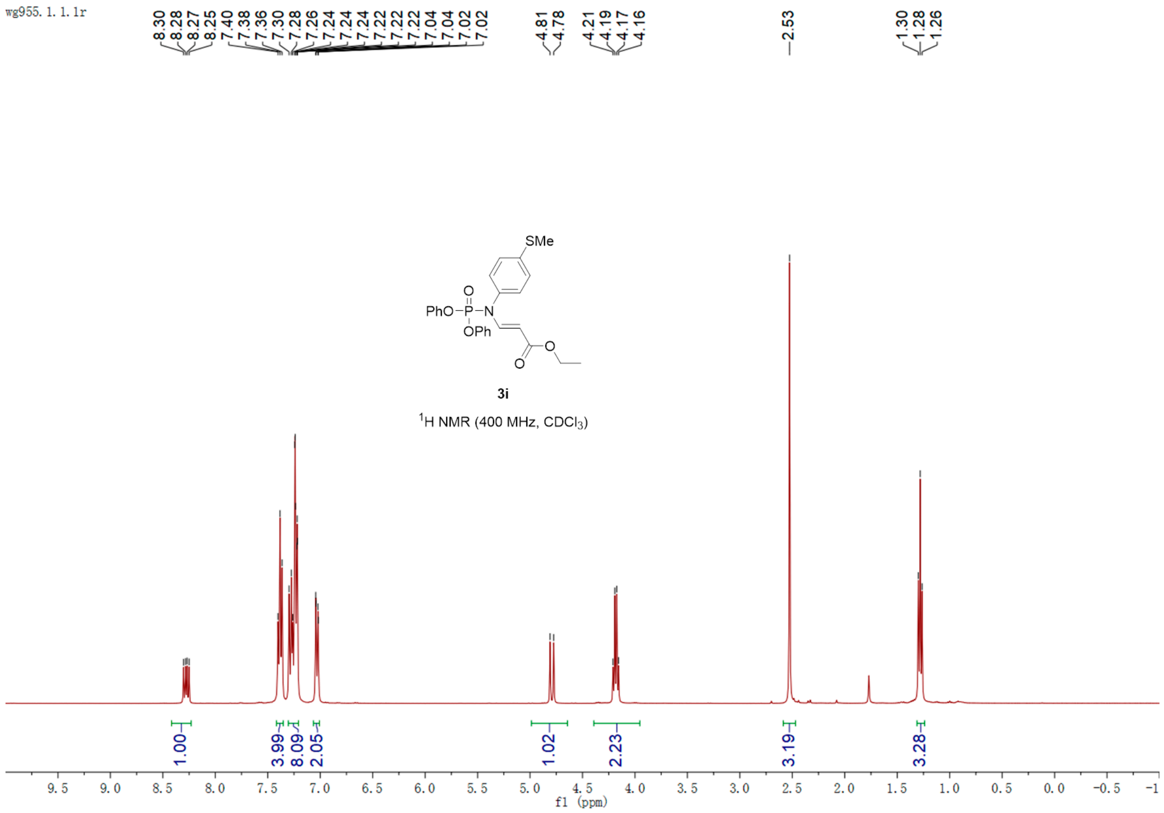
wg955.1.1.1r
8.30
8.28
8.27
8.25
7.40
7.38
7.36
7.30
7.28
7.26
7.24
7.24
7.24
7.22
7.22
7.22
7.04
7.04
7.02
7.02
4.81
4.78
4.21
4.19
4.17
4.16
2.53
1.30
1.28
1.26
SMe
PhO
OPh
3i
1H NMR (400 MHz, CDCl3)
1.00
3.99
8.09
2.05
1.02
2.23
3.19
3.28
f1 (ppm)

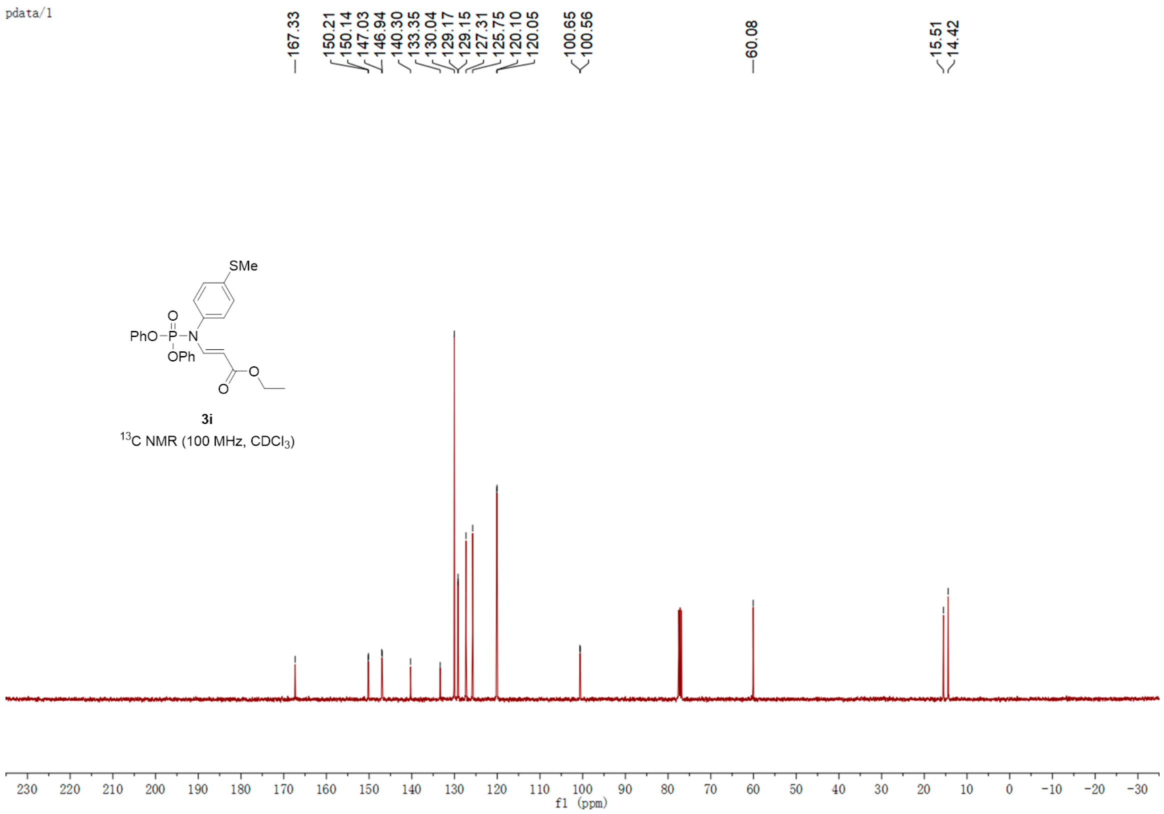
pdata/1
167.33
150.21
150.14
147.03
146.94
140.30
133.35
130.04
129.17
129.15
127.31
125.75
120.10
120.05
100.65
100.56
60.08
15.51
14.42
SMe
PhO
OPh
3i
13C NMR (100 MHz, CDCl3)
f1 (ppm)

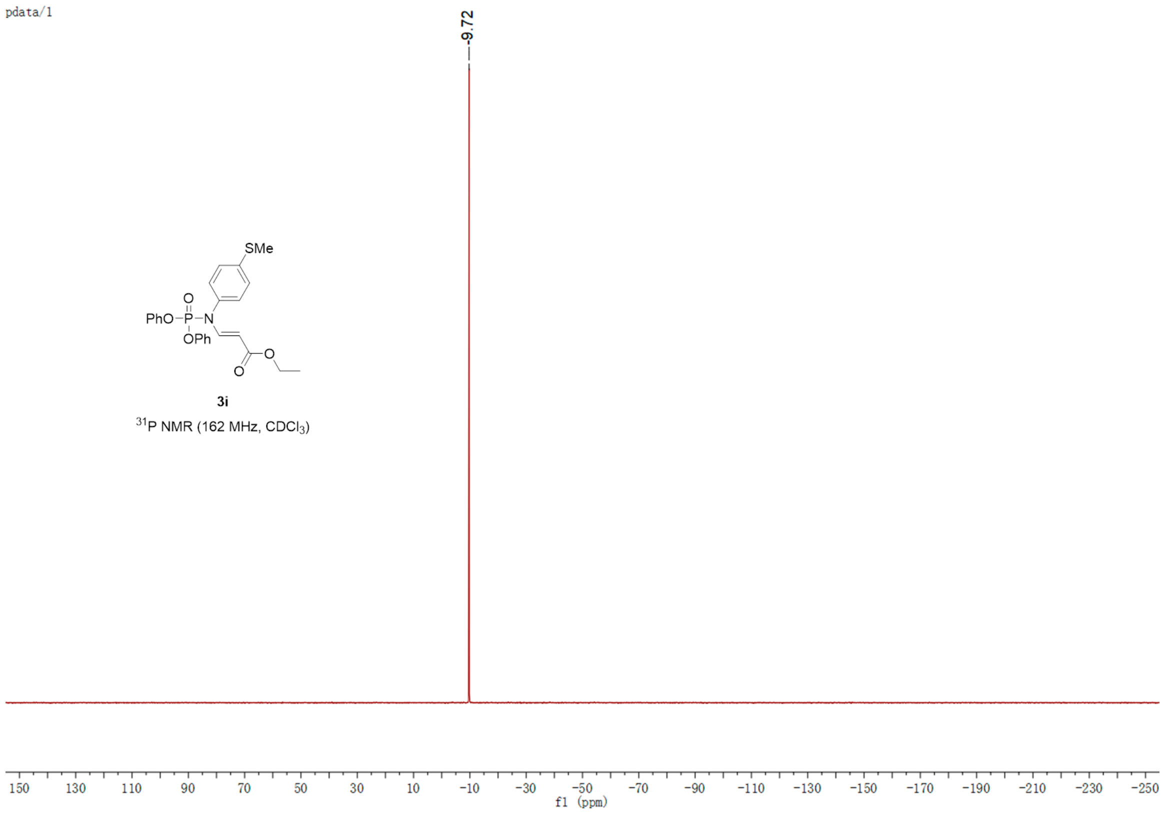
pdata/1
-9.72
SMe
PhO
OPh
3i
31P NMR (162 MHz, CDCl3)
f1 (ppm)

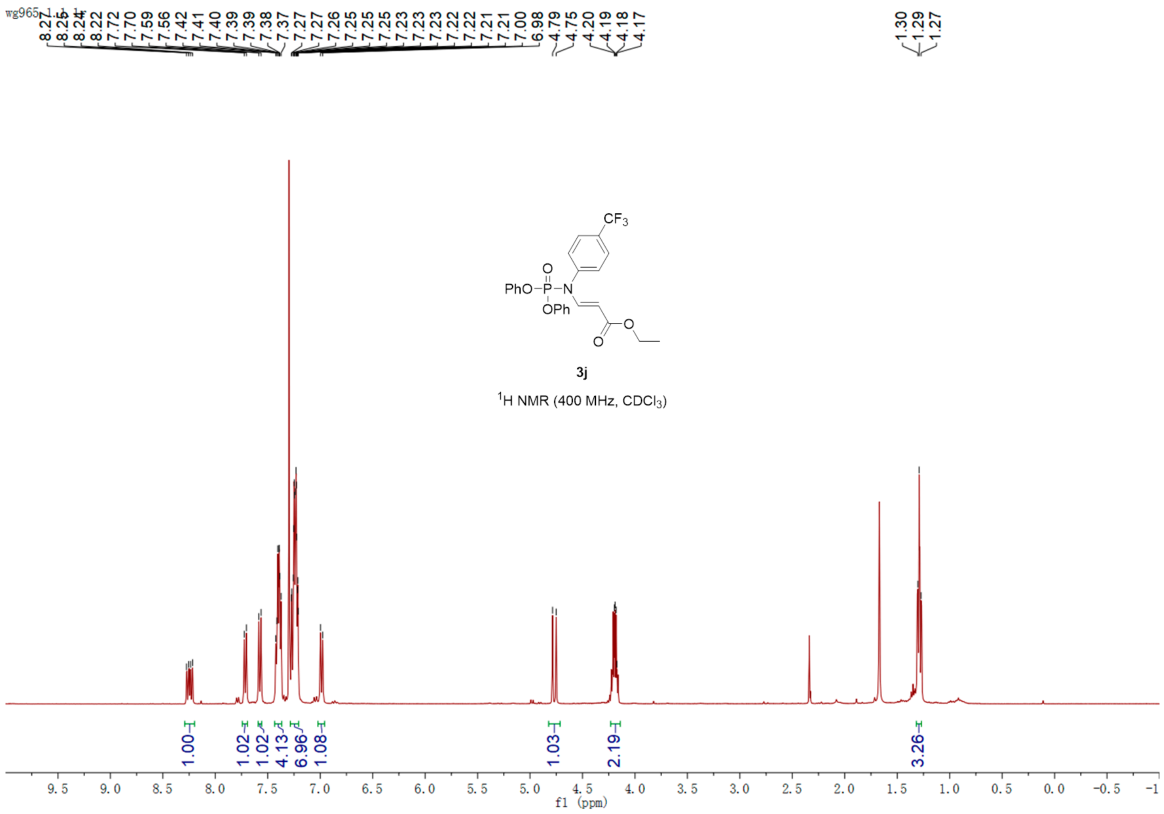
8.27
8.25
8.24
8.22
7.72
7.70
7.59
7.56
7.42
7.41
7.40
7.39
7.39
7.38
7.37
7.27
7.27
7.26
7.25
7.25
7.25
7.23
7.23
7.23
7.22
7.22
7.21
7.21
7.00
6.98
4.79
4.75
4.20
4.19
4.18
4.17
1.30
1.29
1.27
CF3
PhO
OPh
3j
1H NMR (400 MHz, CDCl3)
1.00
1.02
1.02
4.13
6.96
1.08
1.03
2.19
3.26
f1 (ppm)

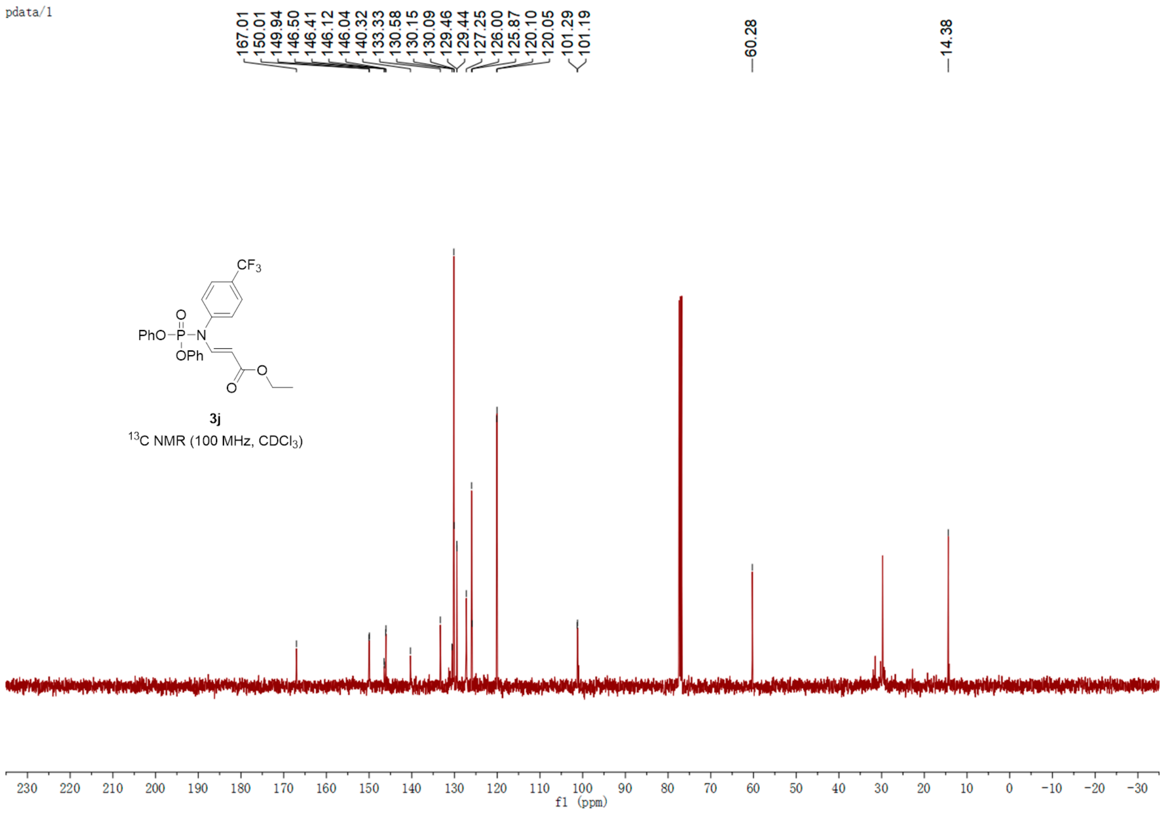
pdata/1
167.01
150.01
149.94
146.50
146.41
146.12
146.04
140.32
133.33
130.58
130.15
130.09
129.46
129.44
127.25
126.00
125.87
120.10
120.05
101.29
101.19
60.28
14.38
CF3
PhO
P
N
O
OPh
O
O
3j
13C NMR (100 MHz, CDCl3)
230 220 210 200 190 180 170 160 150 140 130 120 110 100 90 80 70 60 50 40 30 20 10 0 -10 -20 -30
f1 (ppm)

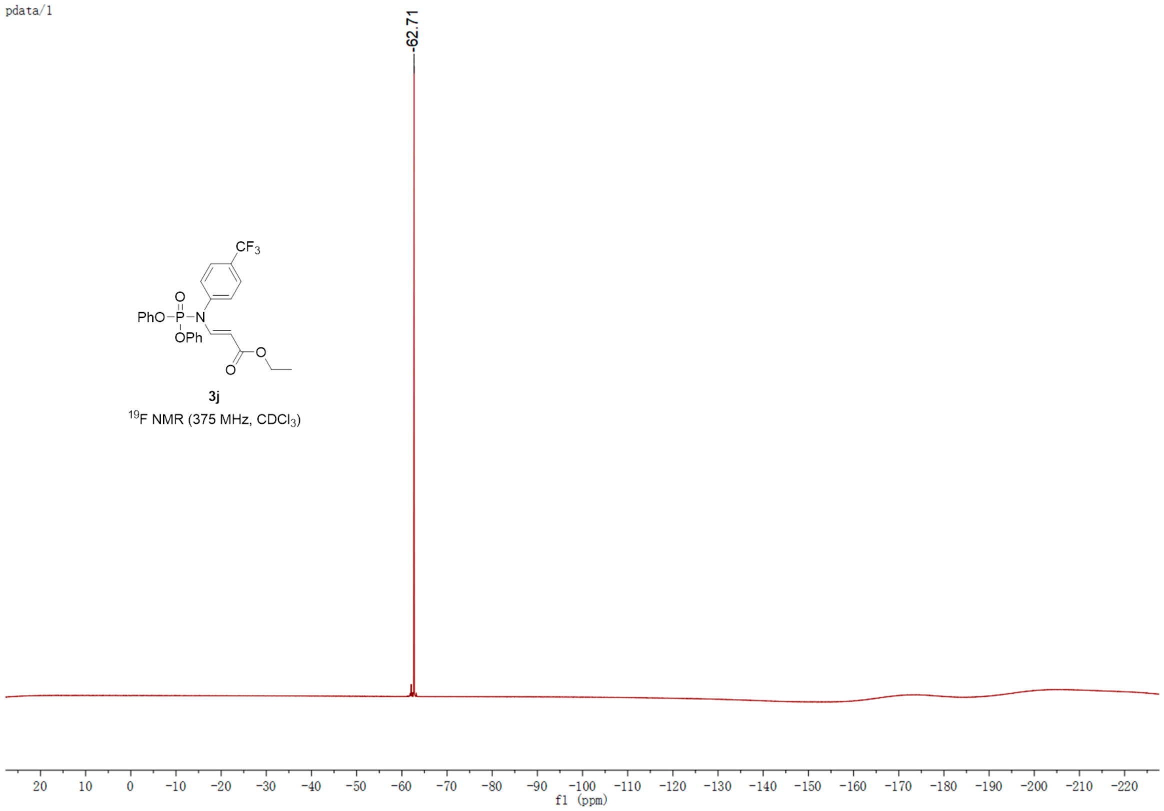
pdata/1
-62.71
CF3
PhO
P
N
O
OPh
O
O
3j
19F NMR (375 MHz, CDCl3)
20 10 0 -10 -20 -30 -40 -50 -60 -70 -80 -90 -100 -110 -120 -130 -140 -150 -160 -170 -180 -190 -200 -210 -220
f1 (ppm)

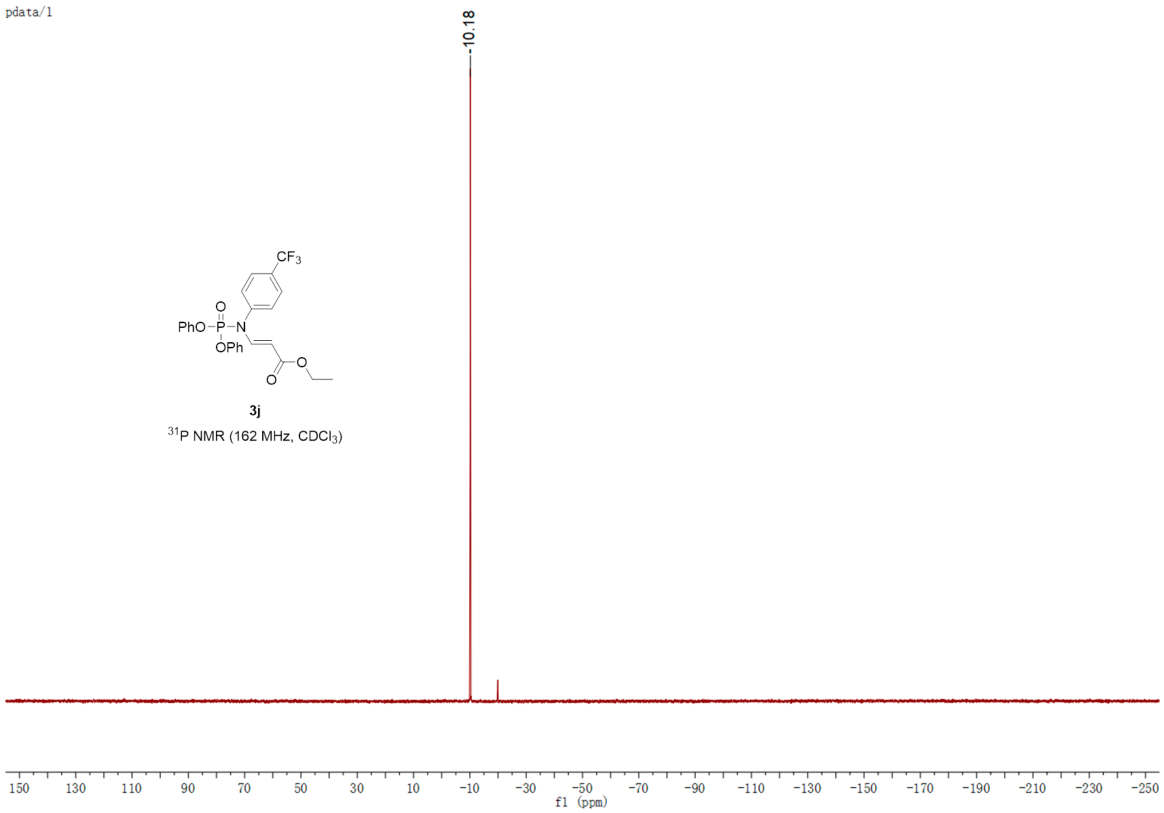
pdata/1
-10.18
CF3
PhO-P-N
O
OPh
O
3j
31P NMR (162 MHz, CDCl3)
150 130 110 90 70 50 30 10 -10 -30 -50 -70 -90 -110 -130 -150 -170 -190 -210 -230 -250
f1 (ppm)

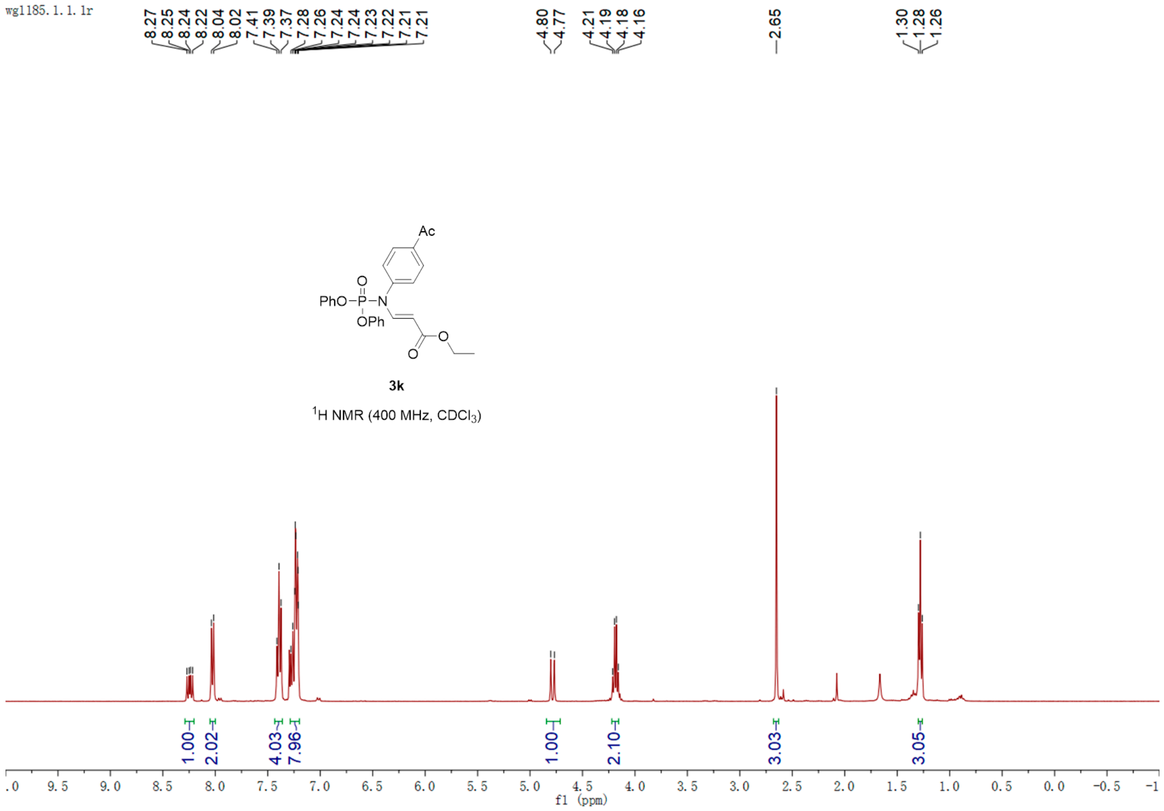
wg1185.1.1.1r
8.27 8.25 8.24 8.22 8.04 8.02 7.41 7.39 7.37 7.28 7.26 7.24 7.24 7.23 7.22 7.21 7.21
4.80 4.77
4.21 4.19 4.18 4.16
2.65
1.30 1.28 1.26
Ac
PhO-P-N
O
OPh
O
3k
1H NMR (400 MHz, CDCl3)
1.00 2.02 4.03 7.96 1.00 2.10 3.03 3.05
9.5 9.0 8.5 8.0 7.5 7.0 6.5 6.0 5.5 5.0 4.5 4.0 3.5 3.0 2.5 2.0 1.5 1.0 0.5 0.0 -0.5
f1 (ppm)

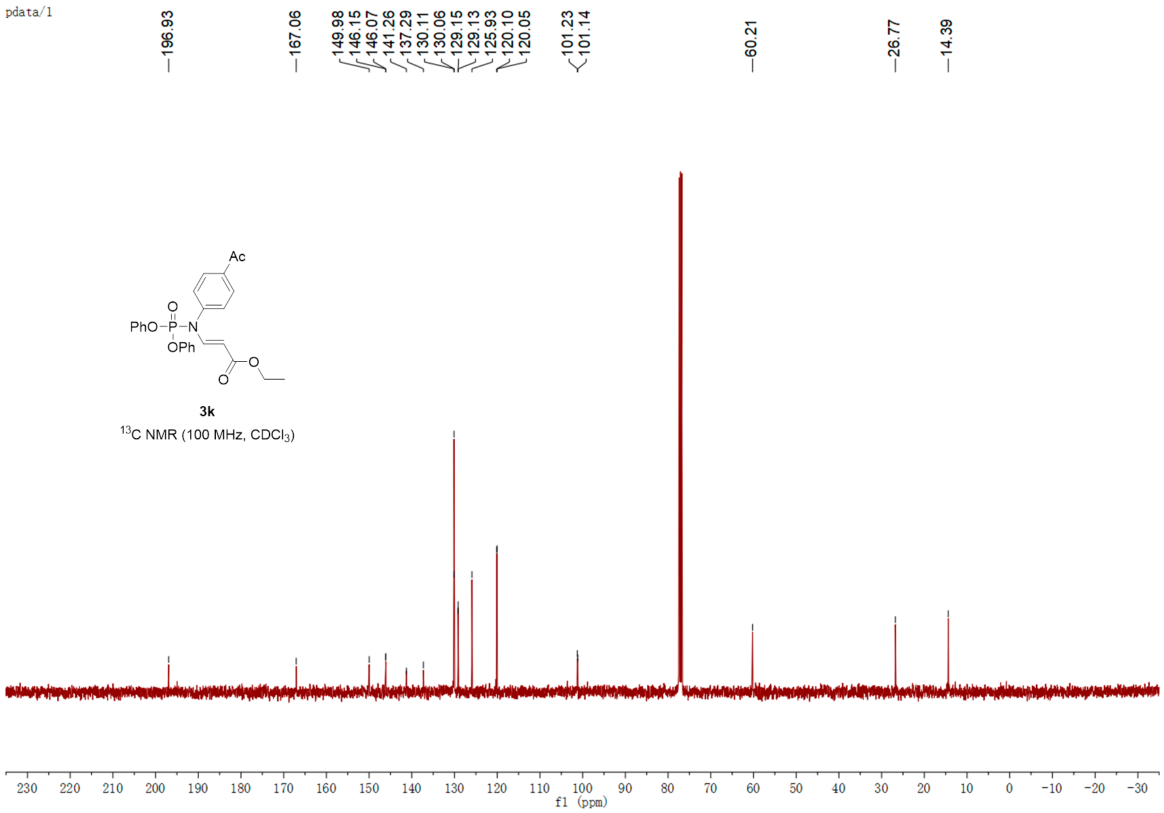
pdata/1
196.93
167.06
149.98
146.15
146.07
141.26
137.29
130.11
130.06
129.15
129.13
125.93
120.10
120.05
101.23
101.14
60.21
26.77
14.39
Ac
PhO
OPh
3k
^{13}C NMR (100 MHz, CDCl$_3$)
f1 (ppm)

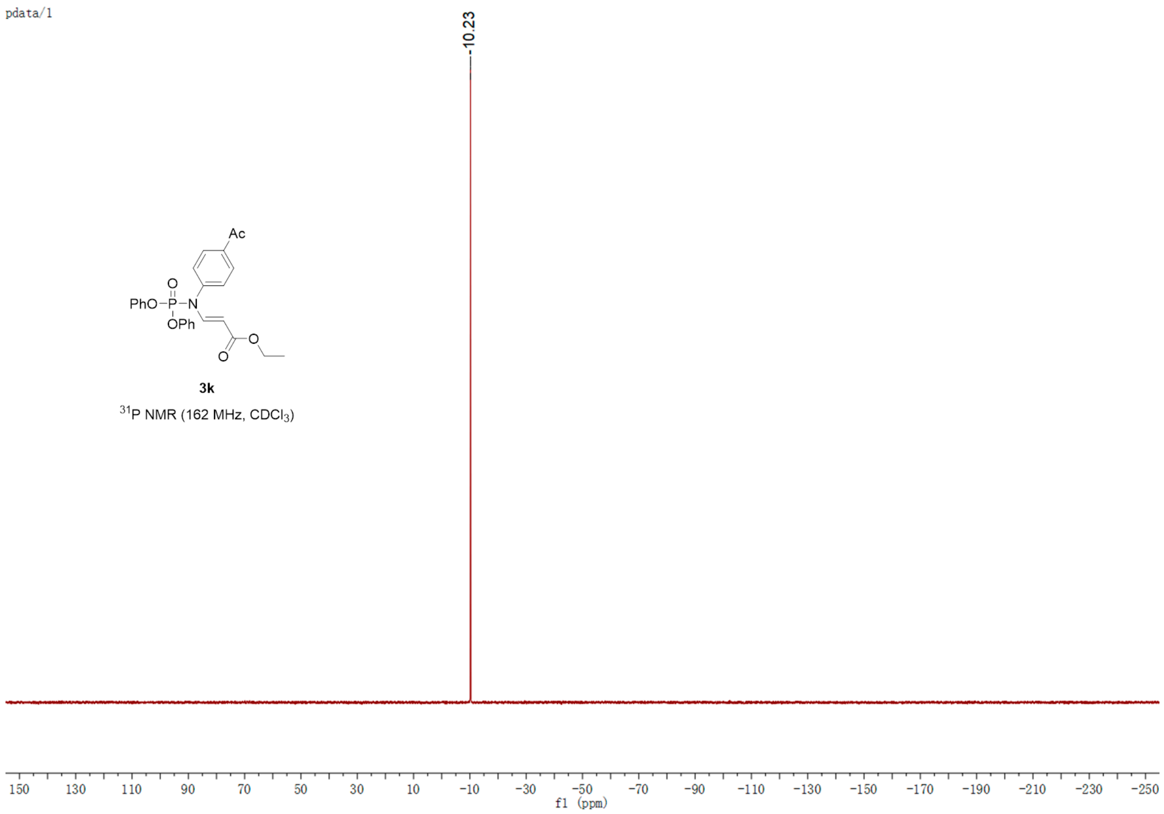
pdata/1
-10.23
Ac
PhO
OPh
3k
^{31}P NMR (162 MHz, CDCl$_3$)
f1 (ppm)

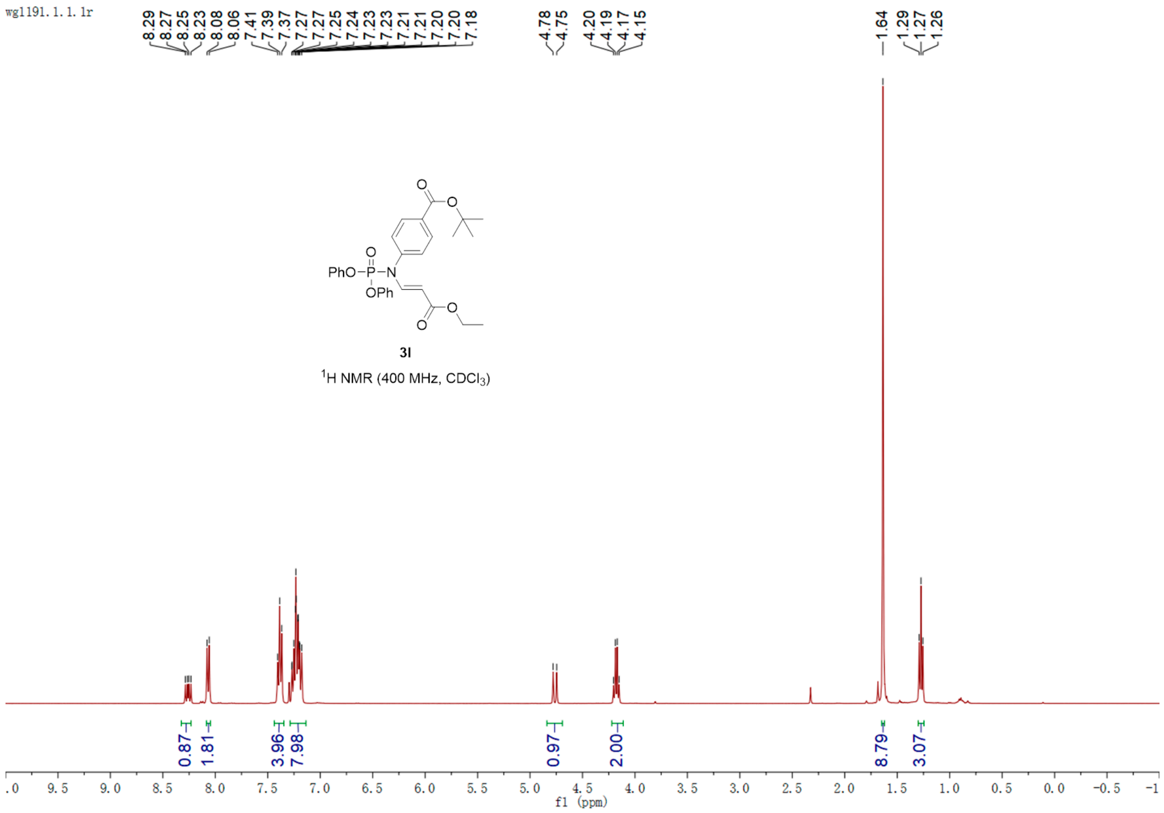

wg1191.1.1.1r
8.29 8.27 8.25 8.23 8.08 8.06 7.41 7.39 7.37 7.27 7.27 7.25 7.24 7.23 7.23 7.21 7.21 7.20 7.20 7.18
4.78 4.75
4.20 4.19 4.17 4.15
1.64
1.29 1.27 1.26
PhO
P
N
O
OPh
3l
1H NMR (400 MHz, CDCl3)
0.87 1.81 3.96 7.98 0.97 2.00 8.79 3.07
f1 (ppm)

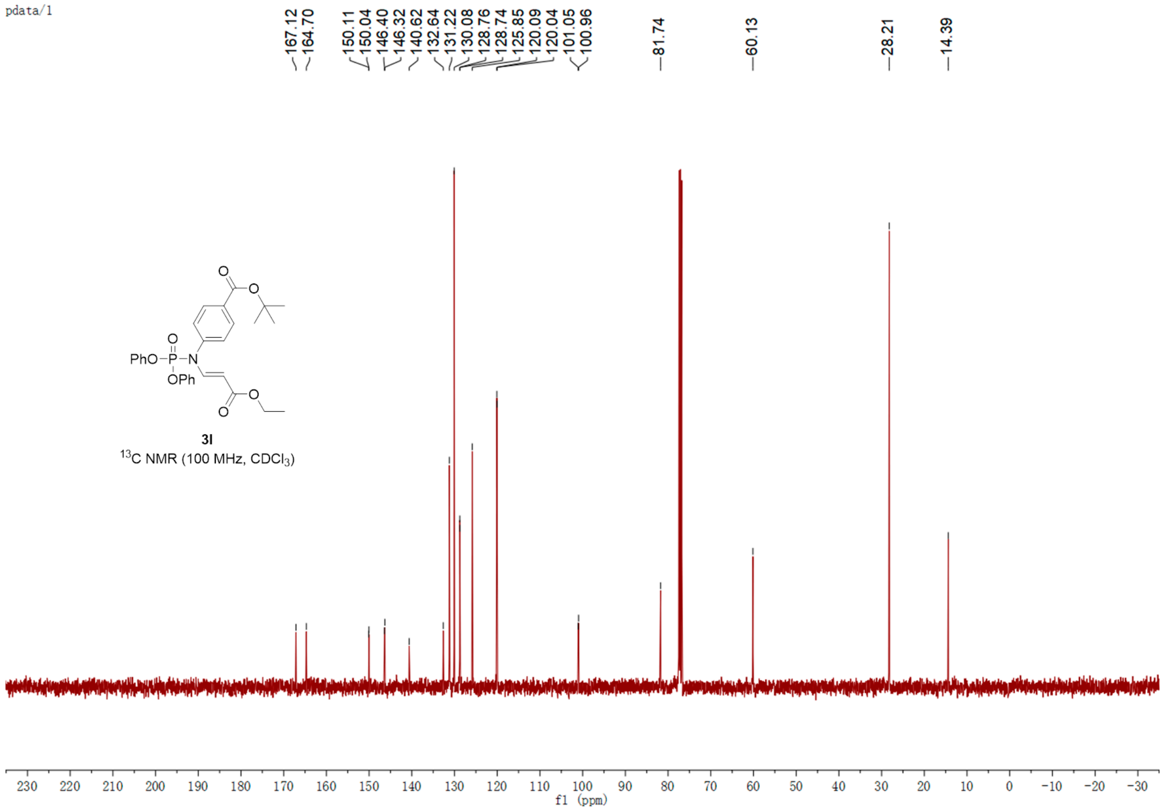

pdata/1
167.12 164.70
150.11 150.04 146.40 146.32 140.62 132.64 131.22 130.08 128.76 128.74 125.85 120.09 120.04 101.05 100.96
81.74
60.13
28.21
14.39
PhO
P
N
O
OPh
3l
13C NMR (100 MHz, CDCl3)
f1 (ppm)

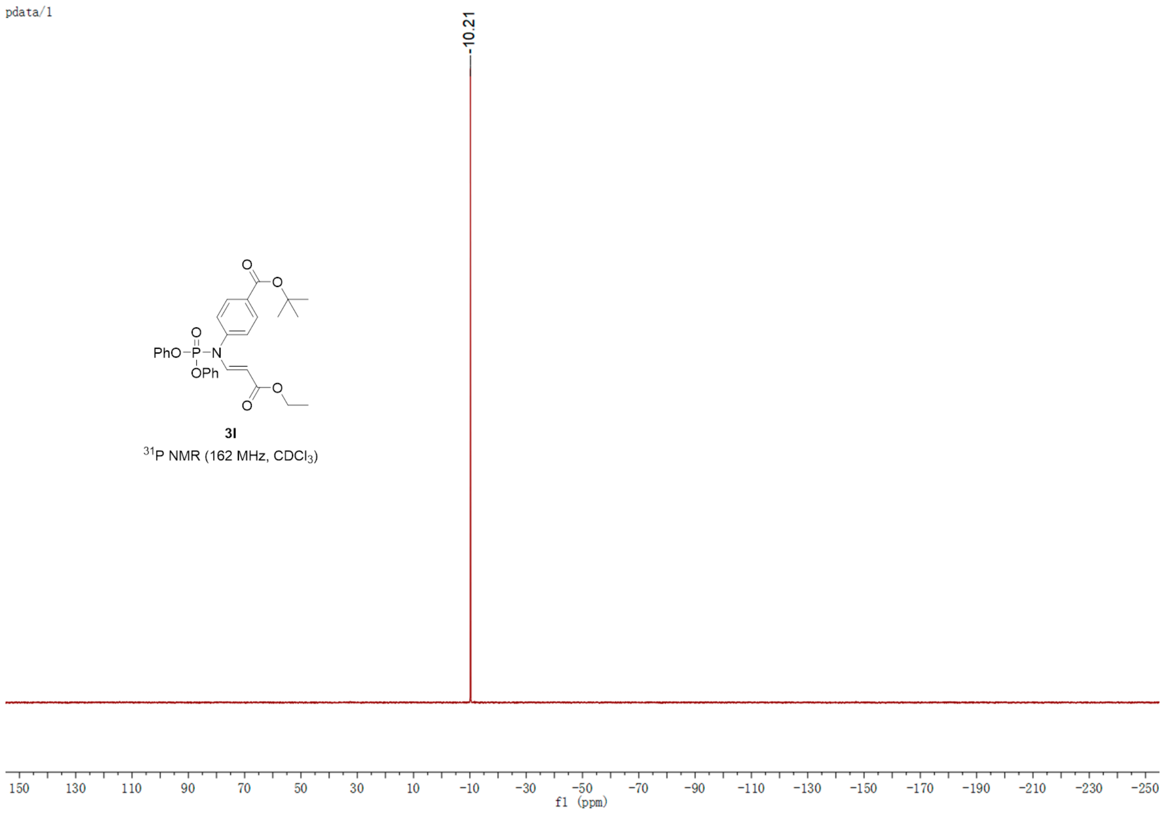
pdata/1
-10.21
PhO
OPh
3l
31P NMR (162 MHz, CDCl3)
f1 (ppm)

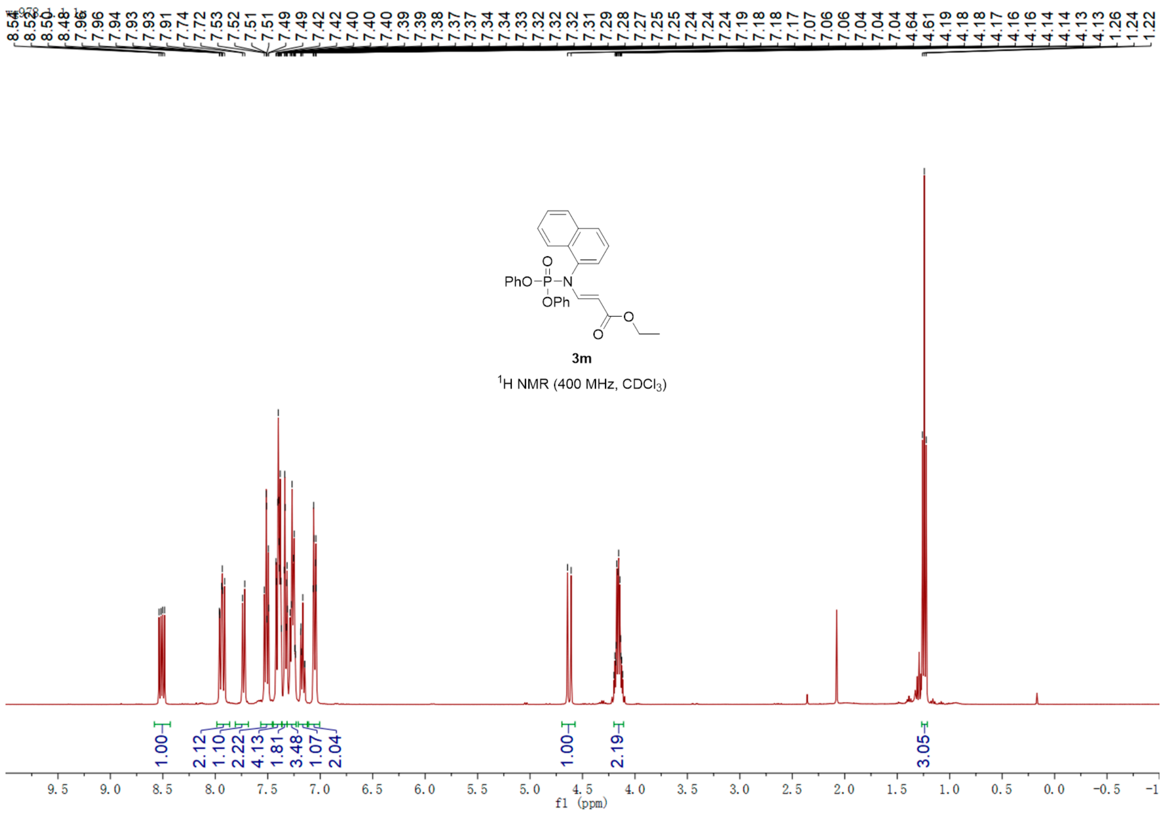
PhO
OPh
3m
1H NMR (400 MHz, CDCl3)
1.00
2.12
1.10
2.22
4.13
1.81
3.48
1.07
2.04
1.00
2.19
3.05
f1 (ppm)

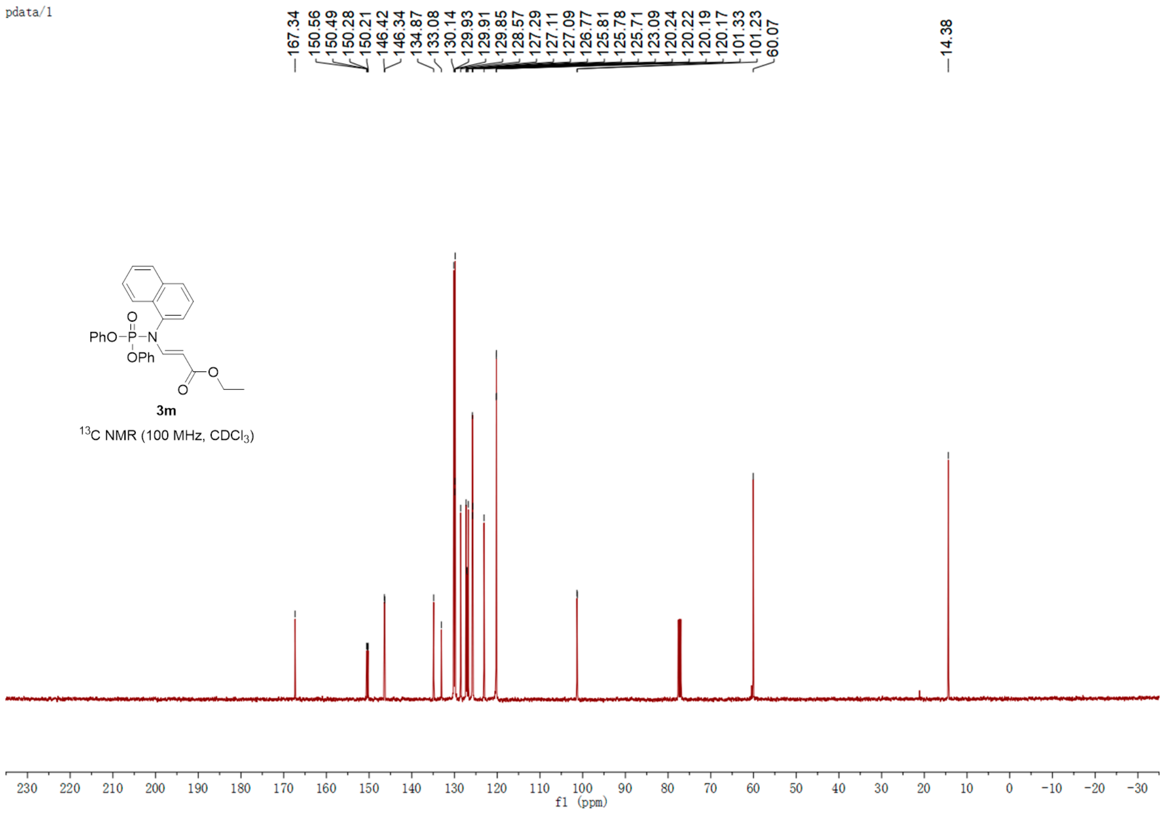
pdata/1
167.34
150.56
150.49
150.28
150.21
146.42
146.34
134.87
133.08
130.14
129.93
129.91
129.85
128.57
127.29
127.11
127.09
126.77
125.81
125.78
125.71
123.09
120.24
120.22
120.19
120.17
101.33
101.23
60.07
14.38
PhO
O
P
N
OPh
O
O
3m
13C NMR (100 MHz, CDCl3)
f1 (ppm)

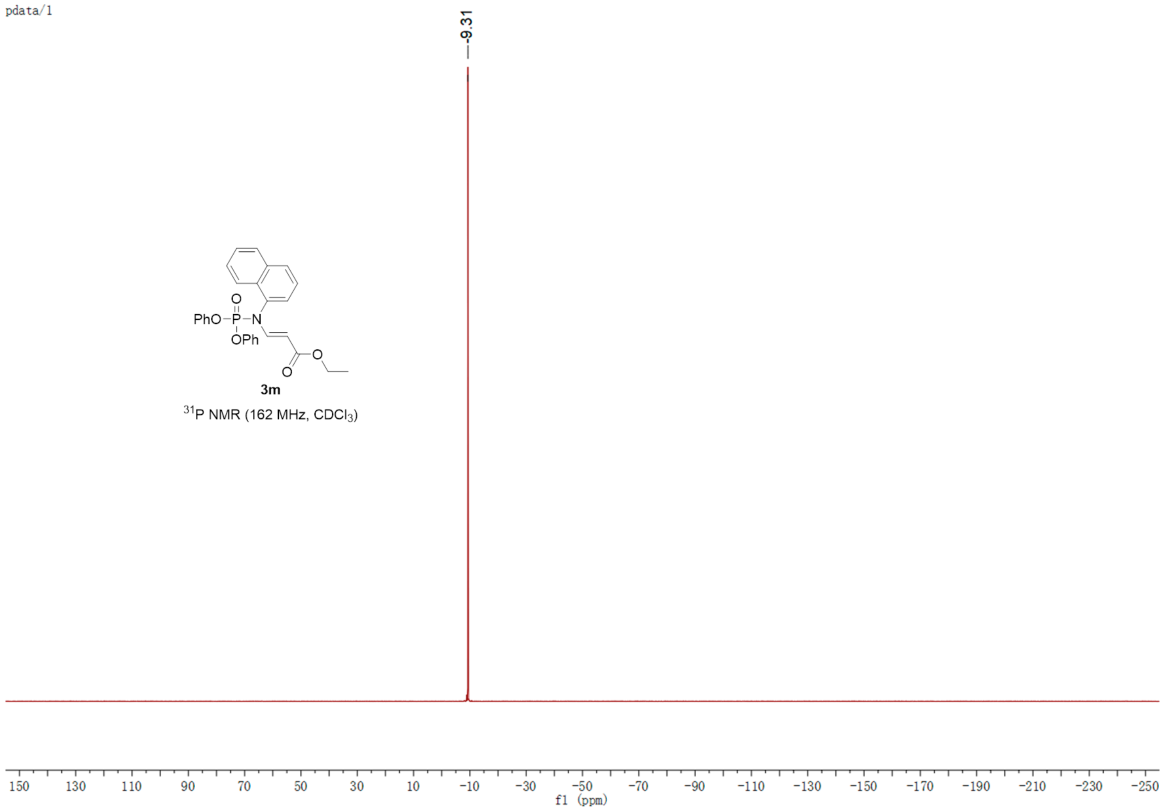
pdata/1
-9.31
PhO
O
P
N
OPh
O
O
3m
31P NMR (162 MHz, CDCl3)
f1 (ppm)

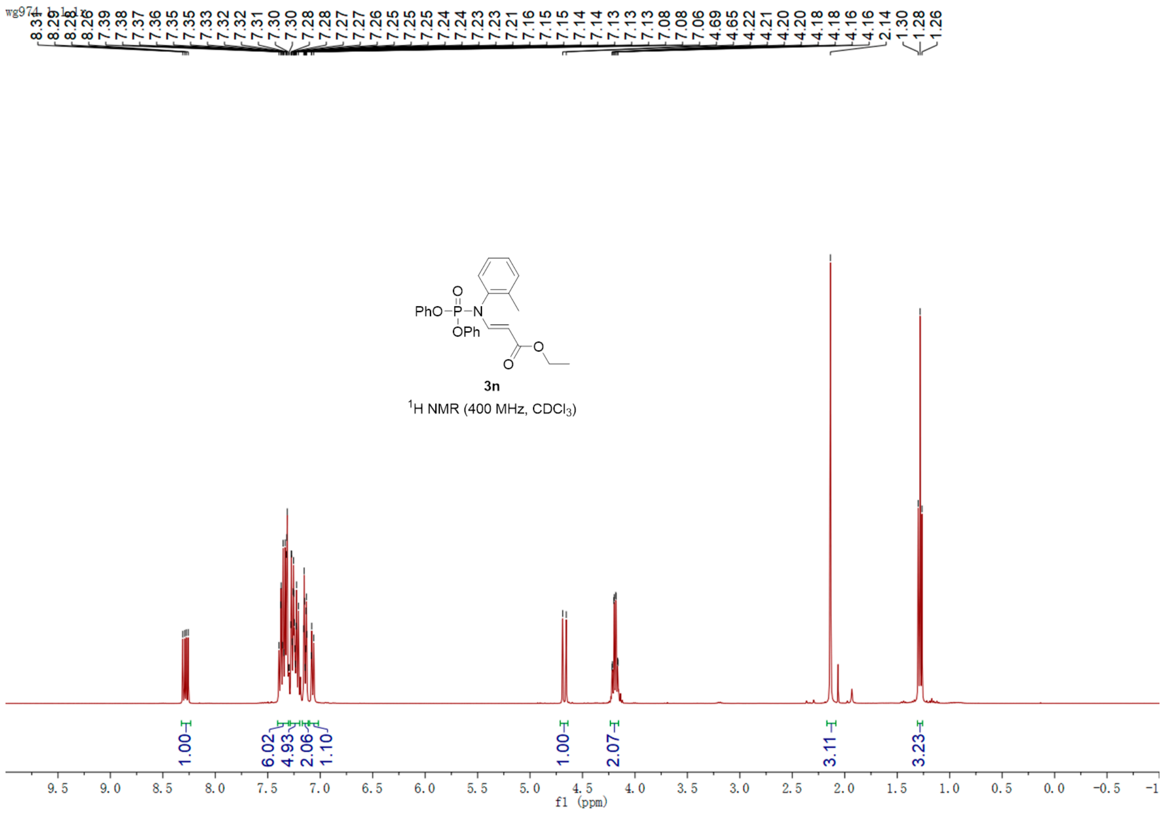
3n
^{1}H NMR (400 MHz, CDCl$_3$)

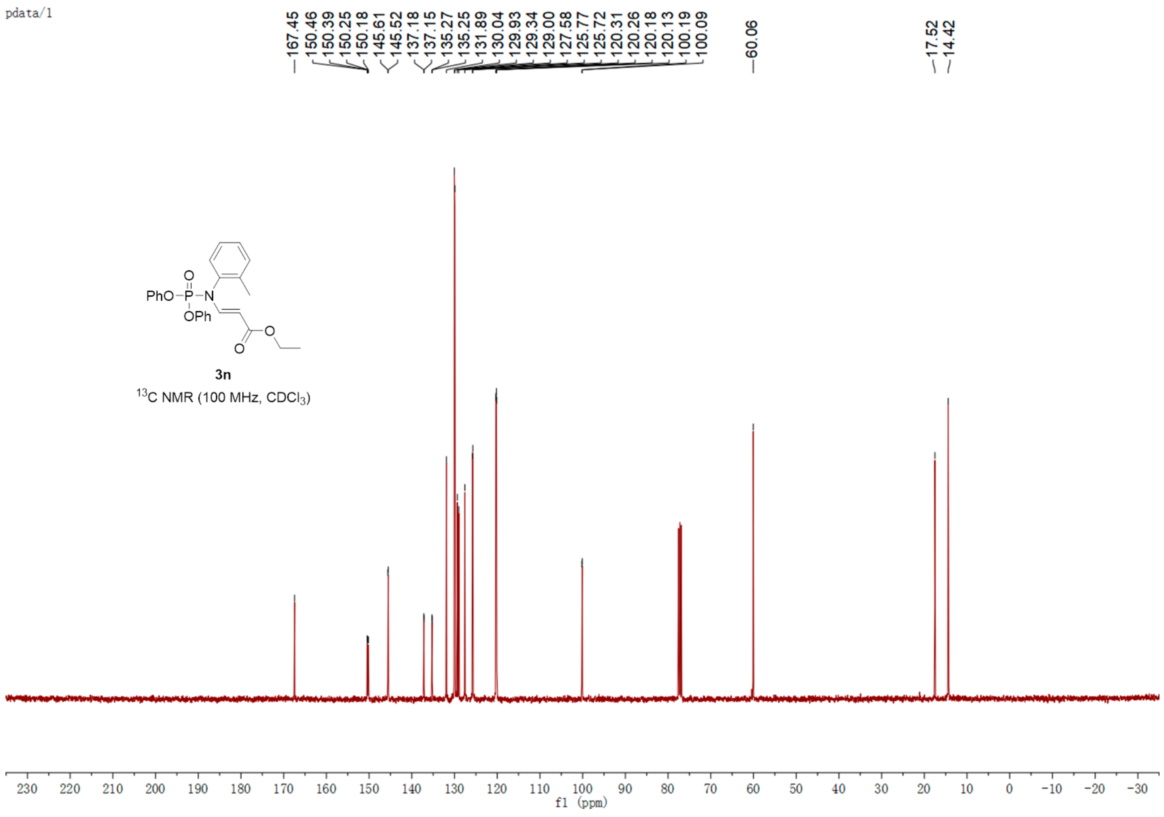
3n
^{13}C NMR (100 MHz, CDCl$_3$)

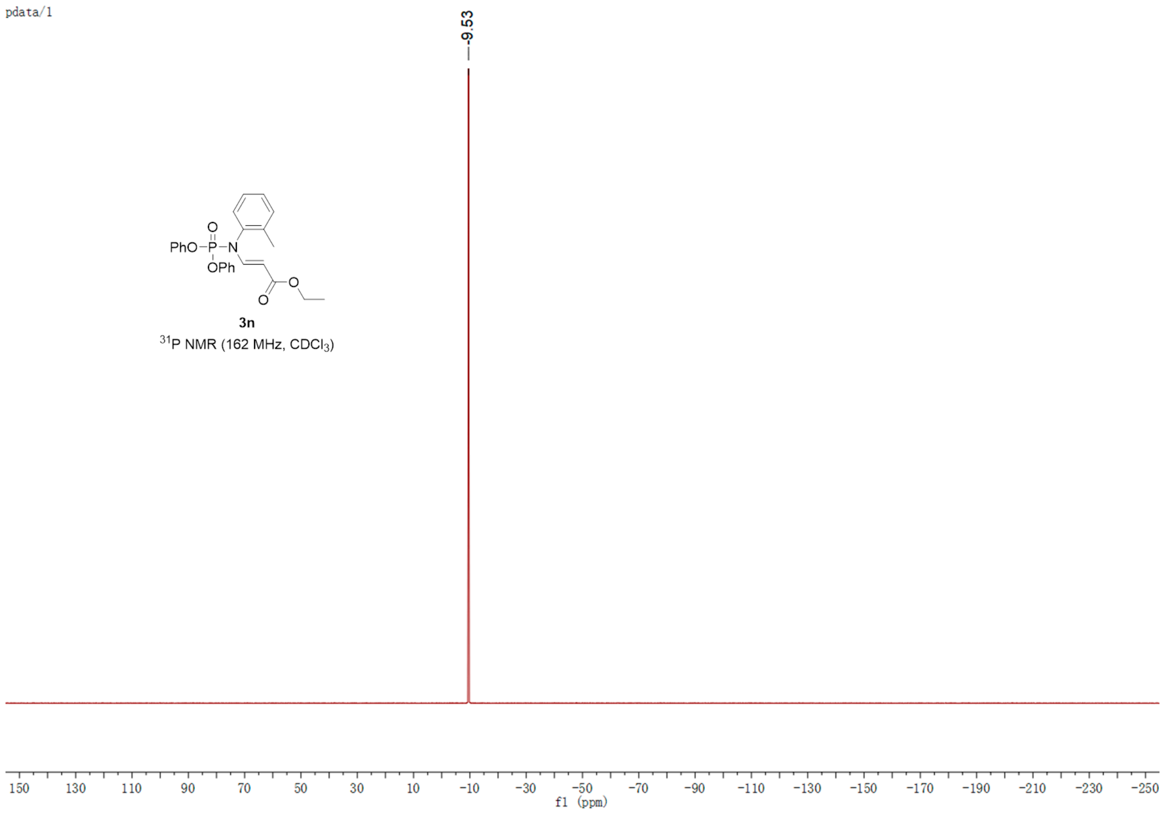
pdata/1
-9.53
PhO
O
P
N
OPh
O
O
3n
31P NMR (162 MHz, CDCl3)
150 130 110 90 70 50 30 10 -10 -30 -50 -70 -90 -110 -130 -150 -170 -190 -210 -230 -250
f1 (ppm)

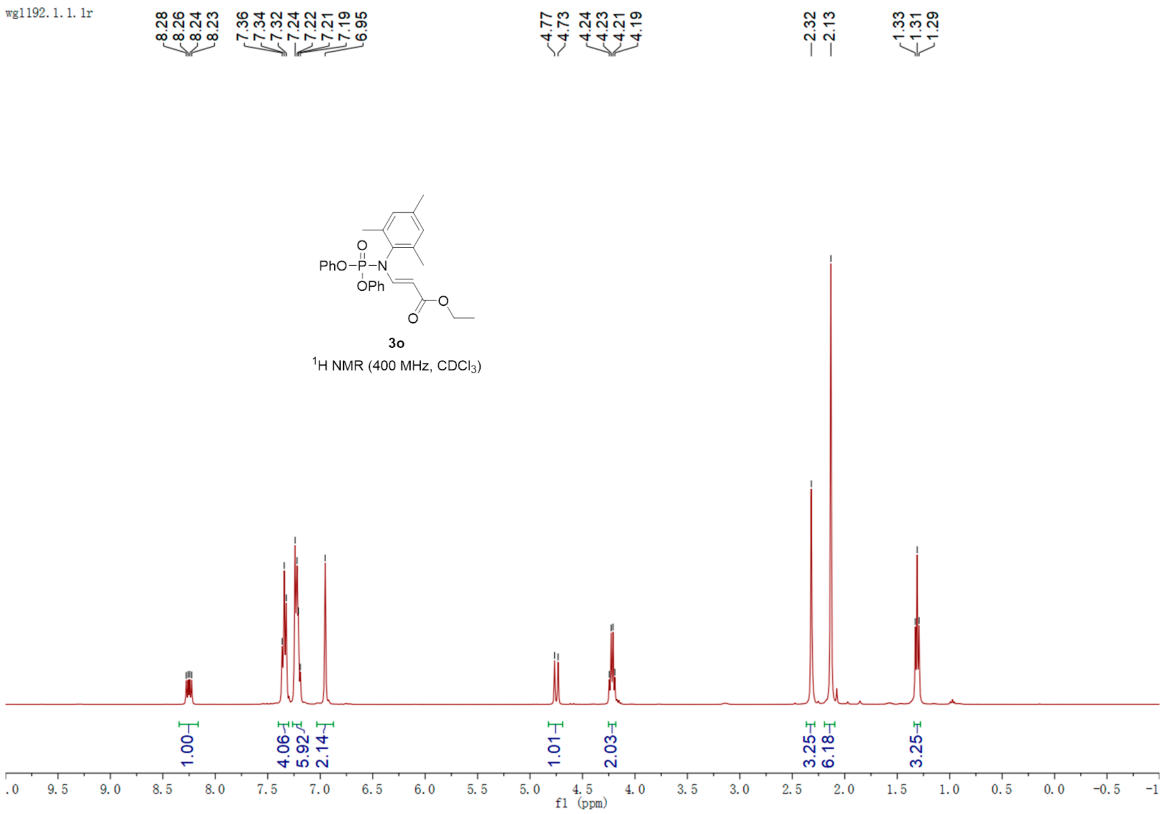
wg1192.1.1.1r
8.28 8.26 8.24 8.23 7.36 7.34 7.32 7.24 7.22 7.21 7.19 6.95
4.77 4.73 4.24 4.23 4.21 4.19
2.32 2.13
1.33 1.31 1.29
PhO
O
P
N
OPh
O
O
3o
1H NMR (400 MHz, CDCl3)
1.00 4.06 5.92 2.14 1.01 2.03 3.25 6.18 3.25
.0 9.5 9.0 8.5 8.0 7.5 7.0 6.5 6.0 5.5 5.0 4.5 4.0 3.5 3.0 2.5 2.0 1.5 1.0 0.5 0.0 -0.5 -1
f1 (ppm)

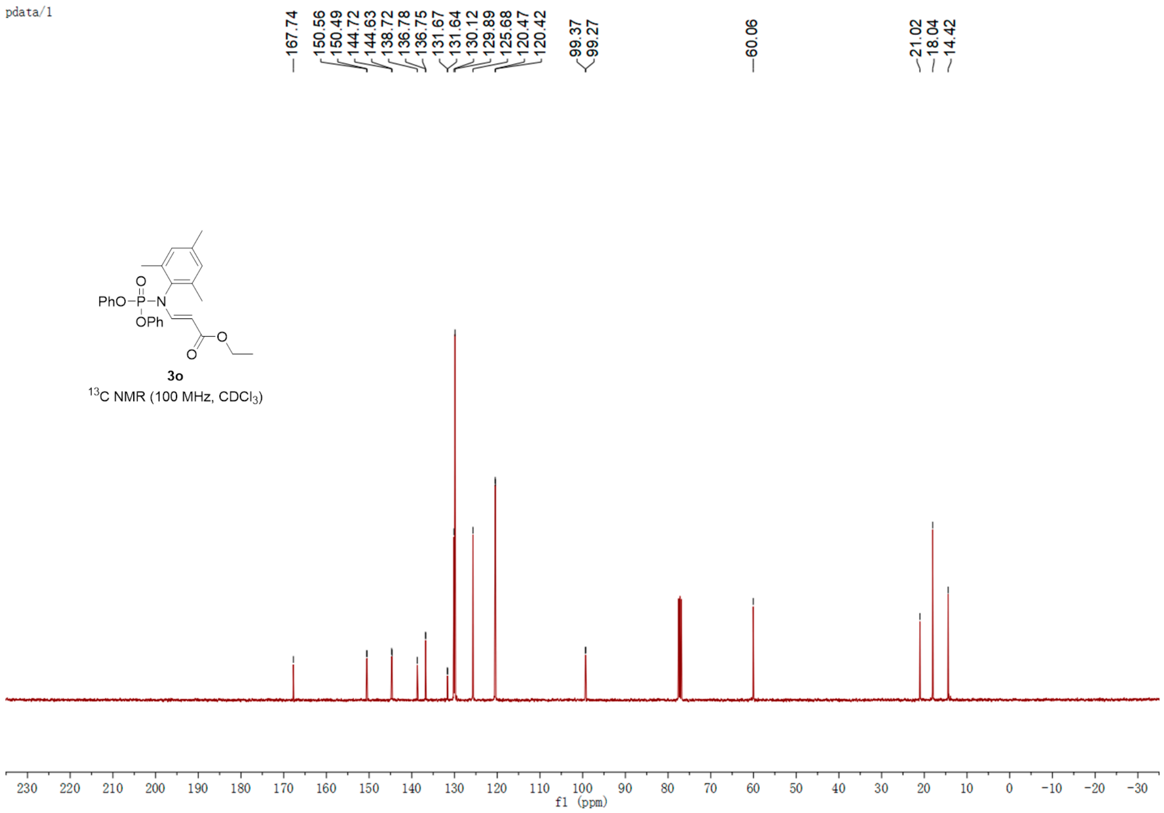
pdata/1
167.74
150.56
150.49
144.72
144.63
138.72
136.78
136.75
131.67
131.64
130.12
129.89
125.68
120.47
120.42
99.37
99.27
60.06
21.02
18.04
14.42
PhO
P
N
O
OPh
O
O
3o
^{13}C NMR (100 MHz, CDCl$_3$)
f1 (ppm)

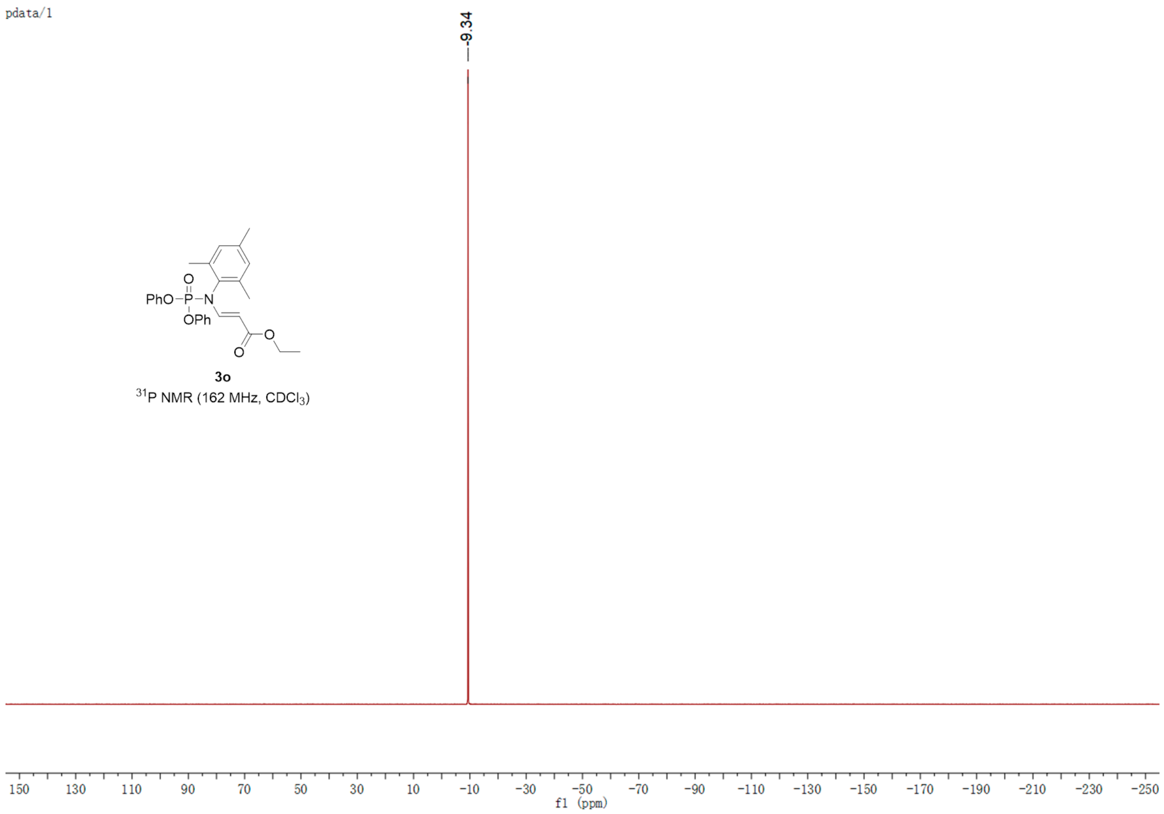
pdata/1
-9.34
PhO
P
N
O
OPh
O
O
3o
^{31}P NMR (162 MHz, CDCl$_3$)
f1 (ppm)

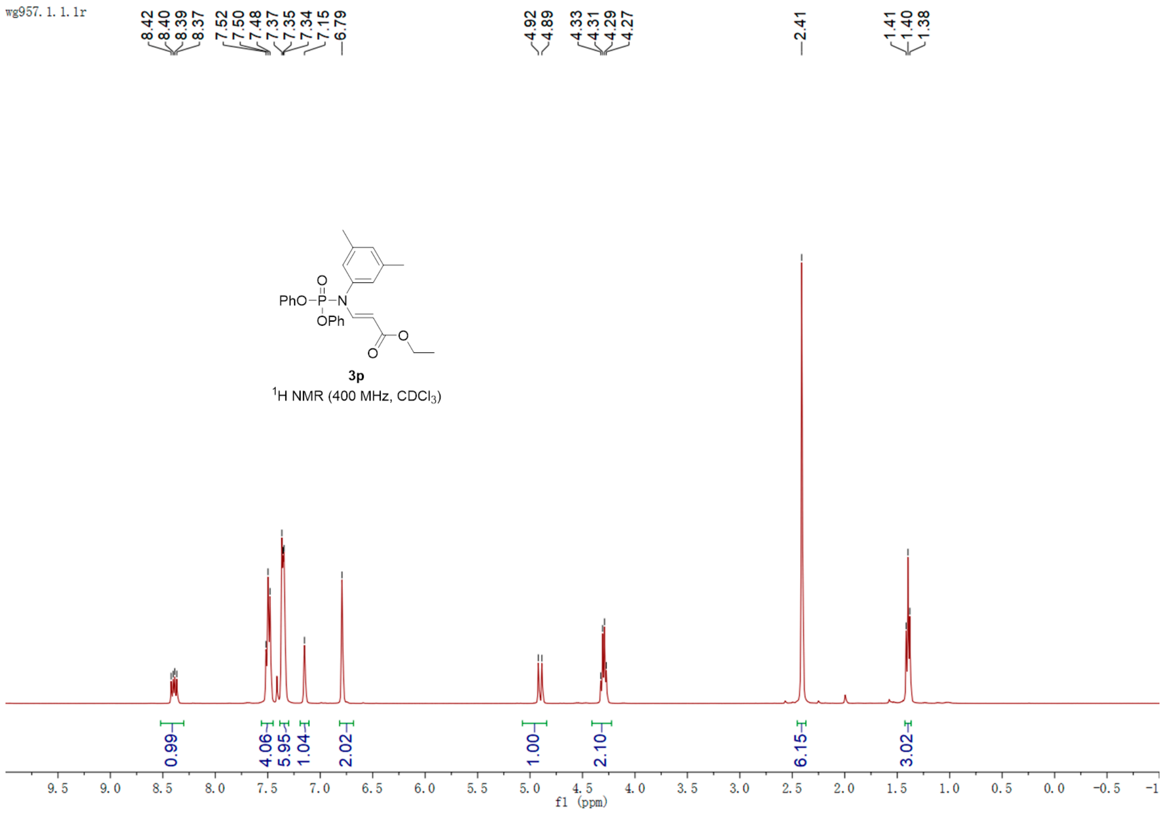
wg957.1.1.1r
8.42
8.40
8.39
8.37
7.52
7.50
7.48
7.37
7.35
7.34
7.15
6.79
4.92
4.89
4.33
4.31
4.29
4.27
2.41
1.41
1.40
1.38
PhO
P
N
O
OPh
O
O
3p
1H NMR (400 MHz, CDCl3)
0.99
4.06
5.95
1.04
2.02
1.00
2.10
6.15
3.02
f1 (ppm)

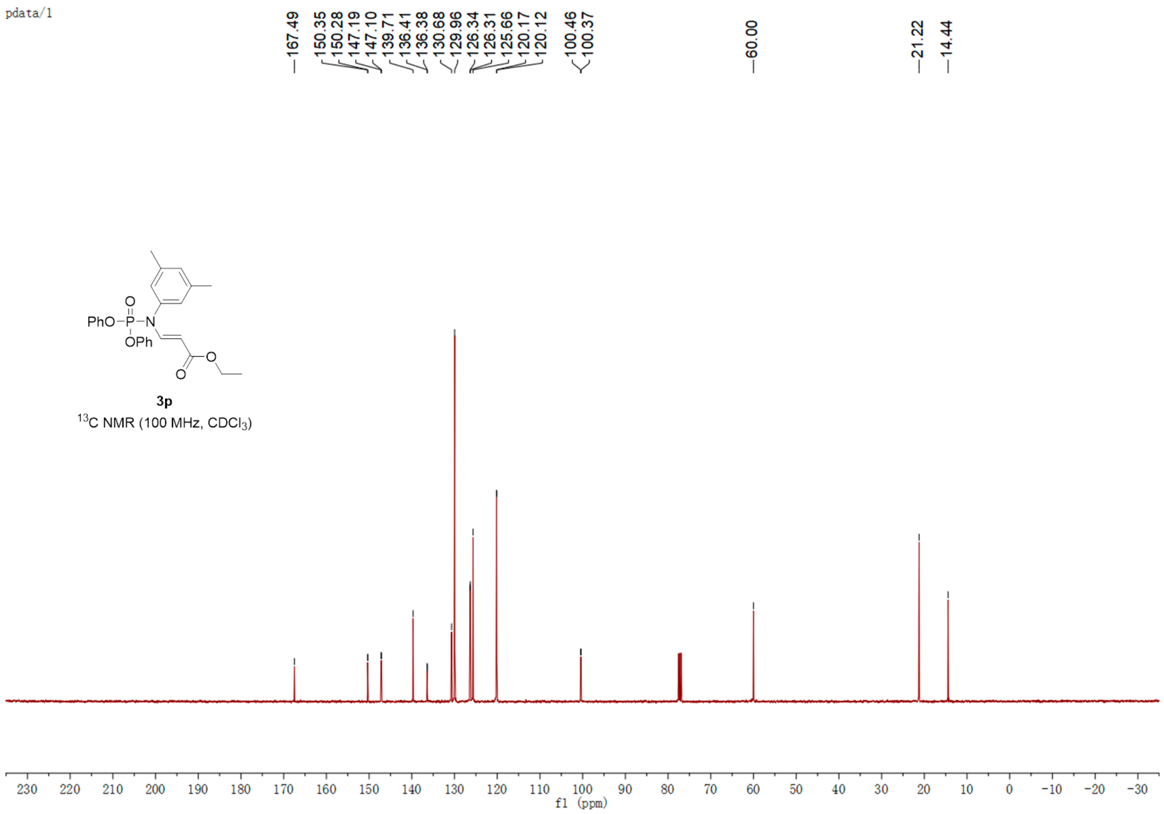
pdata/1
167.49
150.35
150.28
147.19
147.10
139.71
136.41
136.38
130.68
129.96
126.34
126.31
125.66
120.17
120.12
100.46
100.37
60.00
21.22
14.44
PhO
P
N
O
OPh
O
O
3p
13C NMR (100 MHz, CDCl3)
f1 (ppm)

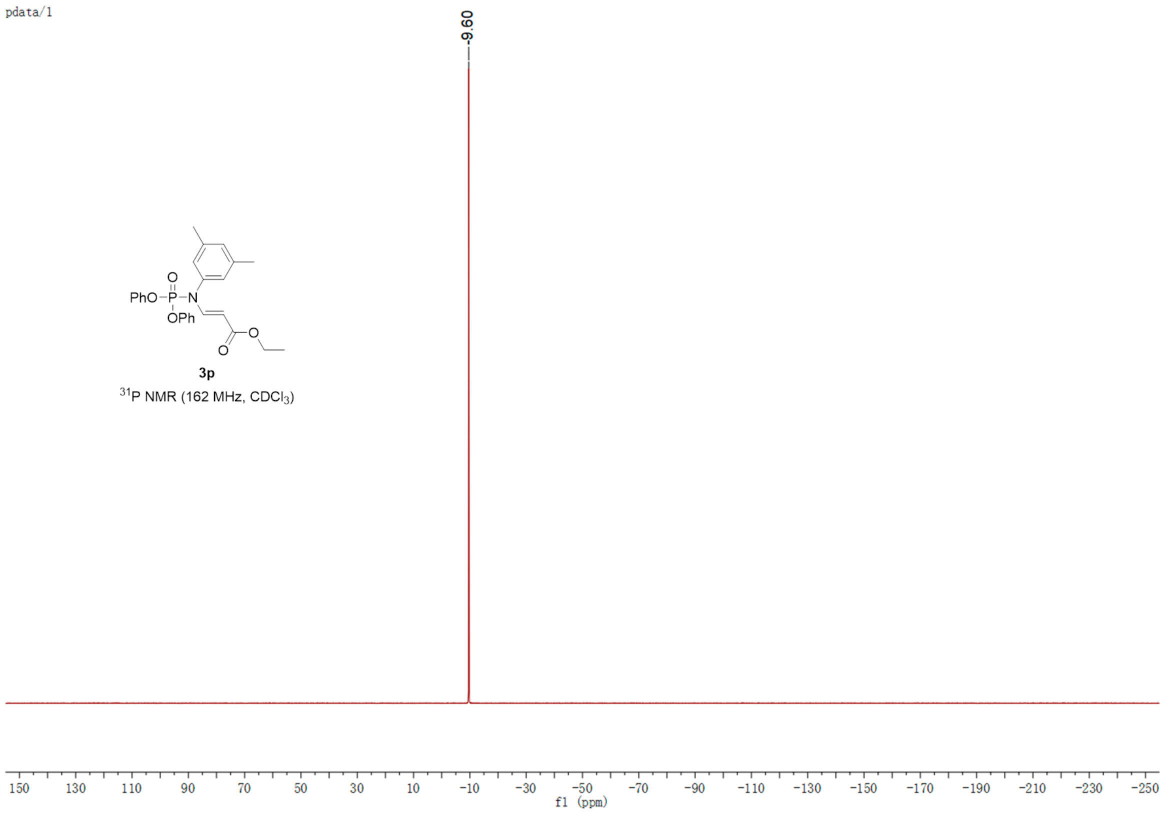
pdata/1
-9.60
PhO
OPh
3p
31P NMR (162 MHz, CDCl3)
f1 (ppm)

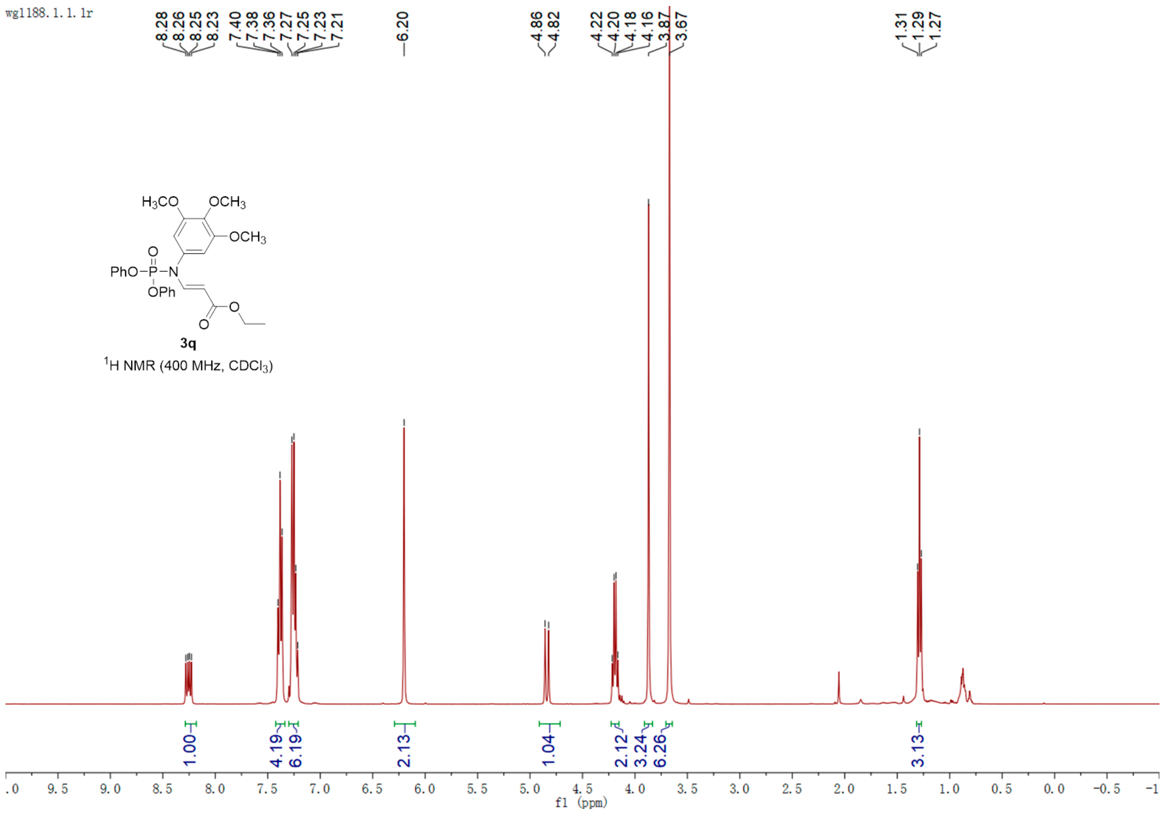
wg1188.1.1.1r
8.28
8.26
8.25
8.23
7.40
7.38
7.36
7.27
7.25
7.23
7.21
6.20
4.86
4.82
4.22
4.20
4.18
4.16
3.87
3.67
1.31
1.29
1.27
H3CO
OCH3
OCH3
PhO
OPh
3q
1H NMR (400 MHz, CDCl3)
1.00
4.19
6.19
2.13
1.04
2.12
3.24
6.26
3.13
f1 (ppm)

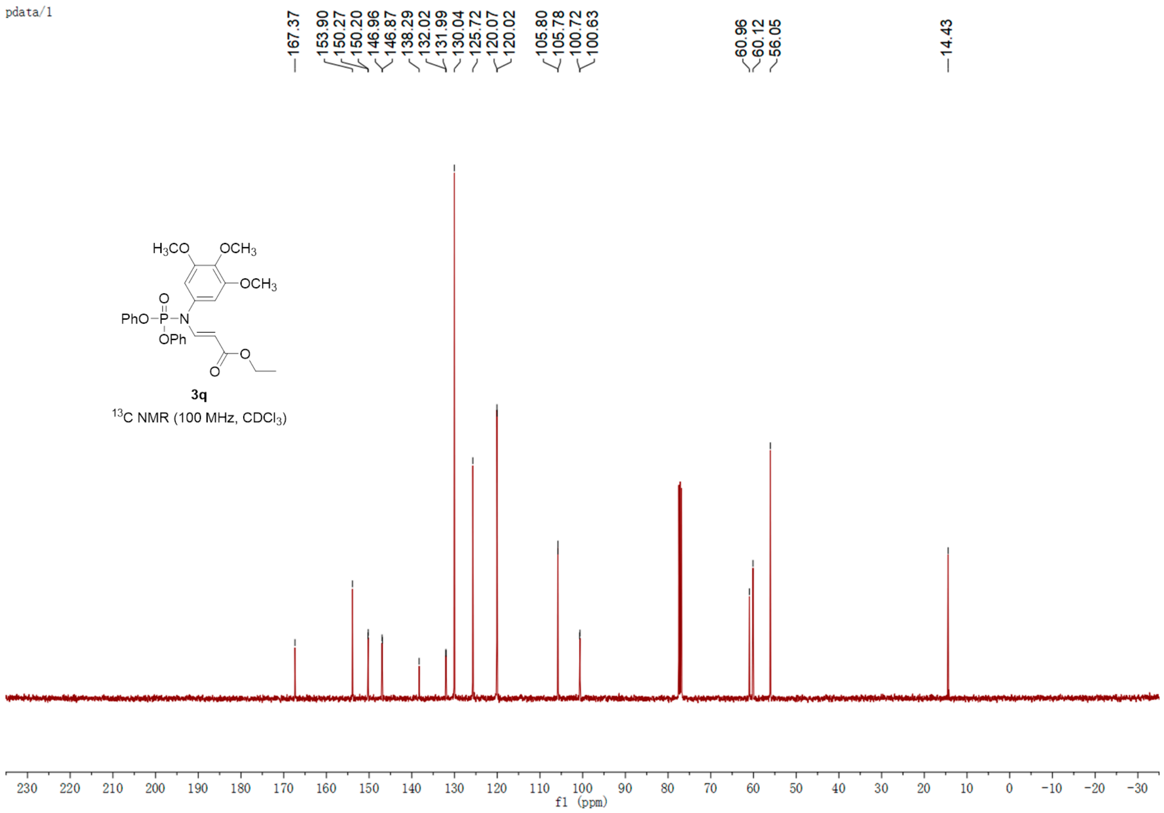
pdata/1
167.37
153.90
150.27
150.20
146.96
146.87
138.29
132.02
131.99
130.04
125.72
120.07
120.02
105.80
105.78
100.72
100.63
60.96
60.12
56.05
14.43
H3CO
OCH3
OCH3
O
PhO-P-N
OPh
O
O
3q
13C NMR (100 MHz, CDCl3)
f1 (ppm)

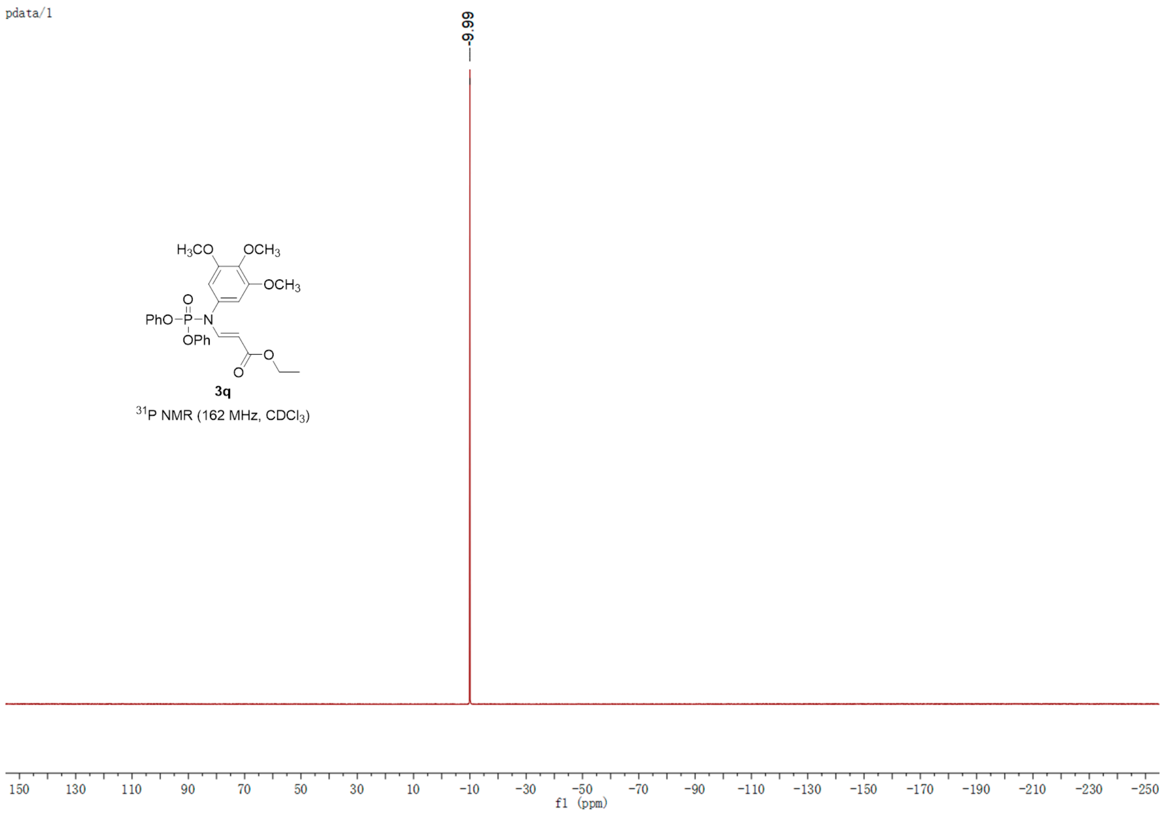
pdata/1
-9.99
H3CO
OCH3
OCH3
O
PhO-P-N
OPh
O
O
3q
31P NMR (162 MHz, CDCl3)
f1 (ppm)

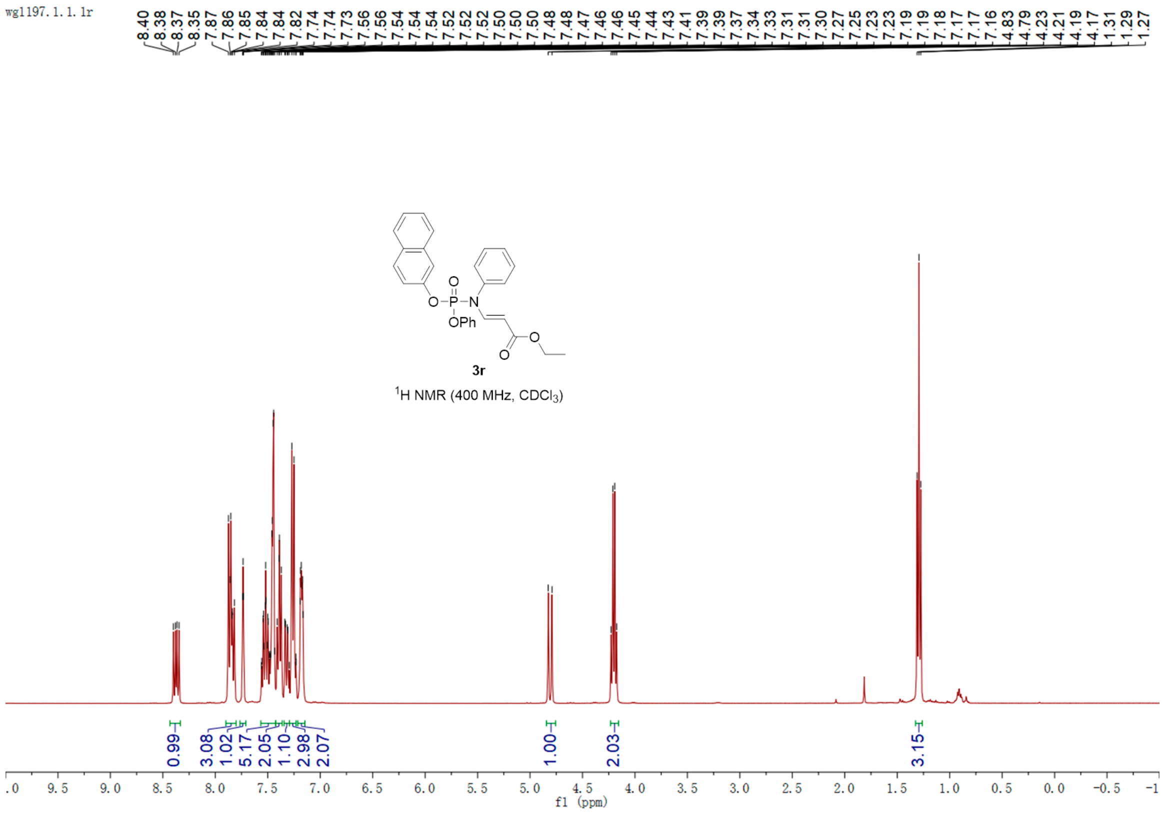
wg1197.1.1.1r
8.40 8.38 8.37 8.35 7.87 7.86 7.85 7.84 7.84 7.82 7.74 7.74 7.73 7.56 7.56 7.54 7.54 7.54 7.52 7.52 7.52 7.50 7.50 7.50 7.48 7.48 7.47 7.46 7.46 7.45 7.44 7.43 7.41 7.39 7.39 7.37 7.34 7.33 7.31 7.31 7.30 7.27 7.25 7.23 7.23 7.19 7.19 7.18 7.17 7.16 4.83 4.79 4.23 4.21 4.19 4.17 1.31 1.29 1.27
O
O
P
N
OPh
O
O
3r
1H NMR (400 MHz, CDCl3)
0.99 3.08 1.02 5.17 2.05 1.10 2.98 2.07 1.00 2.03 3.15
f1 (ppm)

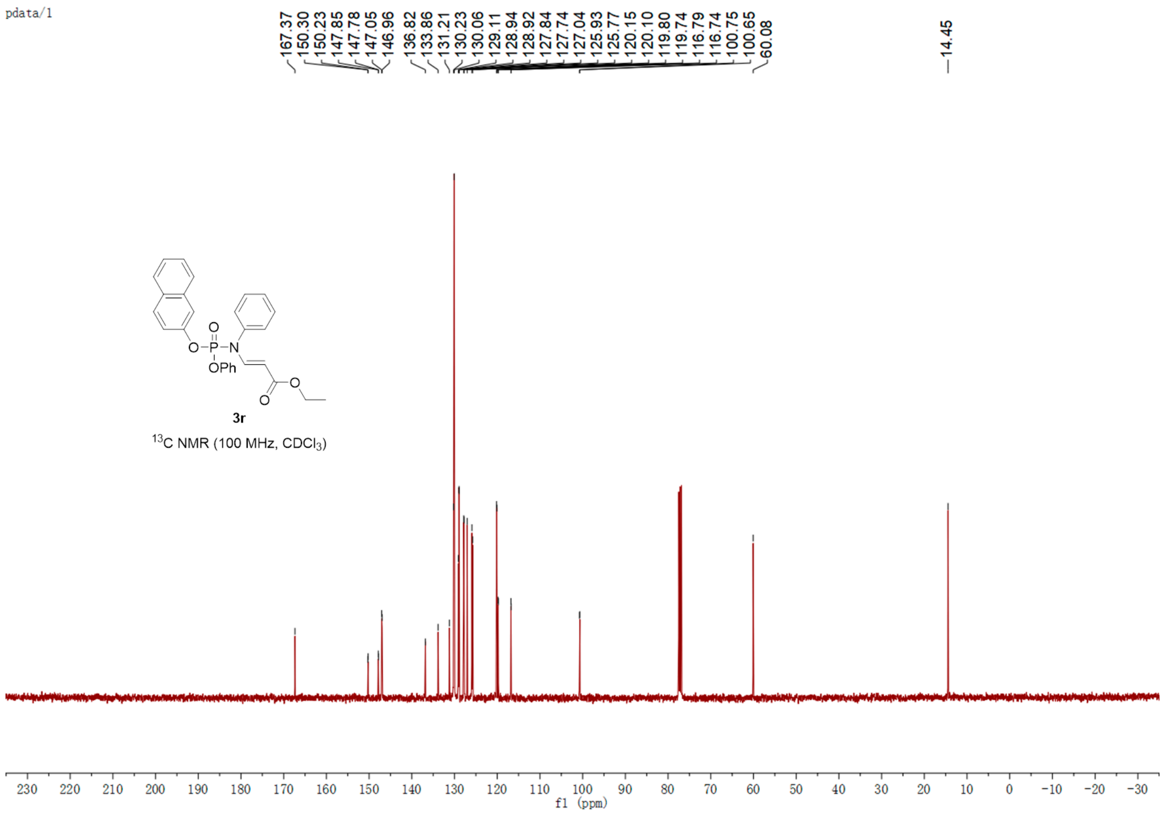
pdata/1
167.37 150.30 150.23 147.85 147.78 147.05 146.96 136.82 133.86 131.21 130.23 130.06 129.11 128.94 128.92 127.84 127.74 127.04 125.93 125.77 120.15 120.10 119.80 119.74 116.79 116.74 100.75 100.65 60.08
14.45
O
O
P
N
OPh
O
O
3r
13C NMR (100 MHz, CDCl3)
f1 (ppm)

pdata/1

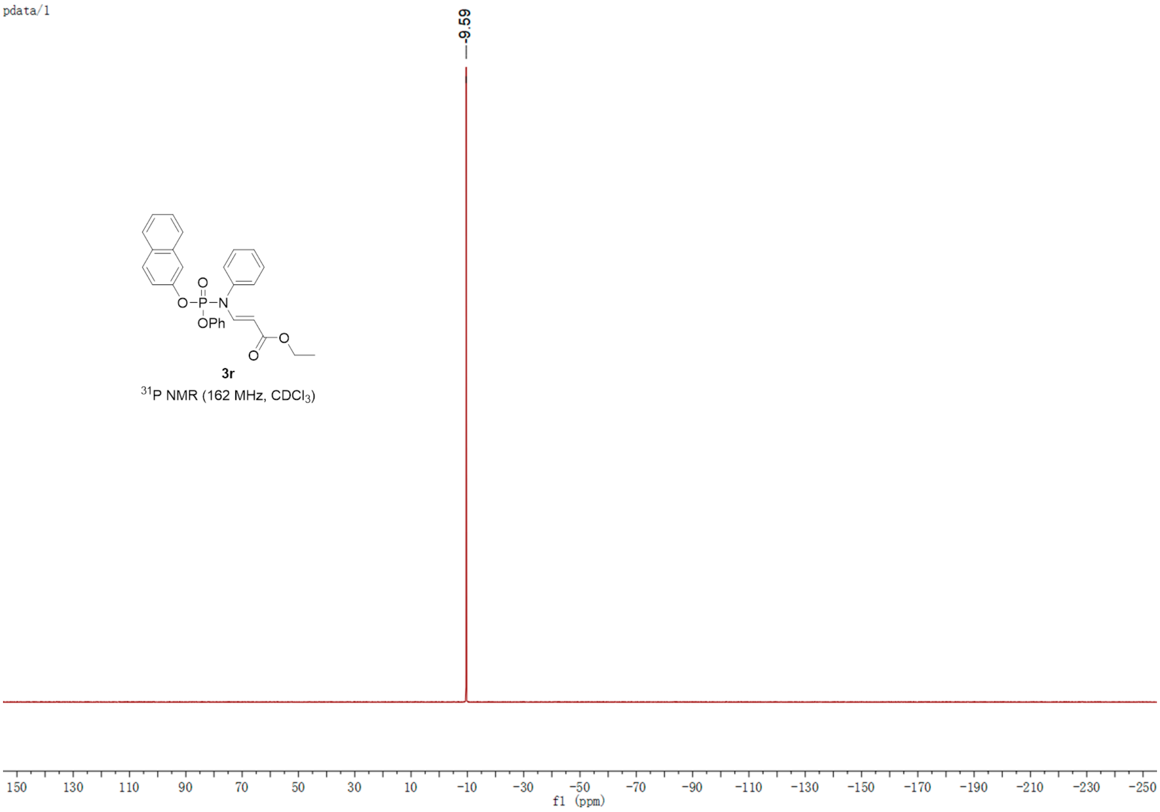

3r

^{31}P NMR (162 MHz, $CDCl_3$)

wg1198.1.1.1r

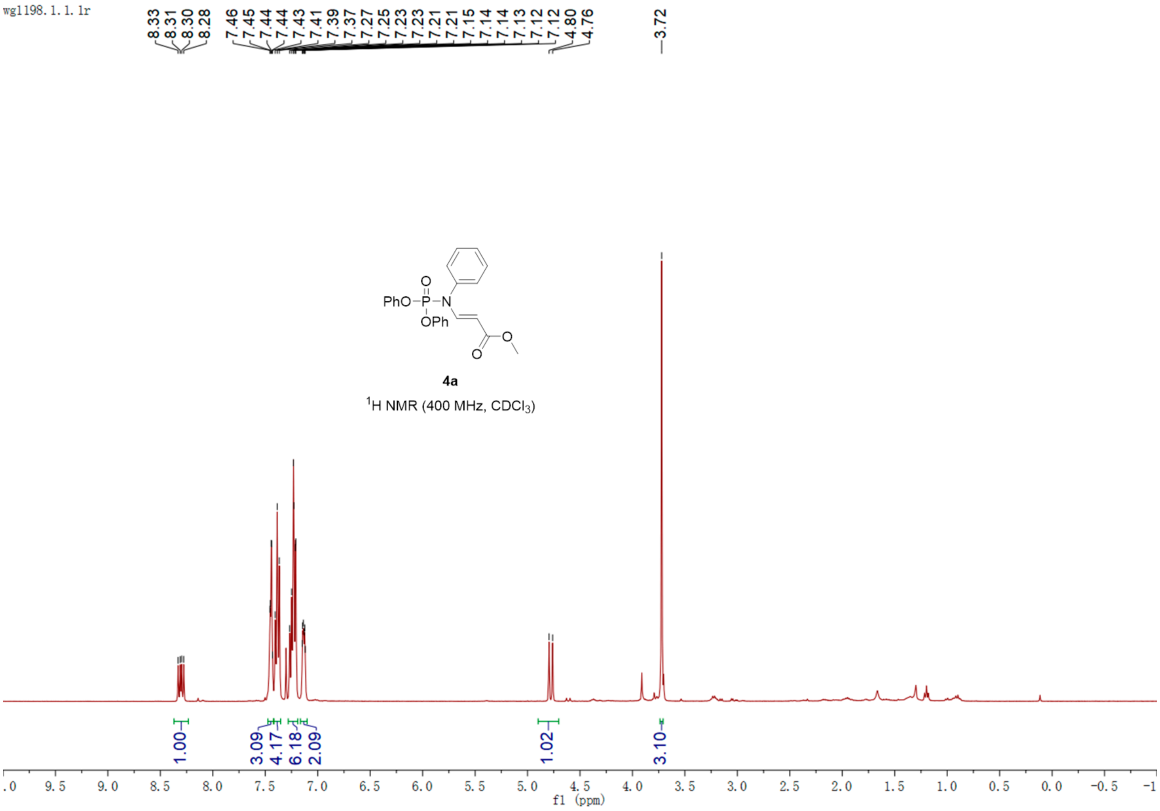

4a

^{1}H NMR (400 MHz, $CDCl_3$)

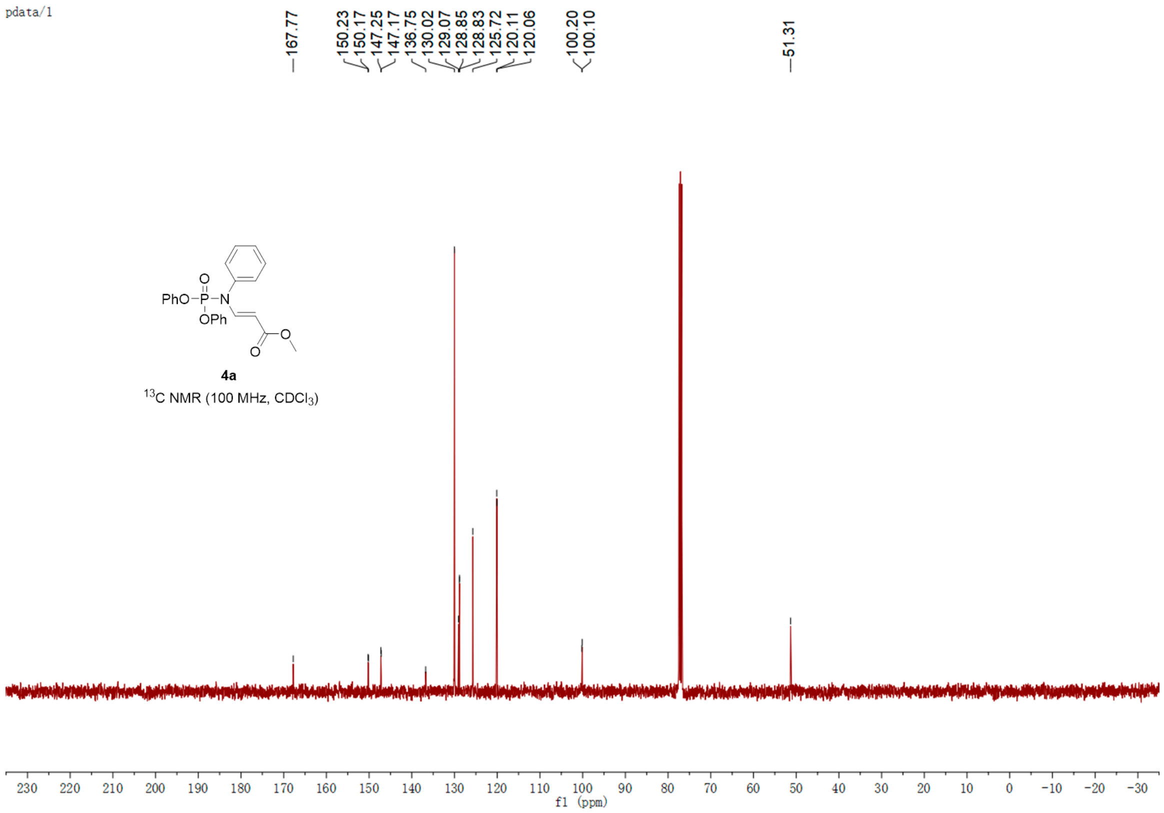

4a

^{13}C NMR (100 MHz, $CDCl_3$)

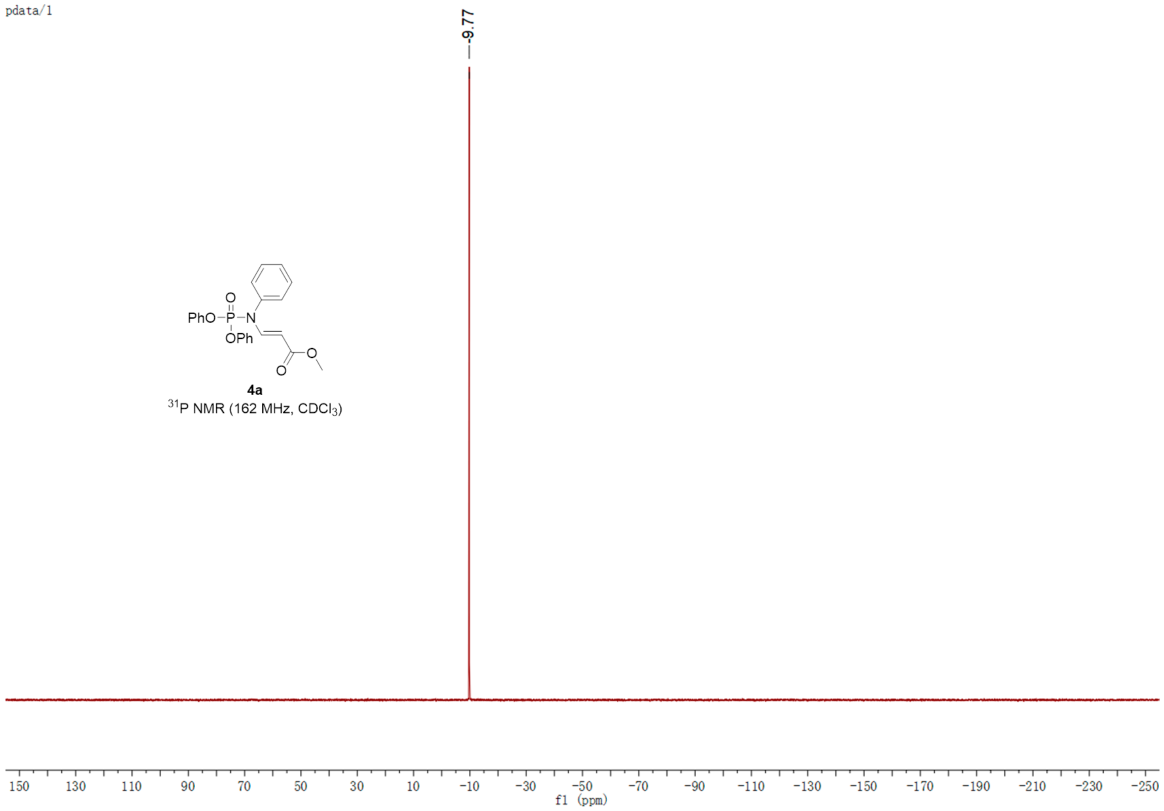

4a

^{31}P NMR (162 MHz, $CDCl_3$)

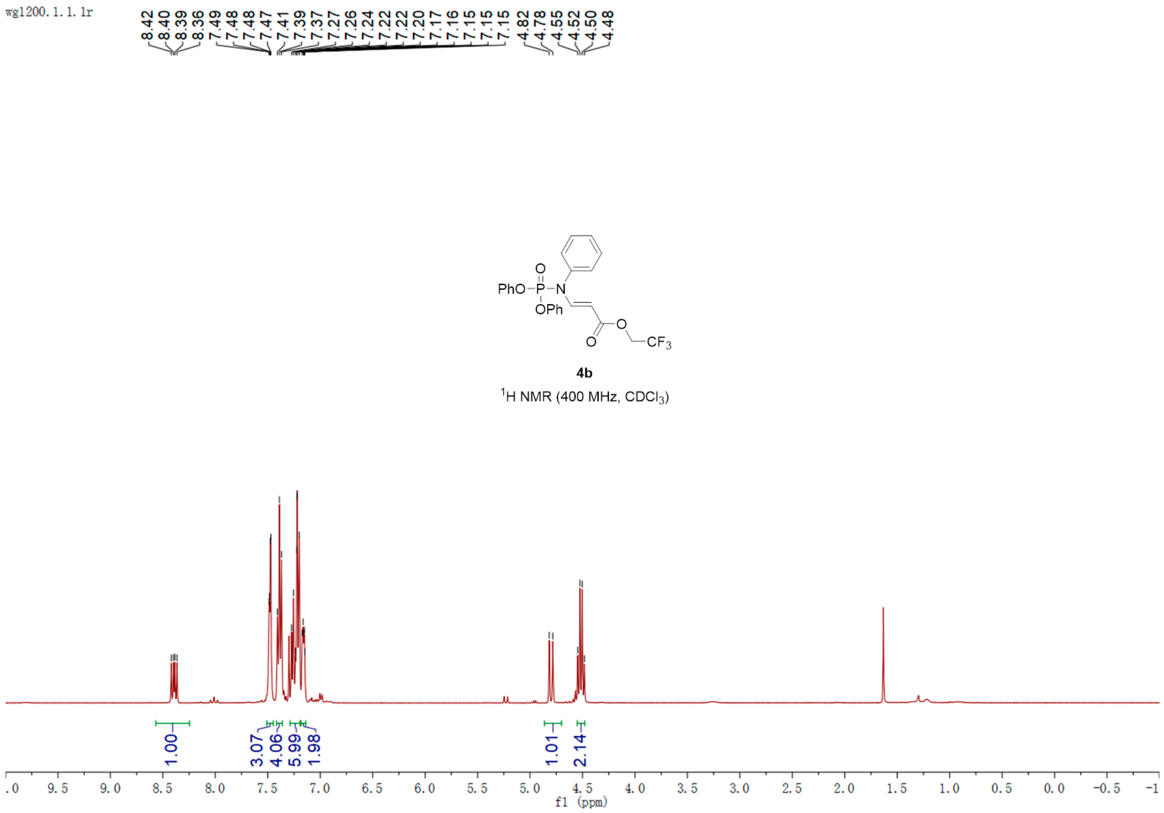
wg1200.1.1.1r
8.42 8.40 8.39 8.36 7.49 7.48 7.48 7.47 7.41 7.39 7.37 7.27 7.26 7.24 7.22 7.22 7.20 7.17 7.16 7.15 7.15 7.15 4.82 4.78 4.55 4.52 4.50 4.48
PhO
O
P
N
OPh
O
O
CF3
4b
1H NMR (400 MHz, CDCl3)
1.00
3.07
4.06
5.99
1.98
1.01
2.14
f1 (ppm)

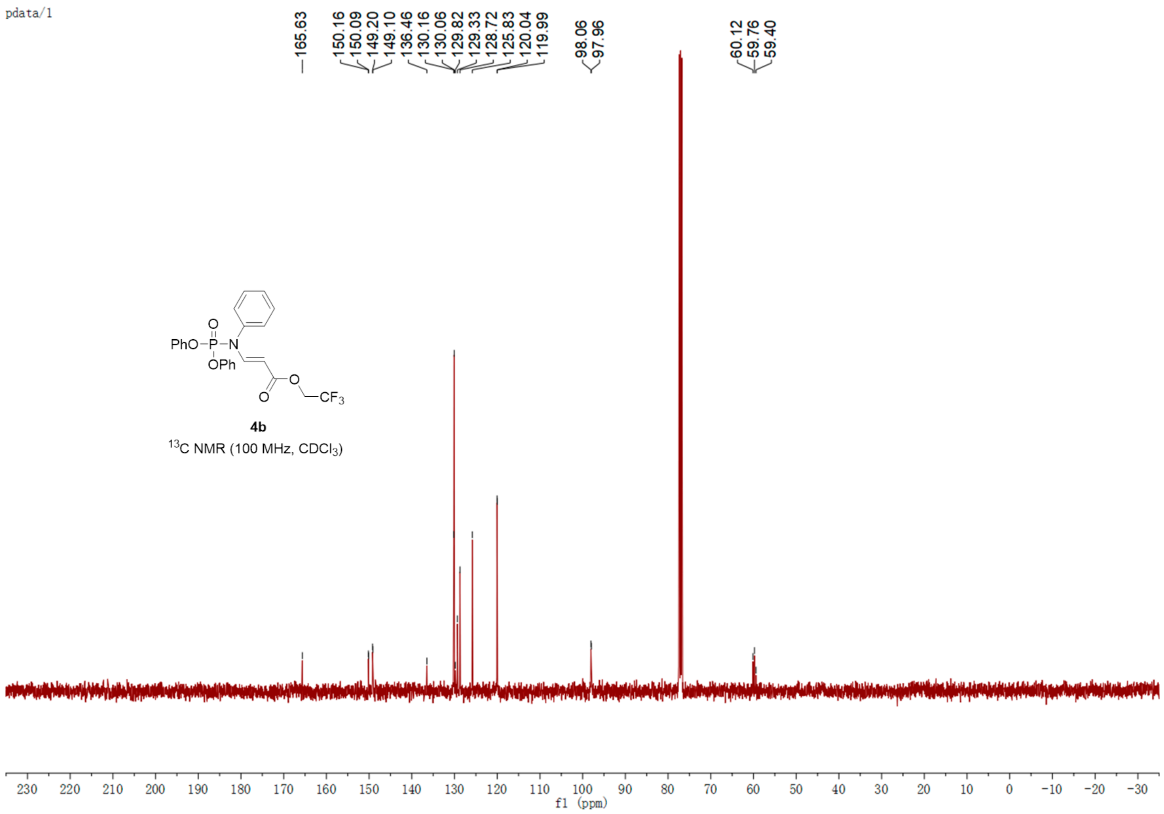
pdata/1
165.63
150.16 150.09 149.20 149.10 136.46 130.16 130.06 129.82 129.33 128.72 125.83 120.04 119.99
98.06 97.96
60.12 59.76 59.40
PhO
O
P
N
OPh
O
O
CF3
4b
13C NMR (100 MHz, CDCl3)
f1 (ppm)

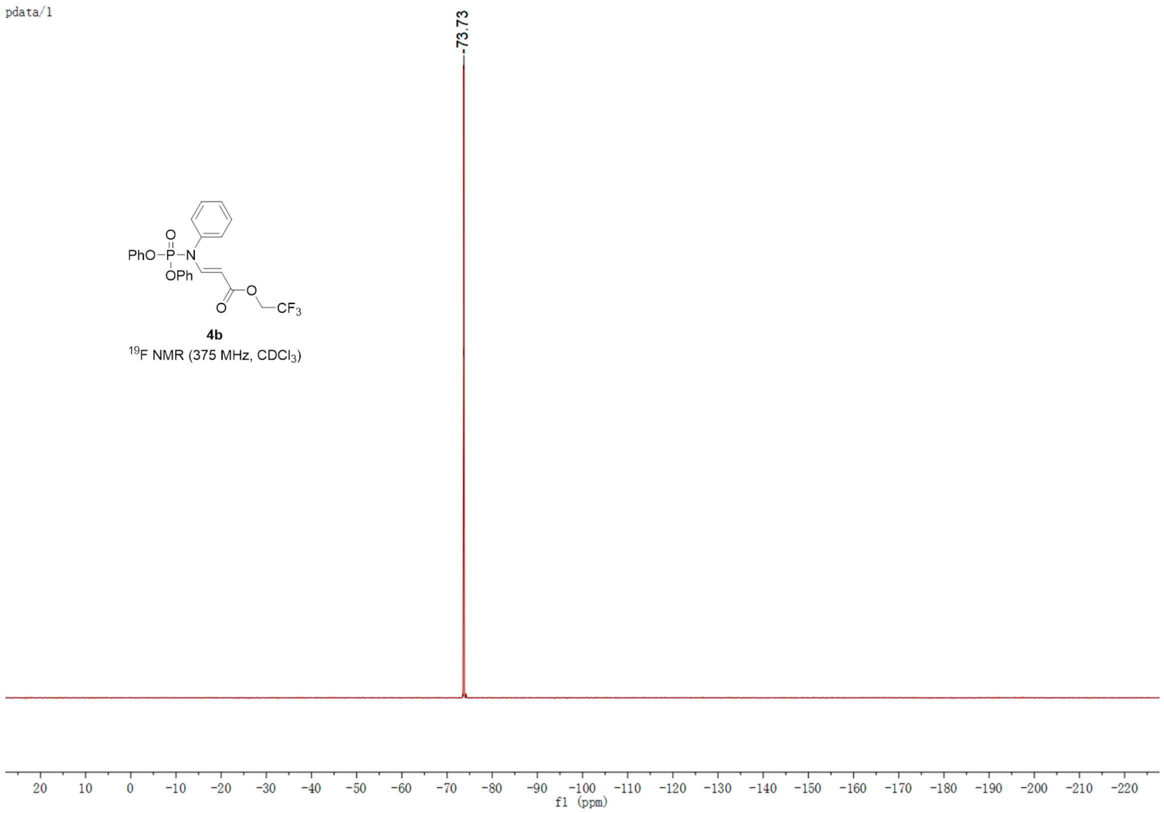
pdata/1
-73.73
PhO
OPh
CF3
4b
19F NMR (375 MHz, CDCl3)
f1 (ppm)

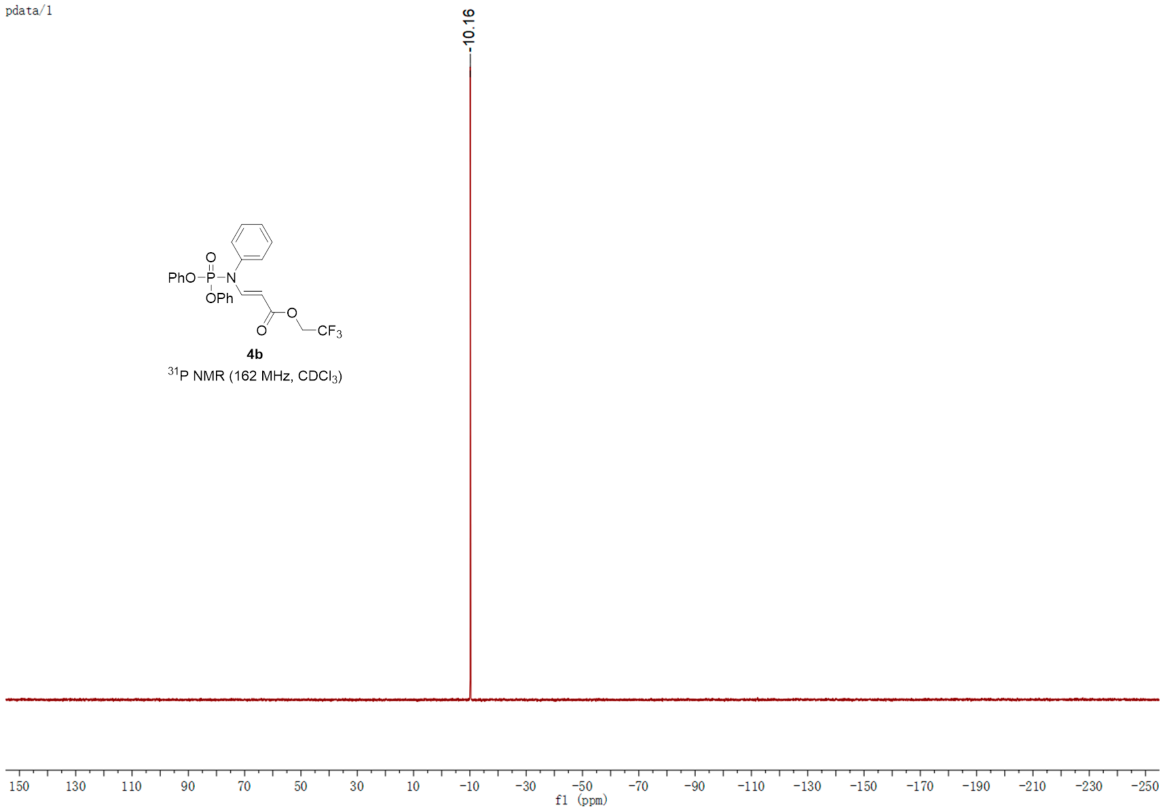
pdata/1
-10.16
PhO
OPh
CF3
4b
31P NMR (162 MHz, CDCl3)
f1 (ppm)

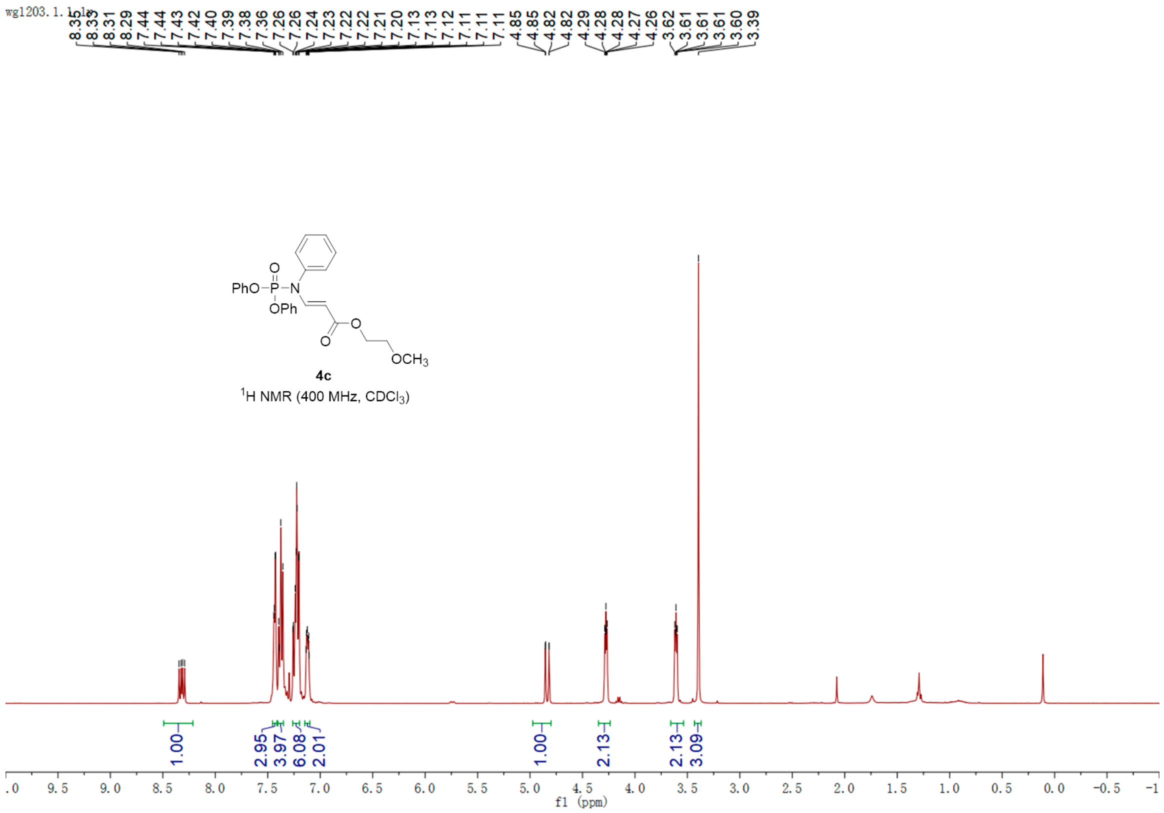

wg1203.1.1.fid
PhO
P
N
O
OPh
O
O
OCH3
4c
1H NMR (400 MHz, CDCl3)
1.00
2.95
3.97
6.08
2.01
1.00
2.13
2.13
3.09
f1 (ppm)

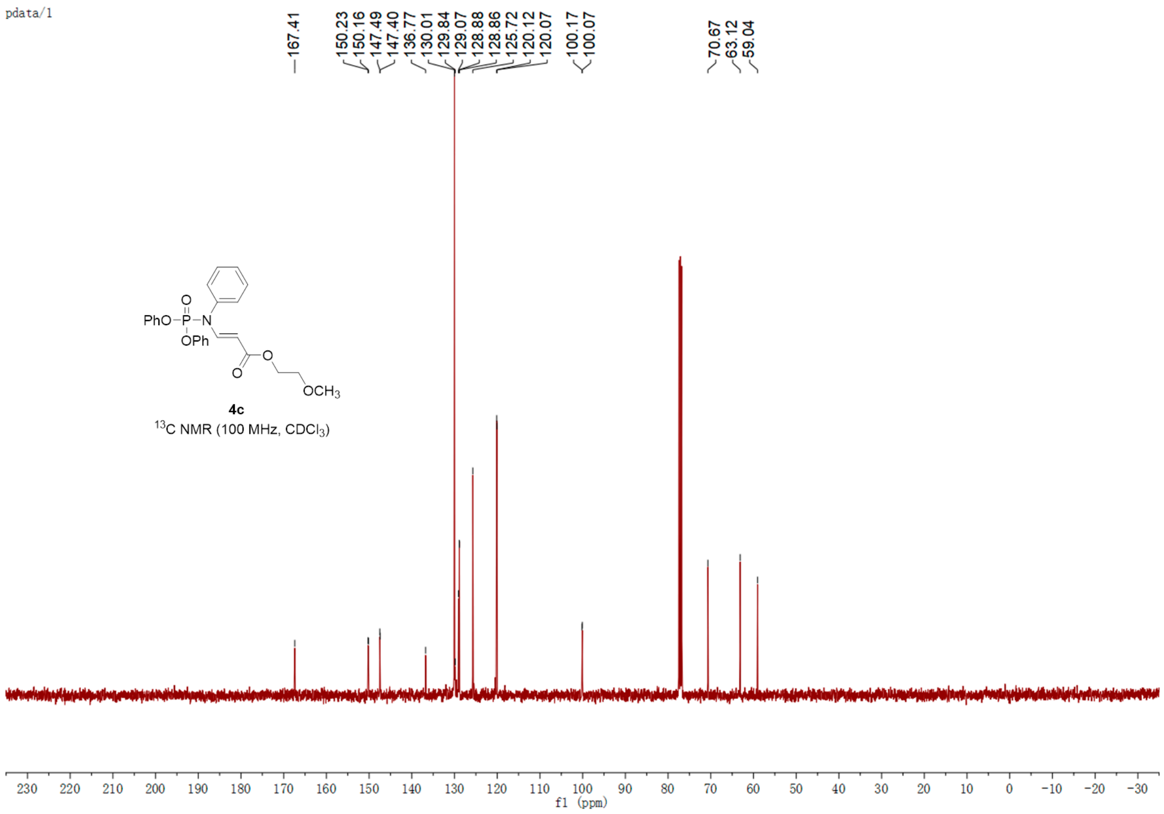

pdata/1
167.41
150.23
150.16
147.49
147.40
136.77
130.01
129.84
129.07
128.88
128.86
125.72
120.12
120.07
100.17
100.07
70.67
63.12
59.04
PhO
P
N
O
OPh
O
O
OCH3
4c
13C NMR (100 MHz, CDCl3)
f1 (ppm)

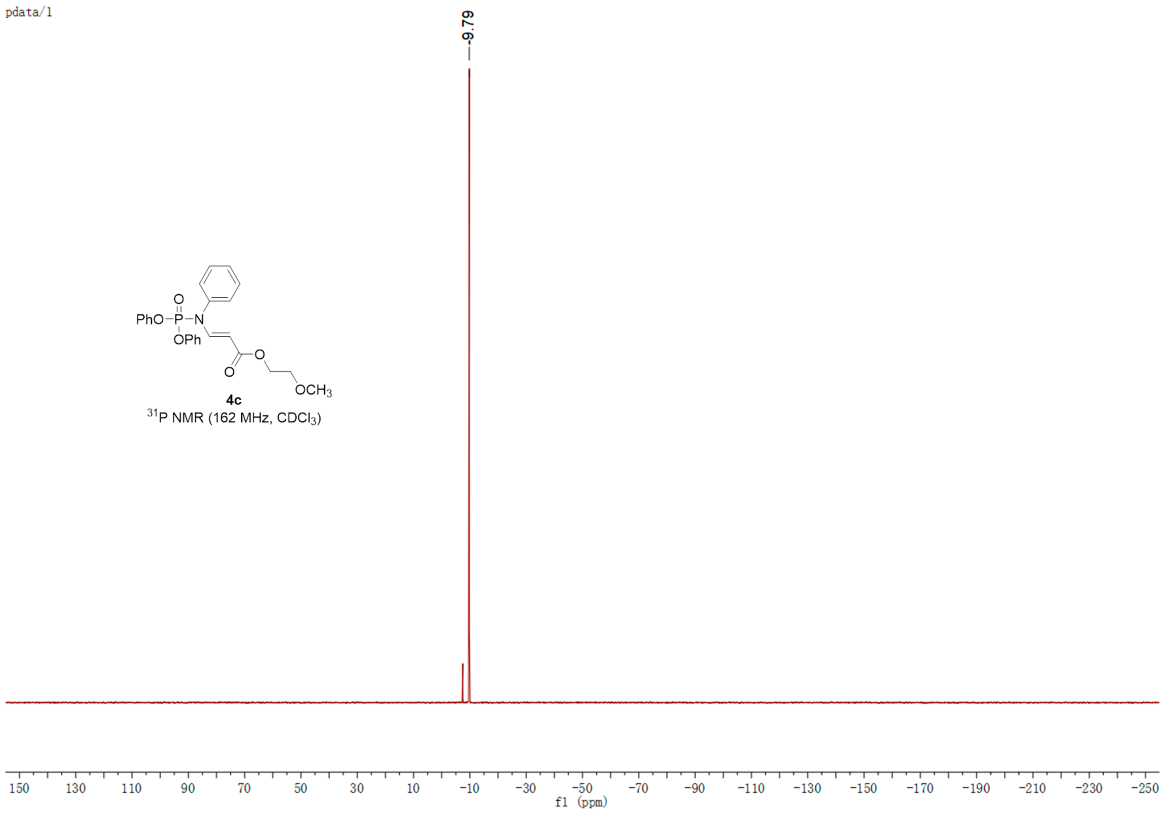
pdata/1
-9.79
PhO
O
P
N
OPh
O
O
OCH3
4c
31P NMR (162 MHz, CDCl3)
150 130 110 90 70 50 30 10 -10 -30 -50 -70 -90 -110 -130 -150 -170 -190 -210 -230 -250
f1 (ppm)

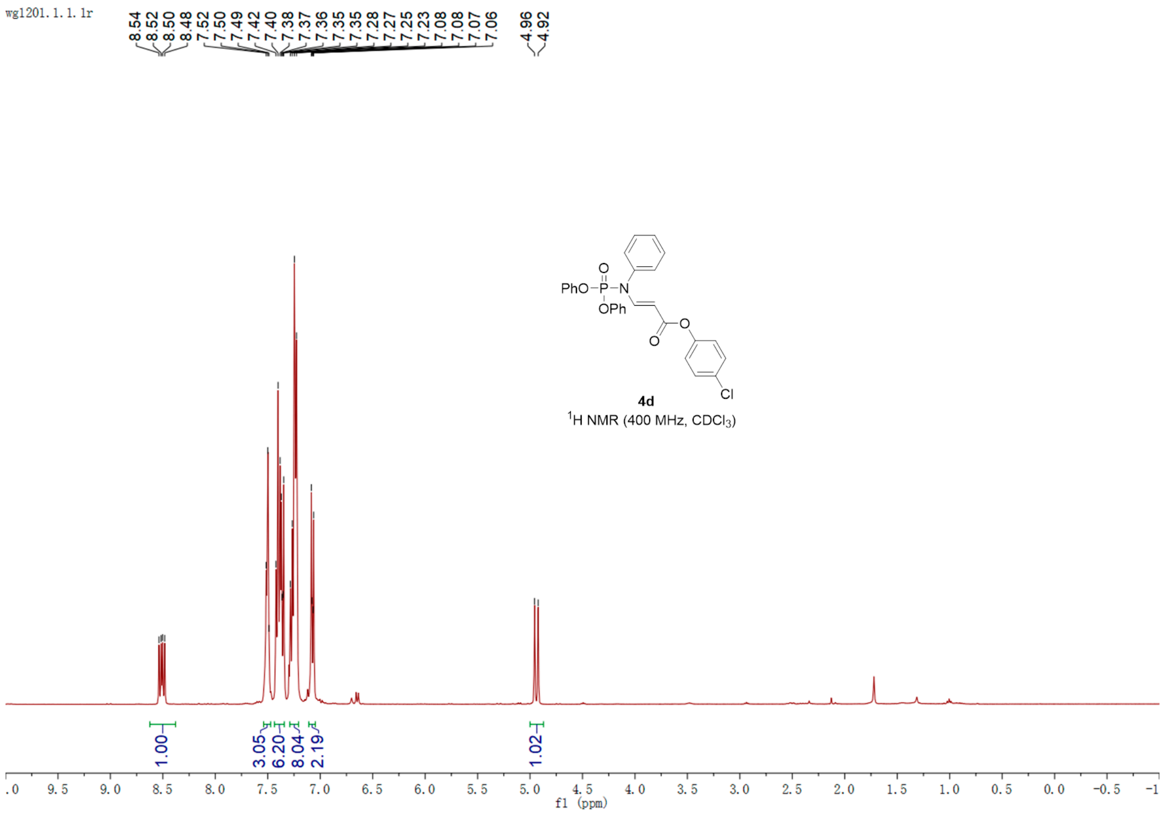
wg1201.1.1.1r
8.54 8.52 8.50 8.48 7.52 7.50 7.49 7.42 7.40 7.38 7.37 7.36 7.35 7.28 7.27 7.25 7.23 7.08 7.07 7.06
4.96 4.92
PhO
O
P
N
OPh
O
O
Cl
4d
1H NMR (400 MHz, CDCl3)
1.00
3.05 6.20 8.04 2.19
1.02
9.5 9.0 8.5 8.0 7.5 7.0 6.5 6.0 5.5 5.0 4.5 4.0 3.5 3.0 2.5 2.0 1.5 1.0 0.5 0.0 -0.5 -1
f1 (ppm)

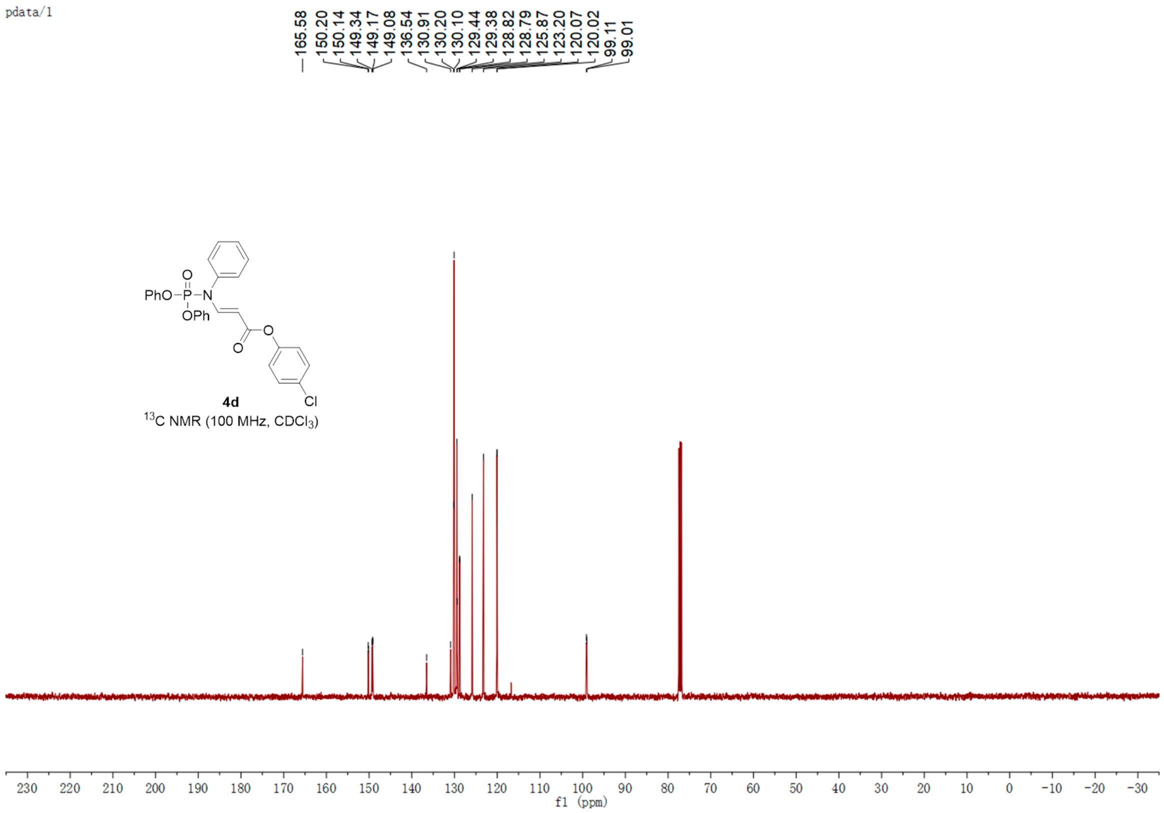
pdata/1
165.58
150.20
150.14
149.34
149.17
149.08
136.54
130.91
130.20
130.10
129.44
129.38
128.82
128.79
125.87
123.20
120.07
120.02
99.11
99.01
PhO
OPh
Cl
4d
^{13}C NMR (100 MHz, $CDCl_3$)
f1 (ppm)

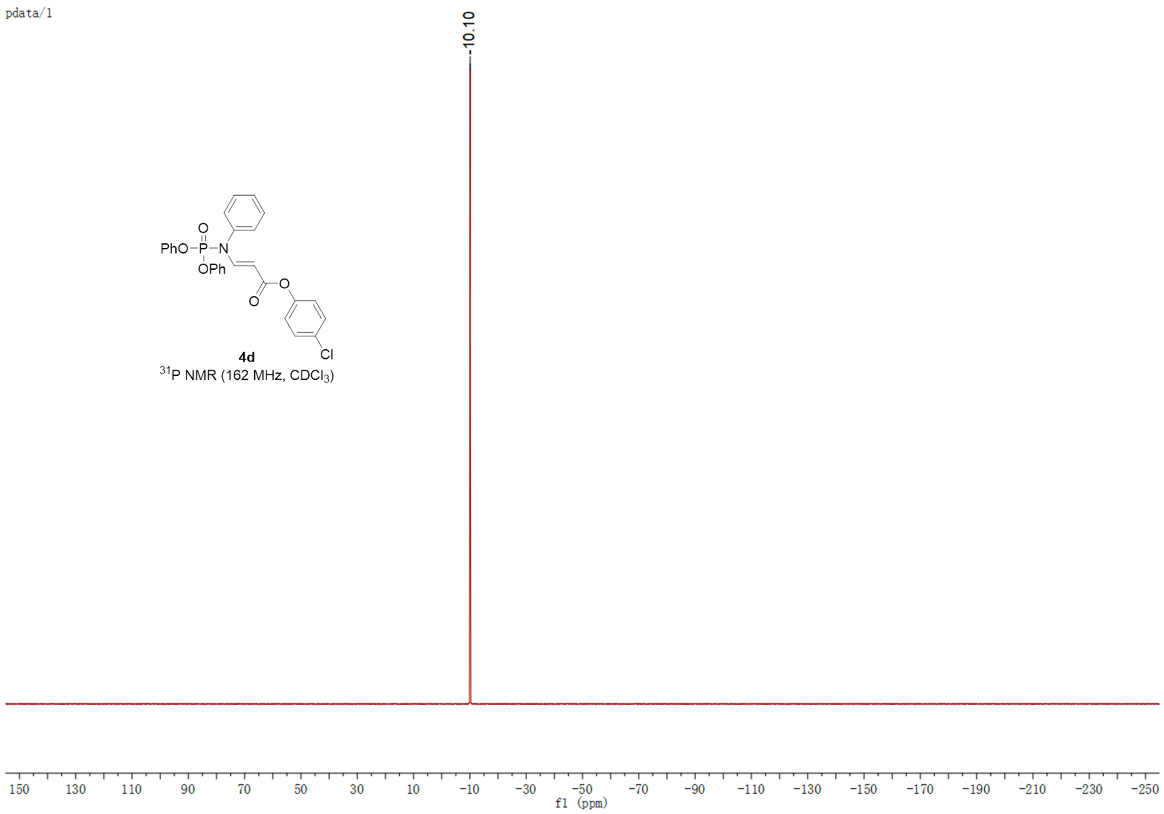
pdata/1
-10.10
PhO
OPh
Cl
4d
^{31}P NMR (162 MHz, $CDCl_3$)
f1 (ppm)

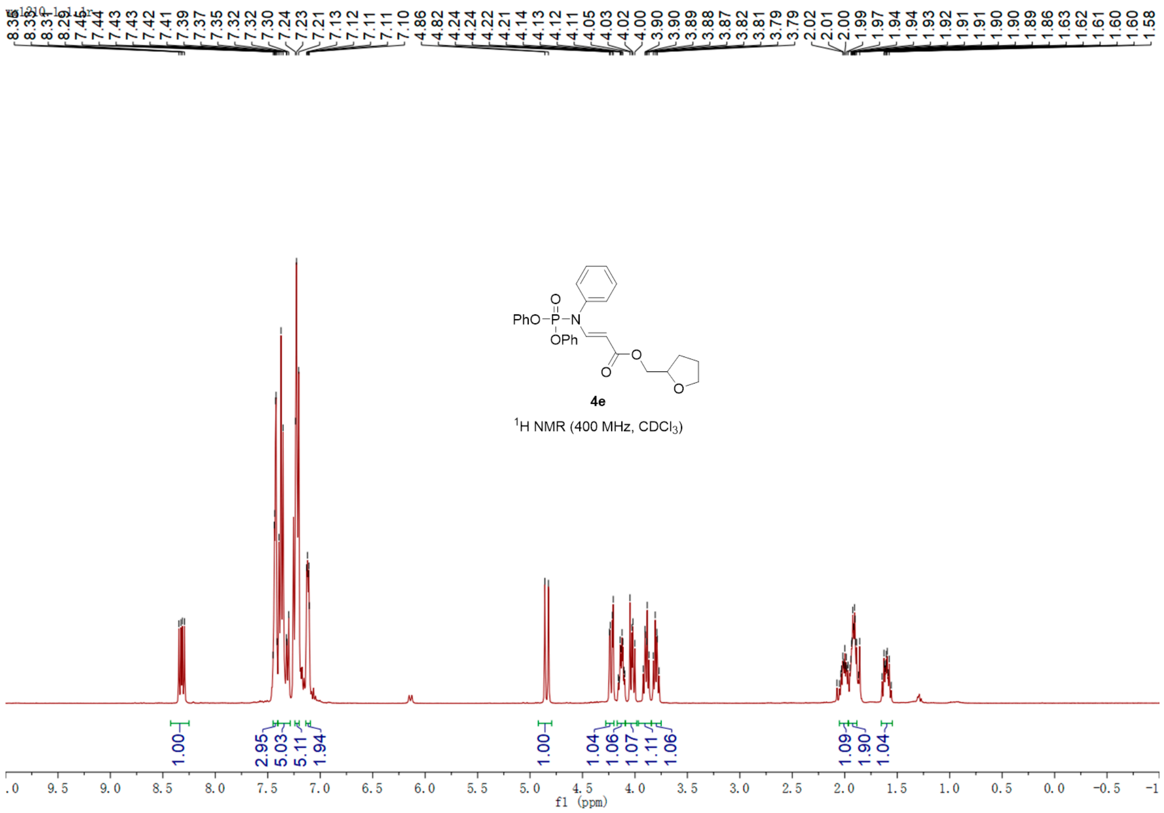
8.33 8.33 8.31 8.29 7.45 7.44 7.43 7.43 7.42 7.41 7.39 7.37 7.35 7.32 7.32 7.30 7.24 7.23 7.21 7.13 7.12 7.11 7.11 7.10 4.86 4.82 4.24 4.24 4.22 4.21 4.14 4.13 4.12 4.11 4.05 4.03 4.02 4.00 3.90 3.90 3.89 3.88 3.87 3.82 3.81 3.79 2.02 2.01 2.00 1.99 1.97 1.94 1.94 1.93 1.92 1.91 1.91 1.90 1.89 1.86 1.63 1.62 1.61 1.60 1.60 1.58
PhO
P
N
O
OPh
O
O
O
4e
^{1}H NMR (400 MHz, $CDCl_3$)
1.00 2.95 5.03 5.11 1.94 1.00 1.04 1.06 1.07 1.11 1.06 1.09 1.90 1.04
f1 (ppm)

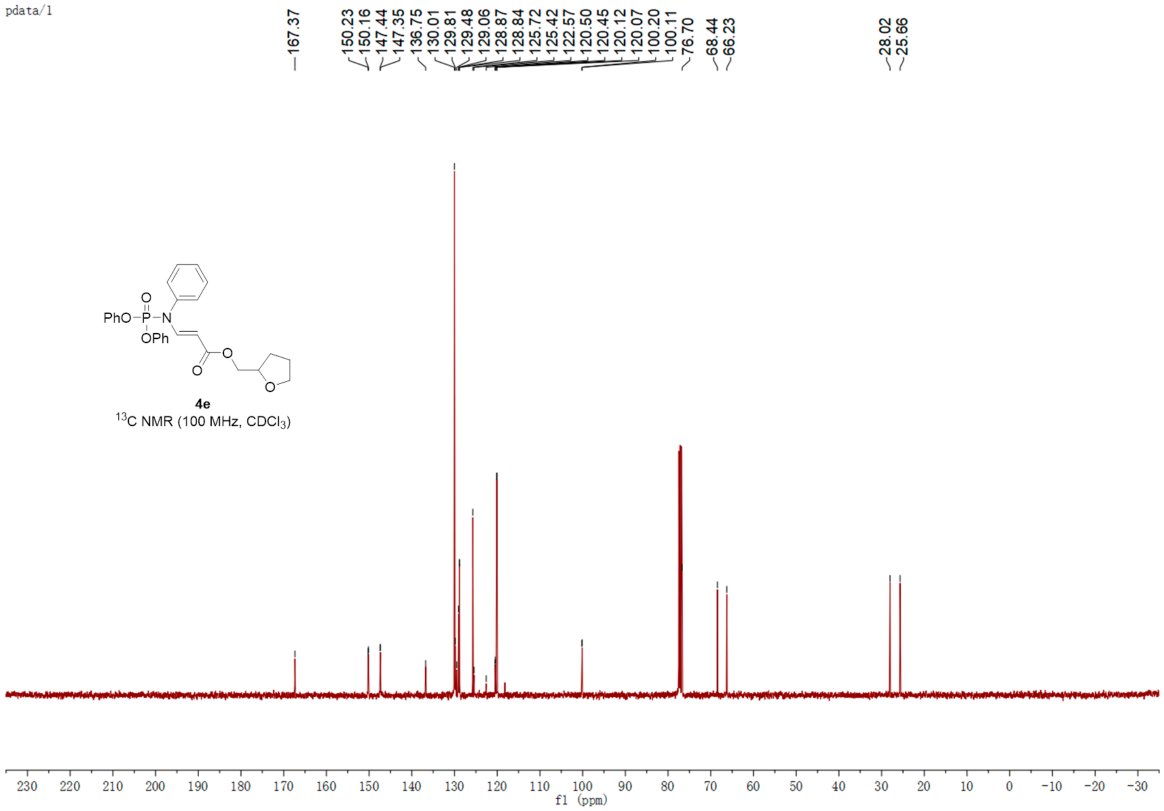
pdata/1
167.37 150.23 150.16 147.44 147.35 136.75 130.01 129.81 129.48 129.06 128.87 128.84 125.72 125.42 122.57 120.50 120.45 120.12 120.07 100.20 100.11 76.70 68.44 66.23 28.02 25.66
PhO
P
N
O
OPh
O
O
O
4e
^{13}C NMR (100 MHz, $CDCl_3$)
f1 (ppm)

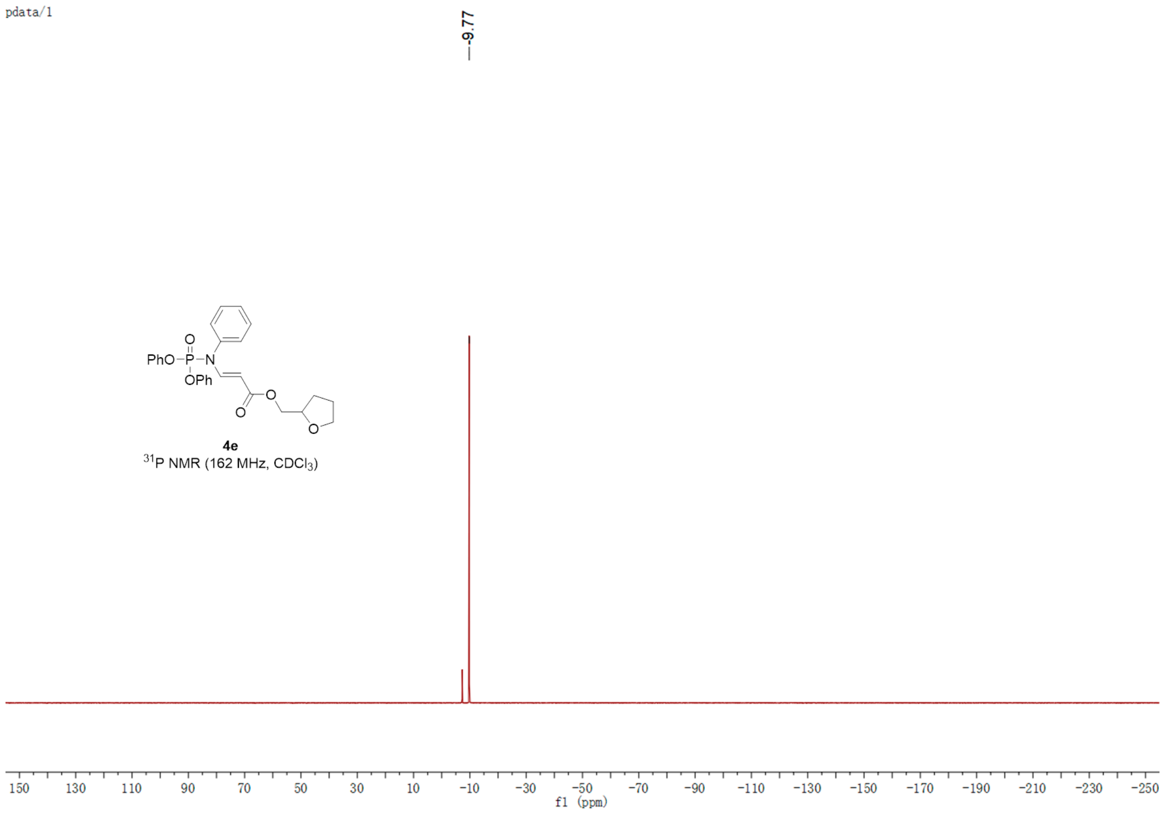

4e

^{31}P NMR (162 MHz, $CDCl_3$)

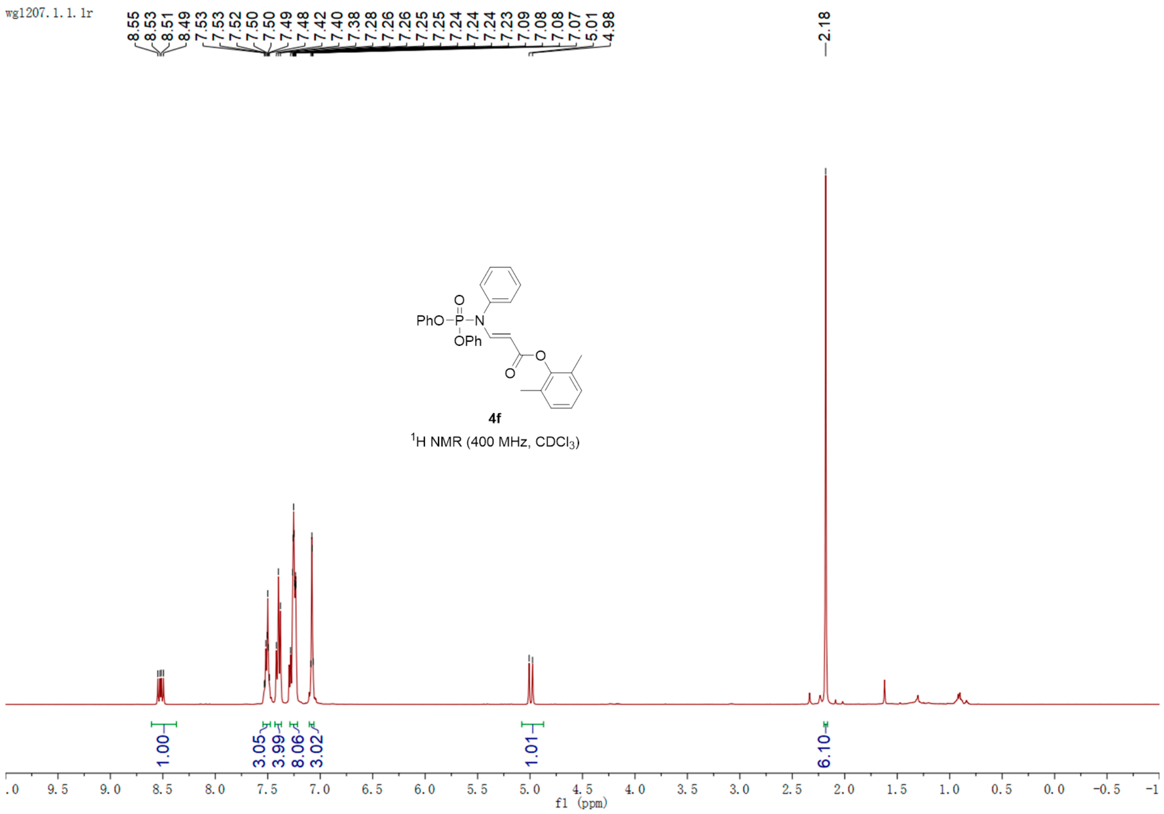

4f

^{1}H NMR (400 MHz, $CDCl_3$)

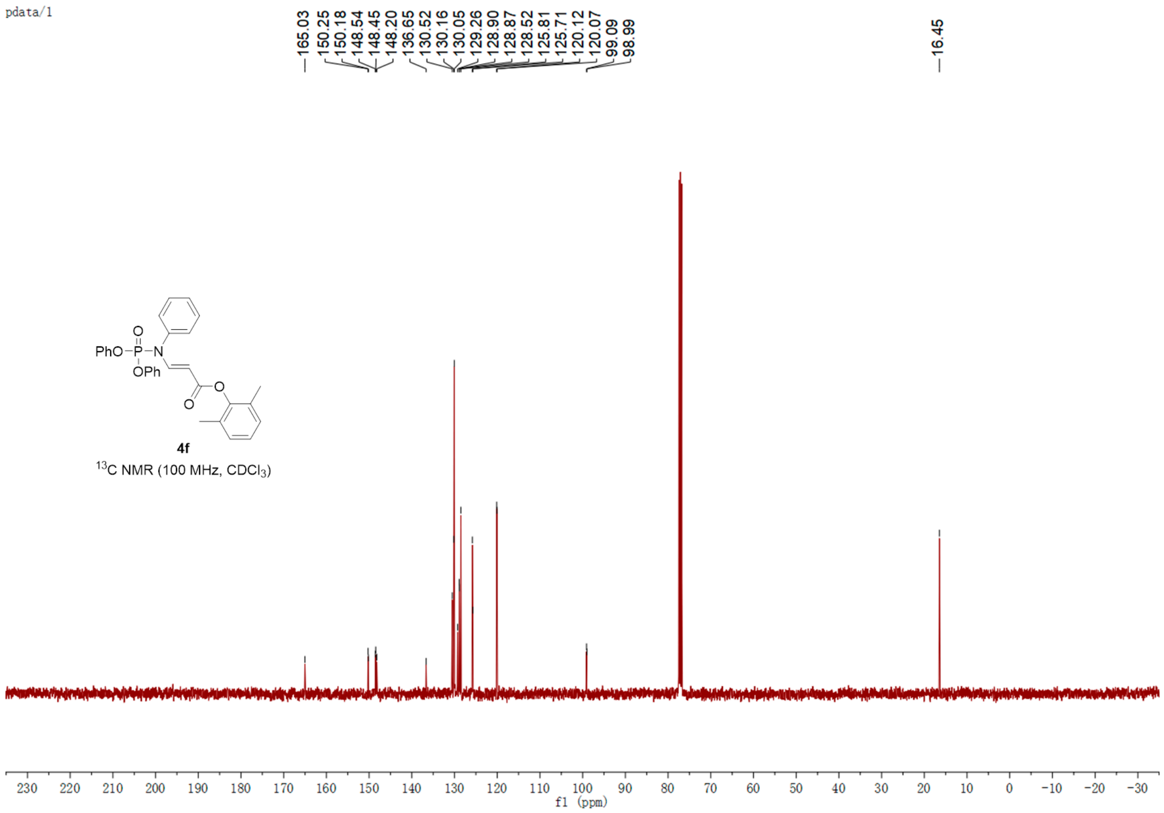
pdata/1
165.03
150.25
150.18
148.54
148.45
148.20
136.65
130.52
130.16
130.05
129.26
128.90
128.87
128.52
125.81
125.71
120.12
120.07
99.09
98.99
16.45
PhO
P
N
O
OPh
O
O
4f
^{13}C NMR (100 MHz, $CDCl_3$)
f1 (ppm)

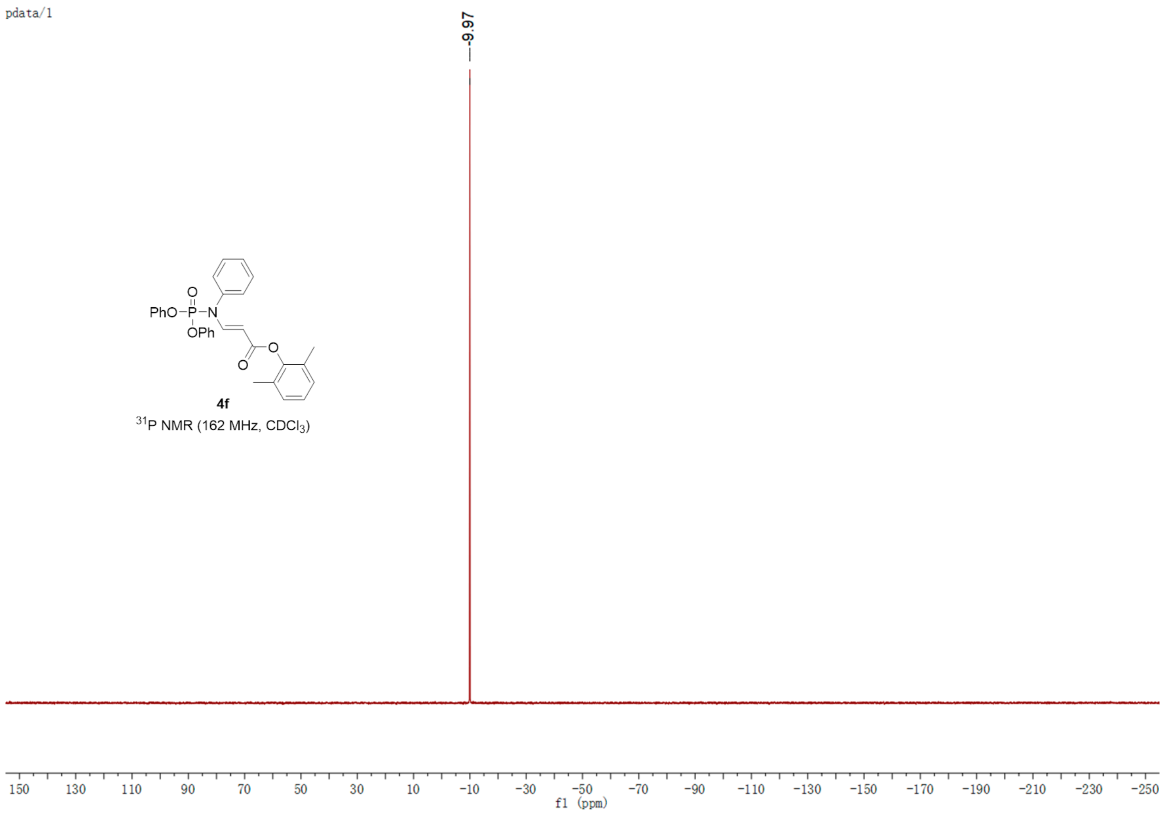
pdata/1
-9.97
PhO
P
N
O
OPh
O
O
4f
^{31}P NMR (162 MHz, $CDCl_3$)
f1 (ppm)

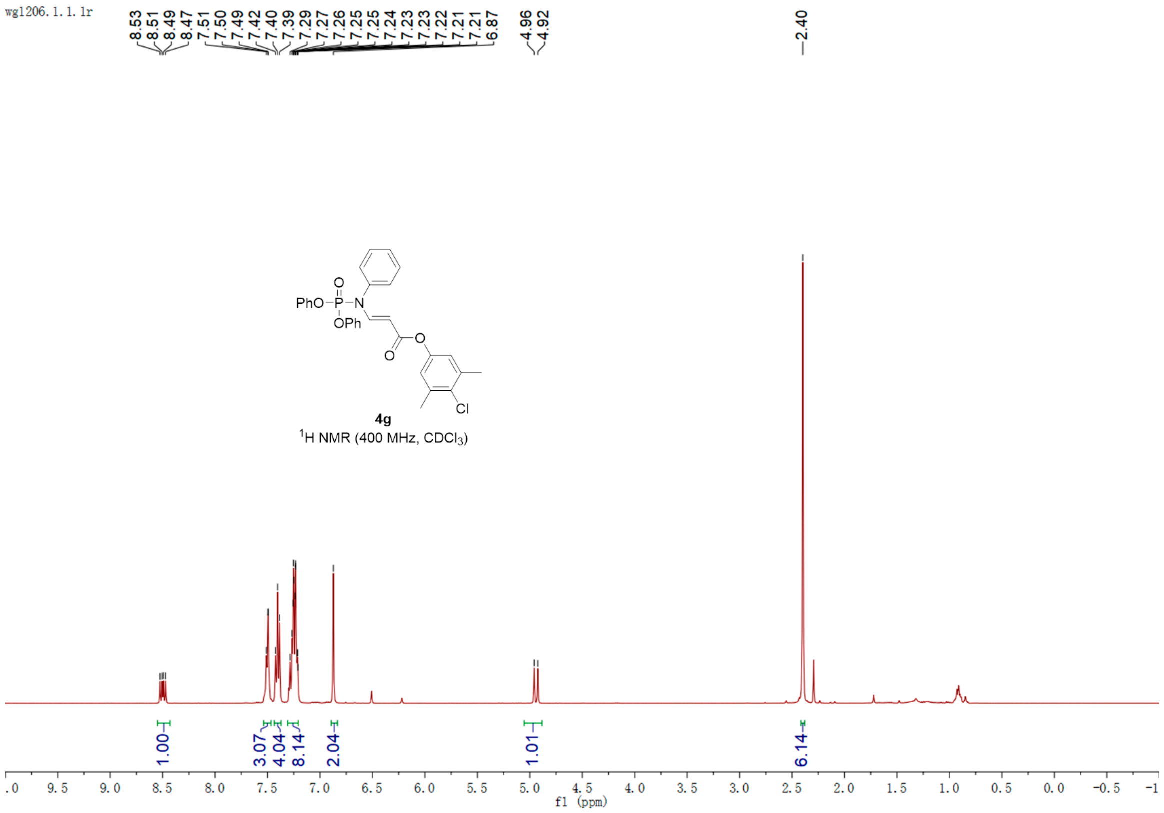
wg1206.1.1.1r
8.53
8.51
8.49
8.47
7.51
7.50
7.49
7.42
7.40
7.39
7.29
7.27
7.26
7.25
7.25
7.24
7.23
7.23
7.22
7.21
7.21
6.87
4.96
4.92
2.40
PhO
O
P
N
OPh
O
O
Cl
4g
^{1}H NMR (400 MHz, $CDCl_3$)
1.00
3.07
4.04
8.14
2.04
1.01
6.14
f1 (ppm)

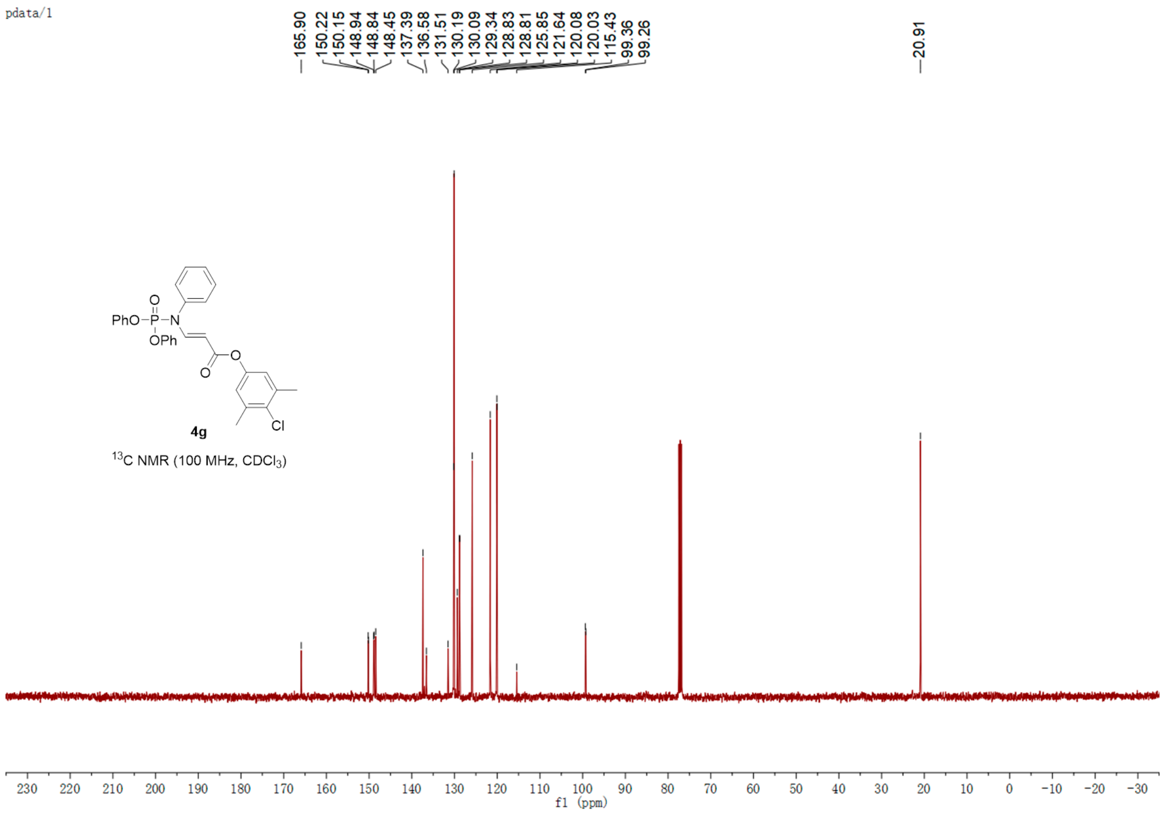
pdata/1
165.90
150.22
150.15
148.94
148.84
148.45
137.39
136.58
131.51
130.19
130.09
129.34
128.83
128.81
125.85
121.64
120.08
120.03
115.43
99.36
99.26
20.91
PhO
O
P
N
OPh
O
O
Cl
4g
^{13}C NMR (100 MHz, $CDCl_3$)
f1 (ppm)

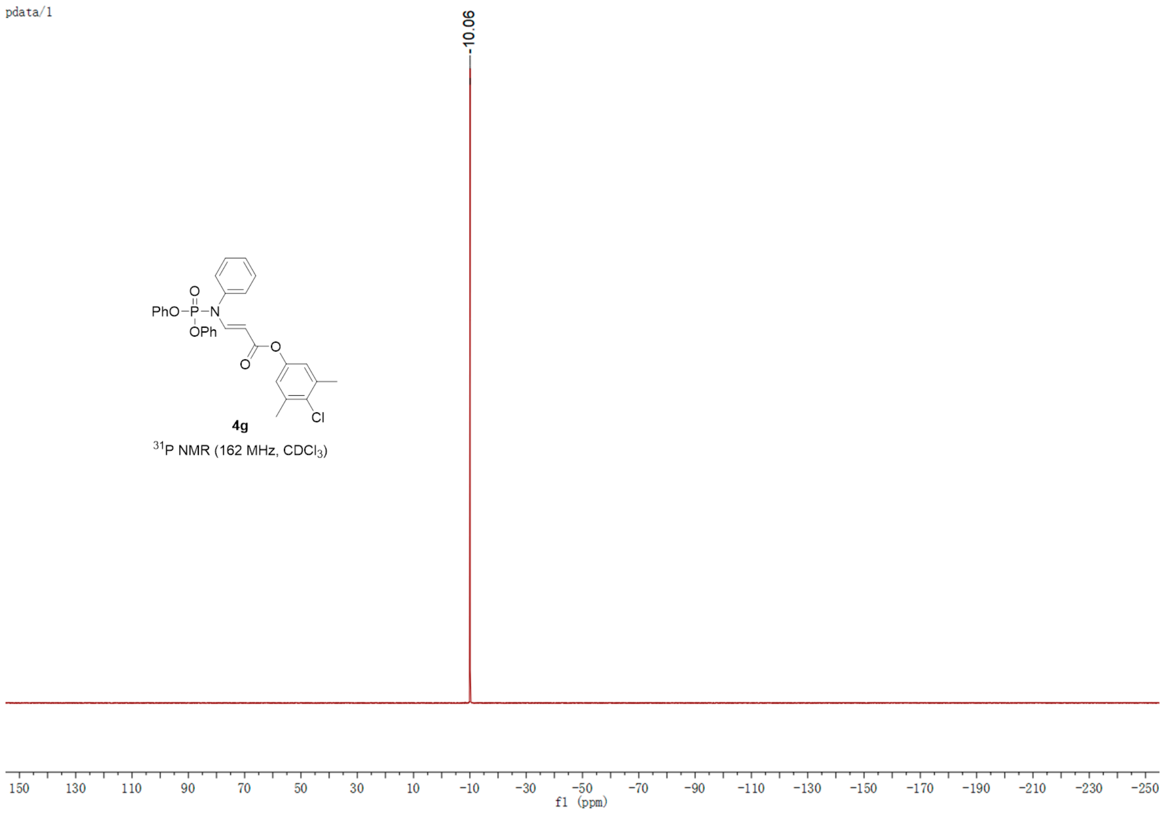
pdata/1
-10.06
PhO
O
P
N
OPh
O
O
Cl
4g
31P NMR (162 MHz, CDCl3)
f1 (ppm)

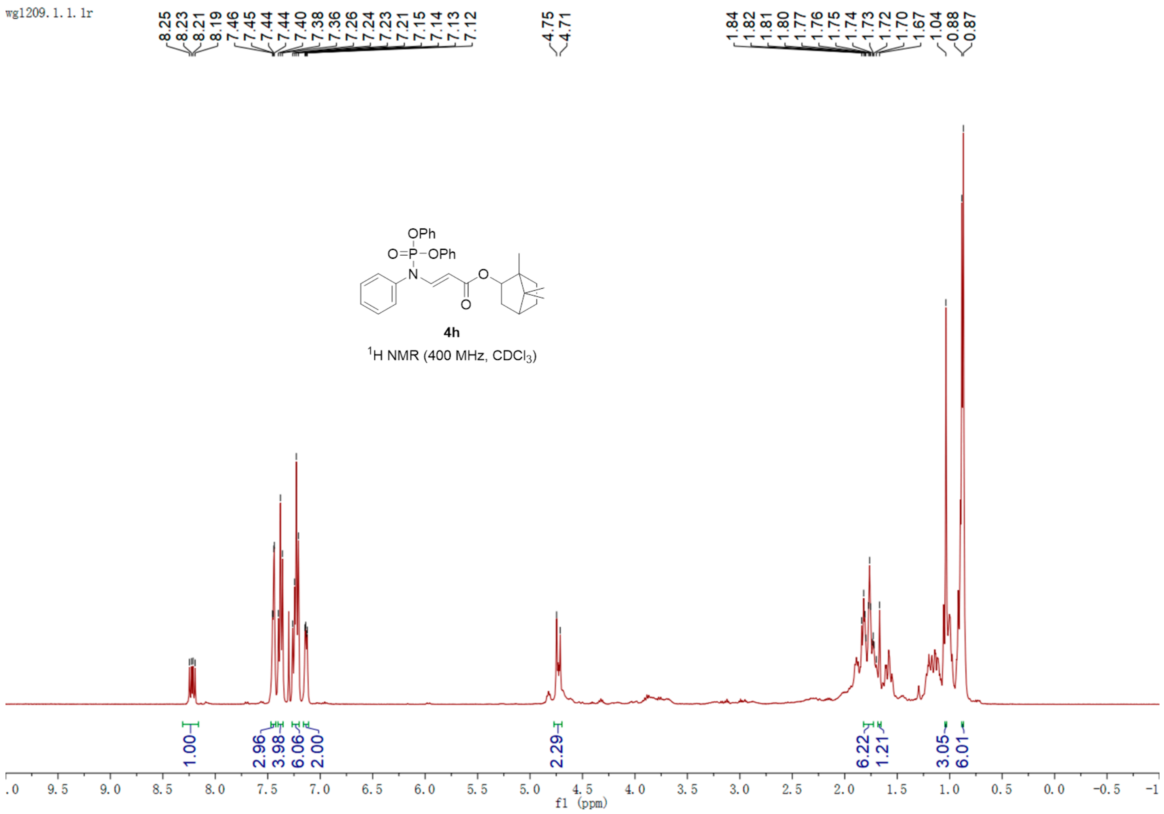
wg1209.1.1.1r
8.25
8.23
8.21
8.19
7.46
7.45
7.44
7.44
7.40
7.38
7.36
7.26
7.24
7.23
7.21
7.15
7.14
7.13
7.12
4.75
4.71
1.84
1.82
1.81
1.80
1.77
1.76
1.75
1.74
1.73
1.72
1.70
1.67
1.04
0.88
0.87
OPh
O=P-OPh
N
O
O
4h
1H NMR (400 MHz, CDCl3)
1.00
2.96
3.98
6.06
2.00
2.29
6.22
1.21
3.05
6.01
f1 (ppm)

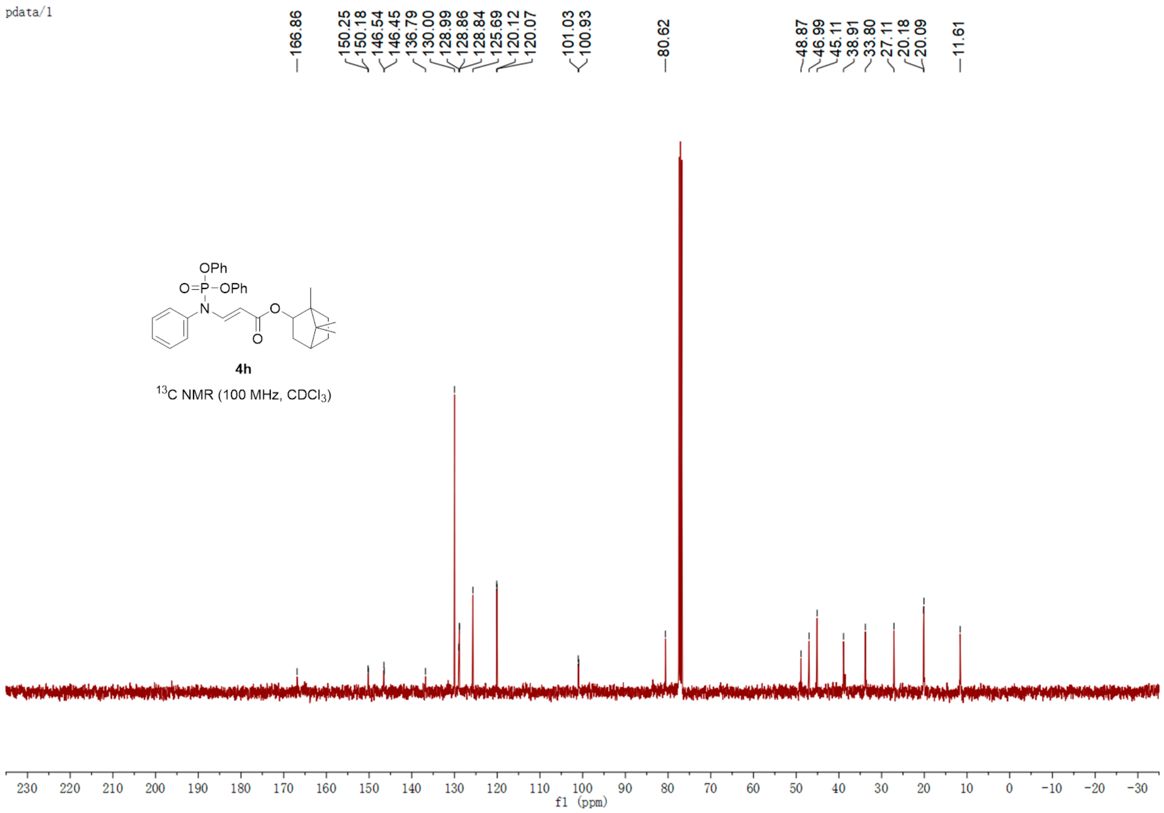
pdata/1
166.86
150.25
150.18
146.54
146.45
136.79
130.00
128.99
128.86
128.84
125.69
120.12
120.07
101.03
100.93
80.62
48.87
46.99
45.11
38.91
33.80
27.11
20.18
20.09
11.61
OPh
O=P-OPh
N
O
O
4h
13C NMR (100 MHz, CDCl3)
f1 (ppm)

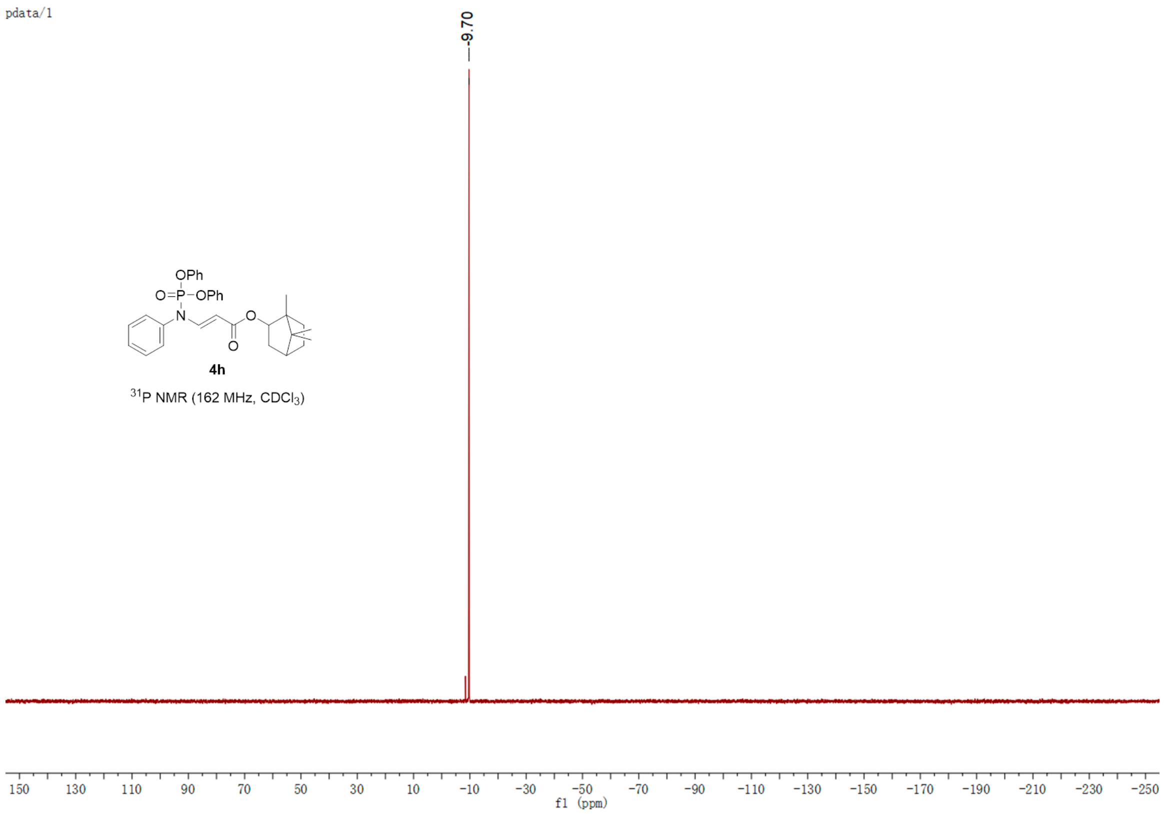
pdata/1
-9.70
OPh
O=P-OPh
N
O
O
4h
31P NMR (162 MHz, CDCl3)
f1 (ppm)

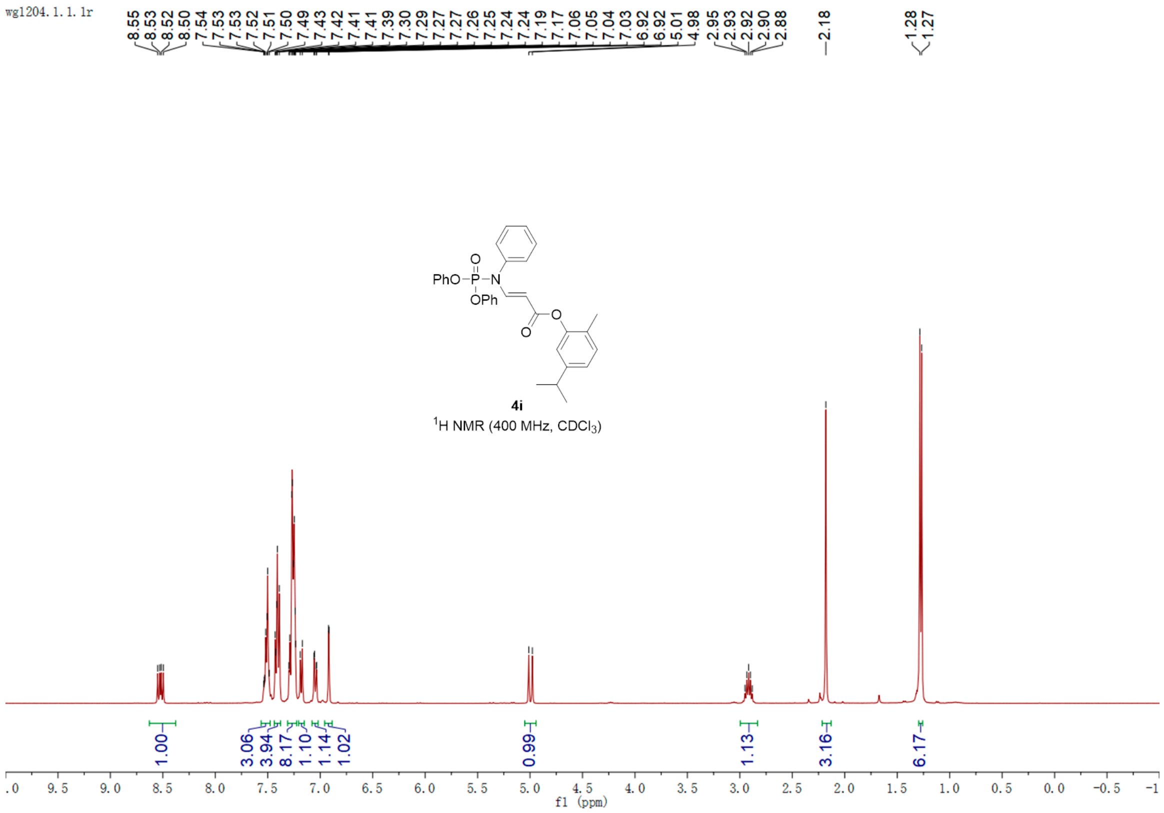
wg1204.1.1.1r
4i
^{1}H NMR (400 MHz, $CDCl_3$)
f1 (ppm)

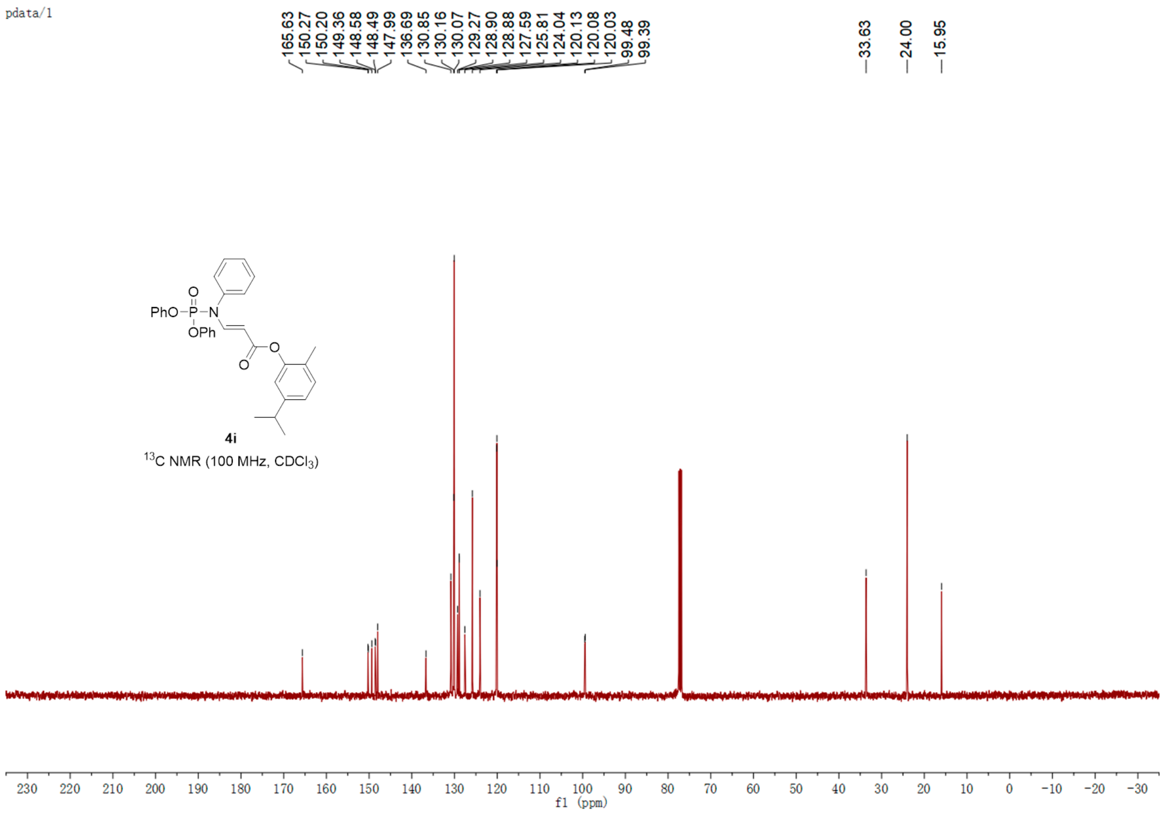
pdata/1
4i
^{13}C NMR (100 MHz, $CDCl_3$)
f1 (ppm)

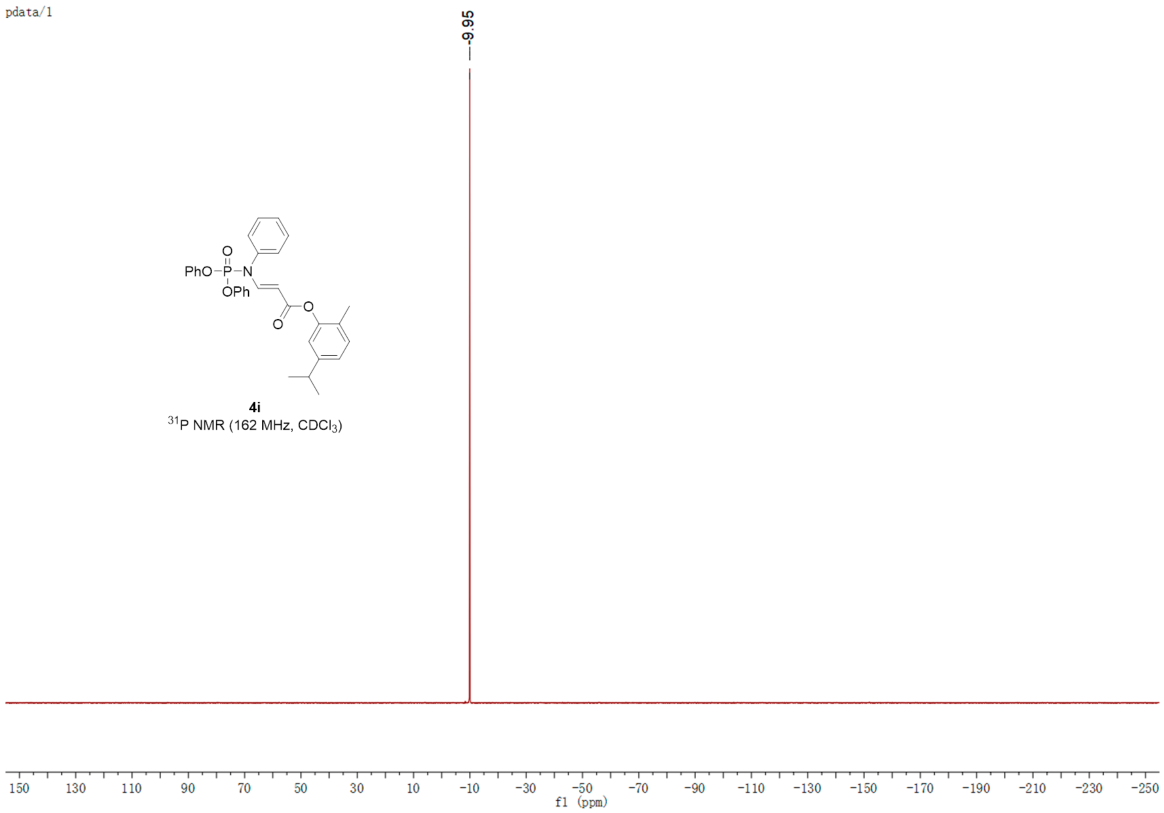
pdata/1
-9.95
PhO
O
P
N
OPh
O
O
4i
^{31}P NMR (162 MHz, CDCl$_3$)
f1 (ppm)

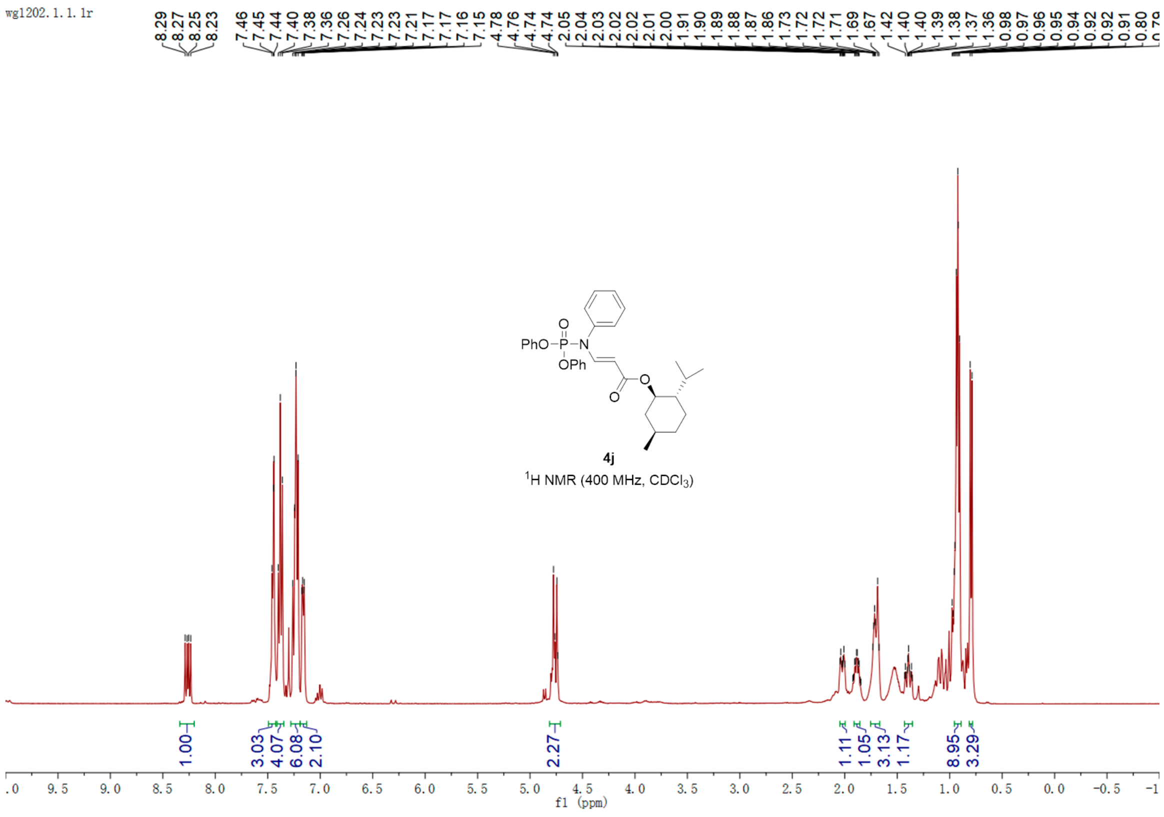
wg1202.1.1.1r
PhO
O
P
N
OPh
O
O
4j
^{1}H NMR (400 MHz, CDCl$_3$)
1.00
3.03
4.07
6.08
2.10
2.27
1.11
1.05
3.13
1.17
8.95
3.29
f1 (ppm)

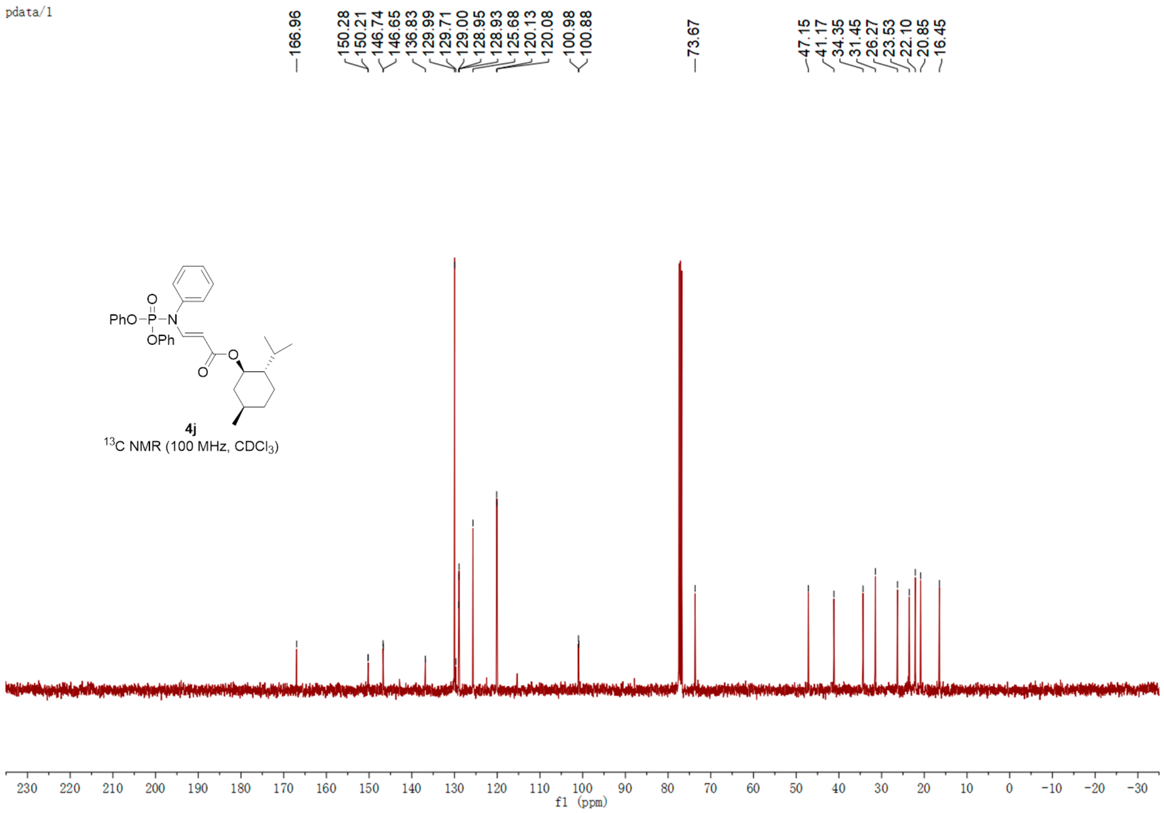

pdata/1
166.96
150.28
150.21
146.74
146.65
136.83
129.99
129.71
129.00
128.95
128.93
125.68
120.13
120.08
100.98
100.88
73.67
47.15
41.17
34.35
31.45
26.27
23.53
22.10
20.85
16.45
PhO
P
O
N
OPh
O
O
4j
13C NMR (100 MHz, CDCl3)
230 220 210 200 190 180 170 160 150 140 130 120 110 100 90 80 70 60 50 40 30 20 10 0 -10 -20 -30
f1 (ppm)

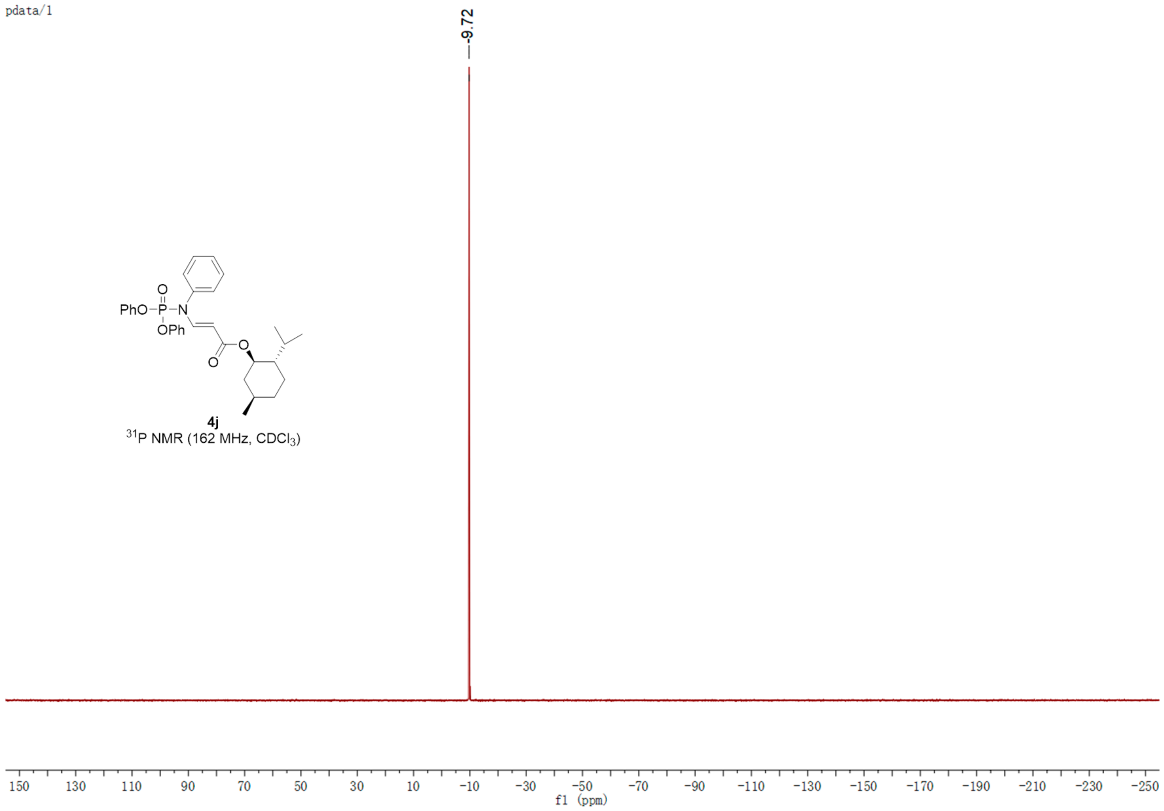

pdata/1
-9.72
PhO
P
O
N
OPh
O
O
4j
31P NMR (162 MHz, CDCl3)
150 130 110 90 70 50 30 10 -10 -30 -50 -70 -90 -110 -130 -150 -170 -190 -210 -230 -250
f1 (ppm)

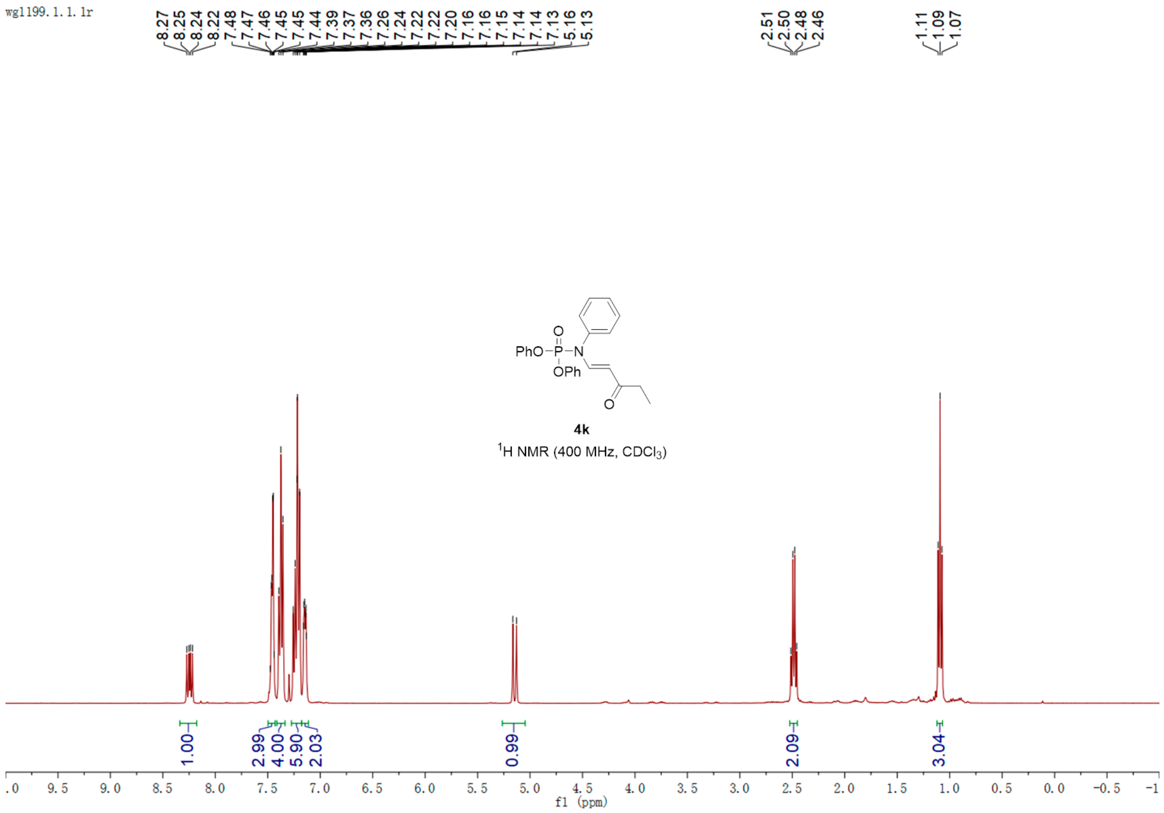

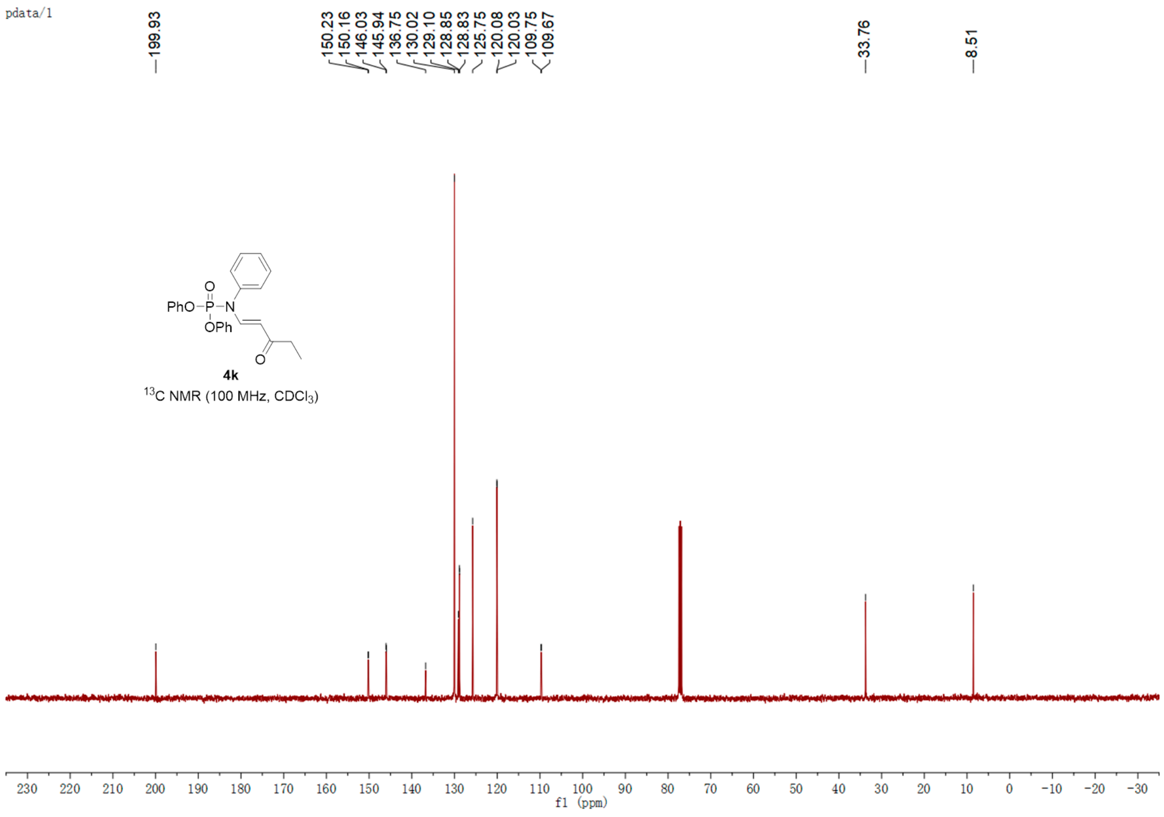

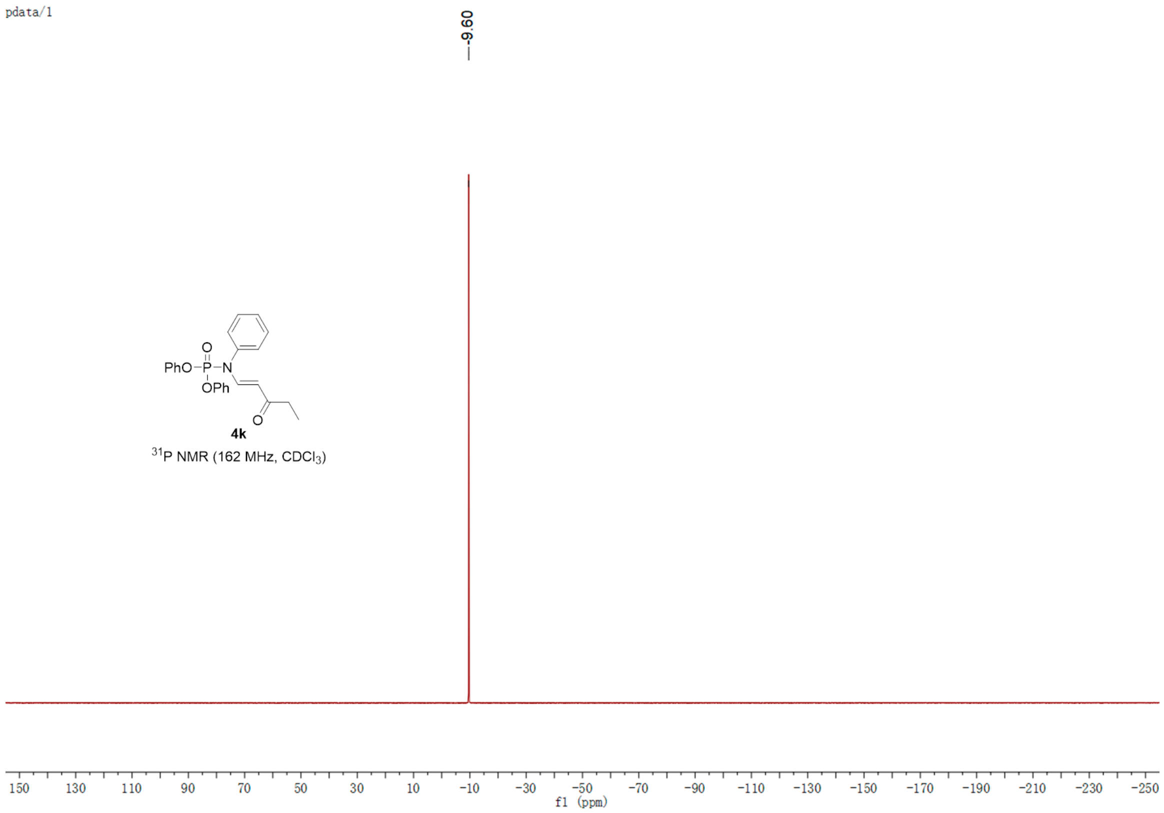
pdata/1
—9.60
PhO–P–N
OPh
O
4k
^{31}P NMR (162 MHz, CDCl$_3$)
150 130 110 90 70 50 30 10 -10 -30 -50 -70 -90 -110 -130 -150 -170 -190 -210 -230 -250
f1 (ppm)

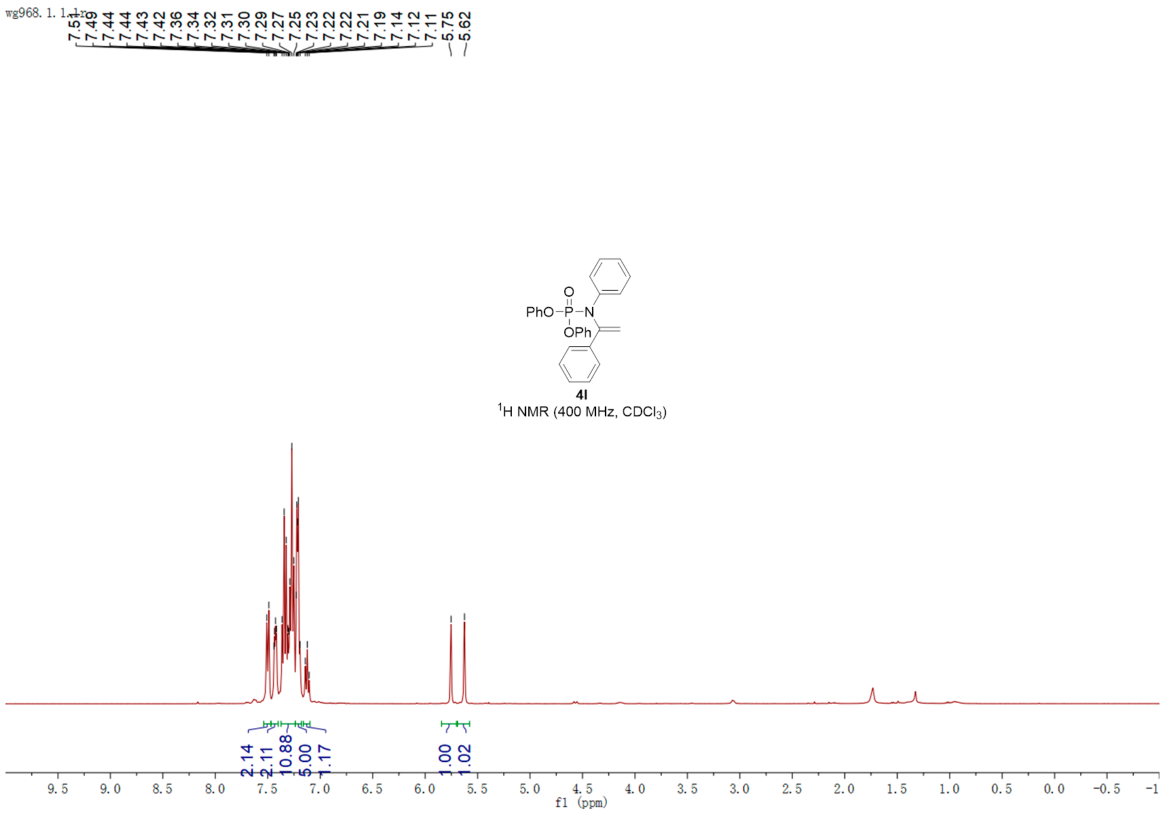
wg968.1.1.1r
7.51 7.49 7.44 7.44 7.43 7.42 7.36 7.34 7.32 7.31 7.30 7.29 7.27 7.25 7.23 7.22 7.22 7.21 7.19 7.14 7.12 7.11 5.75 5.62
PhO–P–N
OPh
4l
^{1}H NMR (400 MHz, CDCl$_3$)
2.14 2.11 10.88 5.00 1.17 1.00 1.02
9.5 9.0 8.5 8.0 7.5 7.0 6.5 6.0 5.5 5.0 4.5 4.0 3.5 3.0 2.5 2.0 1.5 1.0 0.5 0.0 -0.5 -1
f1 (ppm)

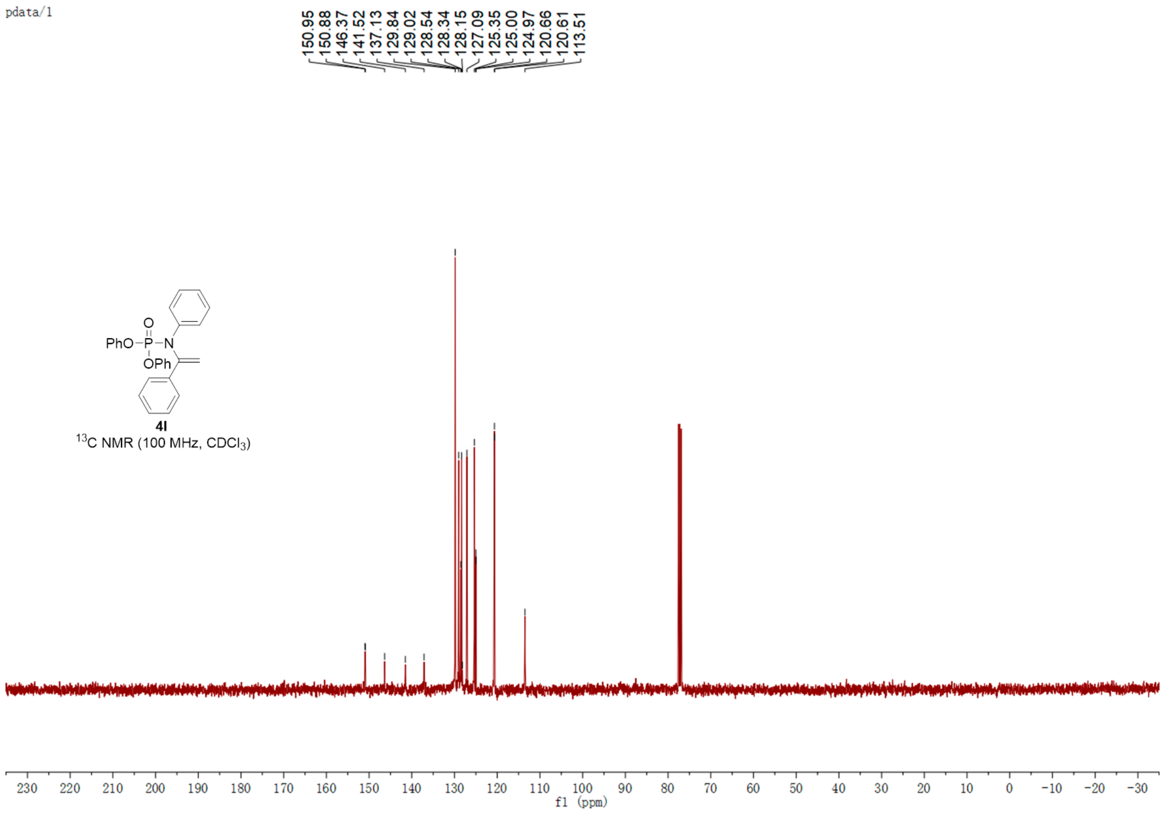
pdata/1
150.95
150.88
146.37
141.52
137.13
129.84
129.02
128.54
128.34
128.15
127.09
125.35
125.00
124.97
120.66
120.61
113.51
PhO
P
N
O
OPh
4l
13C NMR (100 MHz, CDCl3)
230 220 210 200 190 180 170 160 150 140 130 120 110 100 90 80 70 60 50 40 30 20 10 0 -10 -20 -30
f1 (ppm)

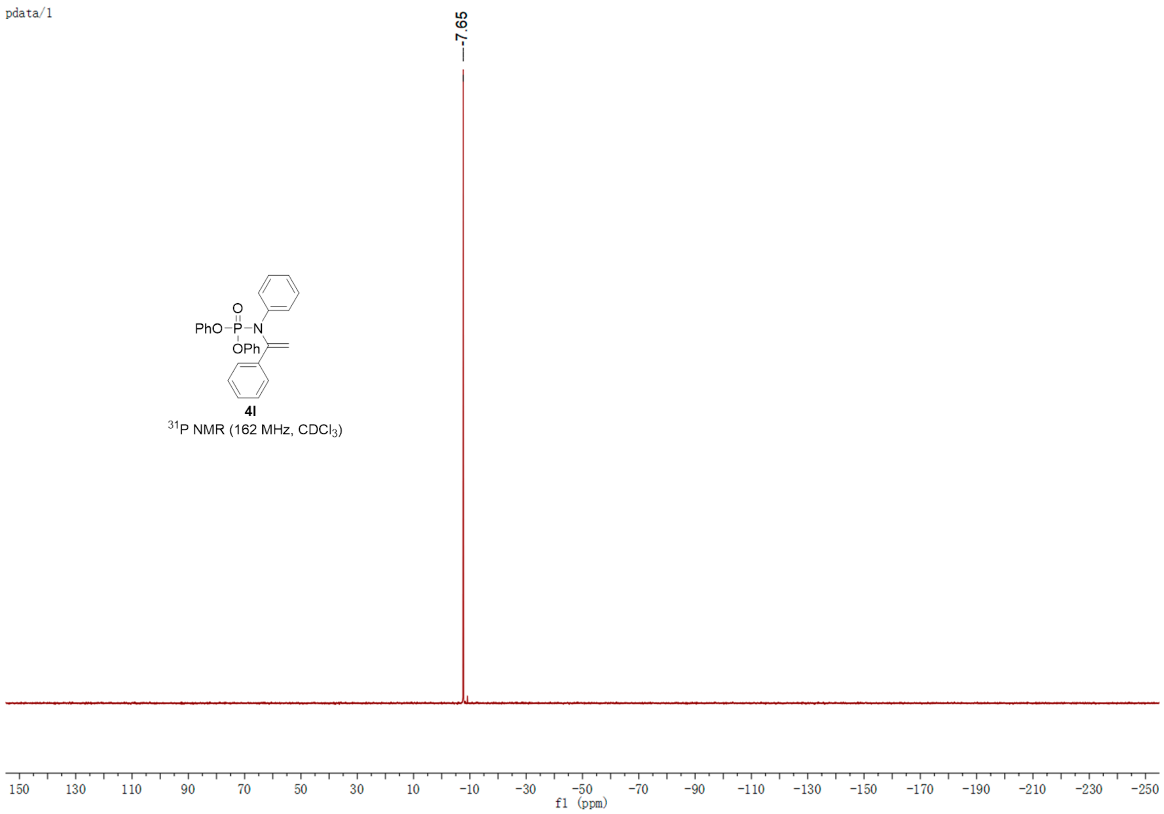
pdata/1
-7.65
PhO
P
N
O
OPh
4l
31P NMR (162 MHz, CDCl3)
150 130 110 90 70 50 30 10 -10 -30 -50 -70 -90 -110 -130 -150 -170 -190 -210 -230 -250
f1 (ppm)

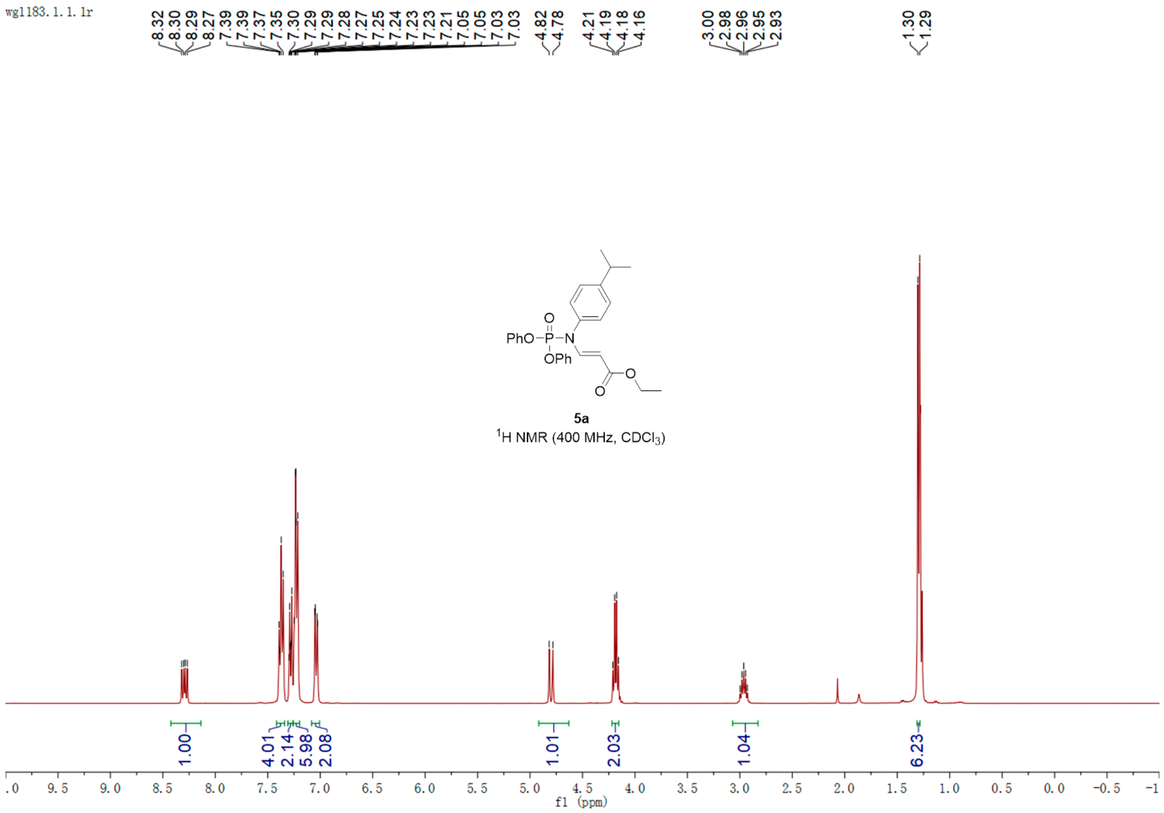
wg1183.1.1.1r
8.32
8.30
8.29
8.27
7.39
7.39
7.37
7.35
7.30
7.29
7.29
7.28
7.27
7.25
7.24
7.23
7.23
7.21
7.05
7.05
7.03
7.03
4.82
4.78
4.21
4.19
4.18
4.16
3.00
2.98
2.96
2.95
2.93
1.30
1.29
PhO
P
O
N
OPh
O
O
5a
1H NMR (400 MHz, CDCl3)
1.00
4.01
2.14
5.98
2.08
1.01
2.03
1.04
6.23
f1 (ppm)

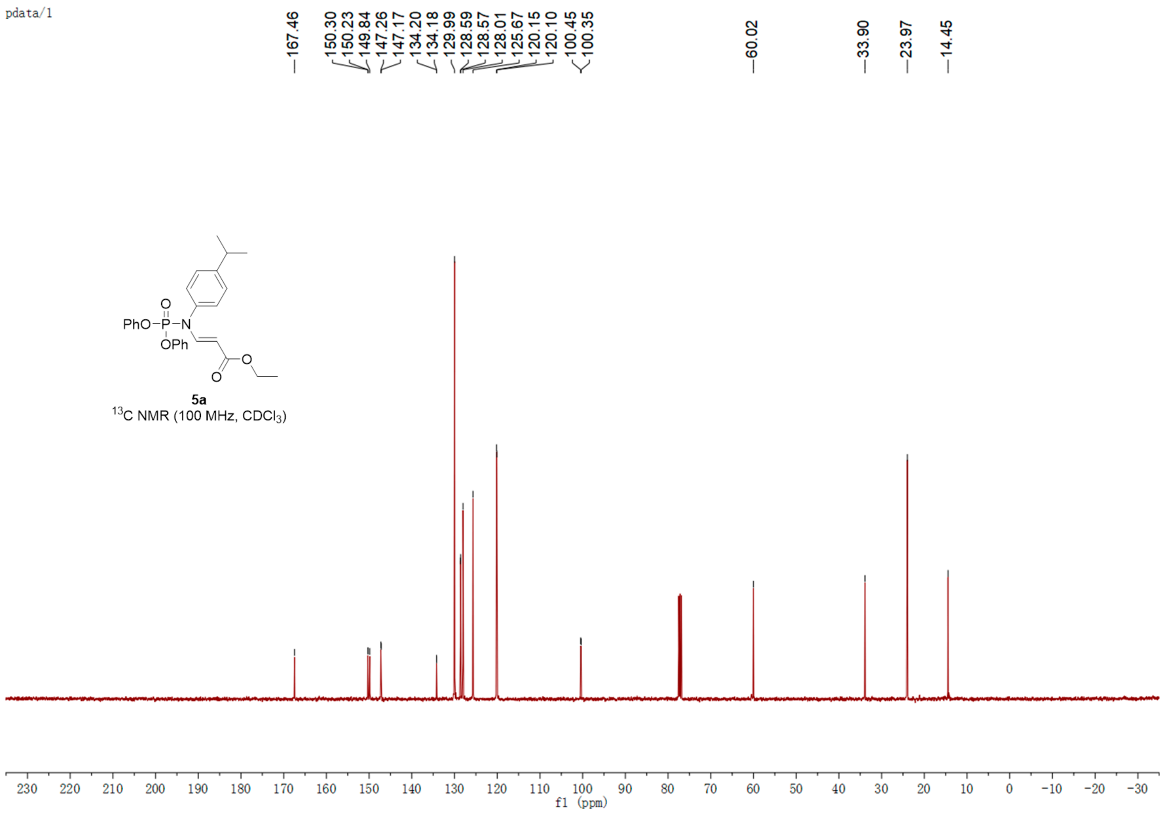
pdata/1
167.46
150.30
150.23
149.84
147.26
147.17
134.20
134.18
129.99
128.59
128.57
128.01
125.67
120.15
120.10
100.45
100.35
60.02
33.90
23.97
14.45
PhO
P
O
N
OPh
O
O
5a
13C NMR (100 MHz, CDCl3)
f1 (ppm)

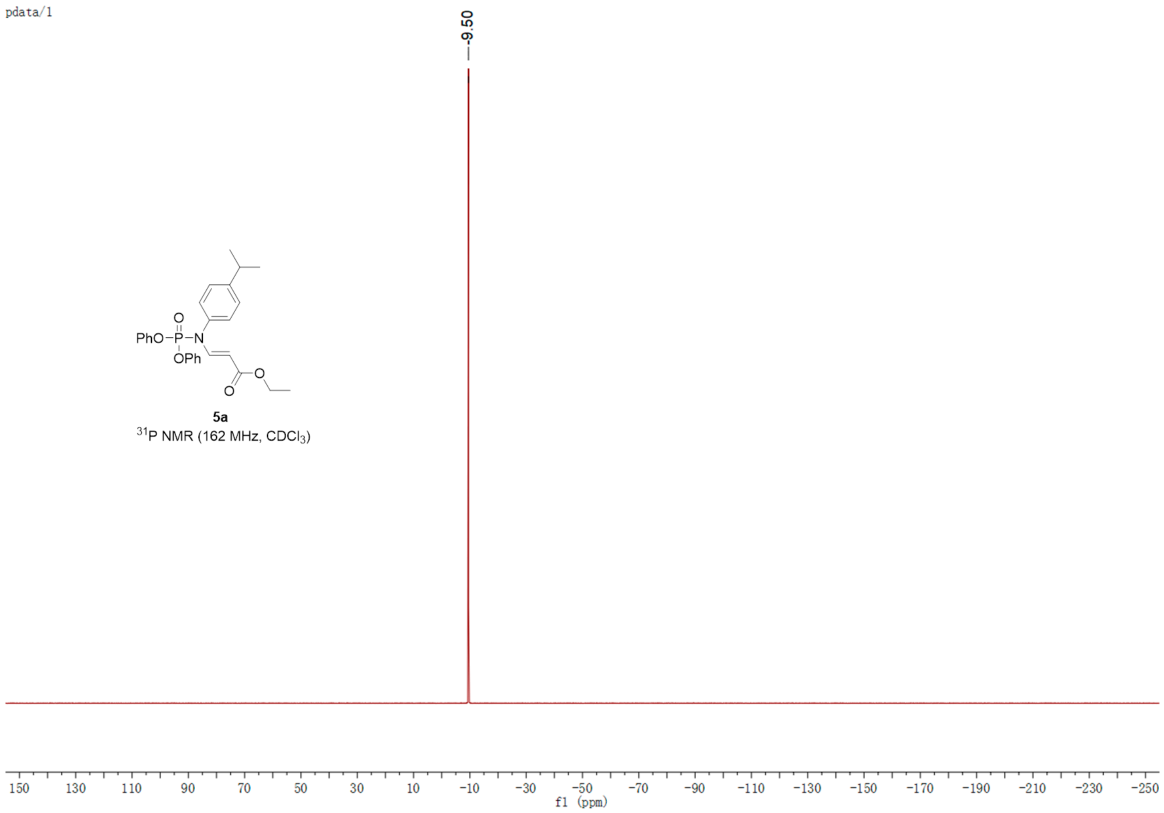
pdata/1
-9.50
PhO
P
N
O
OPh
O
O
5a
31P NMR (162 MHz, CDCl3)
f1 (ppm)

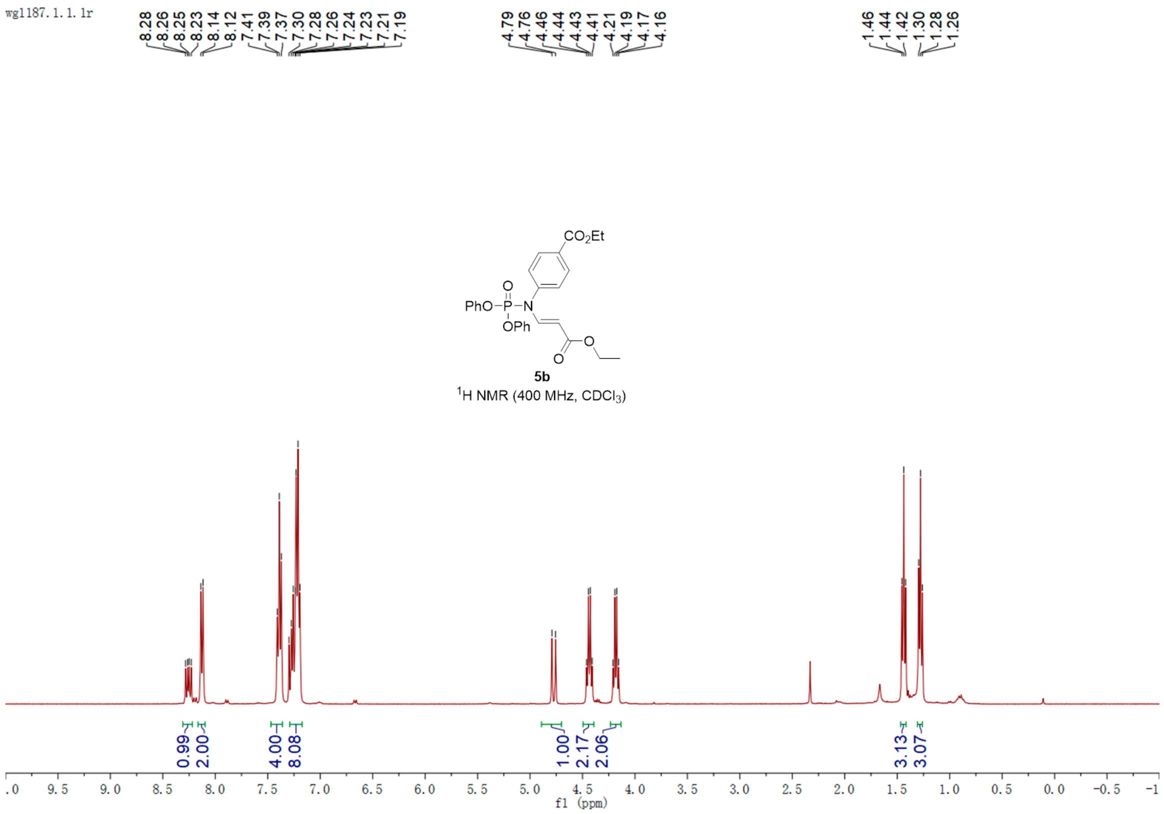
wg1187.1.1.1r
8.28
8.26
8.25
8.23
8.14
8.12
7.41
7.39
7.37
7.30
7.28
7.26
7.24
7.23
7.21
7.19
4.79
4.76
4.46
4.44
4.43
4.41
4.21
4.19
4.17
4.16
1.46
1.44
1.42
1.30
1.28
1.26
CO2Et
PhO
P
N
O
OPh
O
O
5b
1H NMR (400 MHz, CDCl3)
0.99
2.00
4.00
8.08
1.00
2.17
2.06
3.13
3.07
f1 (ppm)

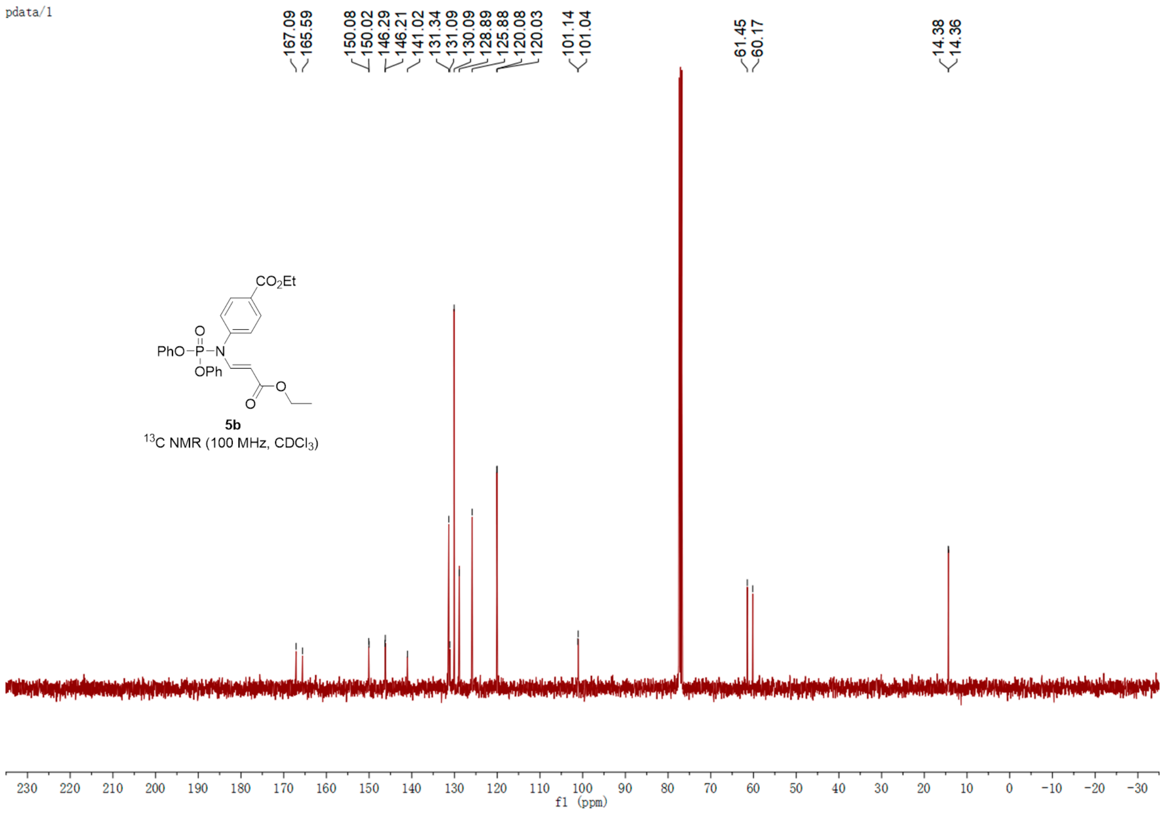
pdata/1
167.09
165.59
150.08
150.02
146.29
146.21
141.02
131.34
131.09
130.09
128.89
125.88
120.08
120.03
101.14
101.04
61.45
60.17
14.38
14.36
CO2Et
PhO
P
N
O
OPh
O
O
5b
13C NMR (100 MHz, CDCl3)
f1 (ppm)

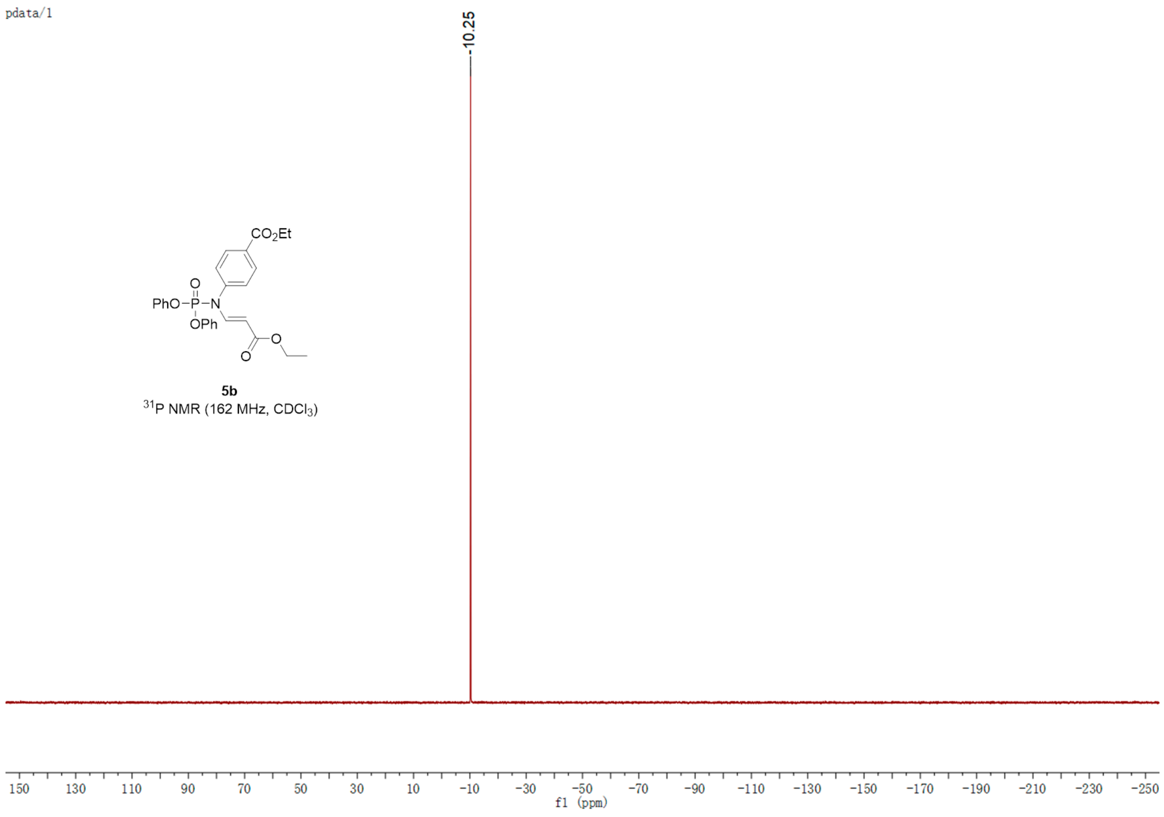
pdata/1
-10.25
CO2Et
PhO
P
N
O
OPh
O
O
5b
31P NMR (162 MHz, CDCl3)
f1 (ppm)

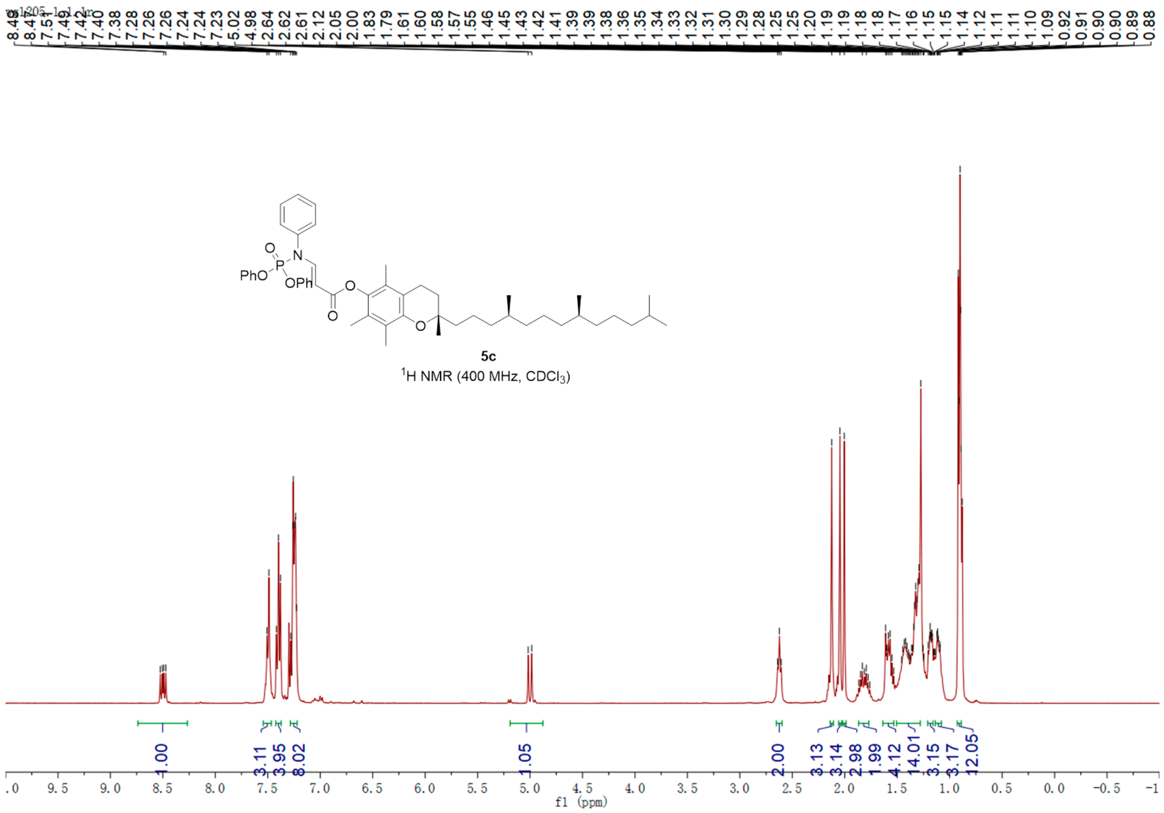

5c
^{1}H NMR (400 MHz, CDCl$_3$)
f1 (ppm)

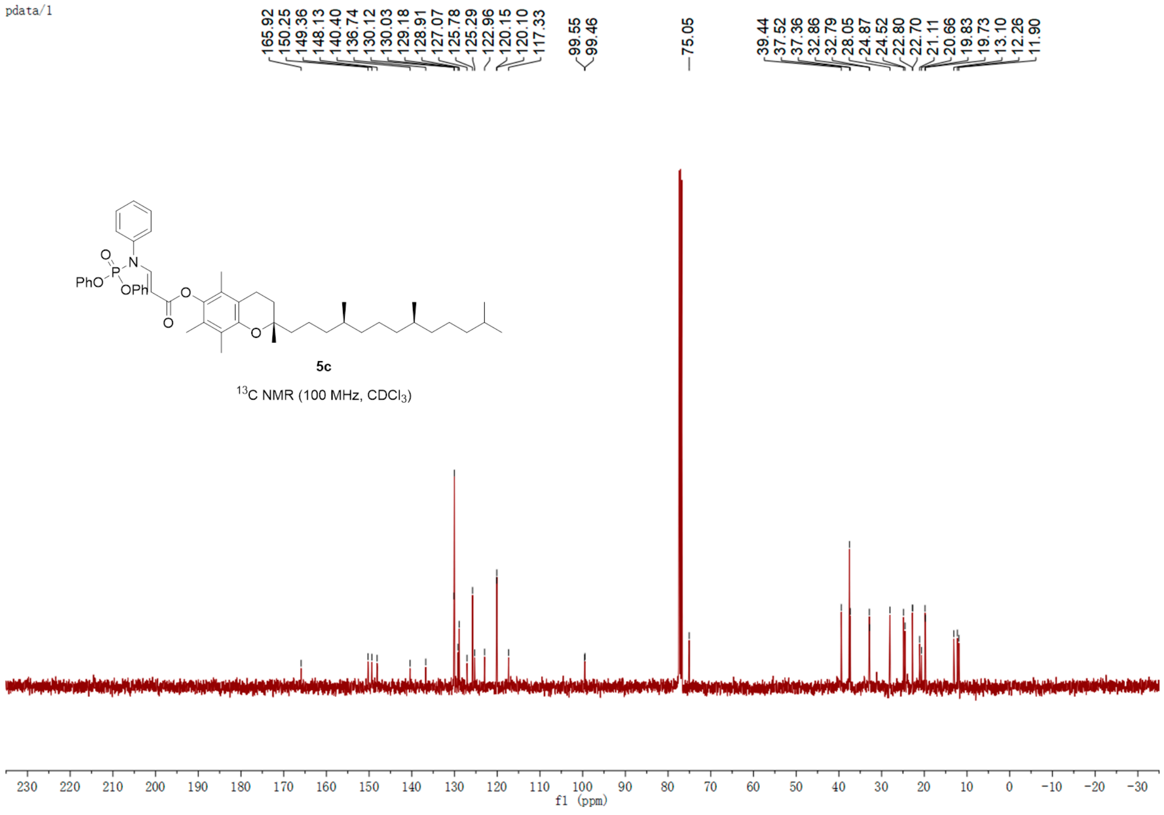

pdata/1
5c
^{13}C NMR (100 MHz, CDCl$_3$)
f1 (ppm)

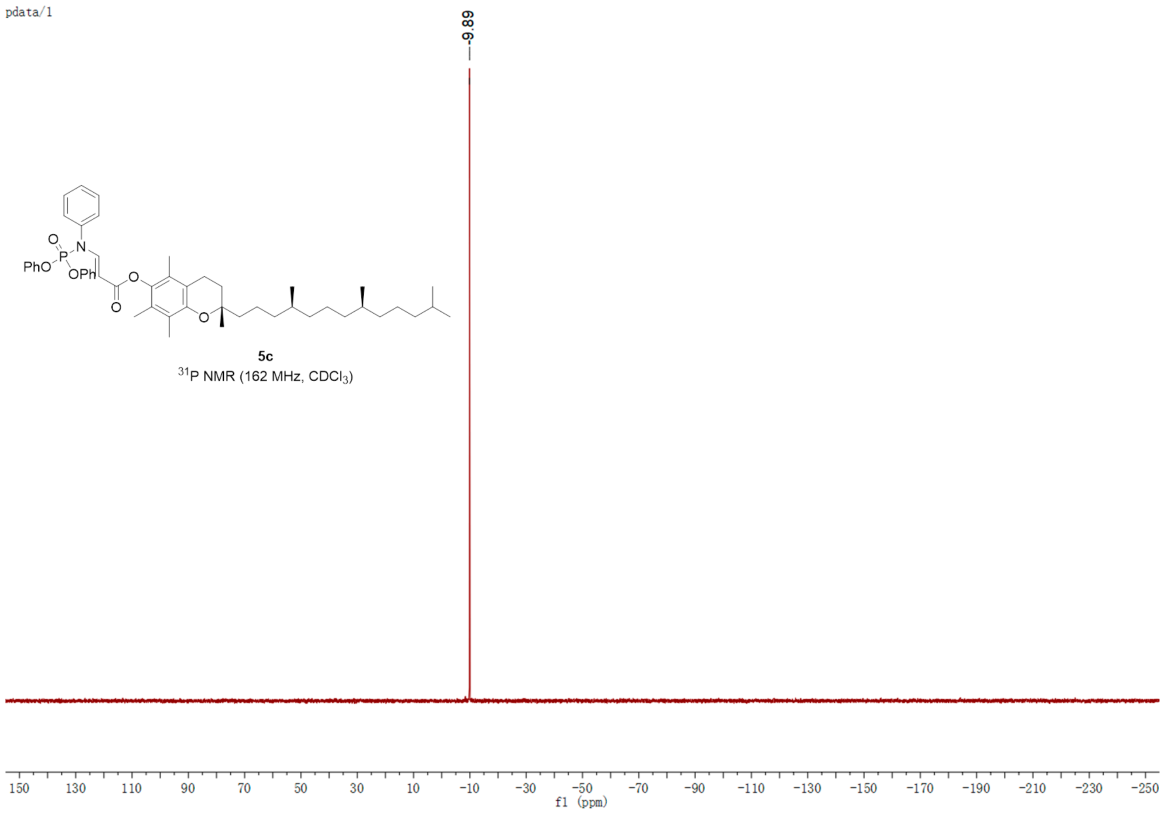

5c

^{31}P NMR (162 MHz, $CDCl_3$)

HRMS spectra of products

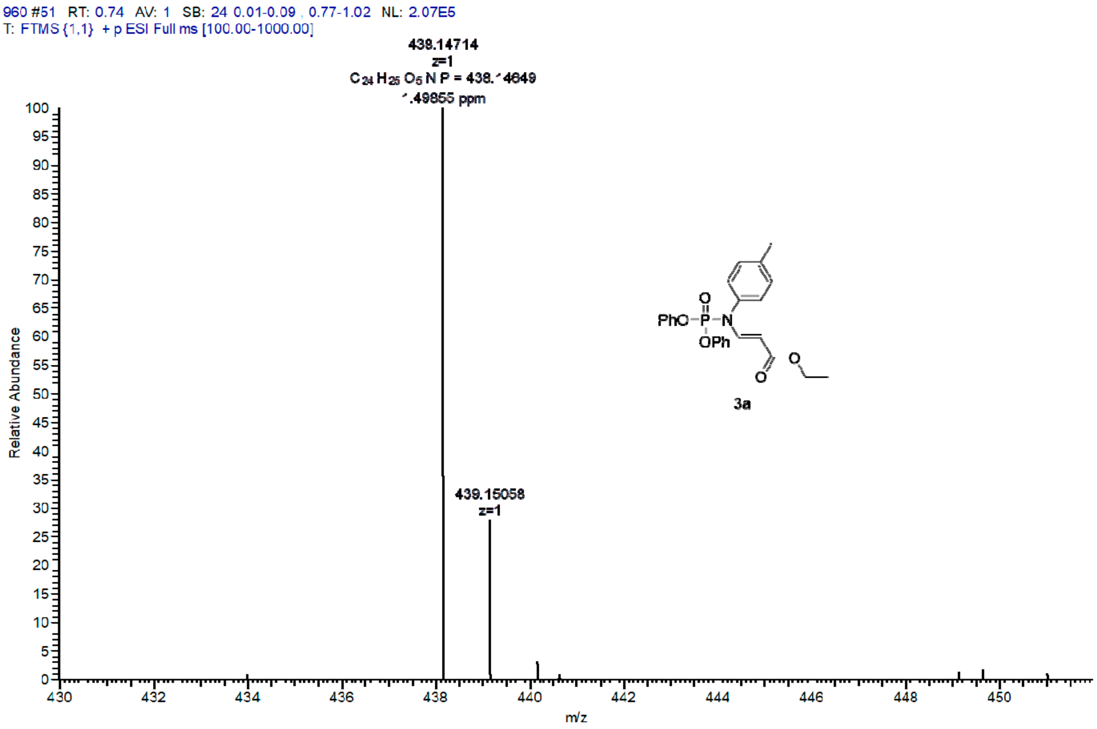

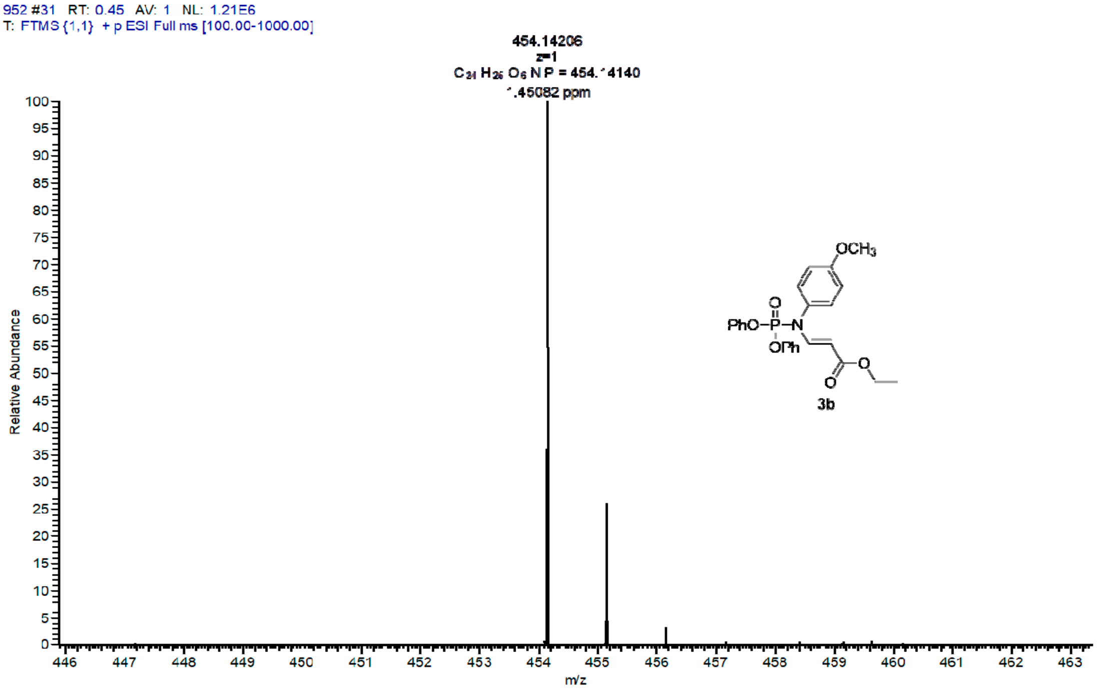

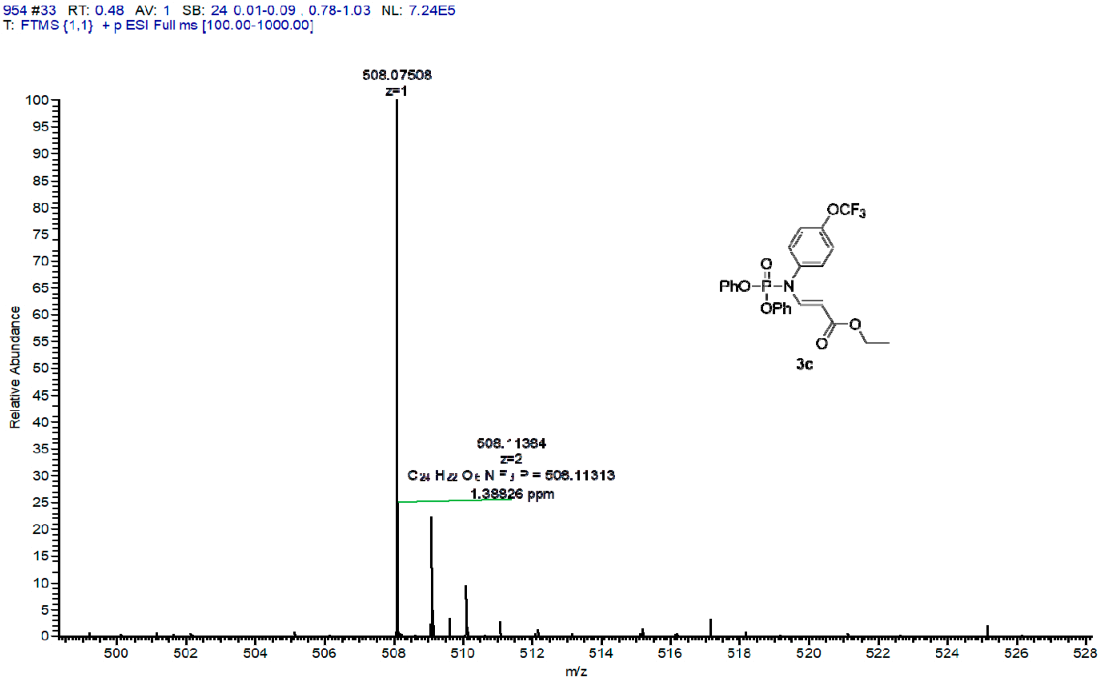
954 #33 RT: 0.48 AV: 1 SB: 24 0.01-0.09 , 0.78-1.03 NL: 7.24E5
T: FTMS {1,1} + p ESI Full ms [100.00-1000.00]
508.07508
z=1
508.11384
z=2
1.38826 ppm
Relative Abundance
m/z
OCF3
PhO
OPh
3c

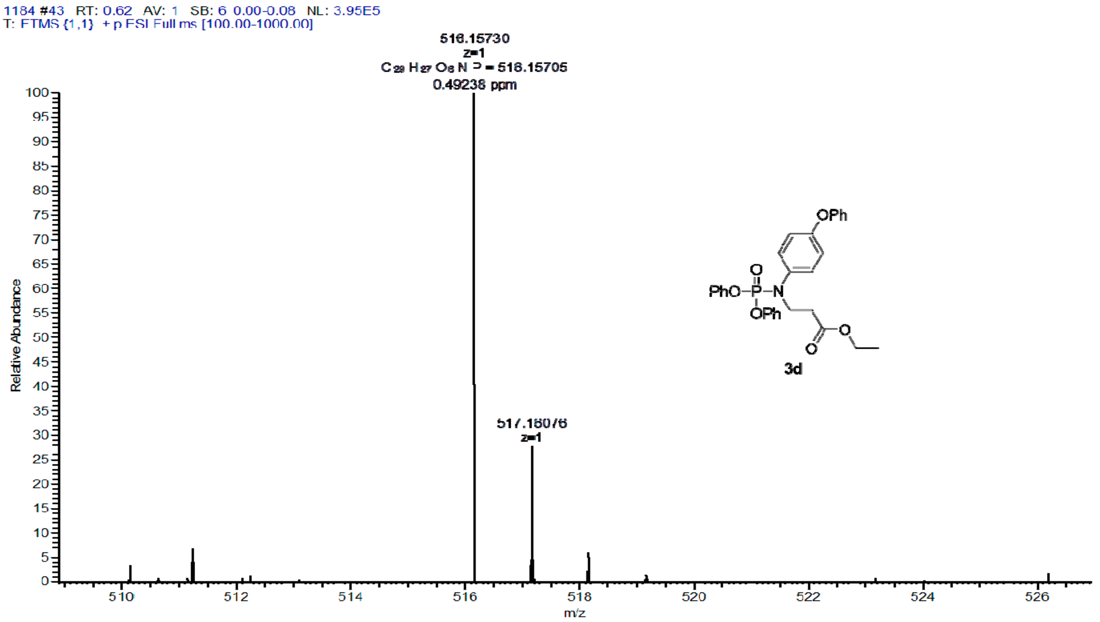
1184 #43 RT: 0.62 AV: 1 SB: 6 0.00-0.08 NL: 3.95E5
T: FTMS {1,1} + p ESI Full ms [100.00-1000.00]
516.15730
z=1
0.49238 ppm
517.16076
z=1
Relative Abundance
m/z
OPh
PhO
OPh
3d

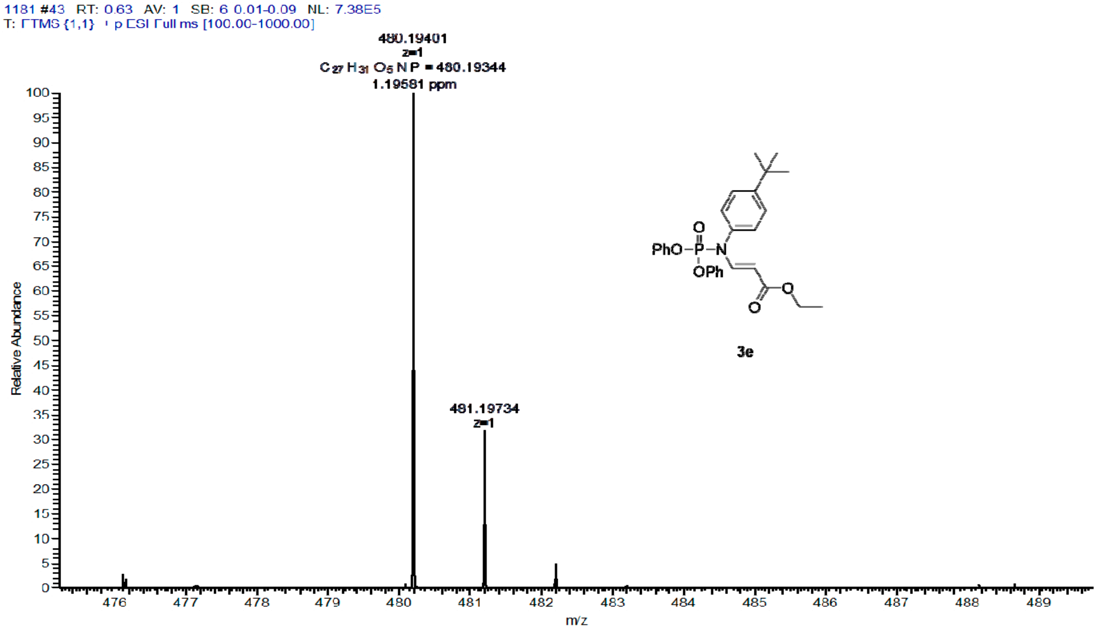
1181 #43 RT: 0.63 AV: 1 SB: 6 0.01-0.09 NL: 7.38E5
T: FTMS {1,1} + p ESI Full ms [100.00-1000.00]
480.19401
z=1
C27 H31 O5 N P = 480.19344
1.19581 ppm
481.19734
z=1
Relative Abundance
m/z
PhO
P
N
O
OPh
O
O
3e

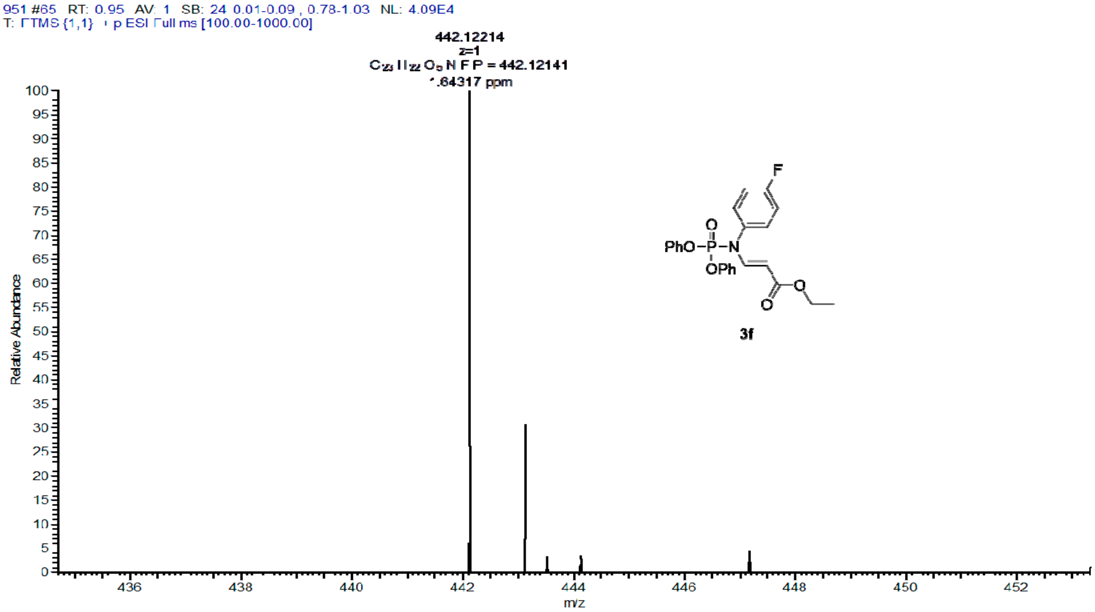
951 #65 RT: 0.95 AV: 1 SB: 24 0.01-0.09 , 0.73-1.03 NL: 4.09E4
T: FTMS {1,1} + p ESI Full ms [100.00-1000.00]
442.12214
z=1
C23 H22 O5 N F P = 442.12141
1.64317 ppm
Relative Abundance
m/z
F
PhO
P
N
O
OPh
O
O
3f

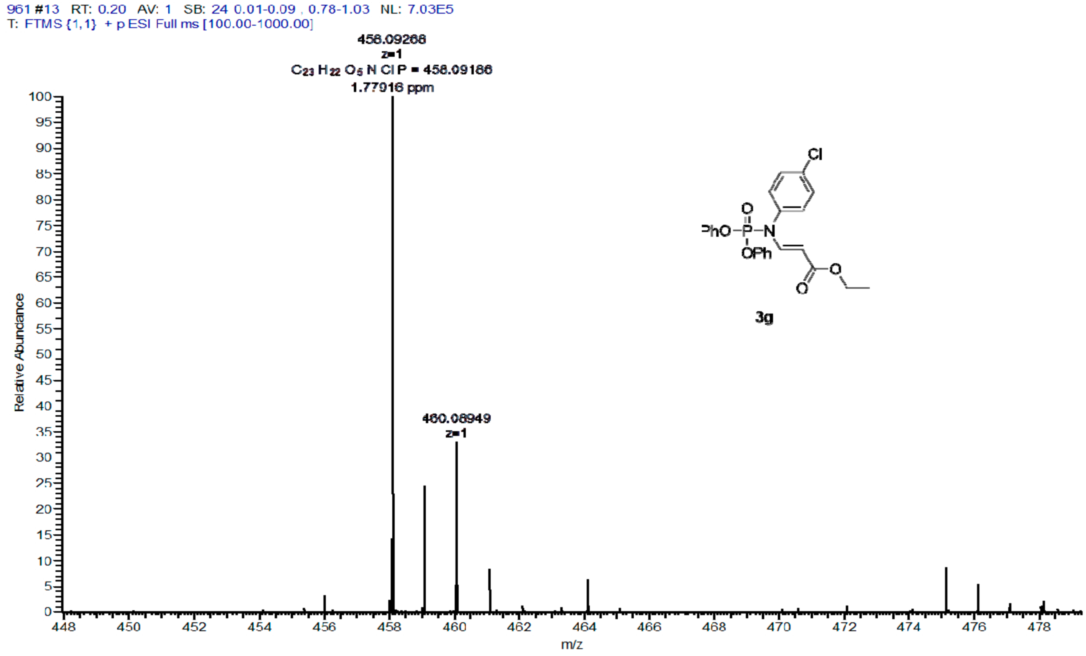
961 #13 RT: 0.20 AV: 1 SB: 24 0.01-0.09 , 0.78-1.03 NL: 7.03E5
T: FTMS {1,1} + p ESI Full ms [100.00-1000.00]
458.09266
z=1
C23 H22 O5 N Cl P = 458.09186
1.77916 ppm
460.08949
z=1
Relative Abundance
m/z
Cl
O
PhO
P
N
OPh
O
O
3g

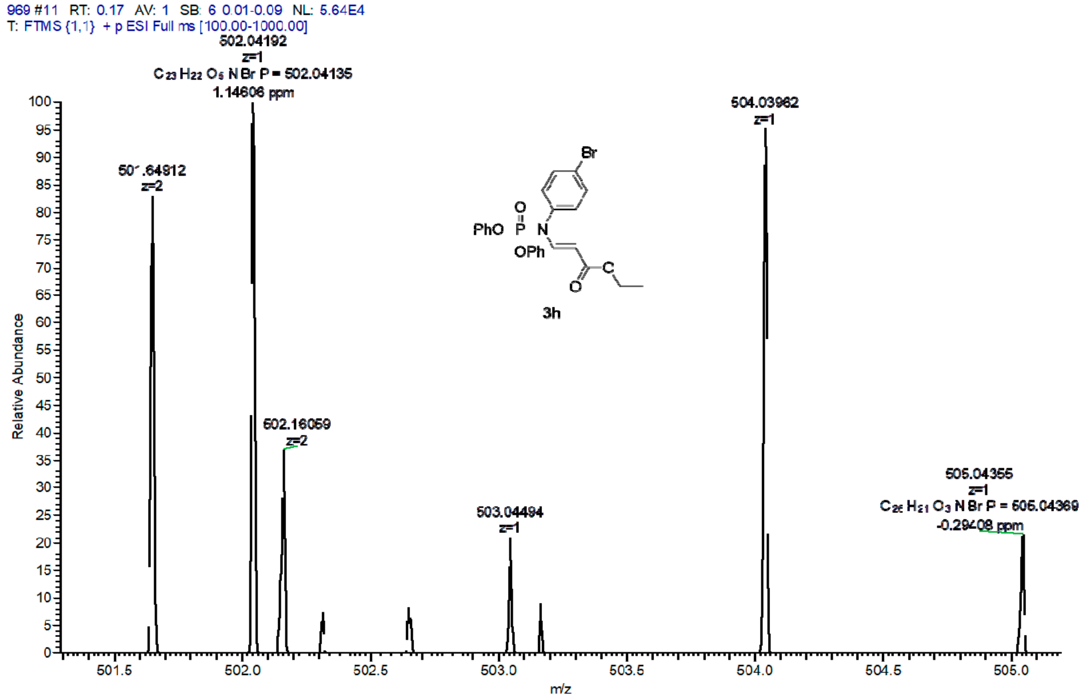
969 #11 RT: 0.17 AV: 1 SB: 6 0.01-0.09 NL: 5.64E4
T: FTMS {1,1} + p ESI Full ms [100.00-1000.00]
502.04192
z=1
C23 H22 O5 N Br P = 502.04135
1.14606 ppm
504.03962
z=1
50´.64912
z=2
502.16059
z=2
503.04494
z=1
505.04355
z=1
C26 H21 O3 N Br P = 505.04369
-0.29408 ppm
Relative Abundance
m/z
Br
O
PhO
P
N
OPh
O
C
3h

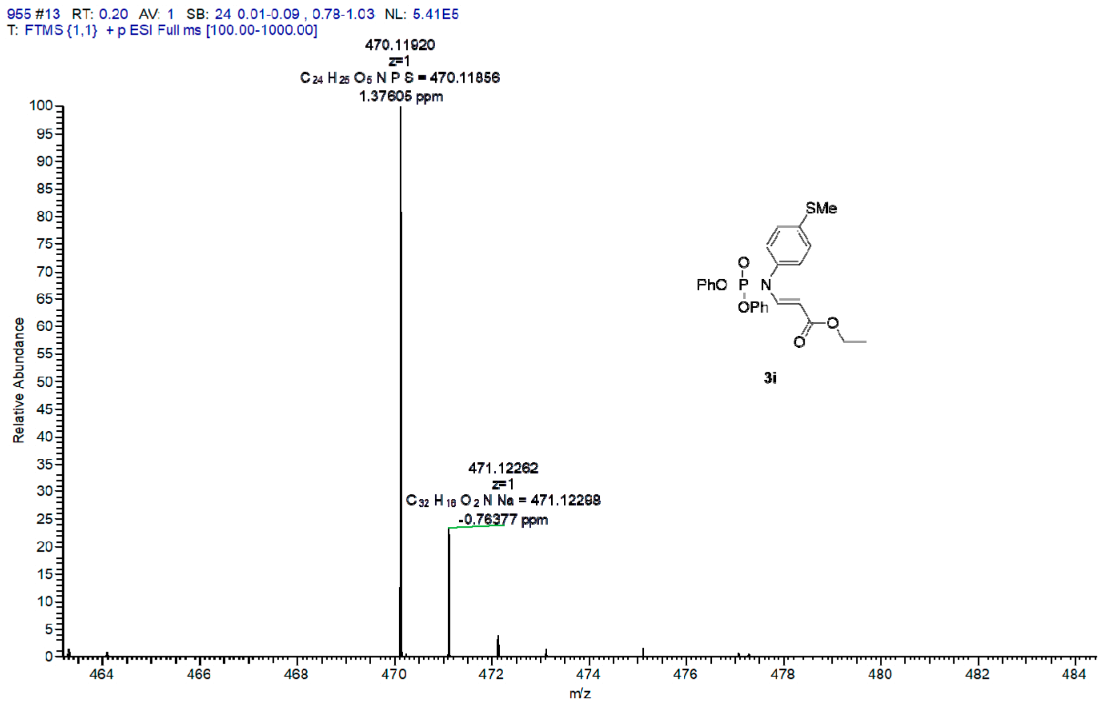
955 #13 RT: 0.20 AV: 1 SB: 24 0.01-0.09 , 0.78-1.03 NL: 5.41E5
T: FTMS {1,1} + p ESI Full ms [100.00-1000.00]
470.11920
z=1
C24 H25 O5 N P S = 470.11856
1.37605 ppm
471.12262
z=1
C32 H18 O2 N Na = 471.12298
-0.76377 ppm
Relative Abundance
m/z
SMe
PhO
OPh
3i

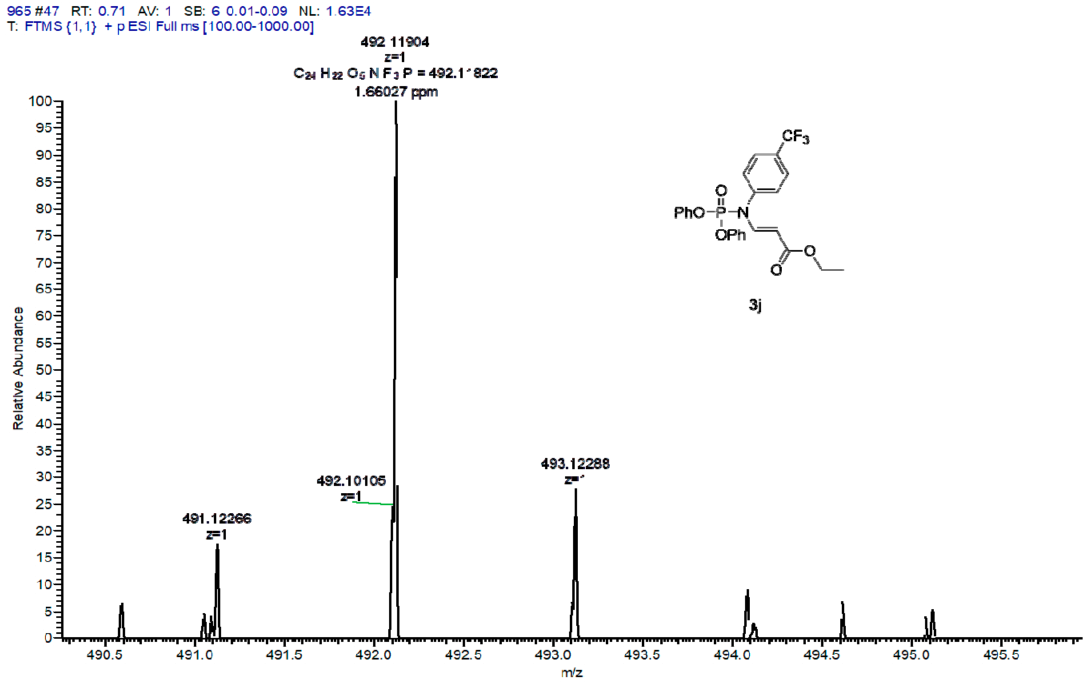
965 #47 RT: 0.71 AV: 1 SB: 6 0.01-0.09 NL: 1.63E4
T: FTMS {1,1} + p ESI Full ms [100.00-1000.00]
492.11904
z=1
1.66027 ppm
491.12266
z=1
492.10105
z=1
493.12288
Relative Abundance
m/z
CF3
PhO
OPh
3j

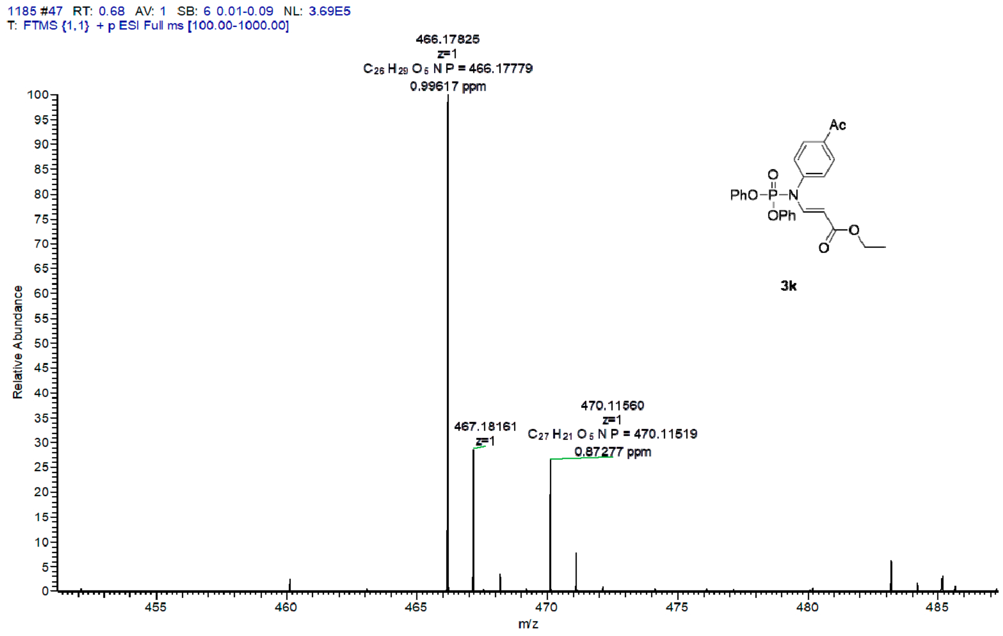
1185 #47 RT: 0.68 AV: 1 SB: 6 0.01-0.09 NL: 3.69E5
T: FTMS {1,1} + p ESI Full ms [100.00-1000.00]
466.17825
z=1
C26 H29 O5 N P = 466.17779
0.99617 ppm
467.18161
z=1
470.11560
z=1
C27 H21 O5 N P = 470.11519
0.87277 ppm
Relative Abundance
m/z
Ac
PhO
OPh
3k

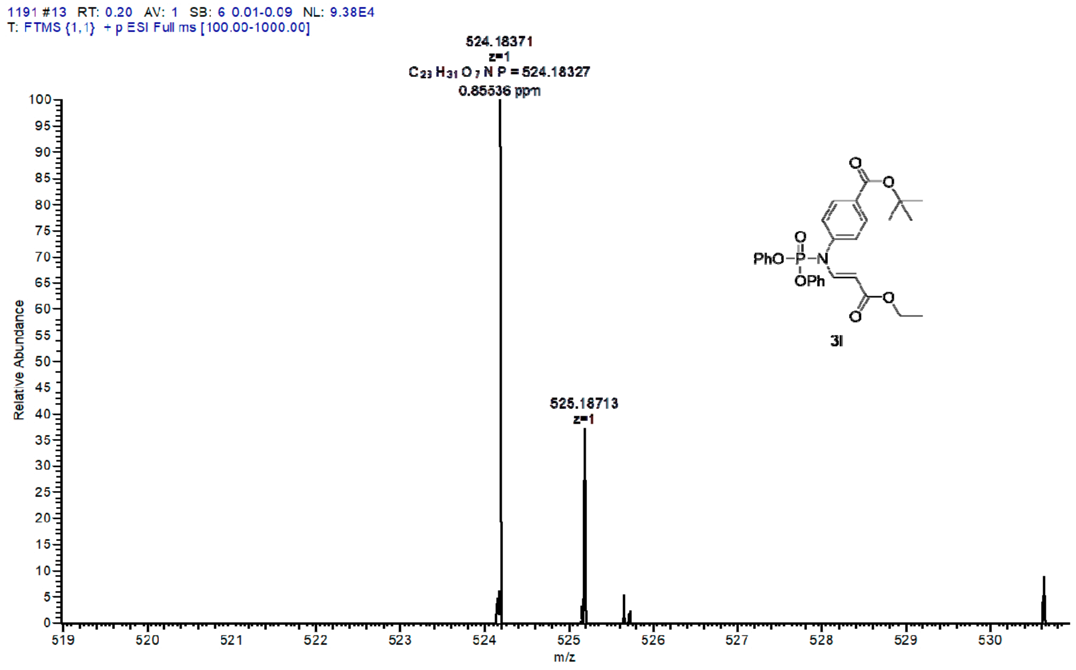
1191 #13 RT: 0.20 AV: 1 SB: 6 0.01-0.09 NL: 9.38E4
T: FTMS {1,1} + p ESI Full ms [100.00-1000.00]
524.18371
z=1
C23 H31 O7 N P = 524.18327
0.85536 ppm
525.18713
z=1
Relative Abundance
m/z
PhO
OPh
3l

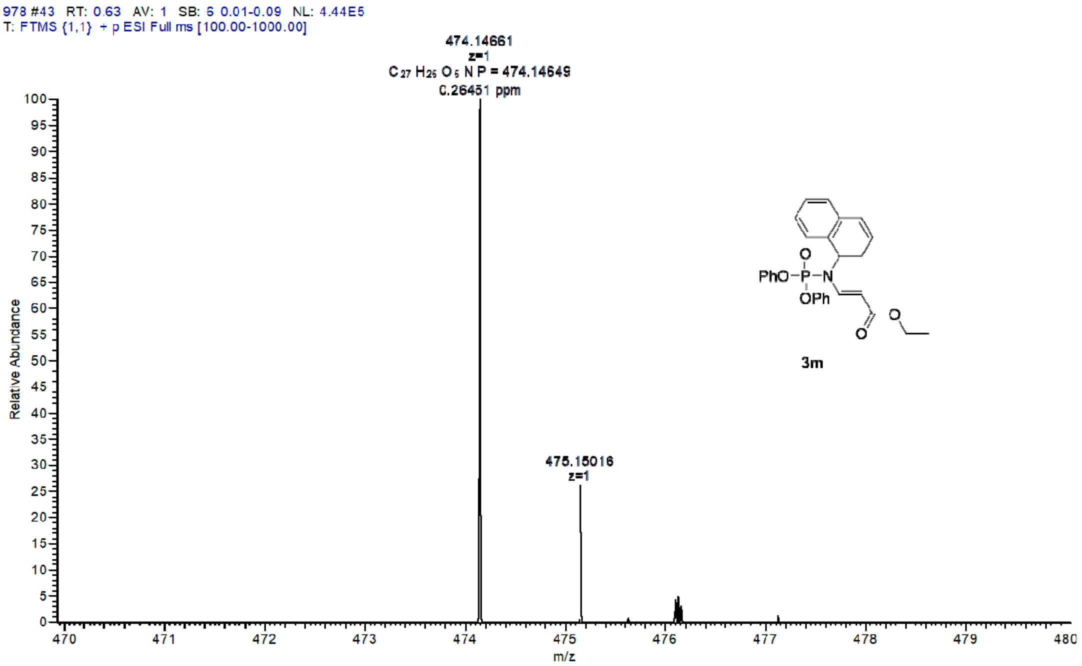
978 #43 RT: 0.63 AV: 1 SB: 6 0.01-0.09 NL: 4.44E5
T: FTMS {1,1} + p ESI Full ms [100.00-1000.00]
474.14661
z=1
C27 H25 O5 N P = 474.14649
0.26451 ppm
475.15016
z=1
Relative Abundance
m/z
PhO
OPh
3m

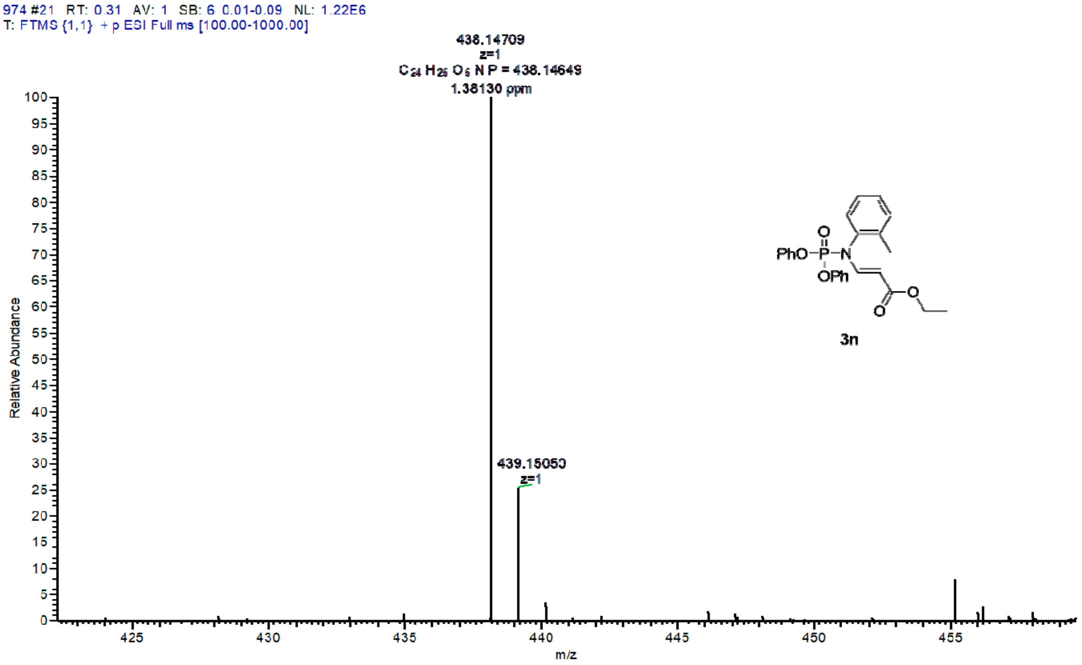
974 #21 RT: 0.31 AV: 1 SB: 6 0.01-0.09 NL: 1.22E6
T: FTMS {1,1} + p ESI Full ms [100.00-1000.00]
438.14709
z=1
C24 H25 O5 N P = 438.14649
1.38130 ppm
439.15050
z=1
Relative Abundance
m/z
PhO
OPh
3n

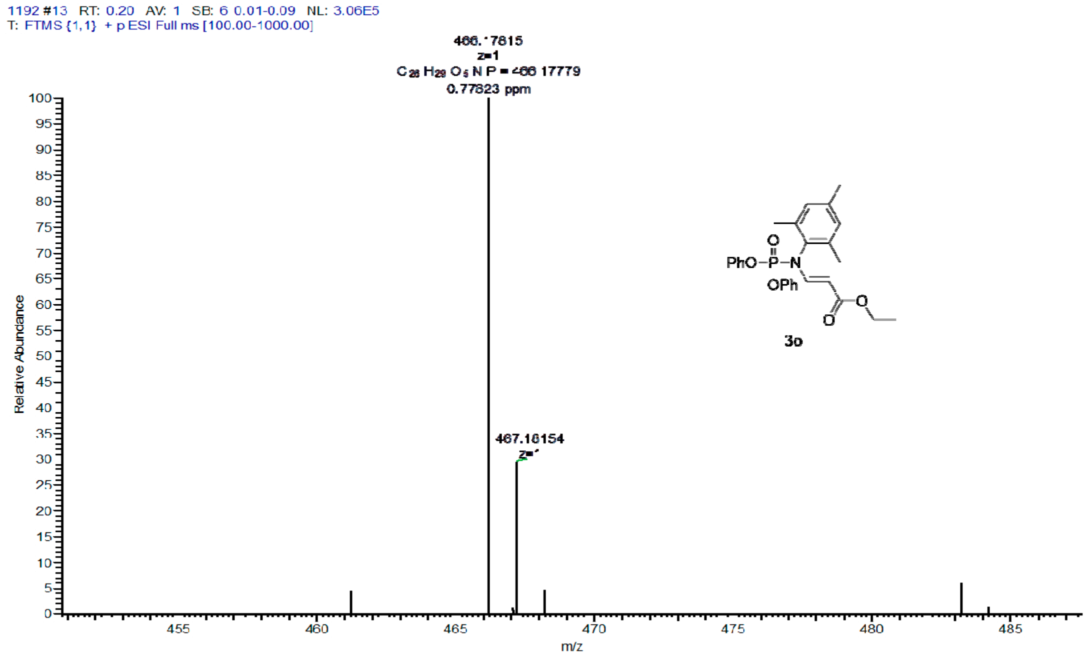
1192 #13 RT: 0.20 AV: 1 SB: 6 0.01-0.09 NL: 3.06E5
T: FTMS {1,1} + p ESI Full ms [100.00-1000.00]
466.·7815
z=1
C26 H29 O5 N P = 466 17779
0.77823 ppm
467.18154
z=·
Relative Abundance
m/z
PhO–P–N
OPh
3o

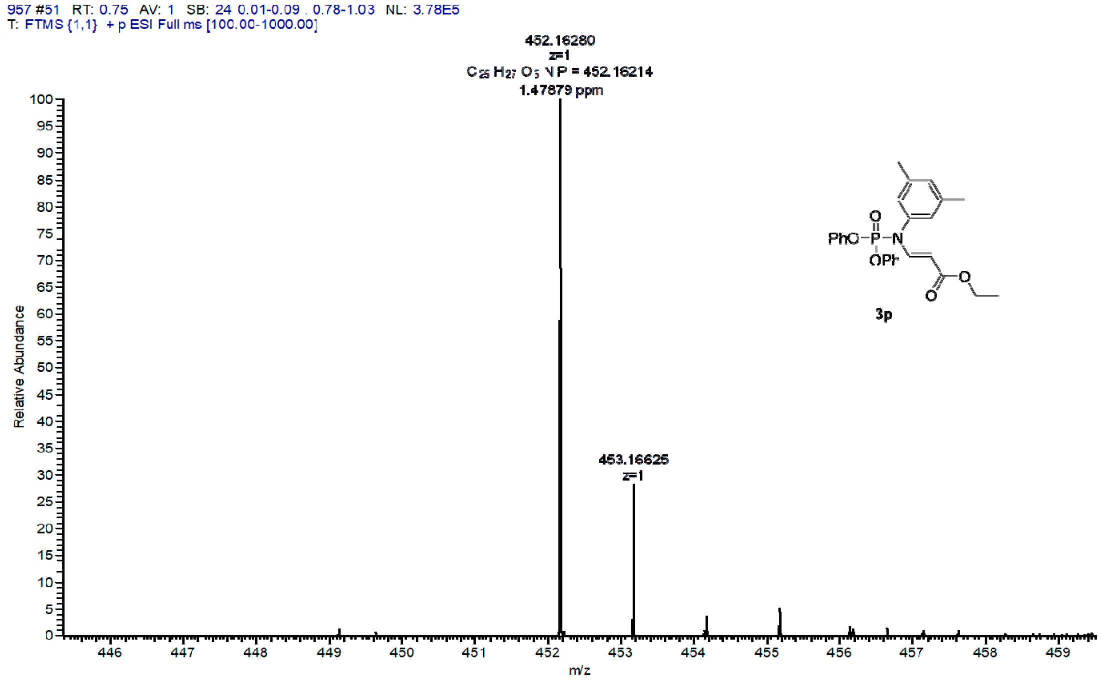
957 #51 RT: 0.75 AV: 1 SB: 24 0.01-0.09 , 0.78-1.03 NL: 3.78E5
T: FTMS {1,1} + p ESI Full ms [100.00-1000.00]
452.16280
z=1
C25 H27 O5 N P = 452.16214
1.47879 ppm
453.16625
z=1
Relative Abundance
m/z
PhO–P–N
OPh
3p

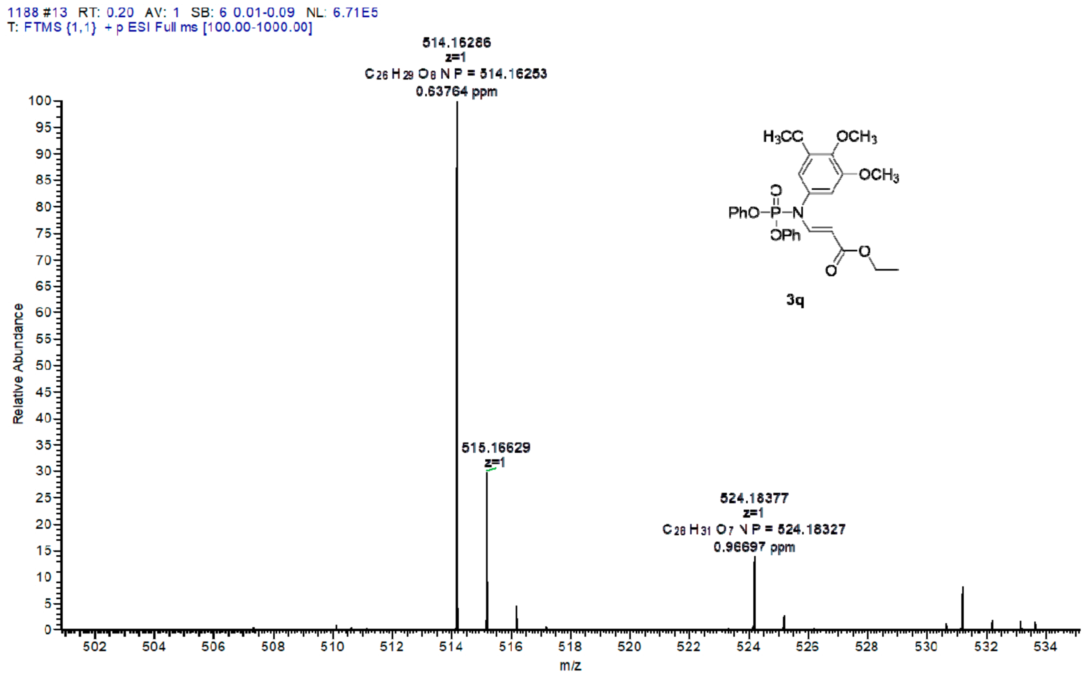

1188 #13 RT: 0.20 AV: 1 SB: 6 0.01-0.09 NL: 6.71E5
T: FTMS {1,1} + p ESI Full ms [100.00-1000.00]
514.16286
z=1
C26 H29 O8 N P = 514.16253
0.63764 ppm
515.16629
z=1
524.18377
z=1
C28 H31 O7 N P = 524.18327
0.96697 ppm
Relative Abundance
m/z
H3CO
OCH3
OCH3
PhO
P
N
OPh
O
3q

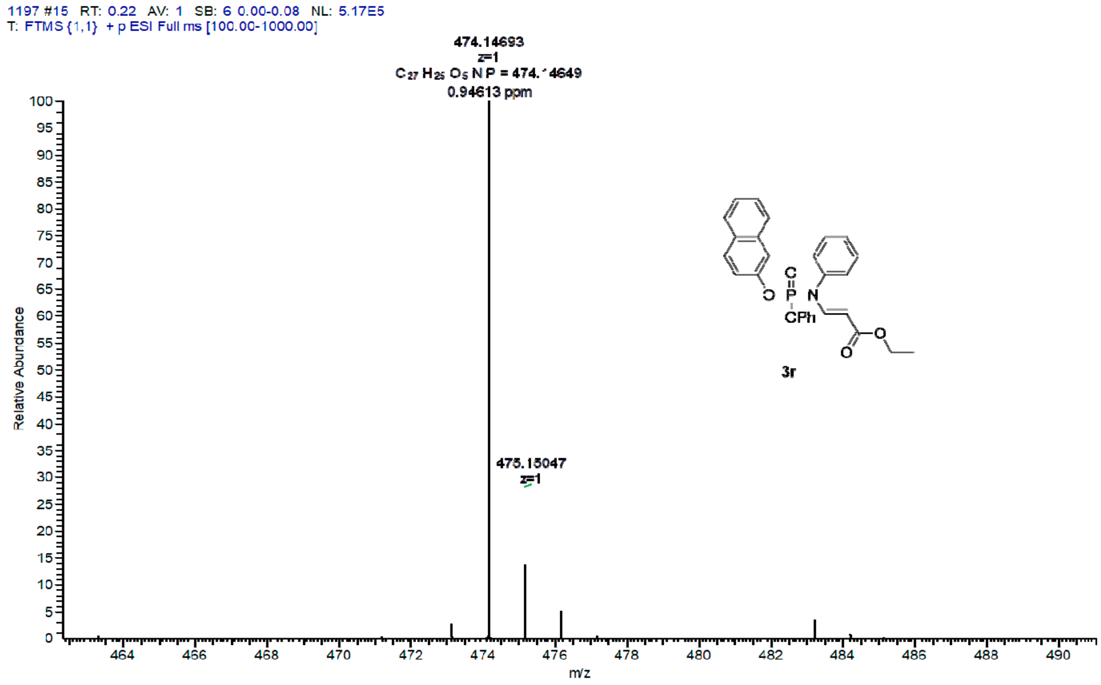

1197 #15 RT: 0.22 AV: 1 SB: 6 0.00-0.08 NL: 5.17E5
T: FTMS {1,1} + p ESI Full ms [100.00-1000.00]
474.14693
z=1
C27 H25 O5 N P = 474.14649
0.94613 ppm
475.15047
z=1
Relative Abundance
m/z
O
P
N
OPh
3r

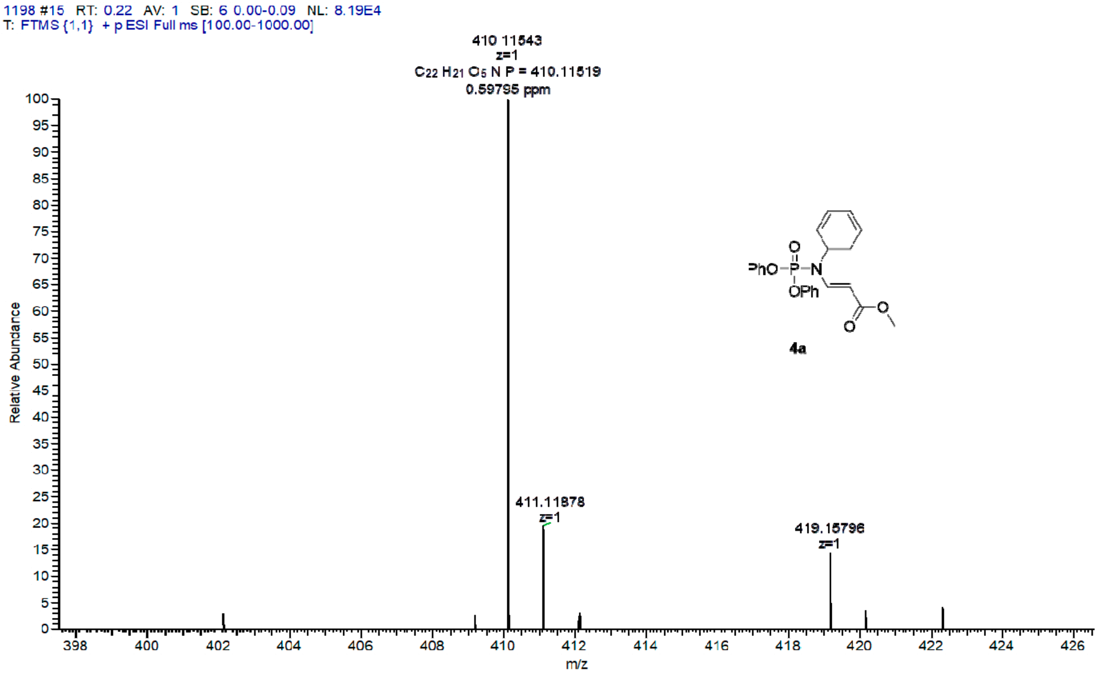
1198 #15 RT: 0.22 AV: 1 SB: 6 0.00-0.09 NL: 8.19E4
T: FTMS {1,1} + p ESI Full ms [100.00-1000.00]
410.11543
z=1
C22 H21 O5 N P = 410.11519
0.59795 ppm
411.11878
z=1
419.15796
z=1
PhO
OPh
4a
Relative Abundance
m/z

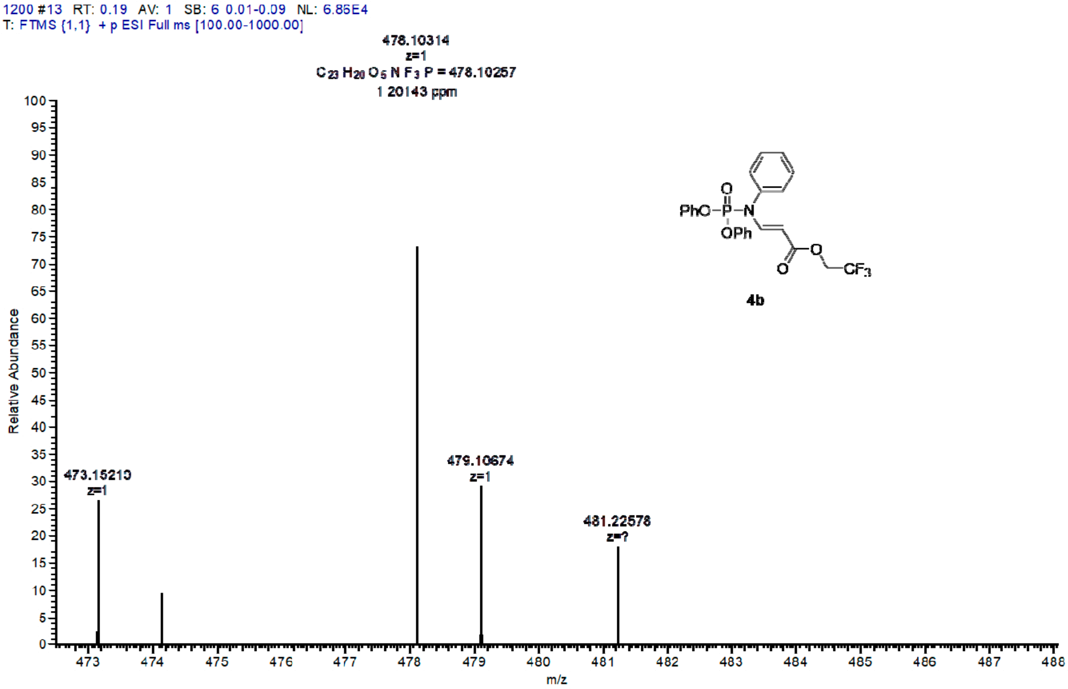
1200 #13 RT: 0.19 AV: 1 SB: 6 0.01-0.09 NL: 6.85E4
T: FTMS {1,1} + p ESI Full ms [100.00-1000.00]
478.10314
z=1
C23 H20 O5 N F3 P = 478.10257
1.20143 ppm
473.15210
z=1
479.10674
z=1
481.22578
z=?
PhO
OPh
CF3
4b
Relative Abundance
m/z

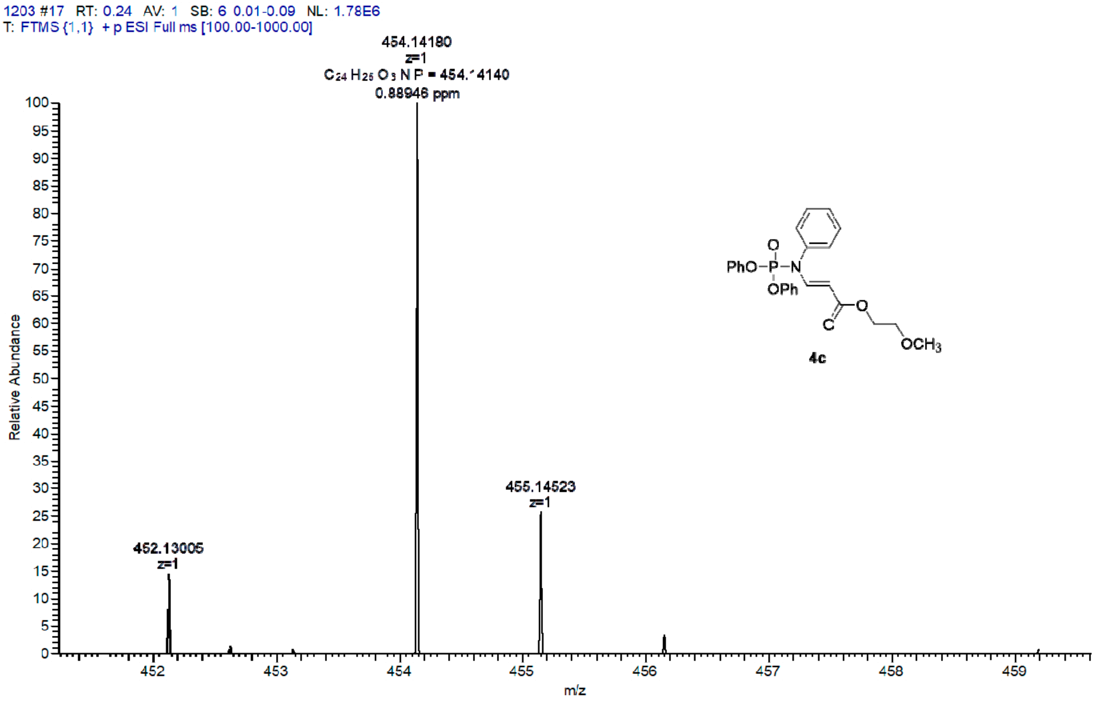

1203 #17 RT: 0.24 AV: 1 SB: 6 0.01-0.09 NL: 1.78E6
T: FTMS {1,1} + p ESI Full ms [100.00-1000.00]
454.14180
z=1
C24 H25 O5 N P = 454.14140
0.88946 ppm
455.14523
z=1
452.13005
z=1
Relative Abundance
m/z
PhO
P
N
OPh
O
OCH3
4c

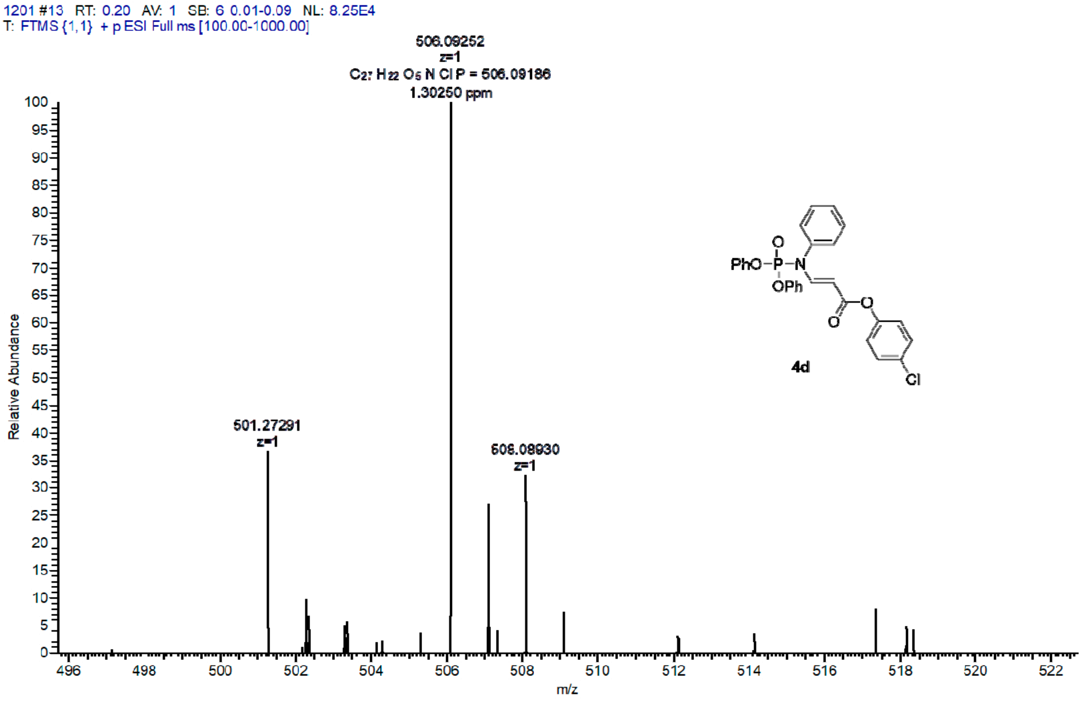

1201 #13 RT: 0.20 AV: 1 SB: 6 0.01-0.09 NL: 8.25E4
T: FTMS {1,1} + p ESI Full ms [100.00-1000.00]
506.09252
z=1
C27 H22 O5 N Cl P = 506.09186
1.30250 ppm
501.27291
z=1
508.08930
z=1
Relative Abundance
m/z
PhO
P
N
OPh
O
Cl
4d

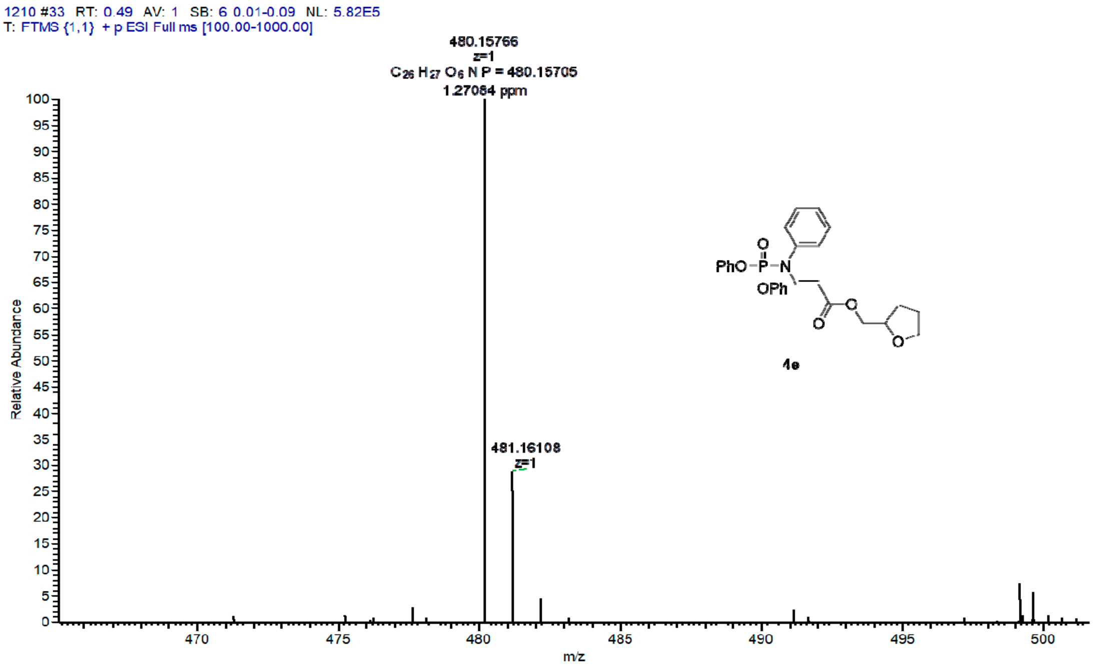
1210 #33 RT: 0.49 AV: 1 SB: 6 0.01-0.09 NL: 5.82E5
T: FTMS {1,1} + p ESI Full ms [100.00-1000.00]
480.15766
z=1
C26 H27 O6 N P = 480.15705
1.27084 ppm
481.16108
z=1
Relative Abundance
m/z
PhO
OPh
4e

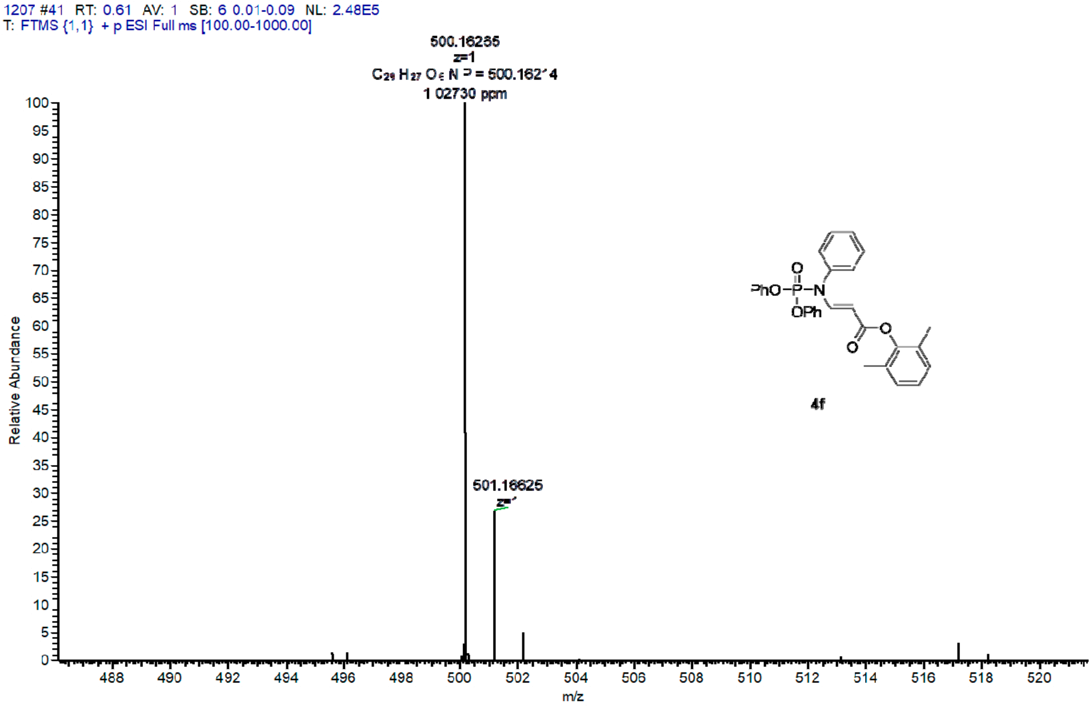
1207 #41 RT: 0.61 AV: 1 SB: 6 0.01-0.09 NL: 2.48E5
T: FTMS {1,1} + p ESI Full ms [100.00-1000.00]
500.16265
z=1
501.16625
Relative Abundance
m/z
OPh
4f

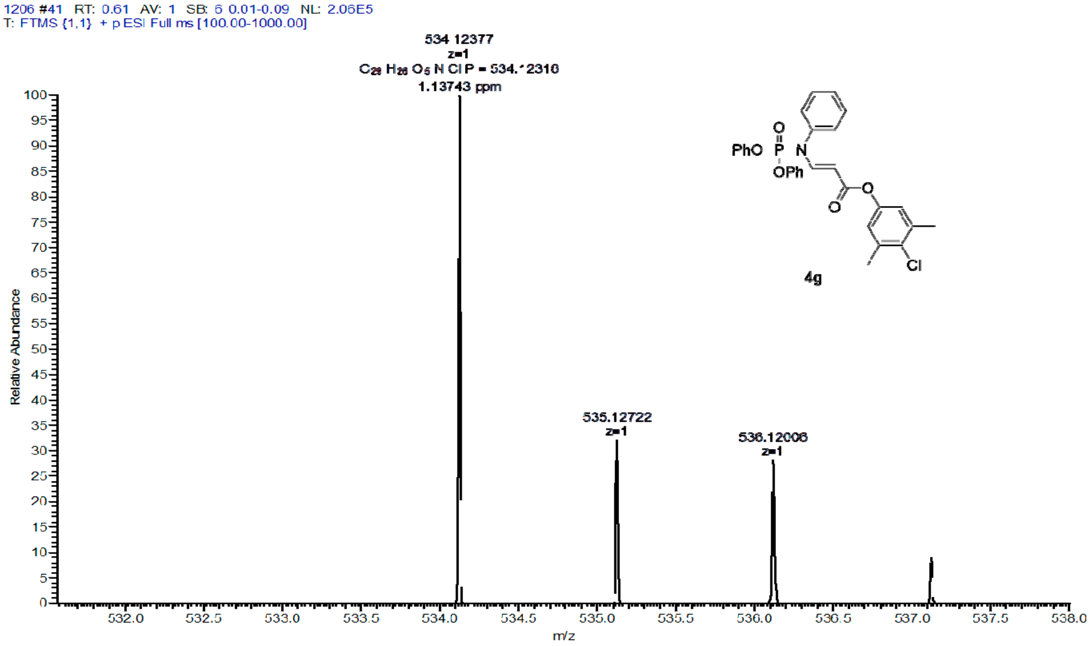
1206 #41 RT: 0.61 AV: 1 SB: 6 0.01-0.09 NL: 2.06E5
T: FTMS {1,1} + p ESI Full ms [100.00-1000.00]
534.12377
z=1
C28 H26 O5 N Cl P = 534.12316
1.13743 ppm
535.12722
z=1
536.12006
z=1
Relative Abundance
m/z
PhO
OPh
Cl
4g

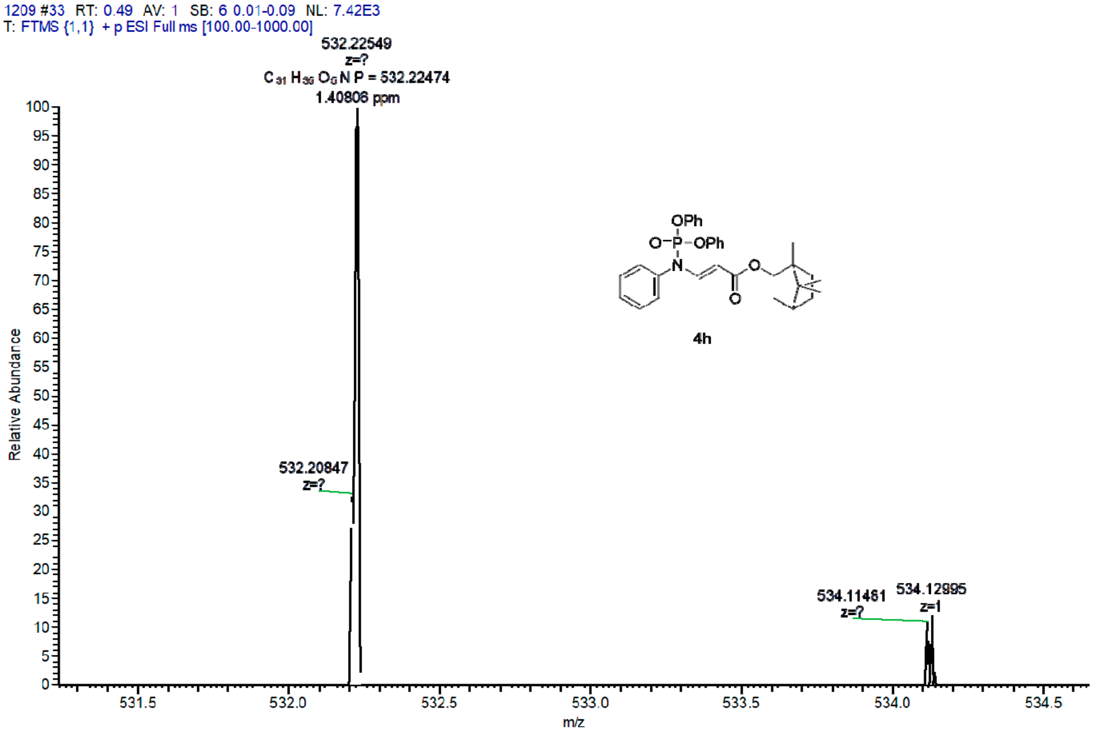
1209 #33 RT: 0.49 AV: 1 SB: 6 0.01-0.09 NL: 7.42E3
T: FTMS {1,1} + p ESI Full ms [100.00-1000.00]
532.22549
z=?
C31 H35 O5 N P = 532.22474
1.40806 ppm
532.20847
z=?
534.11461
z=?
534.12995
z=1
Relative Abundance
m/z
OPh
O-P-OPh
N
4h

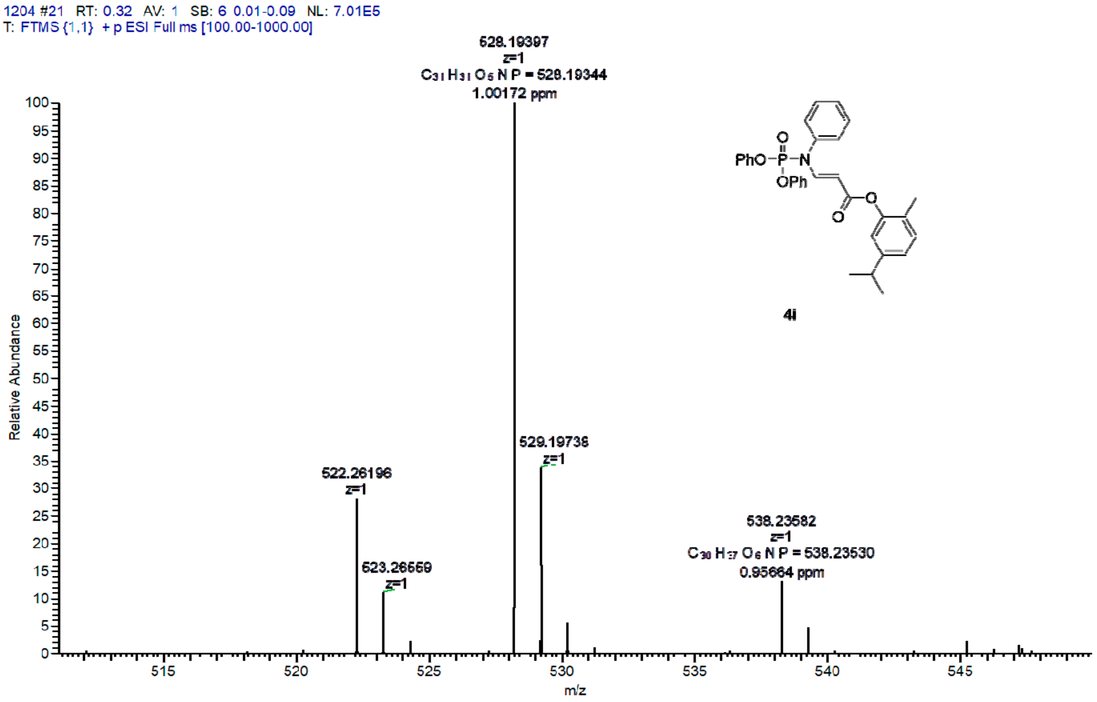
1204 #21 RT: 0.32 AV: 1 SB: 6 0.01-0.09 NL: 7.01E5
T: FTMS {1,1} + p ESI Full ms [100.00-1000.00]
528.19397
z=1
C31 H31 O5 N P = 528.19344
1.00172 ppm
522.26196
z=1
523.26669
z=1
529.19736
z=1
538.23582
z=1
C30 H37 O6 N P = 538.23530
0.95664 ppm
Relative Abundance
m/z
PhO
OPh
4i

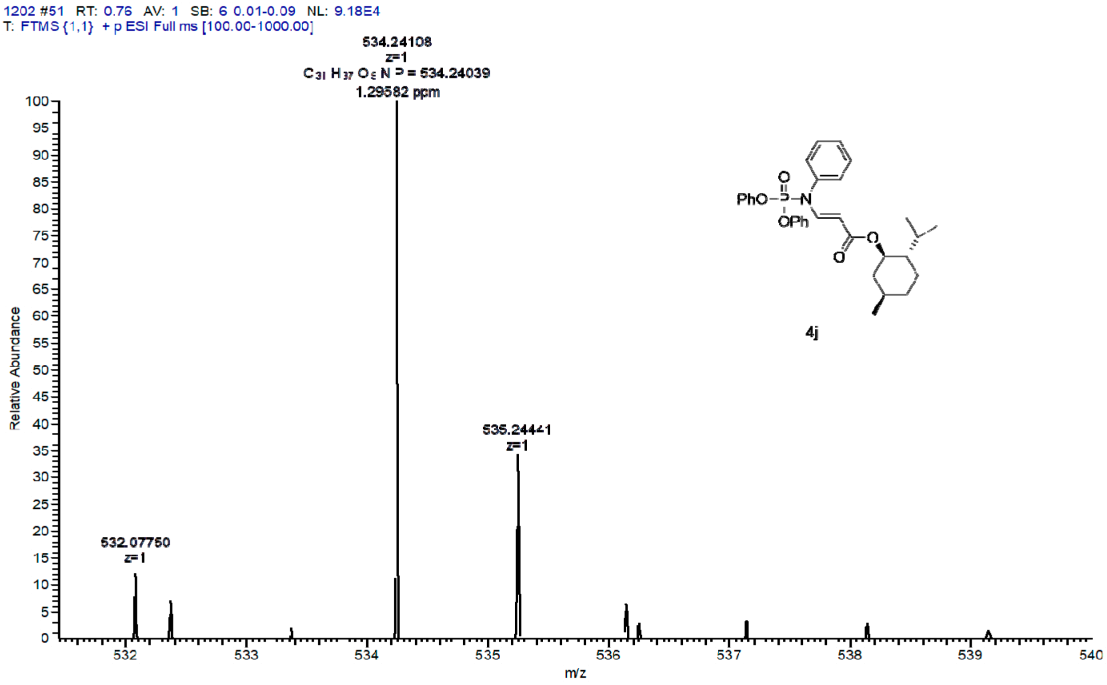
1202 #51 RT: 0.76 AV: 1 SB: 6 0.01-0.09 NL: 9.18E4
T: FTMS {1,1} + p ESI Full ms [100.00-1000.00]
534.24108
z=1
C31 H37 O5 N P = 534.24039
1.29582 ppm
535.24441
z=1
532.07750
z=1
Relative Abundance
m/z
PhO
OPh
4j

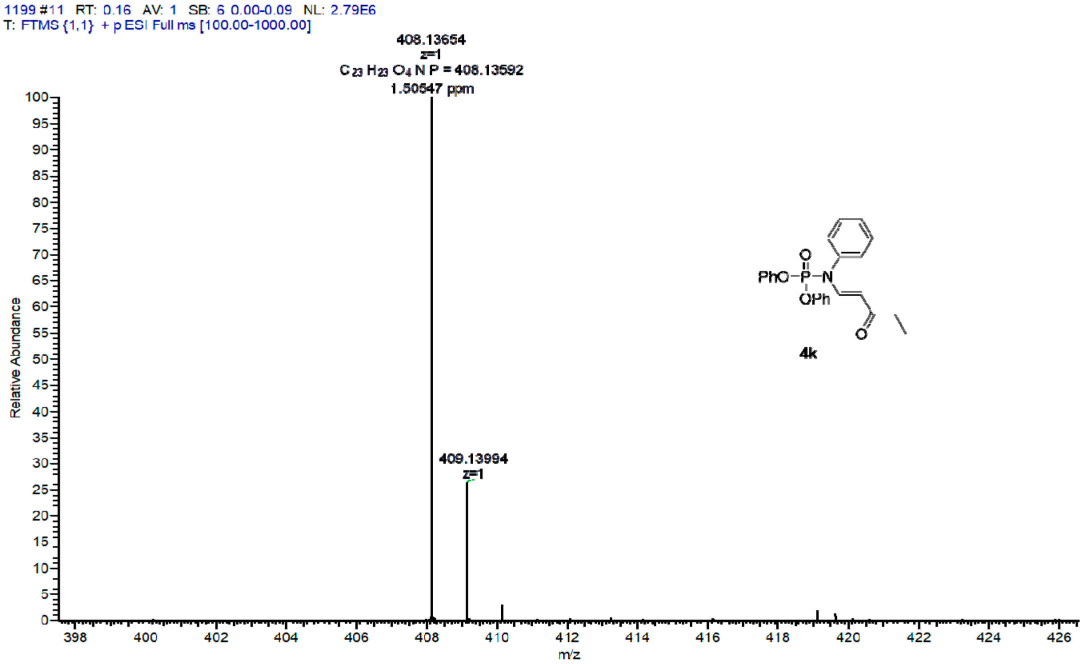
1199 #11 RT: 0.16 AV: 1 SB: 6 0.00-0.09 NL: 2.79E6
T: FTMS {1,1} + p ESI Full ms [100.00-1000.00]
408.13654
z=1
C 23 H 23 O 4 N P = 408.13592
1.50547 ppm
409.13994
z=1
Relative Abundance
m/z
PhO
P
N
O
OPh
O
4k

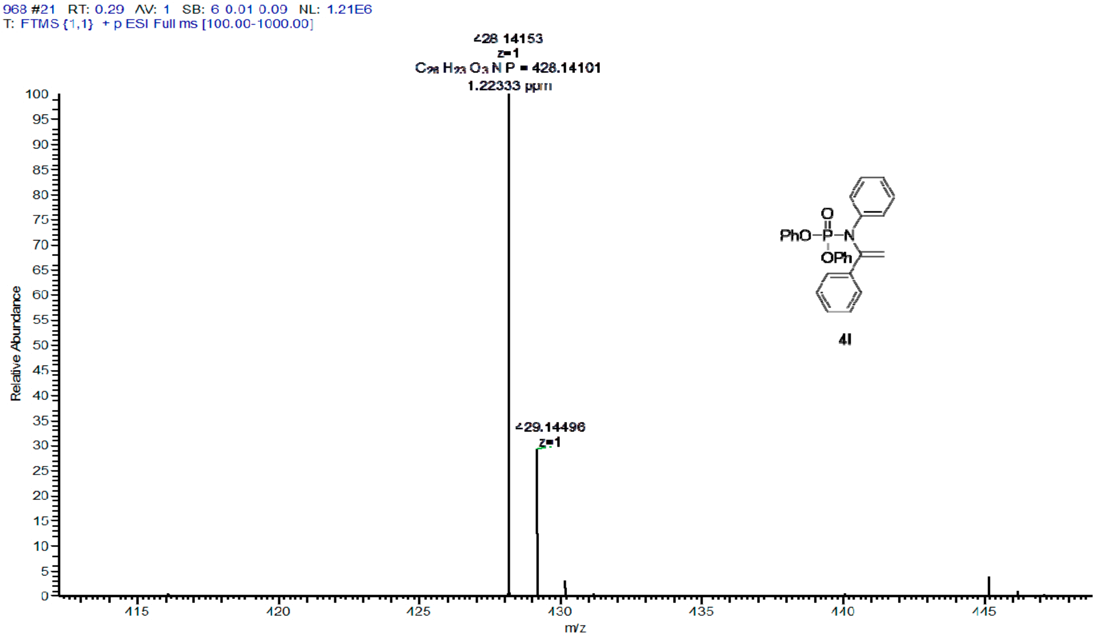
968 #21 RT: 0.29 AV: 1 SB: 6 0.01 0.09 NL: 1.21E6
T: FTMS {1,1} + p ESI Full ms [100.00-1000.00]
428.14153
z=1
C 26 H 23 O 3 N P = 428.14101
1.22333 ppm
429.14496
z=1
Relative Abundance
m/z
PhO
P
N
O
OPh
4l

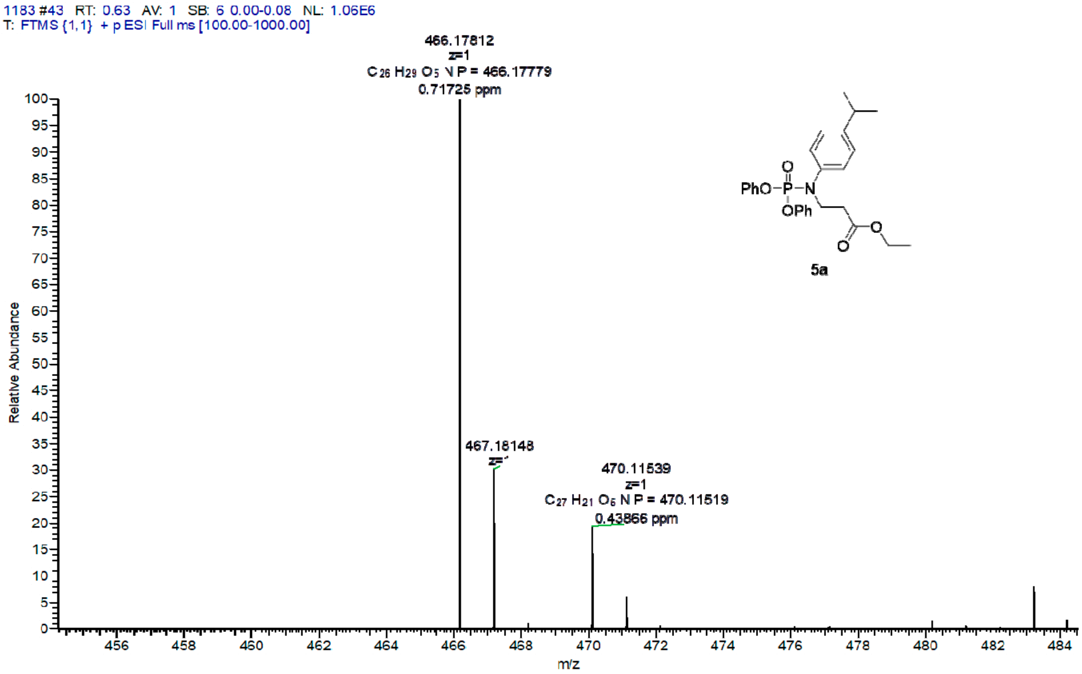
1183 #43 RT: 0.63 AV: 1 SB: 6 0.00-0.08 NL: 1.06E6
T: FTMS {1,1} + p ESI Full ms [100.00-1000.00]
466.17812
z=1
C26 H29 O5 N P = 466.17779
0.71725 ppm
467.18148
470.11539
z=1
C27 H21 O6 N P = 470.11519
0.43866 ppm
Relative Abundance
m/z
PhO–P–N
OPh
O
5a

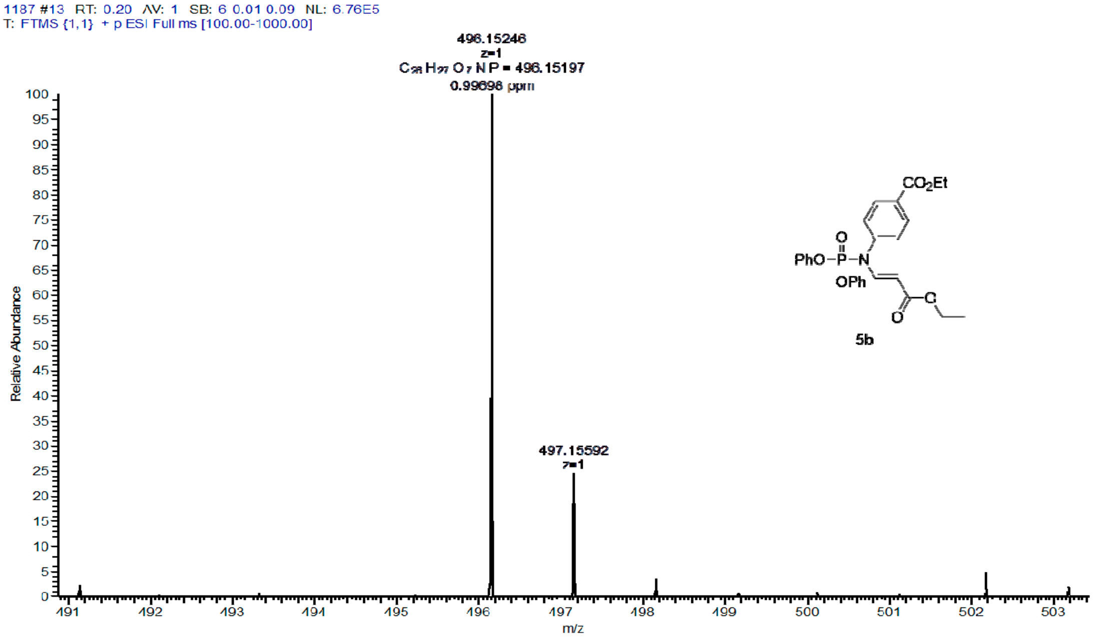
1187 #13 RT: 0.20 AV: 1 SB: 6 0.01 0.09 NL: 6.76E5
T: FTMS {1,1} + p ESI Full ms [100.00-1000.00]
496.15246
z=1
0.99698 ppm
497.15592
z=1
Relative Abundance
m/z
CO2Et
PhO–P–N
OPh
O
5b

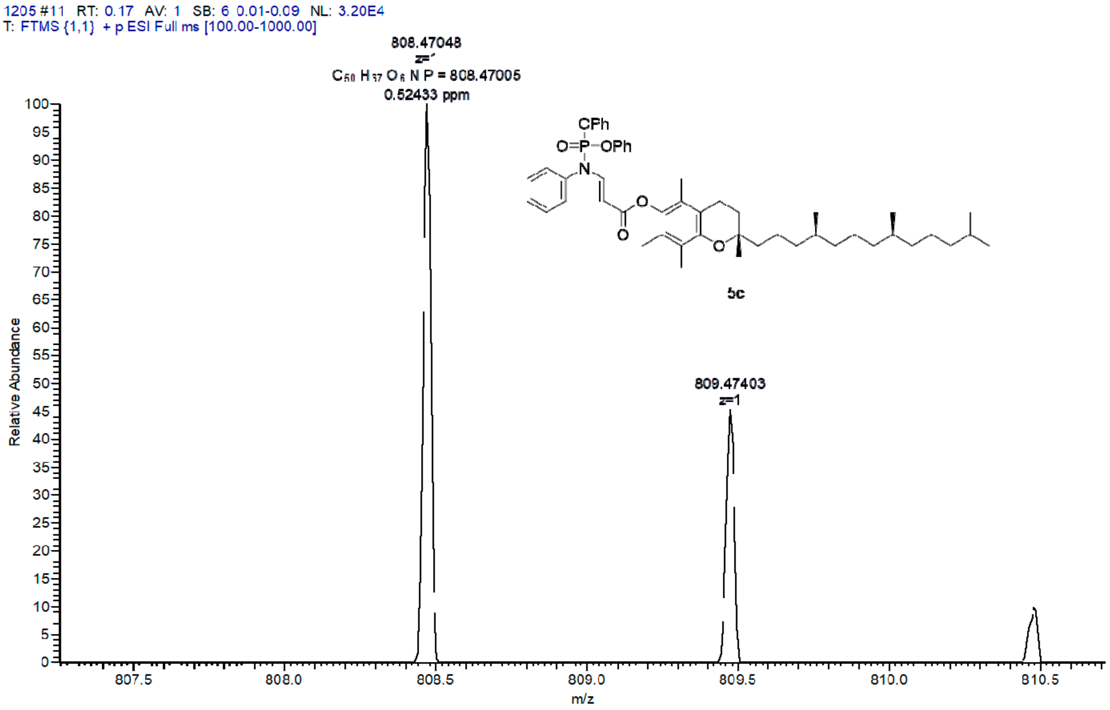
1205 #11 RT: 0.17 AV: 1 SB: 6 0.01-0.09 NL: 3.20E4
T: FTMS {1,1} + p ESI Full ms [100.00-1000.00]
808.47048
C50 H67 O6 N P = 808.47005
0.52433 ppm
809.47403
z=1
Relative Abundance
m/z
OPh
O=P-OPh
N
O
5c
